# Supplementary material for: Quantitative analysis of mammalian translation initiation sites by FACS-seq
Source: Mol Syst Biol. 2014 Aug 28;10(8):748. doi: 10.15252/msb.20145136 (PMC4299517; doi:10.15252/msb.20145136)
Supplement: Supplementary file 1 — Supplementary Figures S1-S6 and Table S6 [file msb0010-0748-SD1.pdf]

Supplementary information

Table of contents:

Supplementary Figure S1. Dual fluorescence readout by TIS reporter
Supplementary Table S1. Dinucleotide PWM of TIS efficiency
Supplementary Figure S2. Comparison of TIS efficiency measured using multiple cell lines, reporter genes, and growth conditions
Supplementary Figure S3. Effect of mRNA secondary structure on TIS efficiency
Supplementary Figure S4. Comparison of -3R +4G TIS motif versus proposed TIS motif
Supplementary Figure S5. Kullback-Leibler divergence of the TIS efficiency distributions
Supplementary Figure S6. Leaky scanning model for in-frame AUG start codons
Supplementary Table S6. FACS-seq primers

Legends only
Supplementary Table S2. TIS efficiency reference table
Supplementary Table S3. TIS mutations in the COSMIC database
Supplementary Table S4. Human genes with predicted translational truncation isoforms
Supplementary Table S5. Mouse genes with predicted translational truncation isoforms
Supplementary Figure S7. Scoring of ribosome footprint profiles

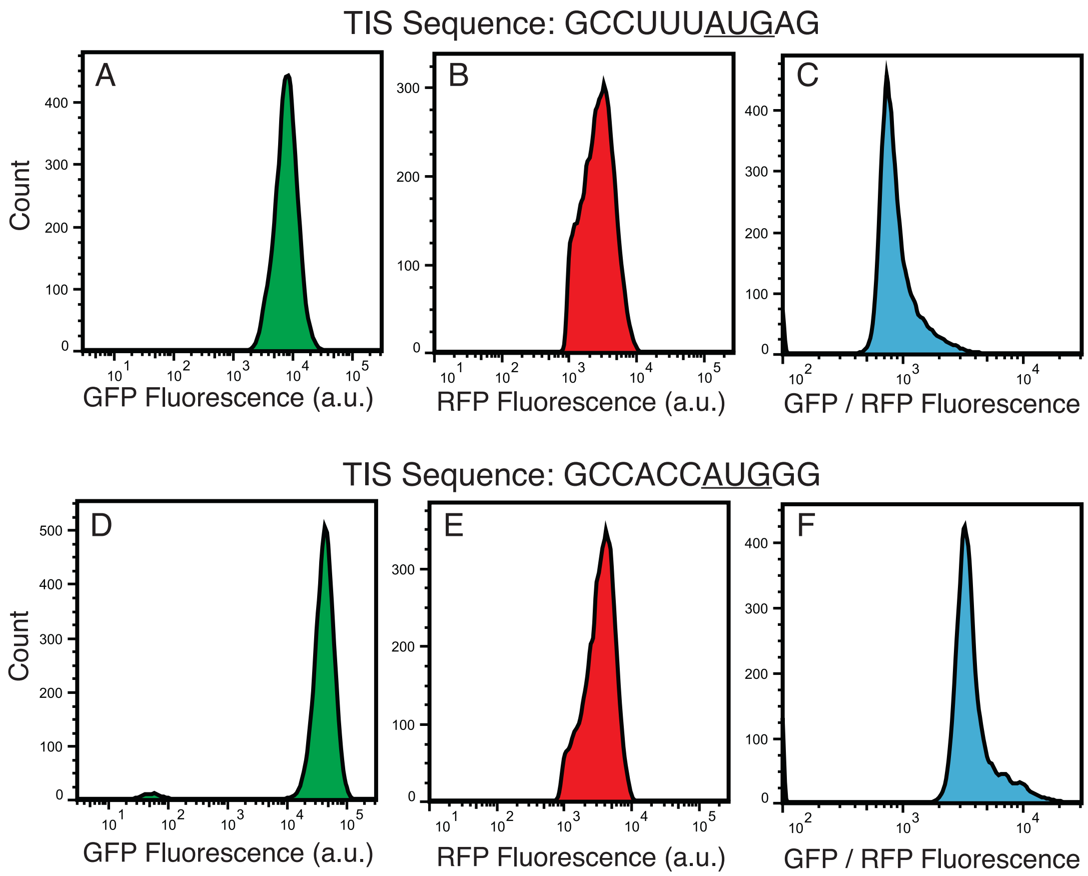

Supplementary **Figure S1. Dual fluorescence readout by TIS reporter** GFP and RFP were expressed from a single mRNA transcript. **(A)** GFP expression from the TIS sequence GCCUUUAUGAG. **(B)** RFP expression from the TIS sequence GCCACCAUGGU. GFP and RFP were separated by an IRES causing RFP translation to be independent from GFP translation. **(C)** GFP expression normalized by RFP expression for the purpose of reducing extrinsic noise (e.g., variation in transcript copy number). **(D-F)** Same as (A-C) except the GFP expression utilized the TIS sequence GCCACCAUGGG.

Supplementary **Table S1 Dinucleotide PWM of TIS efficiency** Normalized values of the dinucleotide PWM ($\exp(C_{b,i})$ and $\exp(C_{b1,i1,b2,i2})$). The values were normalized such that the diagonal values were consistent with the mononucleotide PWM. The off-diagonal values represent the cooperativity between TIS positions. Efficiency values were calculated by multiplying the intercept (79.5) by all of the appropriate base/position values. Values above 1.00 enhance TIS efficiency (red); values below 1.00 reduce TIS efficiency (blue).

Position	-6				-5				-4				-3				-2				-1				+4				+5			
	U	C	A	G	U	C	A	G	U	C	A	G	U	C	A	G	U	C	A	G	U	C	A	G	U	C	A	G	U	C	A	G
-6 U	0.98				1.00	1.03	1.03	1.00	1.07	1.01	1.00	0.94	0.93	0.97	0.99	0.99	1.00	1.03	1.00	0.99	1.01	0.98	0.99	0.97	1.03	1.07	1.00	0.98	1.01	1.00	1.01	0.99
-6 C		0.97			1.00	0.99	1.03	0.98	1.03	0.99	1.00	0.94	0.94	0.98	0.99	0.99	0.98	1.03	1.00	1.00	1.01	1.00	0.99	1.01	1.00	1.03	1.01	0.97	1.04	1.03	1.01	1.00
-6 A			1.00		1.02	1.03	1.02	1.03	1.01	1.00	0.98	1.02	1.02	1.02	0.98	1.00	0.99	0.98	0.98	1.00	1.00	0.99	0.98	0.99	0.99	1.03	0.99	0.96	1.02	0.99	1.00	0.99
-6 G				1.05	0.93	0.96	1.02	0.95	1.05	0.98	0.99	1.00	1.14	1.07	0.98	1.02	1.01	0.99	0.99	1.02	1.10	1.00	0.98	1.00	0.97	1.04	0.99	0.95	1.01	0.96	1.00	0.92
-5 U					1.03				1.01	0.99	0.99	1.02	1.08	1.05	0.96	0.96	0.99	0.99	0.97	1.07	1.02	1.01	0.99	0.98	1.00	1.06	1.00	0.98	1.05	0.95	1.01	0.96
-5 C						1.04			0.98	0.95	1.00	1.04	1.09	1.09	0.97	0.93	0.98	1.00	0.97	1.09	1.01	1.00	1.00	0.98	0.96	1.00	1.01	0.95	1.06	0.94	1.01	0.94
-5 A							0.94		1.01	1.01	0.99	1.02	1.00	1.00	0.96	0.98	0.97	0.96	0.97	0.98	1.02	1.01	0.99	1.00	1.00	1.04	1.00	0.97	1.03	1.00	1.01	0.99
-5 G								0.99	1.01	0.98	1.01	0.97	1.02	1.01	0.97	0.95	1.01	0.98	0.98	1.09	1.01	0.99	1.00	0.99	0.98	1.05	1.01	0.98	1.06	0.96	1.02	1.00
-4 U									0.91				0.87	0.94	1.05	0.98	0.98	1.06	1.01	0.97	0.89	0.94	1.01	1.08	1.05	1.02	0.99	1.00	0.97	1.07	0.99	1.01
-4 C										1.08			1.06	1.02	1.04	0.96	1.05	0.96	1.00	1.01	1.01	0.98	1.00	1.06	1.00	1.05	0.98	0.94	0.98	1.00	0.98	0.98
-4 A											1.07		1.07	1.07	1.03	1.05	1.00	0.98	0.99	1.00	1.01	1.00	0.99	1.00	0.97	1.01	0.97	0.94	0.99	0.96	0.97	0.95
-4 G												0.94	0.86	0.95	1.06	0.99	0.96	0.98	1.02	1.03	0.93	1.02	1.02	1.06	1.02	1.04	1.00	1.02	1.03	1.08	1.00	1.04
-3 U													0.71				0.89	1.16	1.07	0.74	0.79	0.92	1.08	0.97	1.12	0.92	1.00	1.13	0.87	1.24	0.98	1.11
-3 C														0.92			0.97	1.01	1.07	0.84	0.84	0.98	1.07	0.99	1.06	0.95	1.00	1.02	0.91	1.08	0.98	1.06
-3 A															1.24		1.03	1.02	1.02	1.04	1.06	1.05	1.03	1.05	0.96	1.00	0.96	0.93	0.97	0.94	0.94	0.93
-3 G																1.13	1.02	1.03	1.05	1.03	1.03	1.04	1.05	1.03	1.01	0.97	0.98	0.99	1.00	1.02	0.96	1.00
-2 U																	0.99				1.03	1.12	0.97	1.00	1.02	1.01	1.00	0.99	1.00	0.99	1.00	0.99
-2 C																		1.08			1.05	1.00	0.96	0.98	0.96	0.99	0.99	0.92	1.02	0.98	0.99	1.01
-2 A																			1.05		0.99	0.98	0.97	0.98	0.99	1.04	0.99	0.96	1.02	0.99	0.99	0.98
-2 G																				0.88	1.06	0.95	0.98	0.97	1.06	1.03	1.00	1.05	0.98	1.05	1.01	1.01
-1 U																					0.90				1.06	1.01	1.02	0.97	0.98	1.07	1.01	1.03
-1 C																						1.00			1.01	1.05	1.01	0.94	1.00	1.01	1.01	0.99
-1 A																							1.08		0.99	1.04	0.99	0.96	1.01	0.98	0.99	0.97
-1 G																								1.02	0.99	1.01	1.01	0.95	0.99	0.98	1.00	0.97
+4 U																									1.06				1.03	0.78	1.02	0.93
+4 C																										0.91			0.92	0.68	1.06	0.89
+4 A																											0.98		1.04	1.01	1.01	1.00
+4 G																												1.04	0.98	1.31	0.98	1.36
+5 U																													0.93			
+5 C																														1.09		
+5 A																															0.99	
+5 G																																0.99

Intercept 79.5

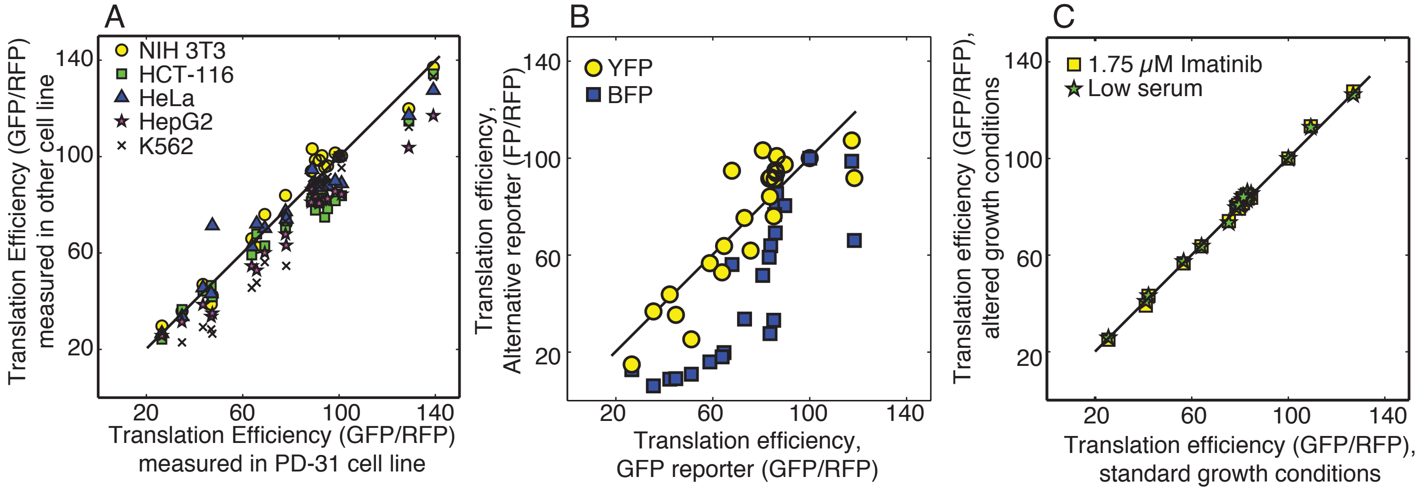

Supplementary **Figure S2. Comparison of TIS efficiency measured using multiple cell lines, reporter genes, and growth conditions** Cells were stably transduced with a single TIS reporter construct and were analyzed by conventional flow cytometry. **(A)** TIS efficiency values measured in various cell lines (R^2 = 0.92, $p = 1.1\cdot10^{-90}$) **(B)** Comparison of TIS efficiency values measured using a GFP, mCitrine (YFP), or mTagBFP (BFP) reporter. Measurements were from PD-31 cells (YFP: R^2 = 0.76, $p = 1.1\cdot10^{-19}$, BFP: R^2 = 0.39, $p = 2.1\cdot10^{-11}$). **(C)** Comparison of TIS efficiency values from PD-31 cells cultured with standard growth conditions (10% serum), 1.75 μM Imatinib, or low serum (1%). The PD-31 cell line is Abelson-transformed. Addition of imatinib selectively inhibits the v-Abelson tyrosine kinase.

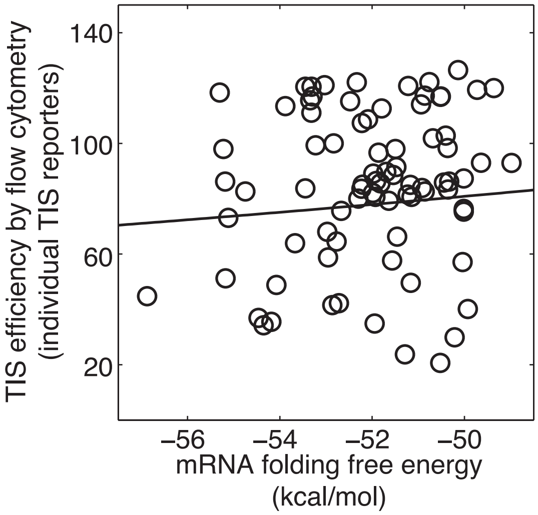

Supplementary **Figure S3. Effect of mRNA secondary structure on TIS efficiency** The mRNA folding free energy was calculated for 65,536 mRNA sequences, each sequence consisting of an 11 base TIS sequence, 70 bases upstream of the TIS, and 70 bases downstream of the TIS. The folding free energies were compared to TIS efficiency values from PD-31 cells expressing a single TIS reporter and analyzed by conventional flow cytometry. Solid line represents linear regression model of the folding free energy vs. the natural logarithm of the TIS efficiency as measured by FACS-seq (R^2 = 0.02; p = 0.18).

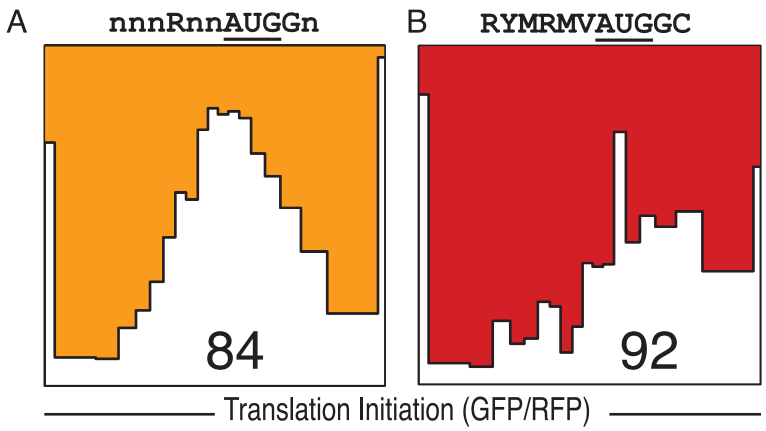

Supplementary **Figure S4. Comparison of -3R +4G TIS motif versus proposed TIS motif (A)** Composite FACS-seq histograms of all TIS sequences with a purine in the -3 and a G in the +4. **(B)** Composite FACS-seq histograms of all TIS sequences that fit our proposed TIS motif. The background color corresponds to the median TIS efficiency labeled on each histogram. n = U, C, A, or G; R = A or G; Y = U or C; M = A or C; V = C, A, or G.

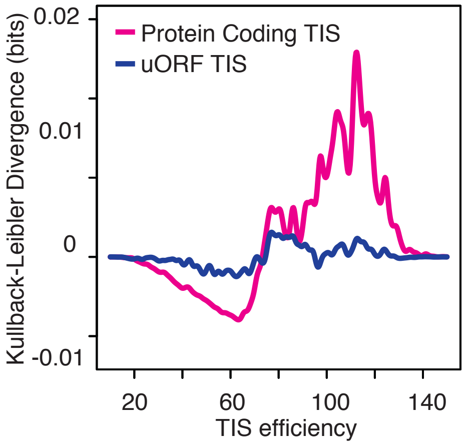

Supplementary **Figure S5. Kullback-Leibler divergence of the TIS efficiency distributions** The TIS efficiency distributions of the protein coding TISs and of the uORF TISs were both compared to the distribution of all possible TIS sequence (i.e., TIS sequence space). The divergence of these distributions was calculated using the Kullback-Leibler formula $D_{KL}(P||Q) = \Sigma_i \log_2[P(i)/Q(i)] \cdot P(i)$ where Q(i) is the efficiency distribution of the TIS sequence space and P(i) is either the protein coding TIS efficiency distribution (D_{KL} = 0.24) or the uORF TIS efficiency distribution (D_{KL} = 0.005).

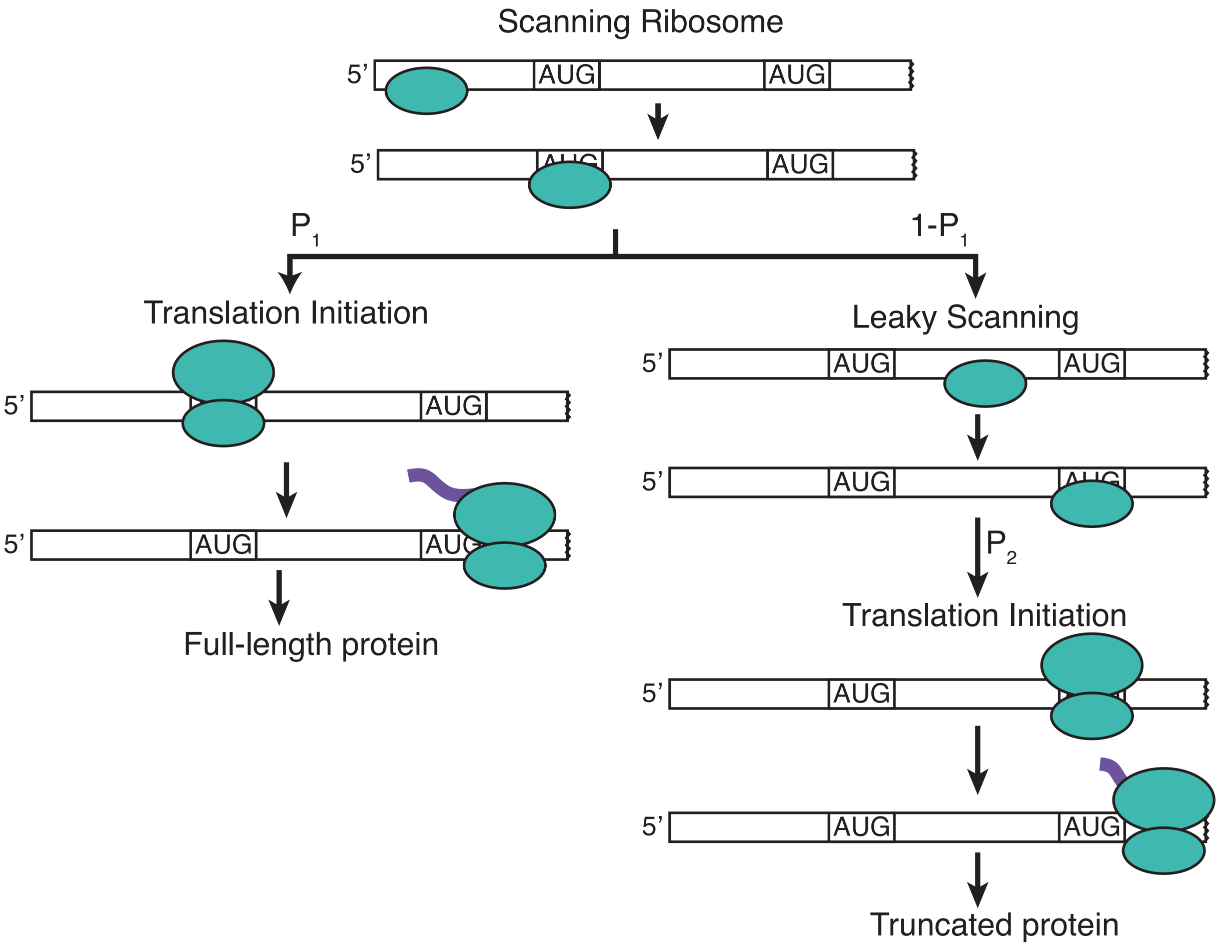

Supplementary **Figure S6. Leaky scanning model for in-frame AUG start codons** The 43S preinitiation complex consisting of the small
40S ribosomal subunits, Met-tRNA, and initiation factors begins scanning from a transcript's 5'-cap. Upon encountering the first AUG start codon, the ribosome initiates with a probability P1 and leaks past with a probability (1-P1). The leaky scanning allows for initiation at a downstream, in-frame start codon, where the ribosome initiates with a probability P2. Initiation at the first start codon results in the translation of a full-length protein, while initiation at the second start codon results in the translation of a truncated protein isoform. The probability of initiation, P, is related to the TIS efficiency value, E, by the equation P = k•E/100, where k = 0.86 (Ferreira et al, 2013). 43S preinitiation complex, small blue oval; 60S subunit, large blue oval; protein, purple squiggle.

Supplementary **Table S7. FACS-seq primers** Primer sets used to PCR-amplify the genomic region containing the TIS sequence. Barcodes were used to identify from which gate the sample was sorted.

First PCR

Forward Primers	Sequence
BarCode-01	CTACACGACGCTCTTCCGATCT CAAC CCTCGCCCTTGCTGAATTCG
BarCode-02	CTACACGACGCTCTTCCGATCT TAAT CCTCGCCCTTGCTGAATTCG
BarCode-03	CTACACGACGCTCTTCCGATCT GAAG CCTCGCCCTTGCTGAATTCG
BarCode-04	CTACACGACGCTCTTCCGATCT CACA CCTCGCCCTTGCTGAATTCG
BarCode-05	CTACACGACGCTCTTCCGATCT TACC CCTCGCCCTTGCTGAATTCG
BarCode-07	CTACACGACGCTCTTCCGATCT AACG CCTCGCCCTTGCTGAATTCG
BarCode-08	CTACACGACGCTCTTCCGATCT TATA CCTCGCCCTTGCTGAATTCG
BarCode-09	CTACACGACGCTCTTCCGATCT GATC CCTCGCCCTTGCTGAATTCG
BarCode-10	CTACACGACGCTCTTCCGATCT AATT CCTCGCCCTTGCTGAATTCG
BarCode-11	CTACACGACGCTCTTCCGATCT CATG CCTCGCCCTTGCTGAATTCG
BarCode-12	CTACACGACGCTCTTCCGATCT GAGA CCTCGCCCTTGCTGAATTCG
BarCode-13	CTACACGACGCTCTTCCGATCT AAGC CCTCGCCCTTGCTGAATTCG
BarCode-14	CTACACGACGCTCTTCCGATCT CAGT CCTCGCCCTTGCTGAATTCG
BarCode-15	CTACACGACGCTCTTCCGATCT TAGG CCTCGCCCTTGCTGAATTCG
BarCode-16	CTACACGACGCTCTTCCGATCT CCAA CCTCGCCCTTGCTGAATTCG
BarCode-17	CTACACGACGCTCTTCCGATCT GCAT CCTCGCCCTTGCTGAATTCG
BarCode-18	CTACACGACGCTCTTCCGATCT ACAG CCTCGCCCTTGCTGAATTCG
BarCode-19	CTACACGACGCTCTTCCGATCT TCCA CCTCGCCCTTGCTGAATTCG
BarCode-20	CTACACGACGCTCTTCCGATCT ACCT CCTCGCCCTTGCTGAATTCG
**Reverse Primer**	**Sequence**
Cru5-seq3	TAAGAACCTAGAACCTCGCTGGAAAGG

Second PCR

Name	Sequence
P7 Adapter	CAAGCAGAAGACGGCATACGAGCTCTTCCGATCTTAAGAACCTAGAACCTCGCTGGAAAG
P5 Adapater	AATGATACGGCGACCACCGAGATCTACACTCTTTCCCTACACGACGCTCTTCCGATC

Supplementary **Table S2. TIS efficiency reference table** TIS efficiency values for all 65,536 possible AUG containing TIS sequence. Values were obtained with the dinucleotide PWM. 95% confidence intervals are provided.

Supplementary **Table S3. TIS mutations in the COSMIC database** All mutations in the TIS sequences (-6 to +5, excluding AUG start codon) from the Catalogue of Somatic Mutations in Cancer (COSMIC). The predicted change in TIS efficiency was calculated using the dinucleotide PWM.

Supplementary **Table S4. Human genes with predicted translational truncation isoforms** Human genes where at least one transcript had a predicted initiation ratio, X, > 0.50. The pair of TIS sequences and distance between TISs are provided.

Supplementary **Table S5. Mouse genes with predicted translational truncation isoforms** All mouse genes with a possible truncation TIS isoform. The TIS sequences and the predicted initiation ratio, X, are provided with the filtering criteria used when analyzing the ribosome footprint profiling data: mean number of Harringtonine reads at the annotated TIS, number of mRNA isoforms, and distance between TISs.

Supplementary **Figure S7. Scoring of ribosome footprint profiles** All transcripts used in scoring ribosome footprintSprofiling data are included with the ribosome density traces with (+H) and without (-H) Harringtonine treatment. The location of the annotated TIS and putative truncation TIS are indicated by vertical lines. The sequences of the TISs are indicated below the footprinting traces. Gene names, transcript names, predicted initiation ratios, and scoring results are provided.
