# Supplementary figures and images for: Quantitative analysis of mammalian translation initiation sites by FACS-seq
Source: Mol Syst Biol. 2014 Aug 28;10(8):748. doi: 10.15252/msb.20145136 (PMC4299517; doi:10.15252/msb.20145136)

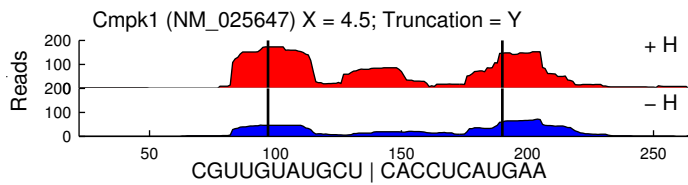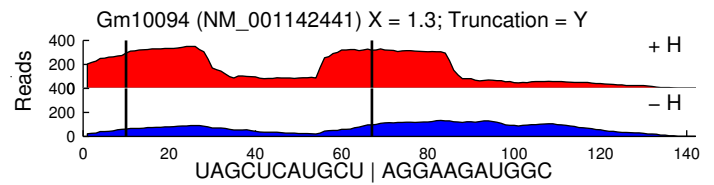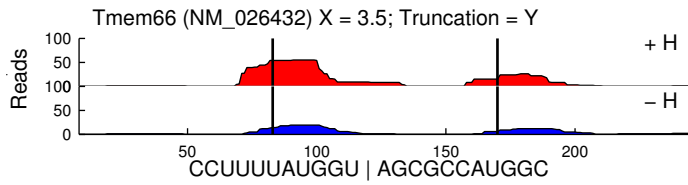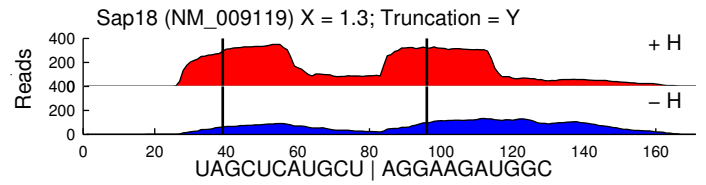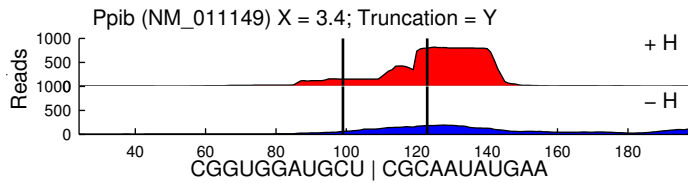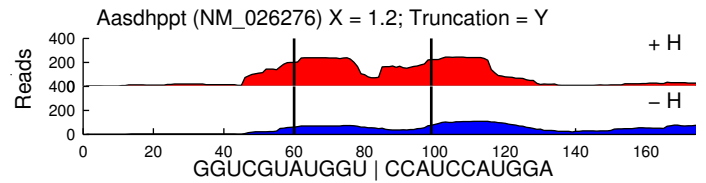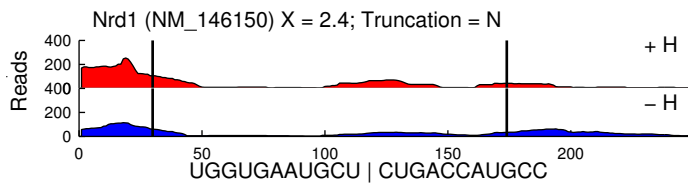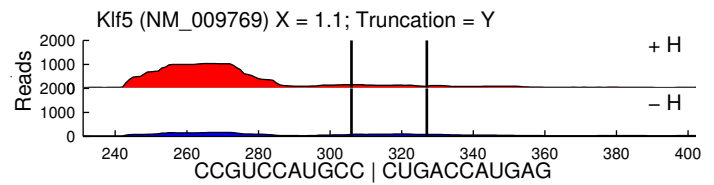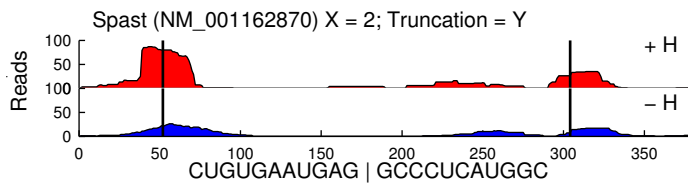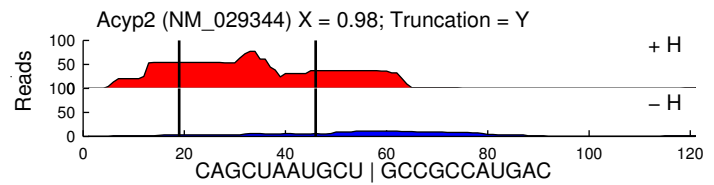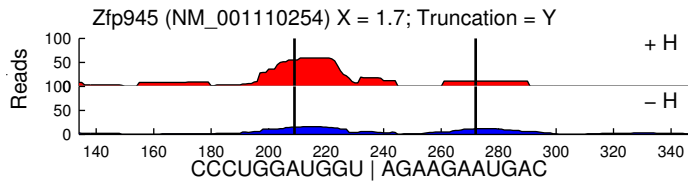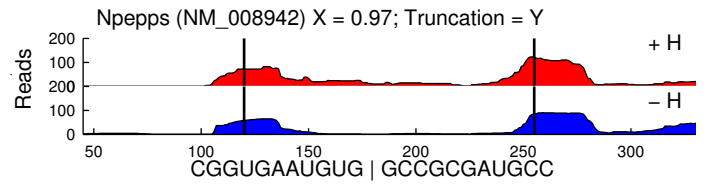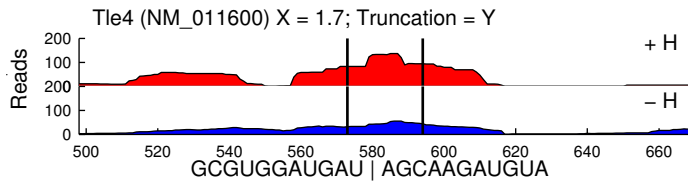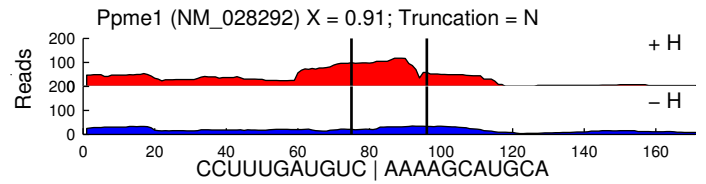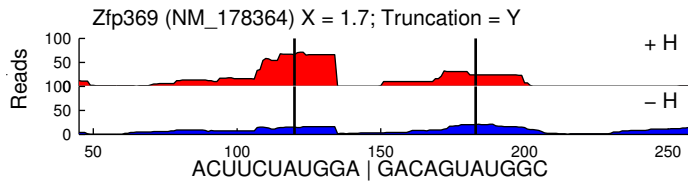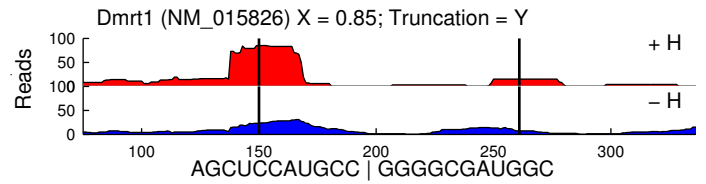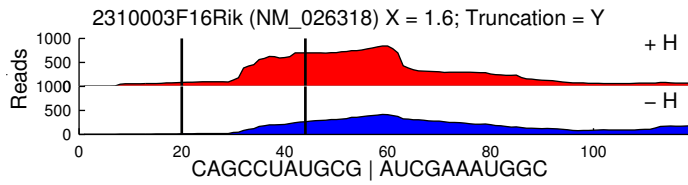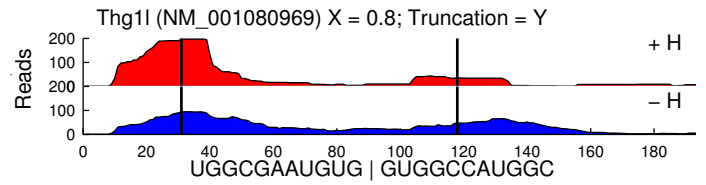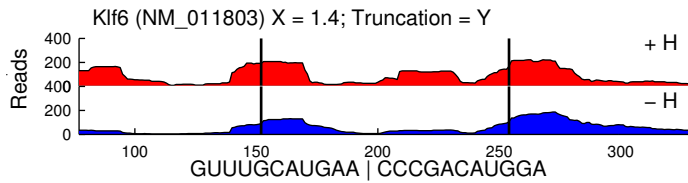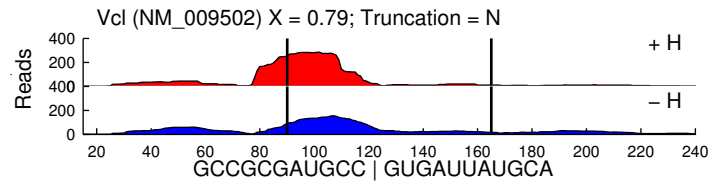

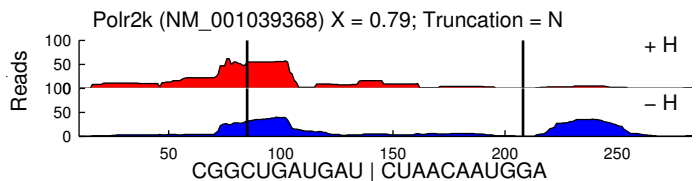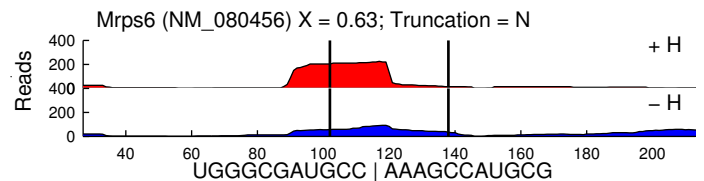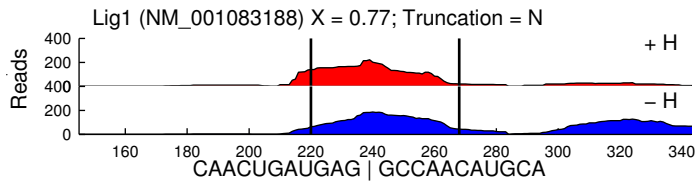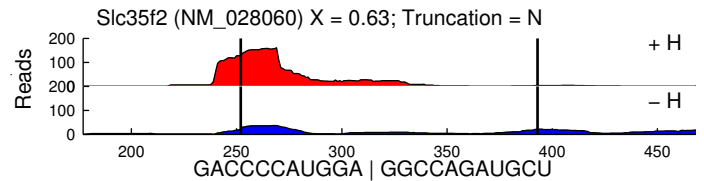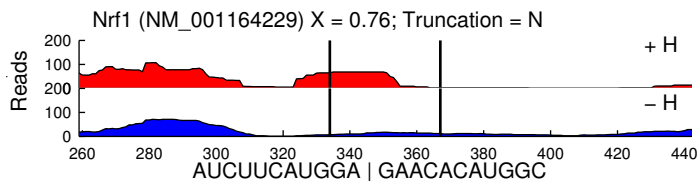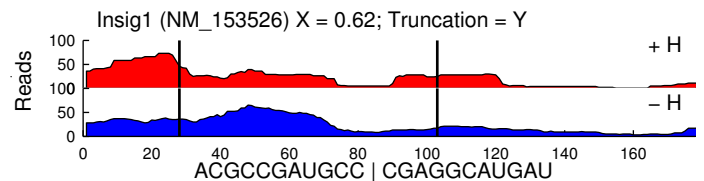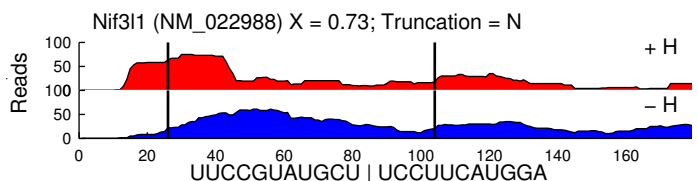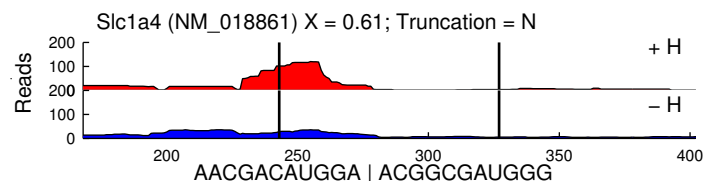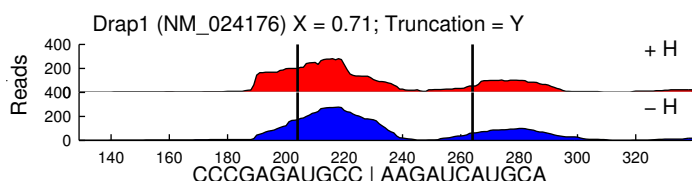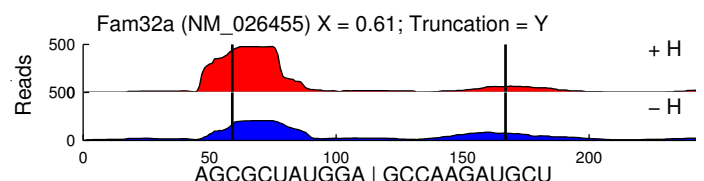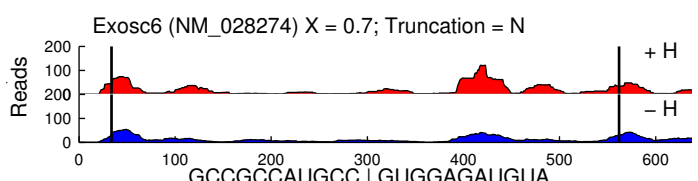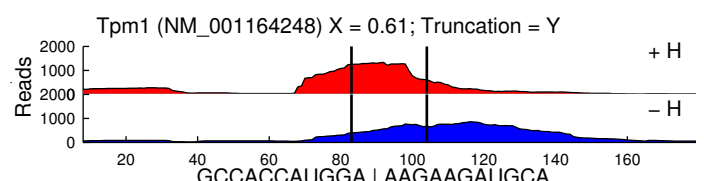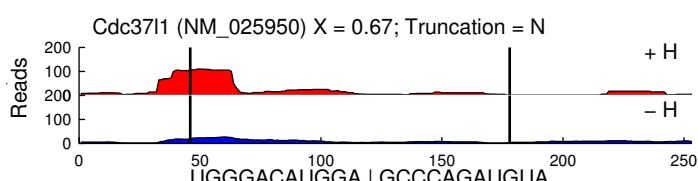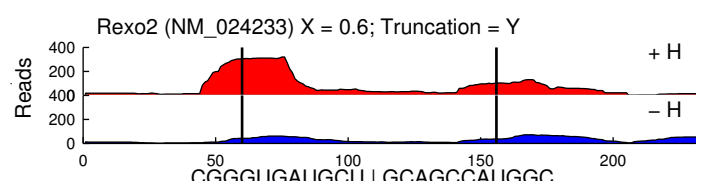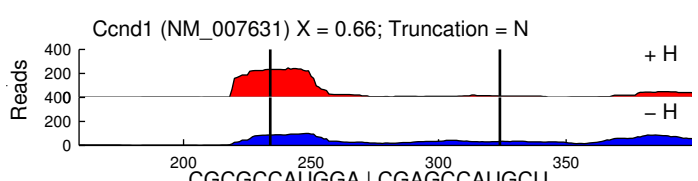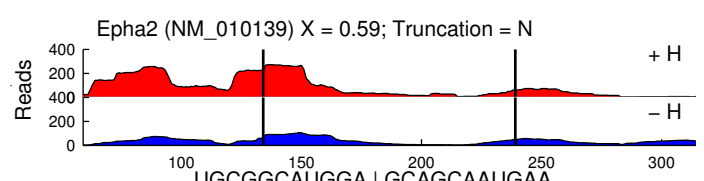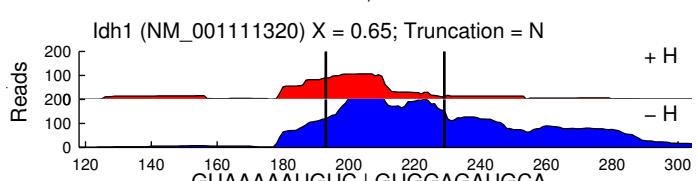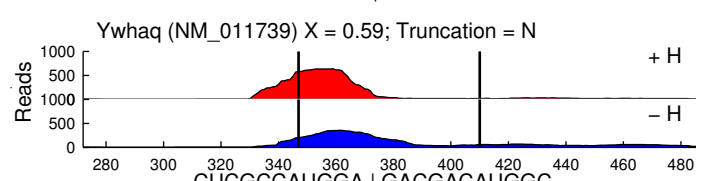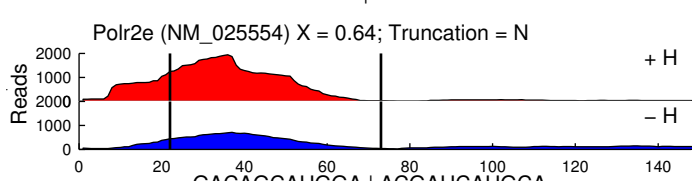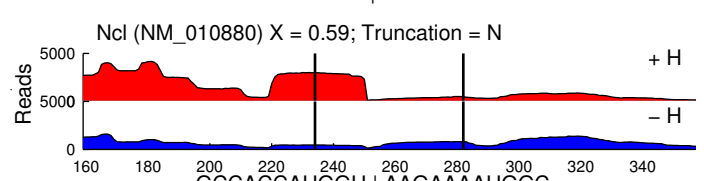

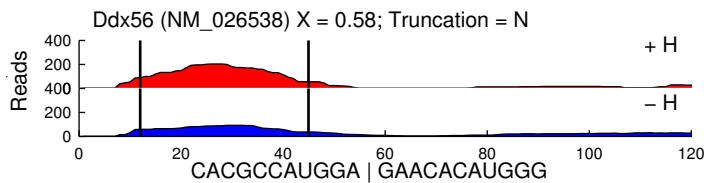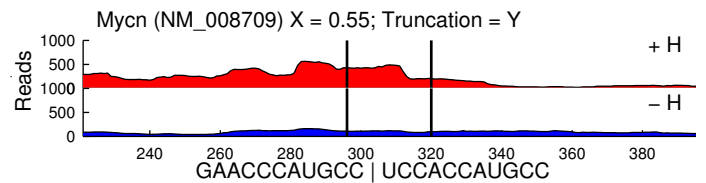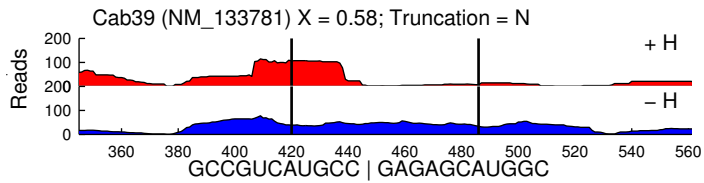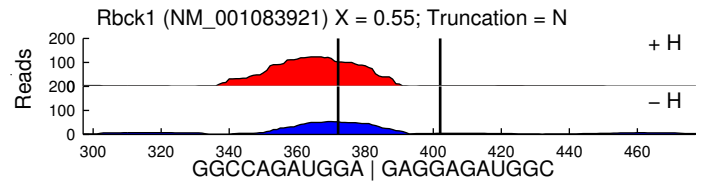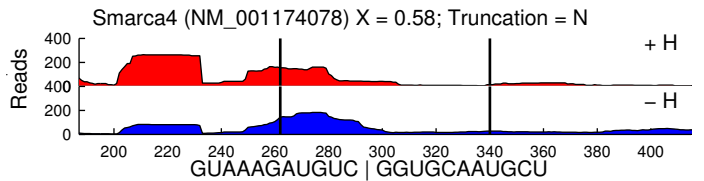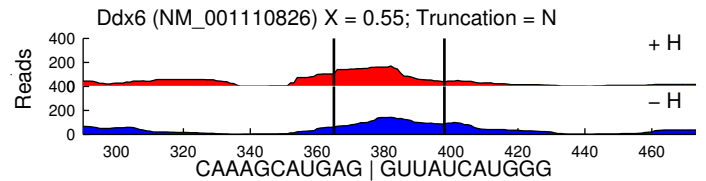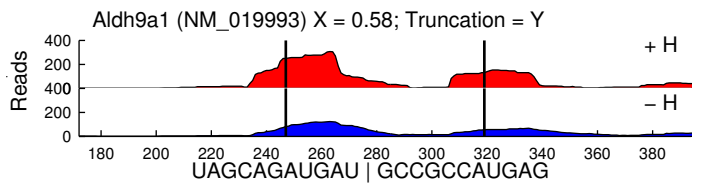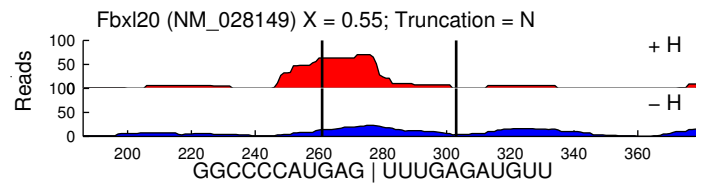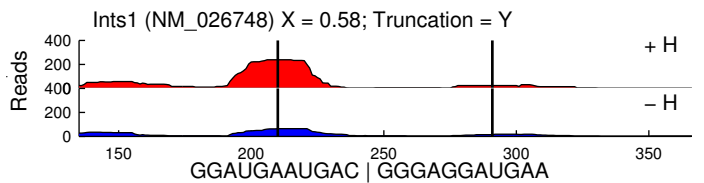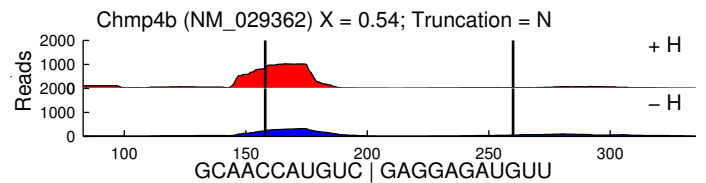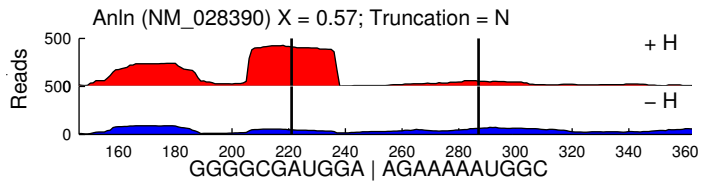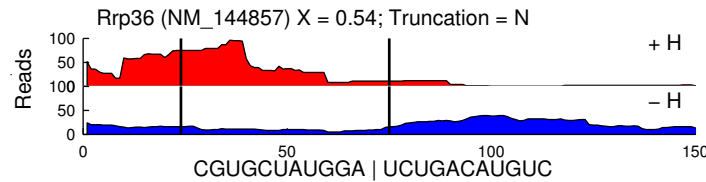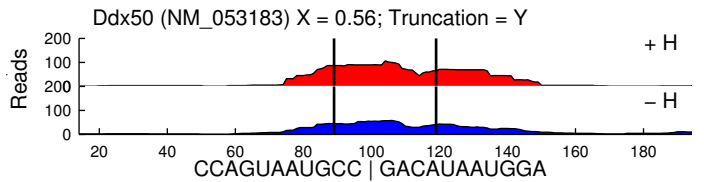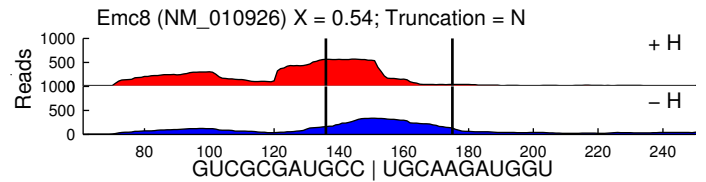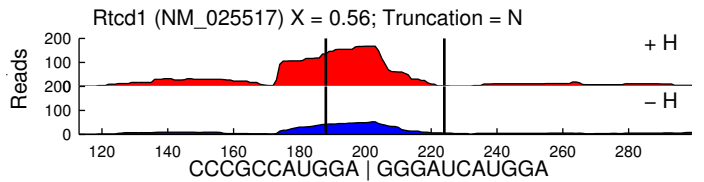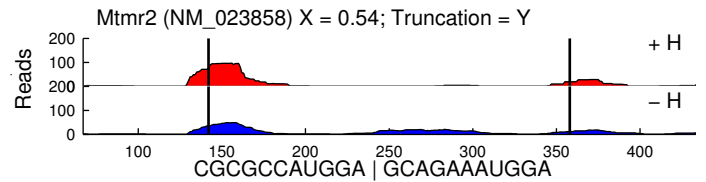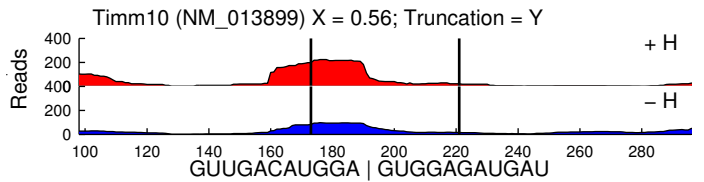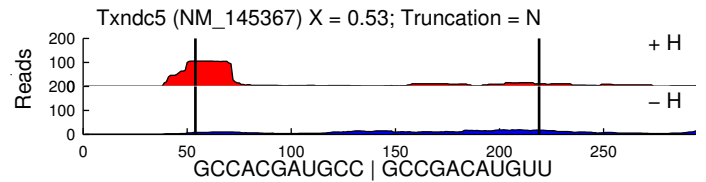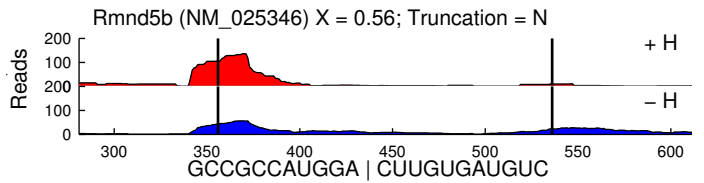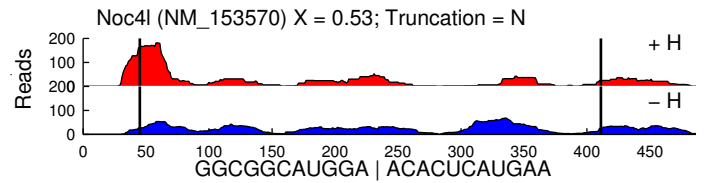

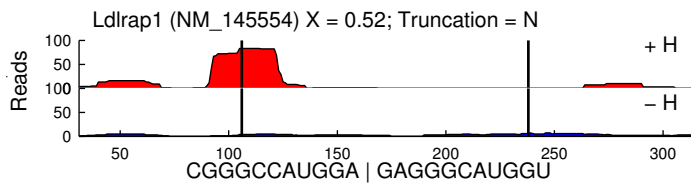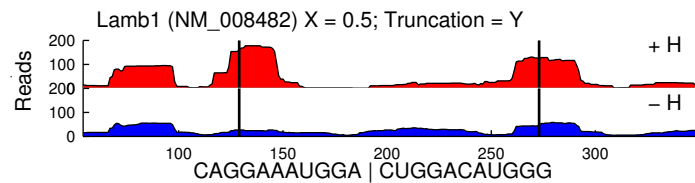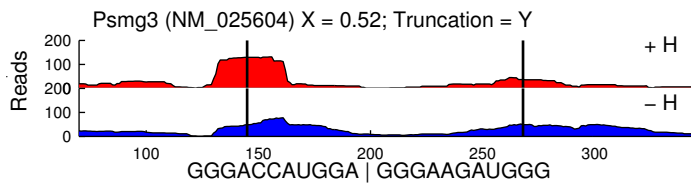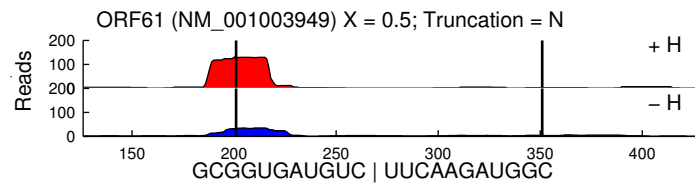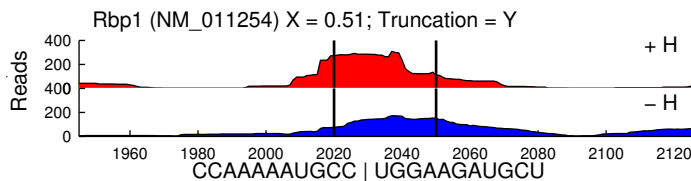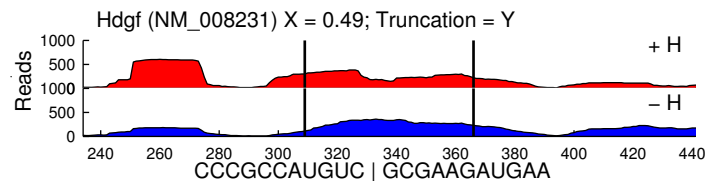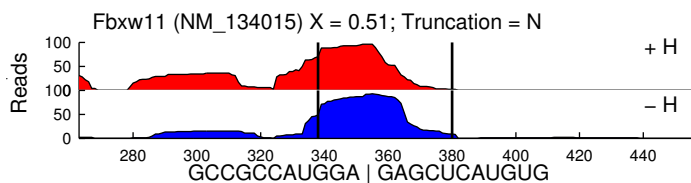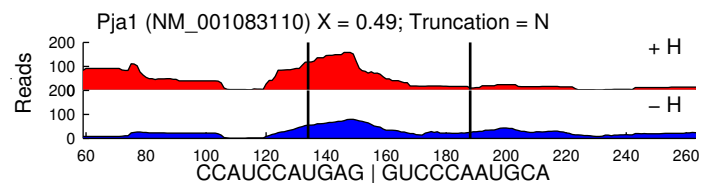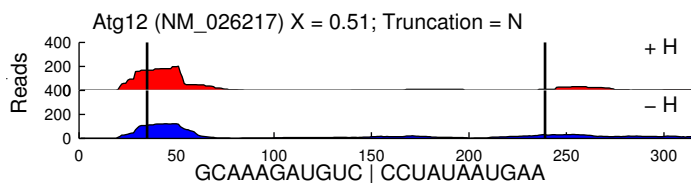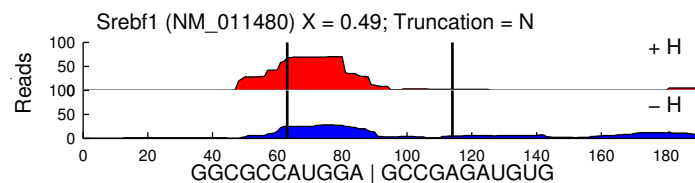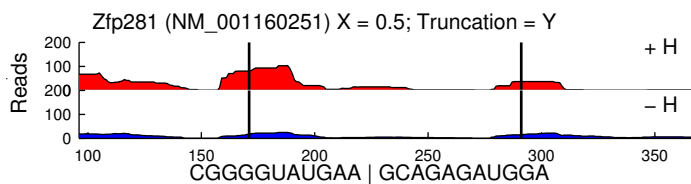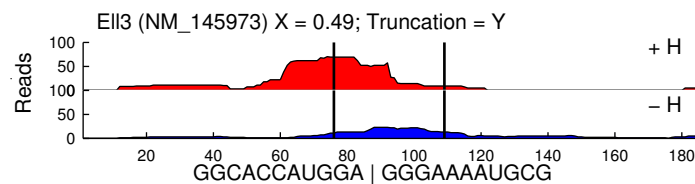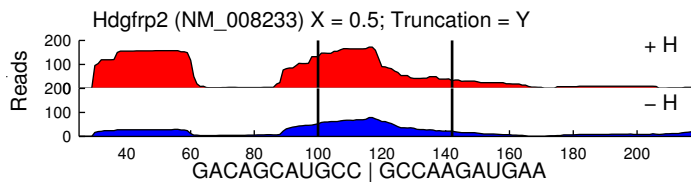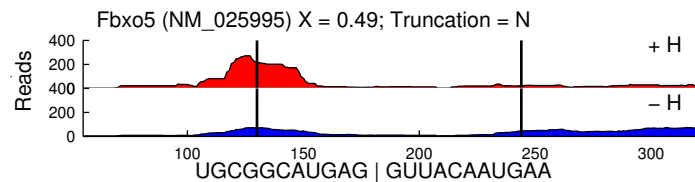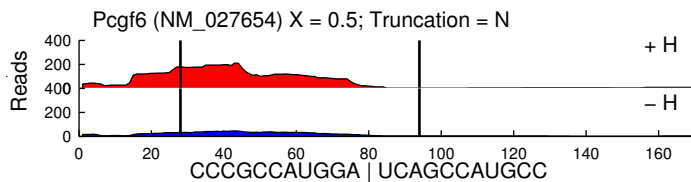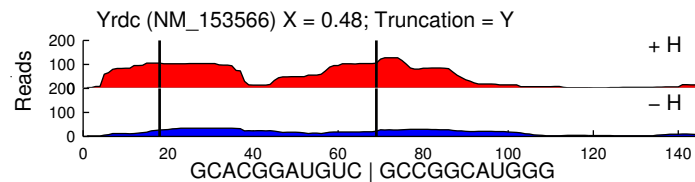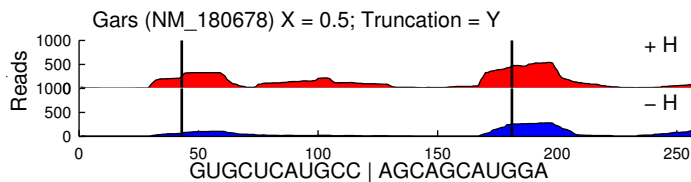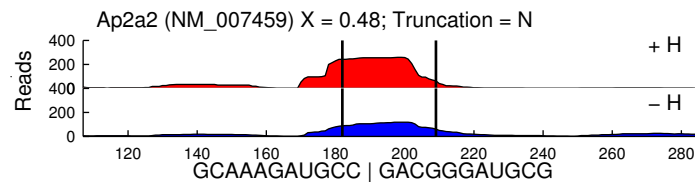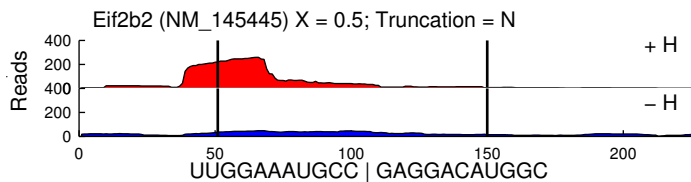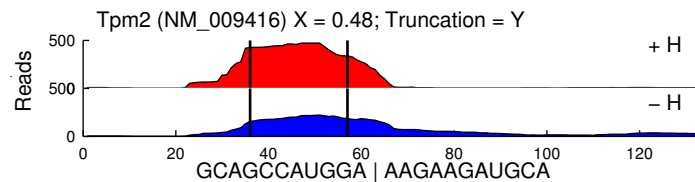

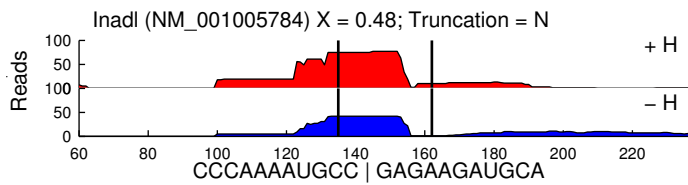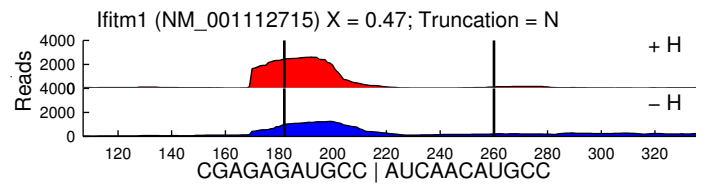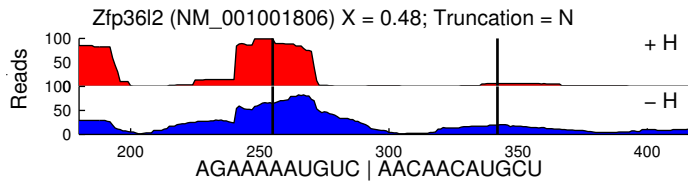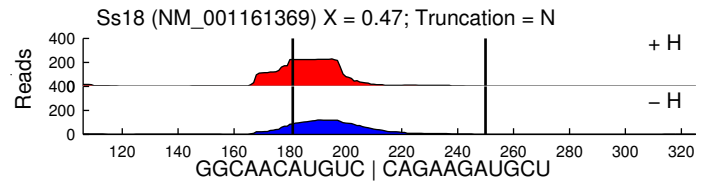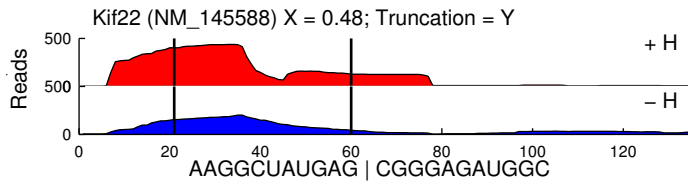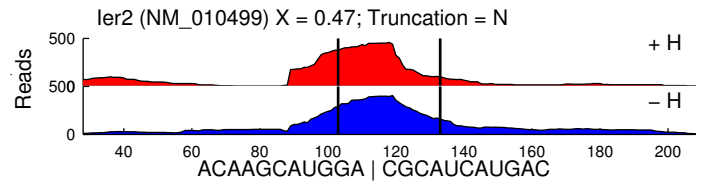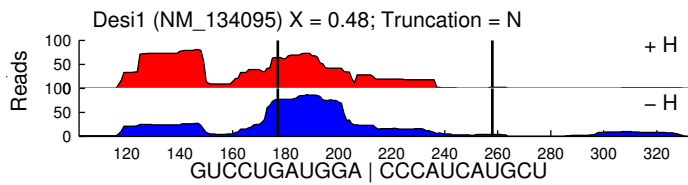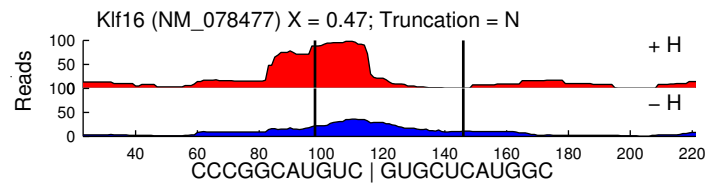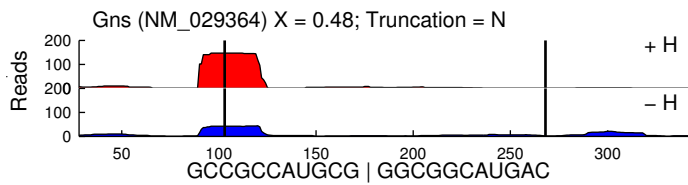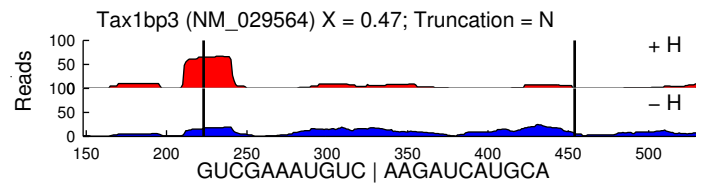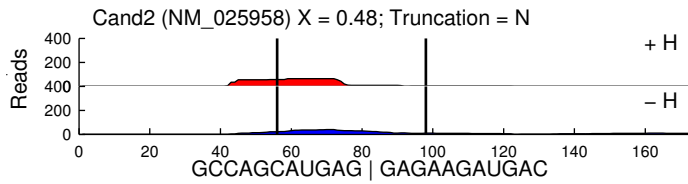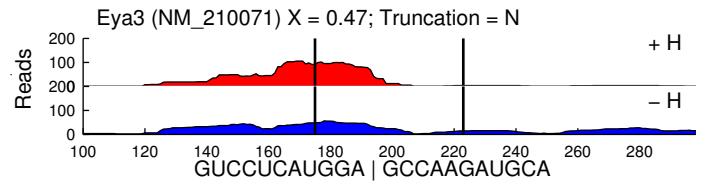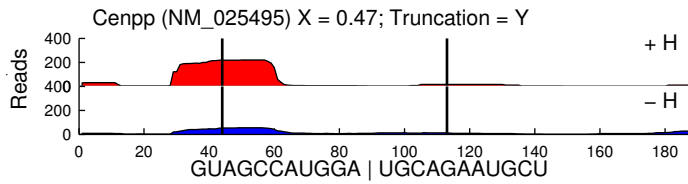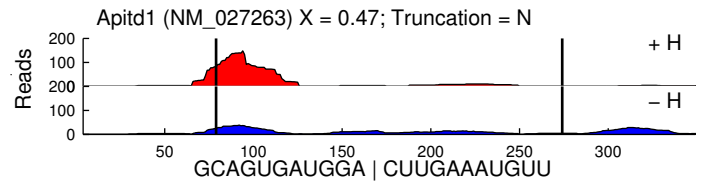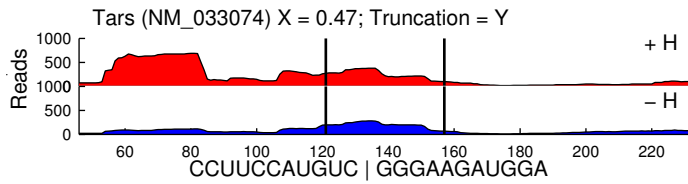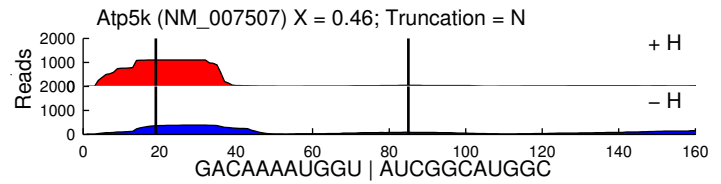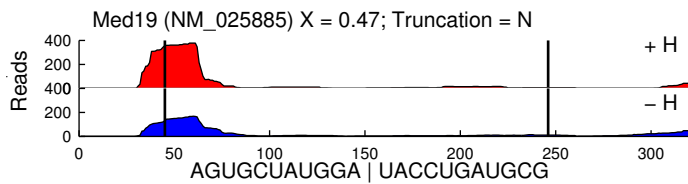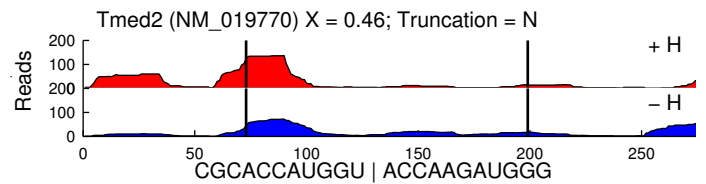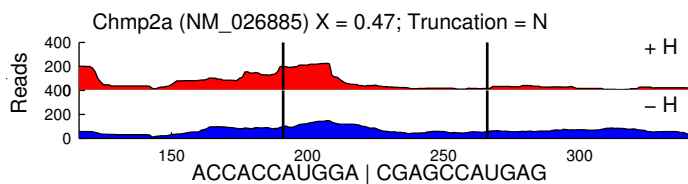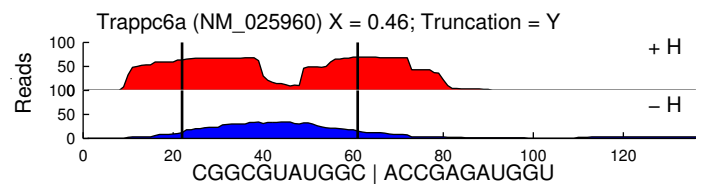

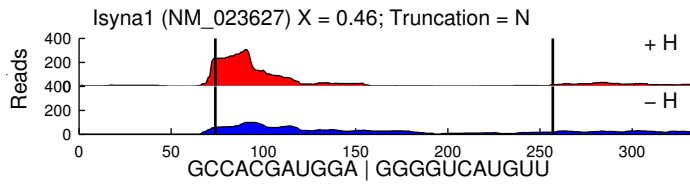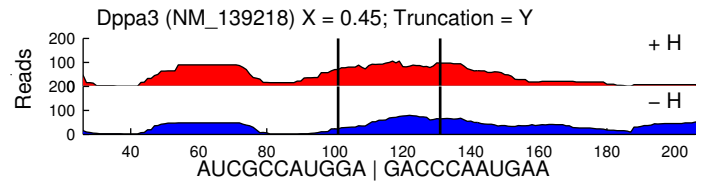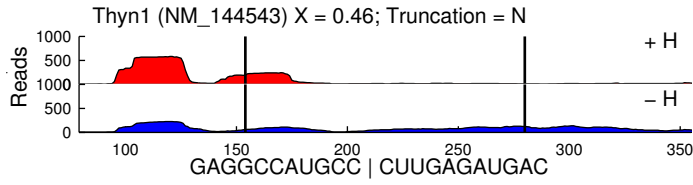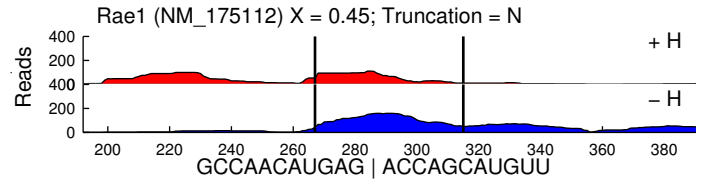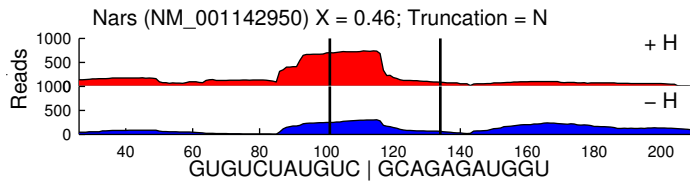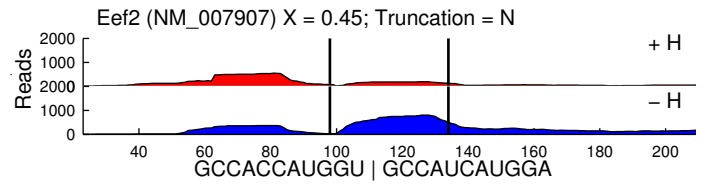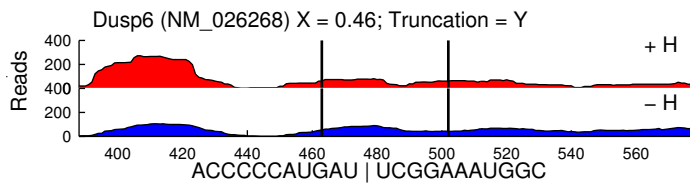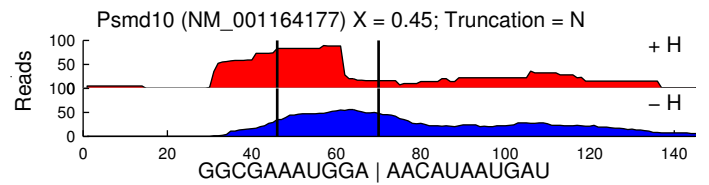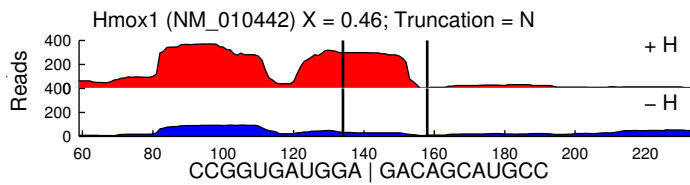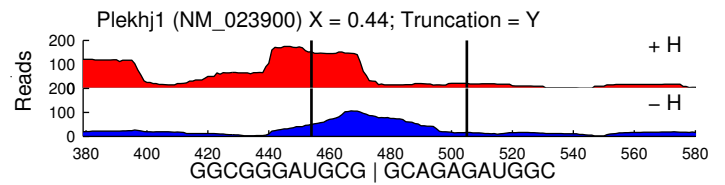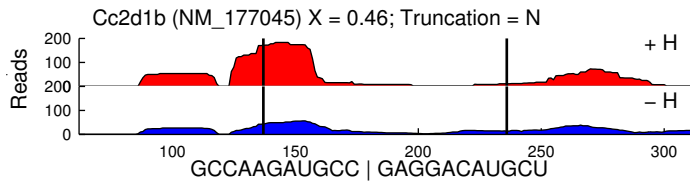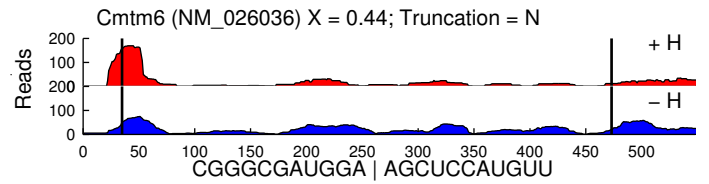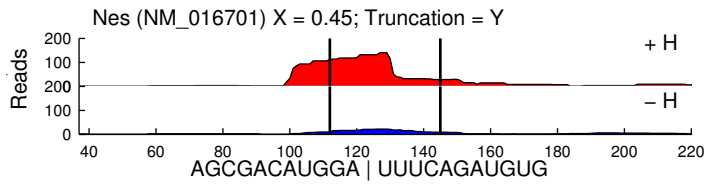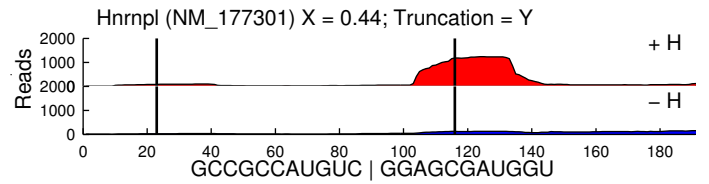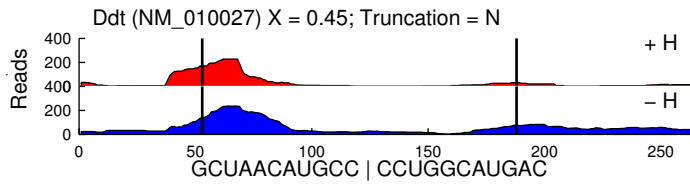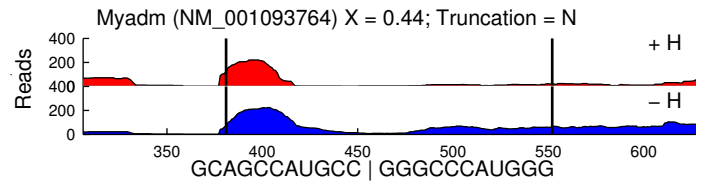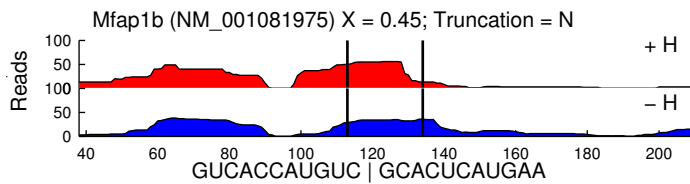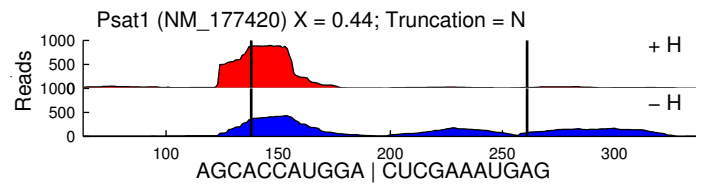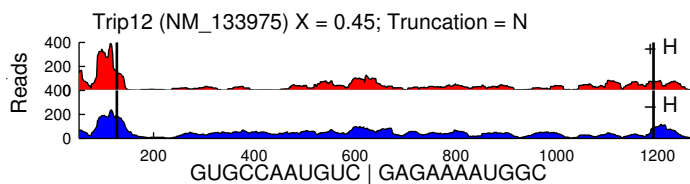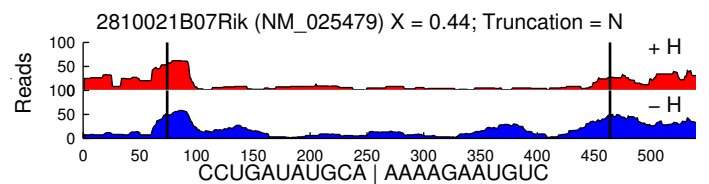

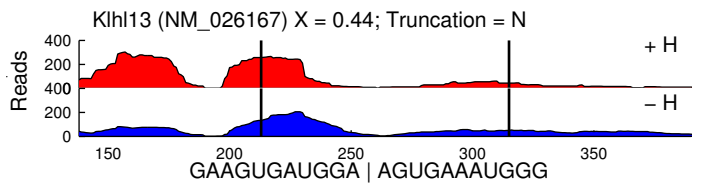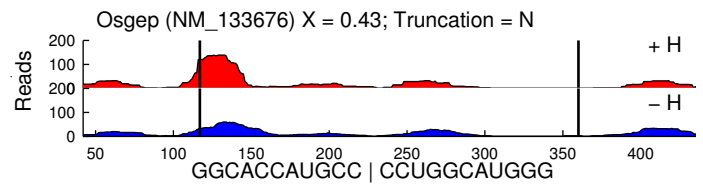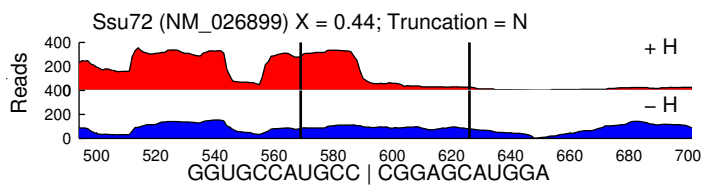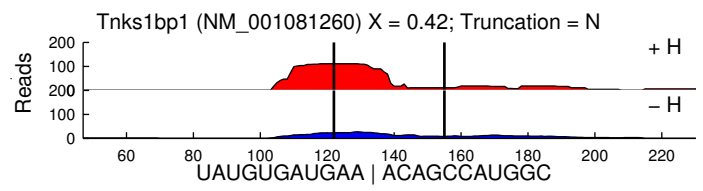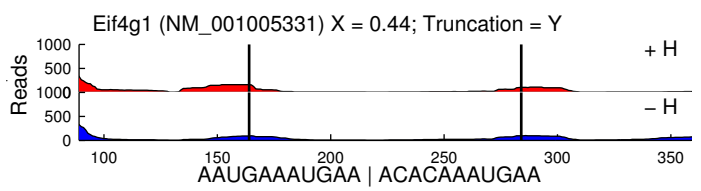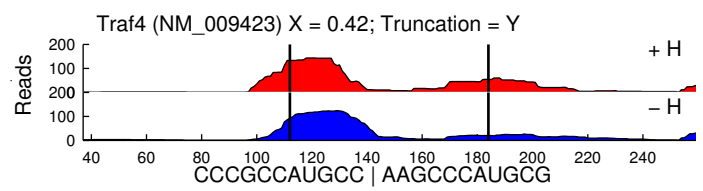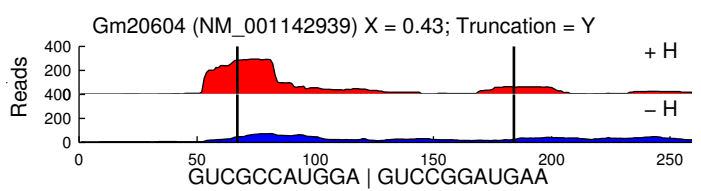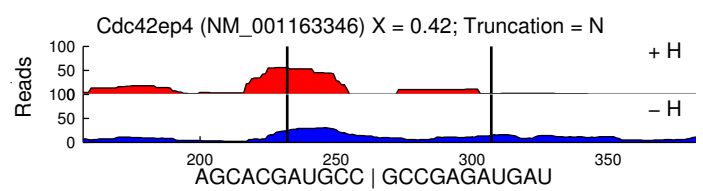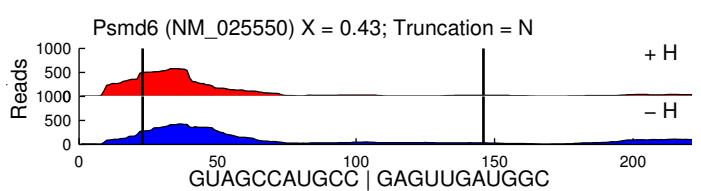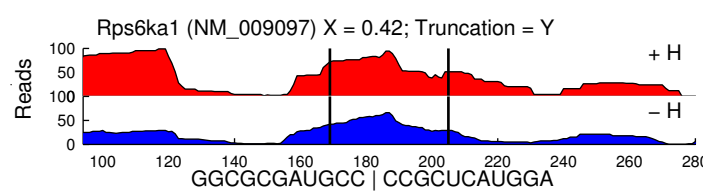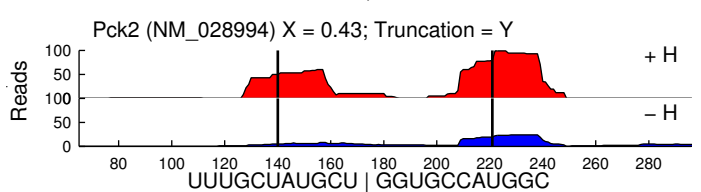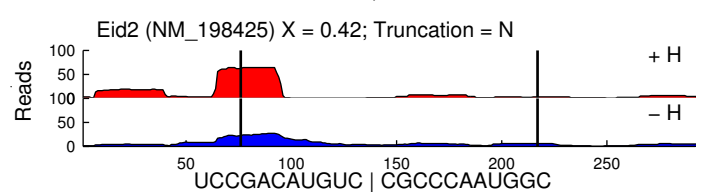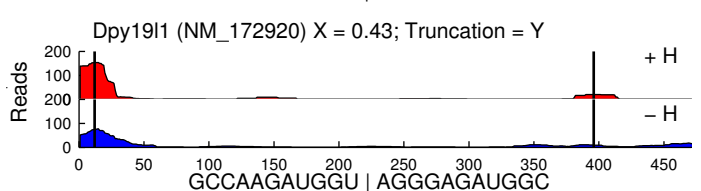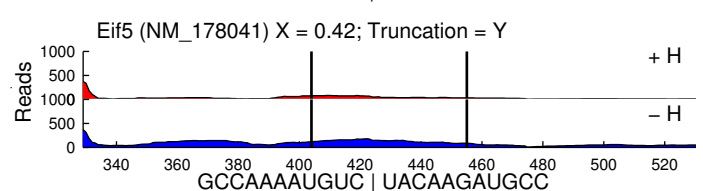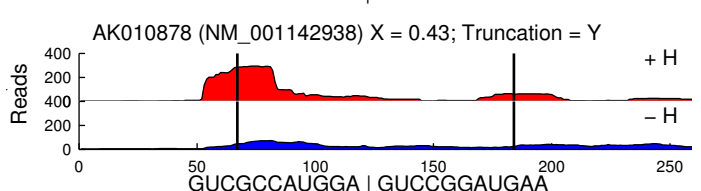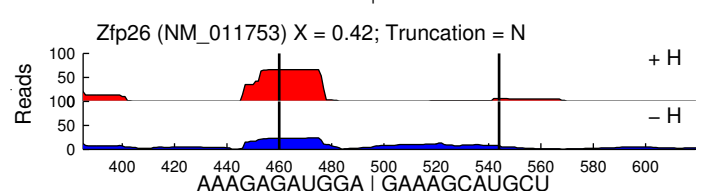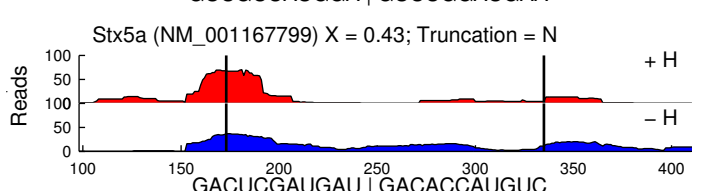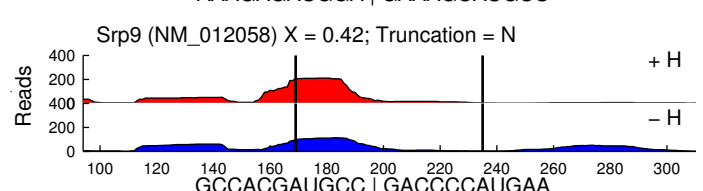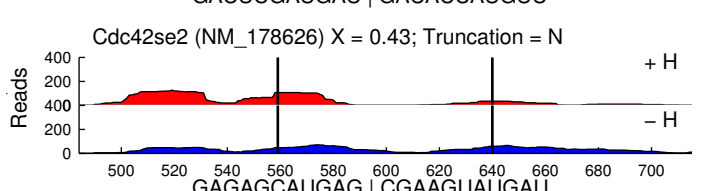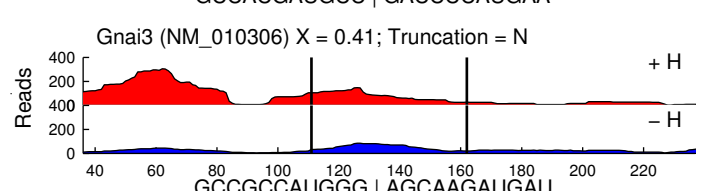

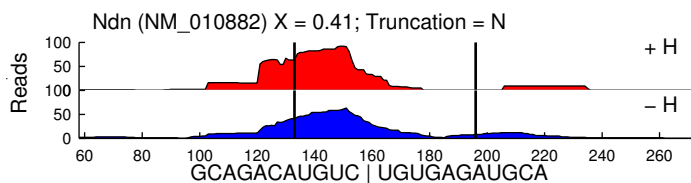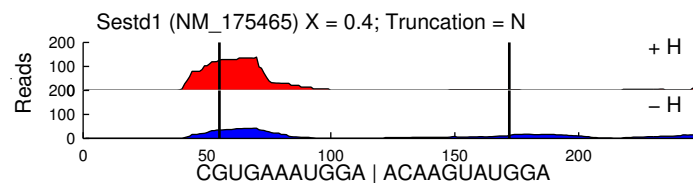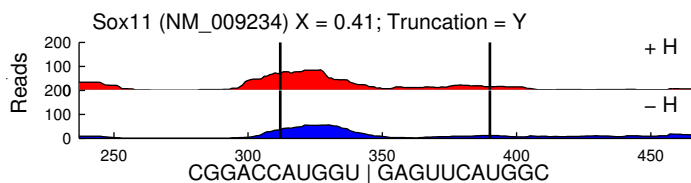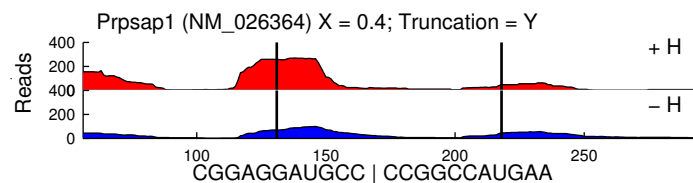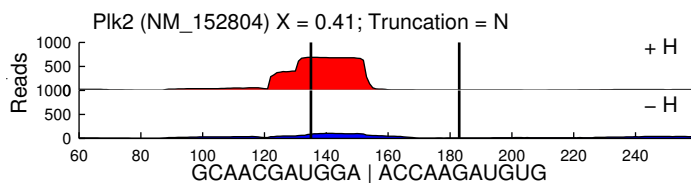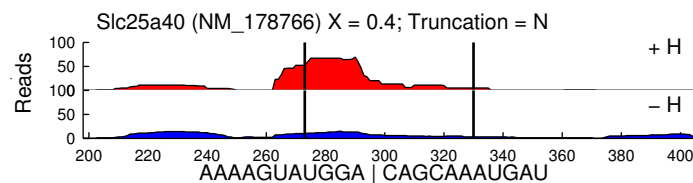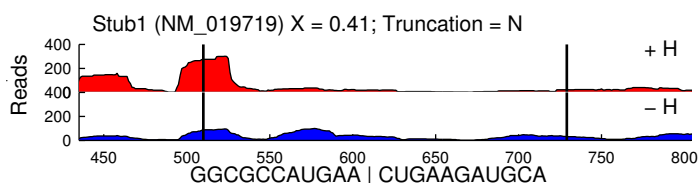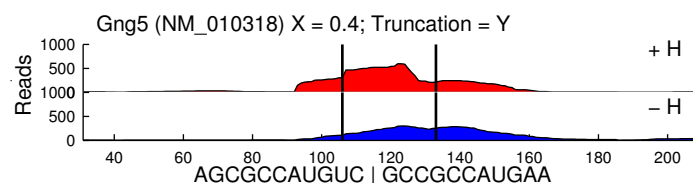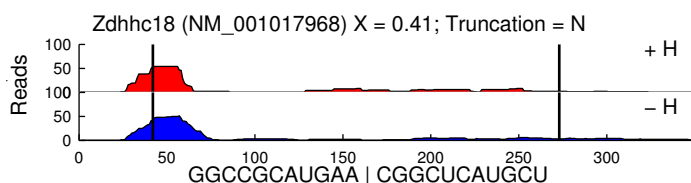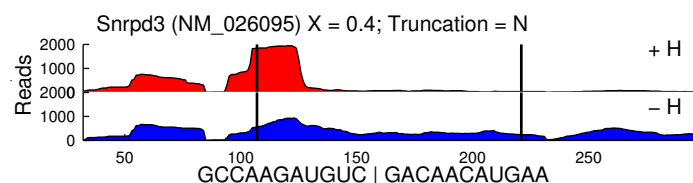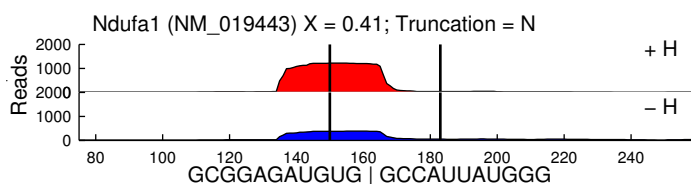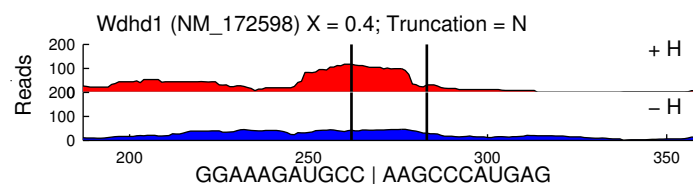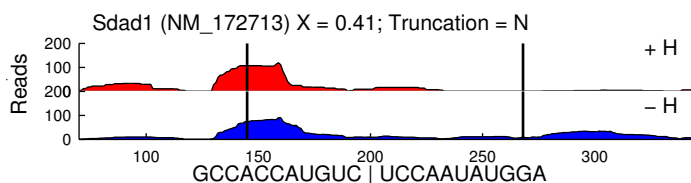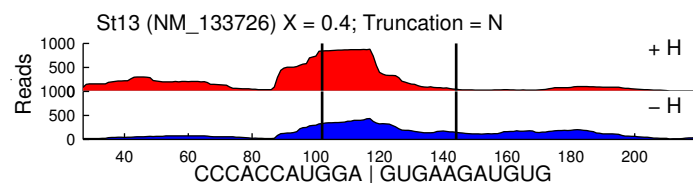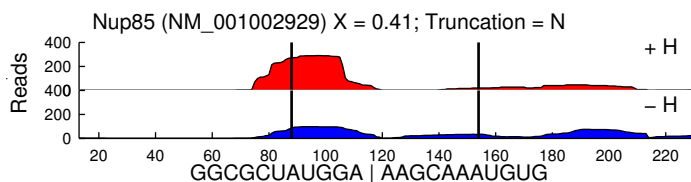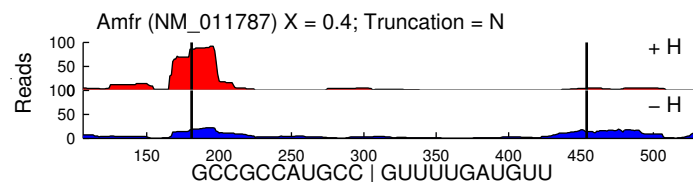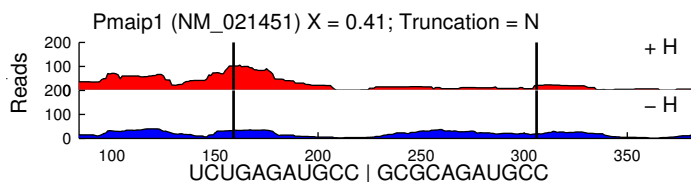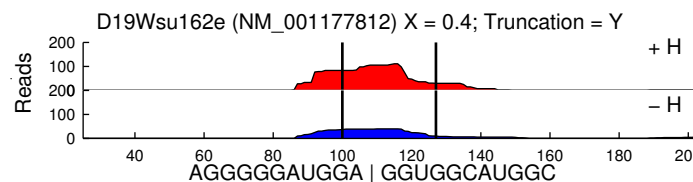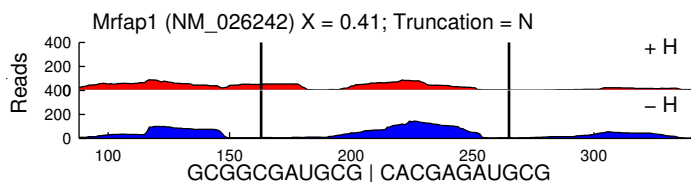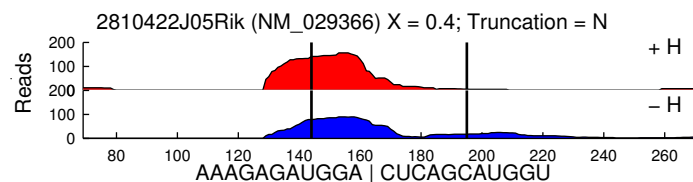

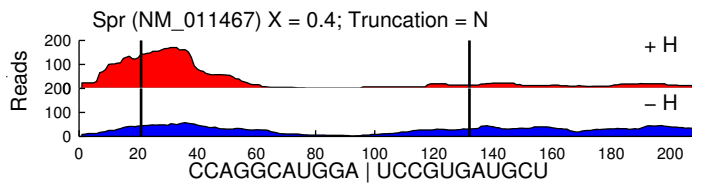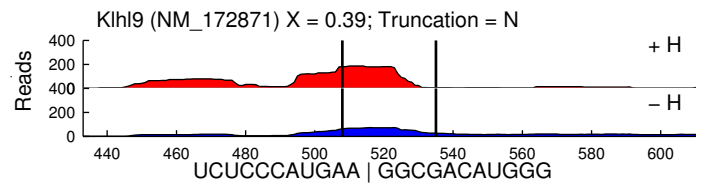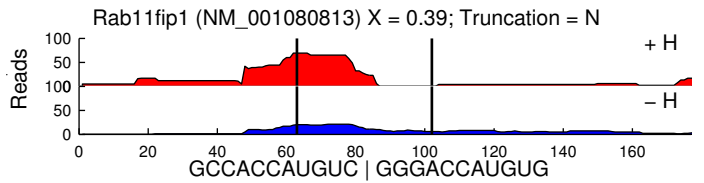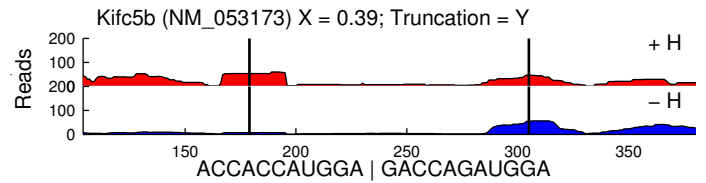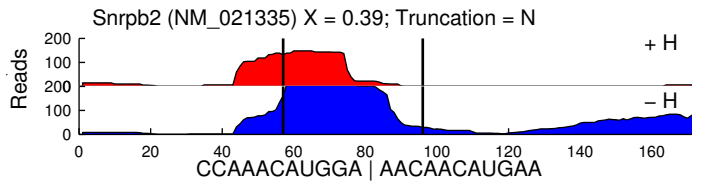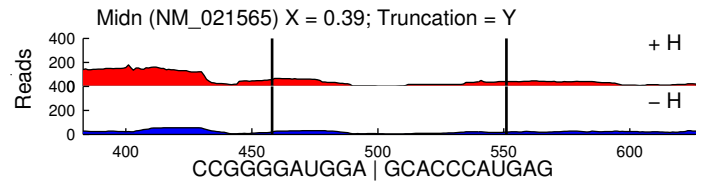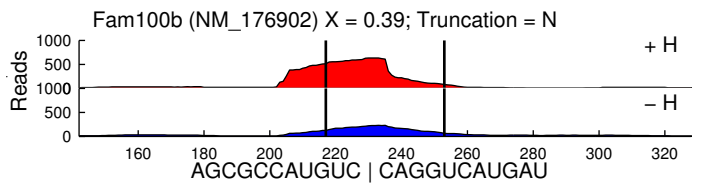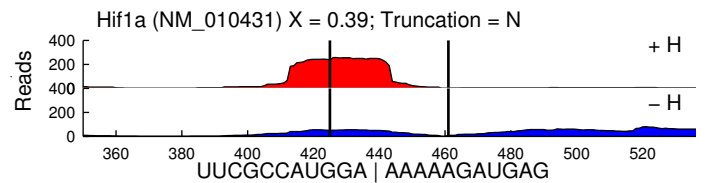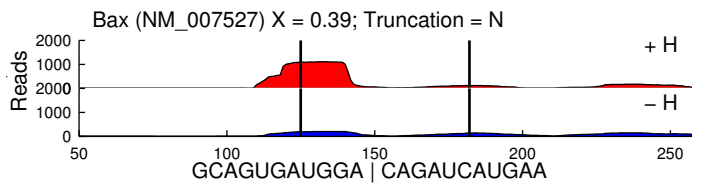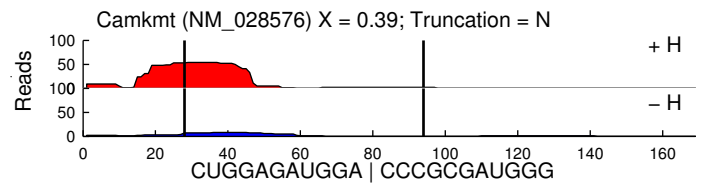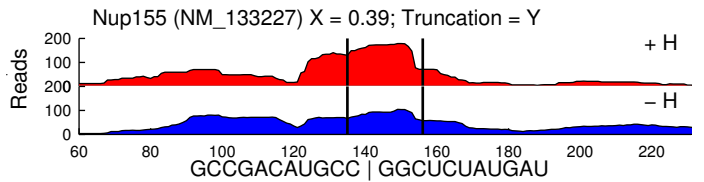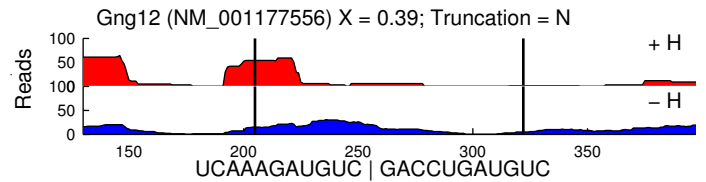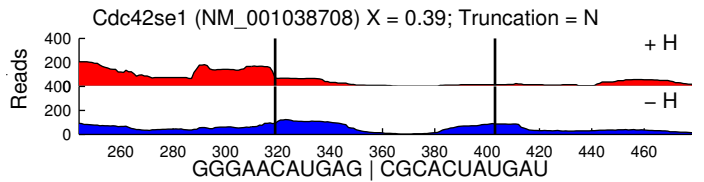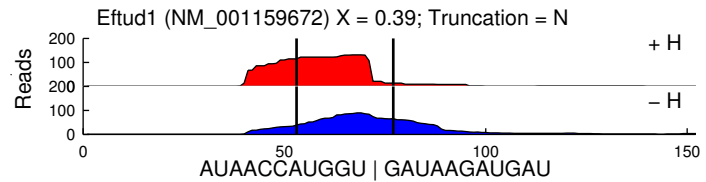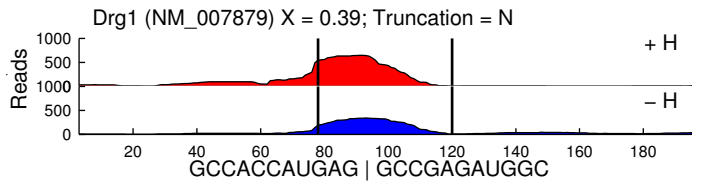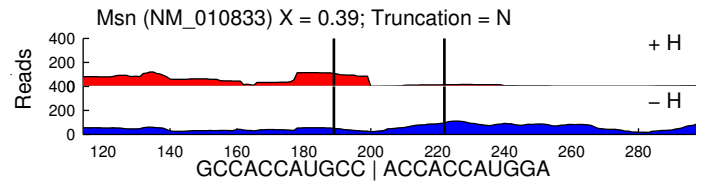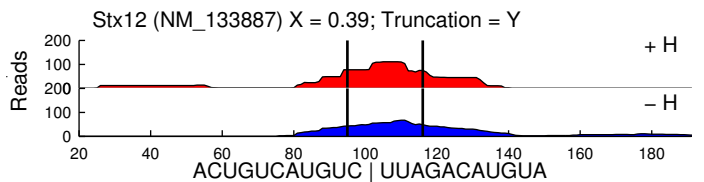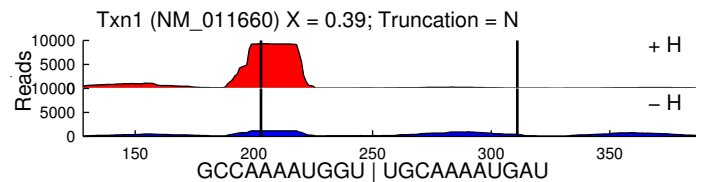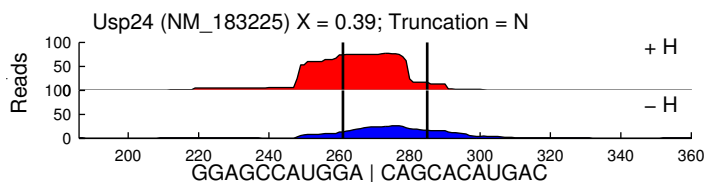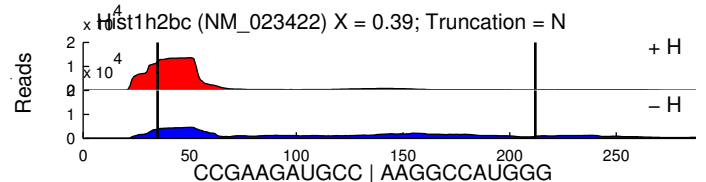

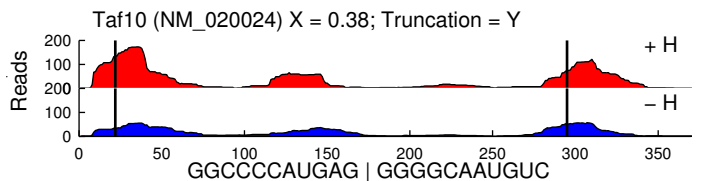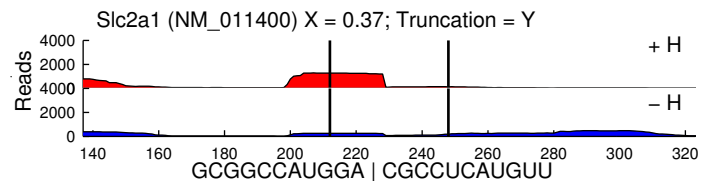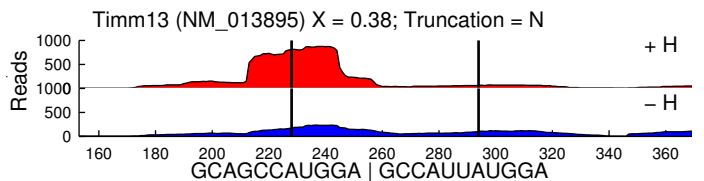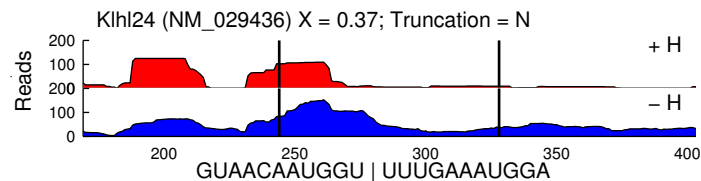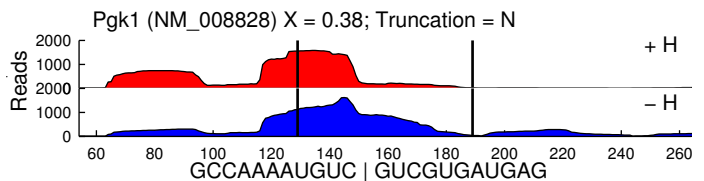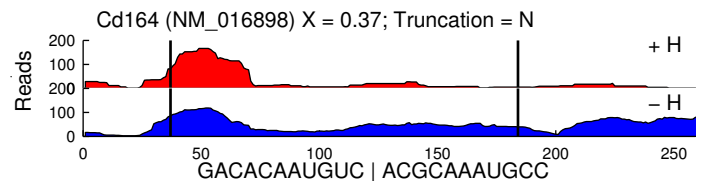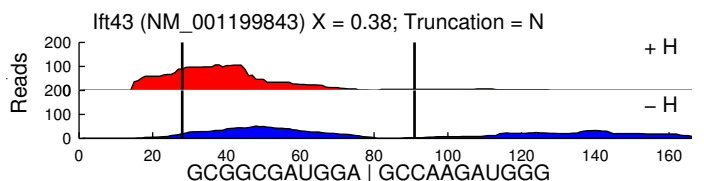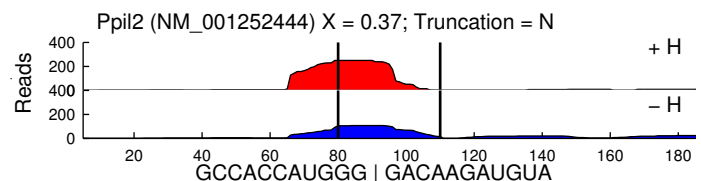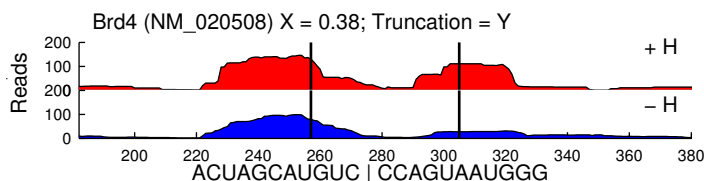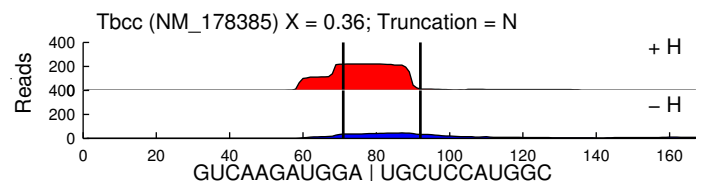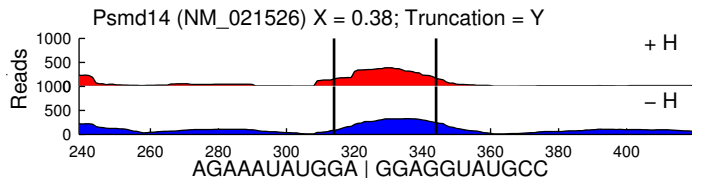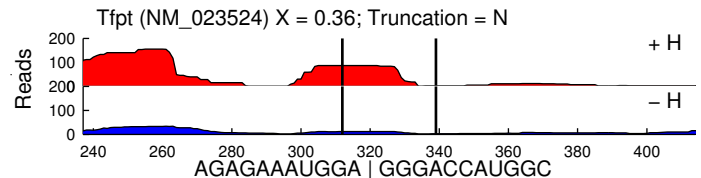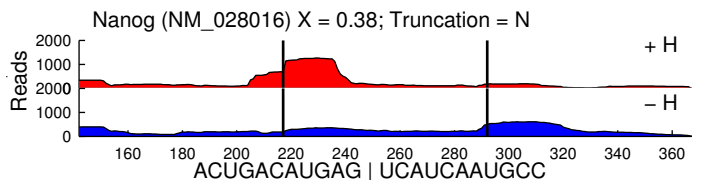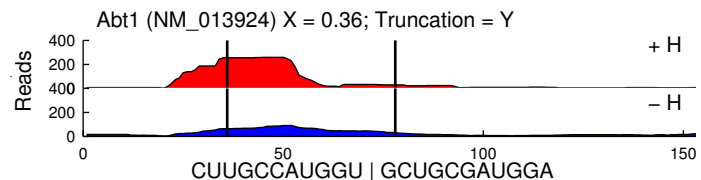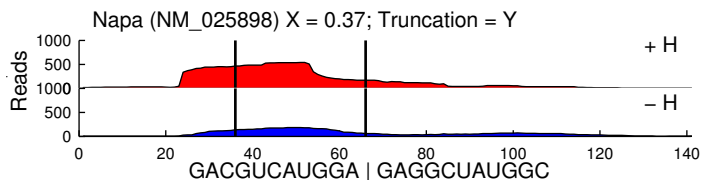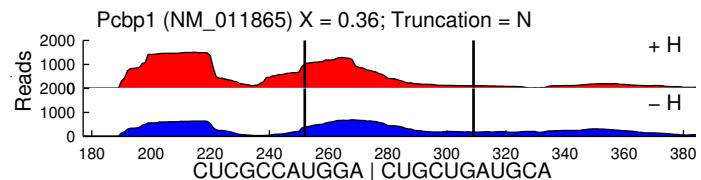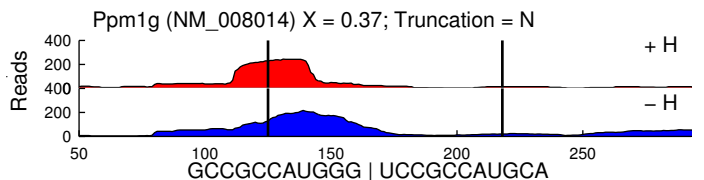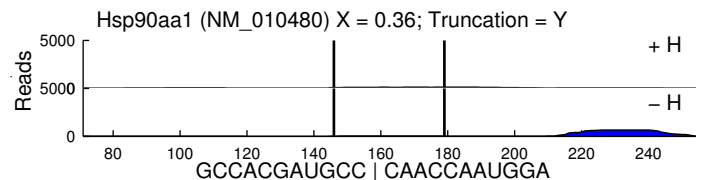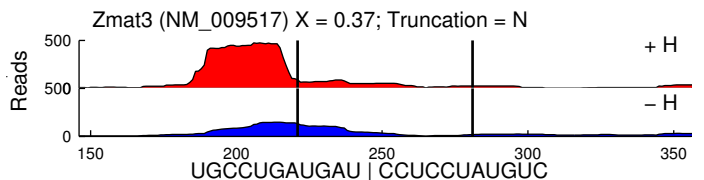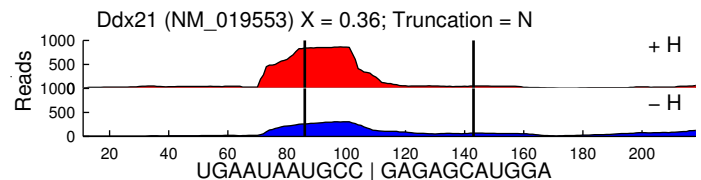

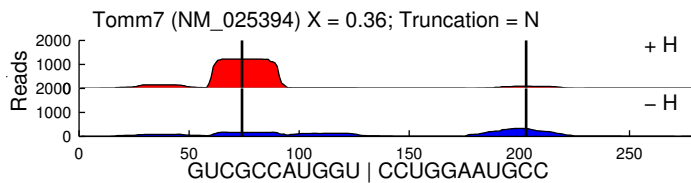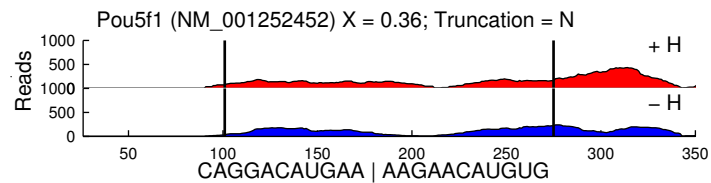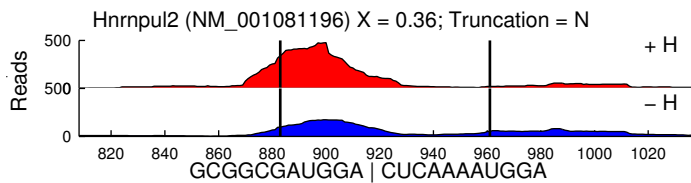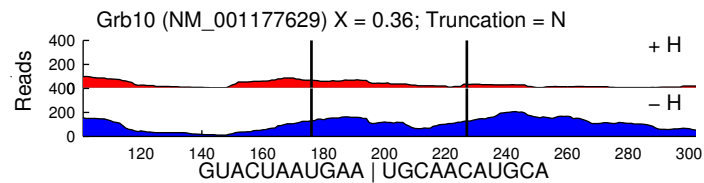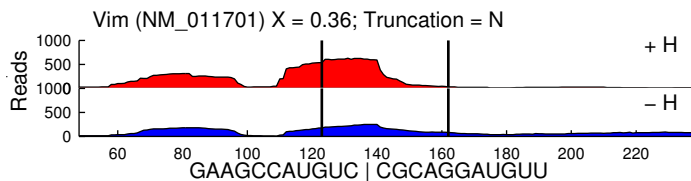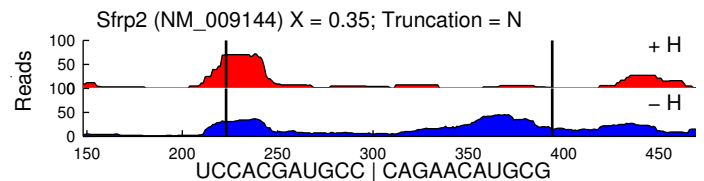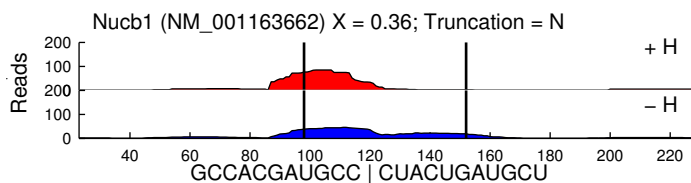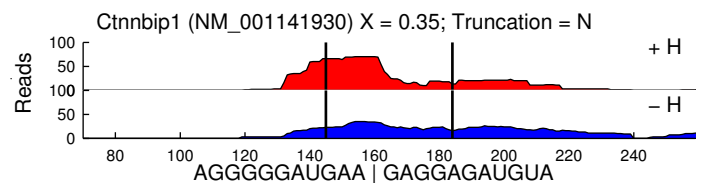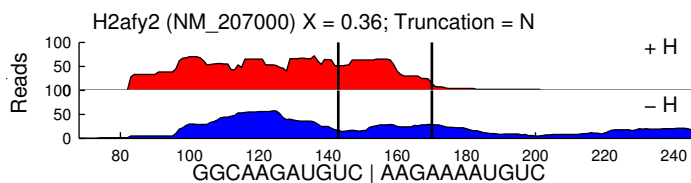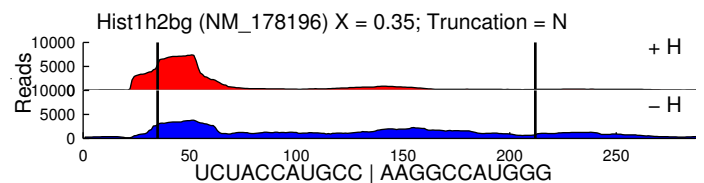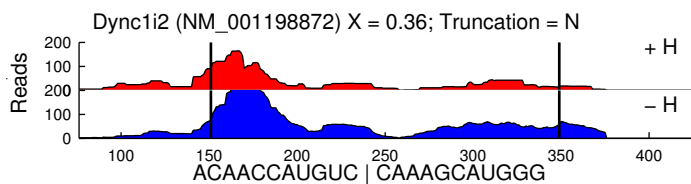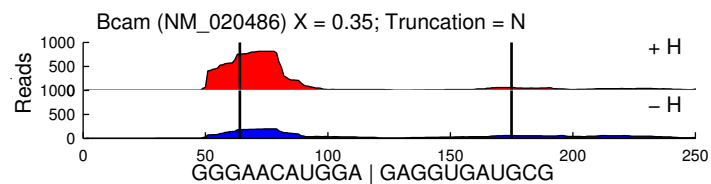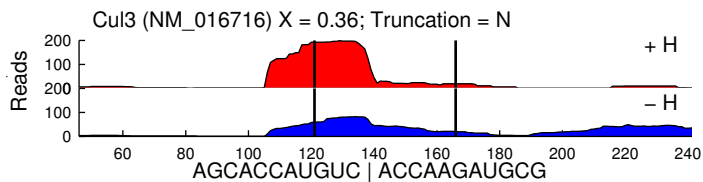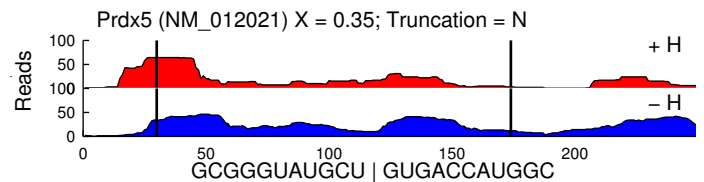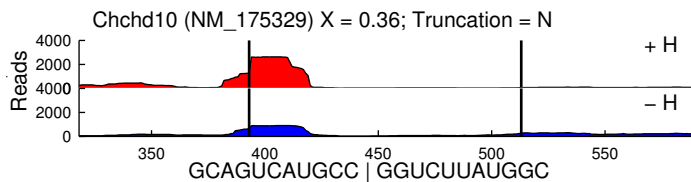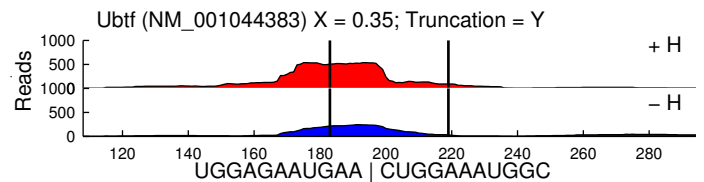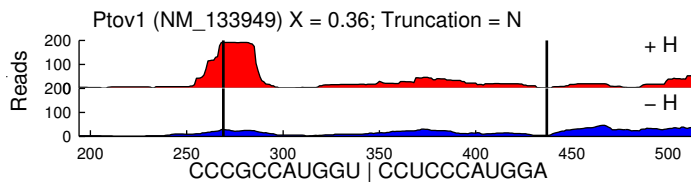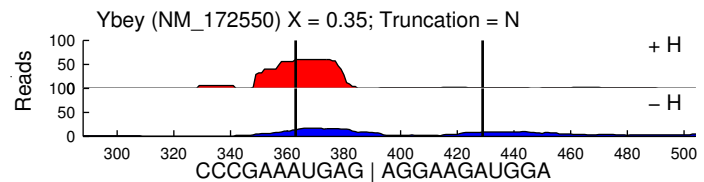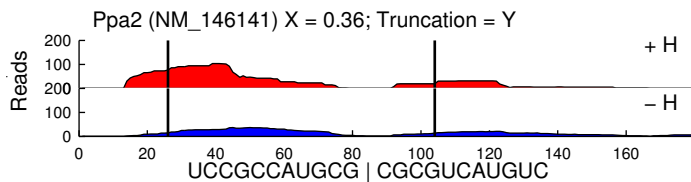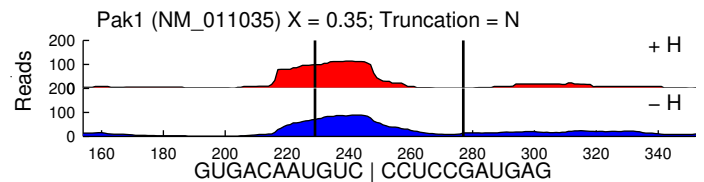

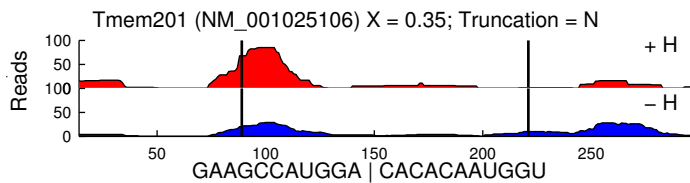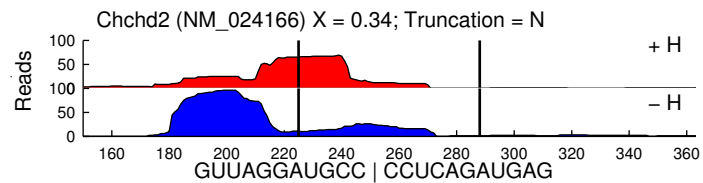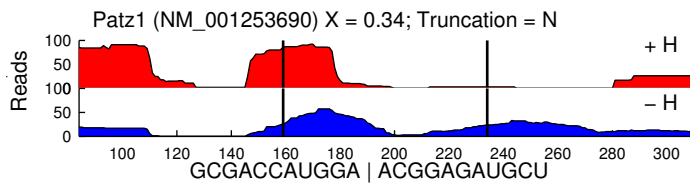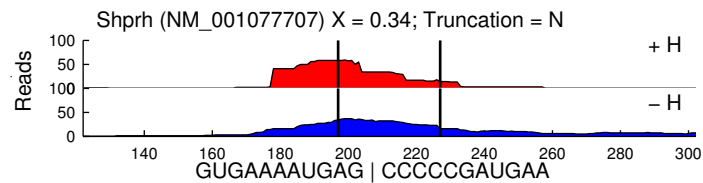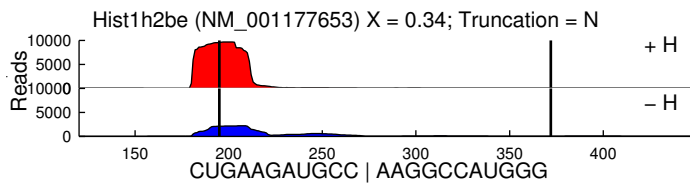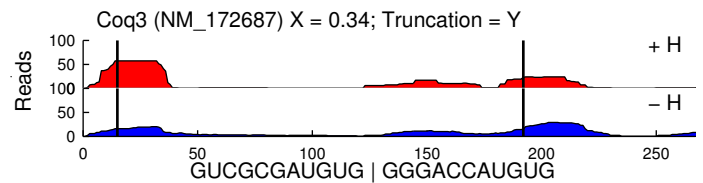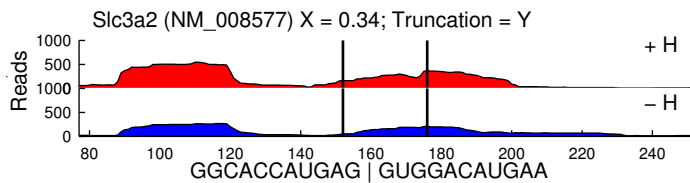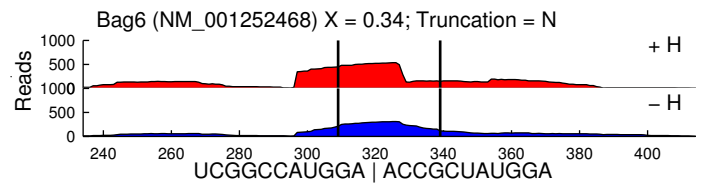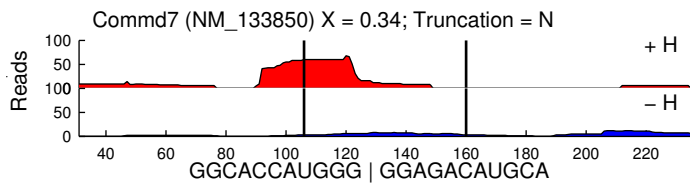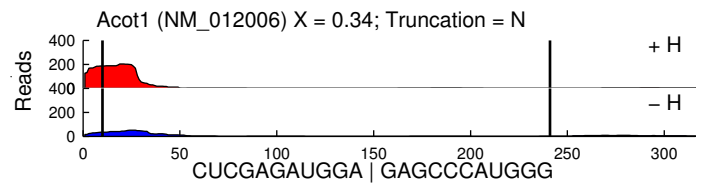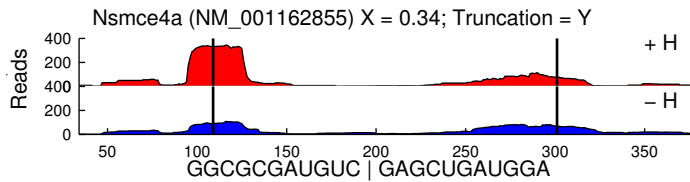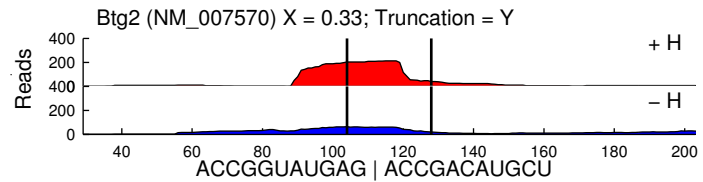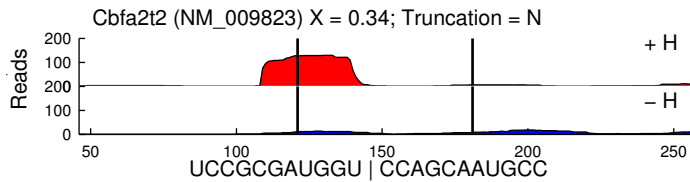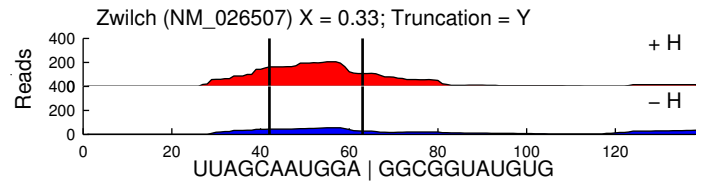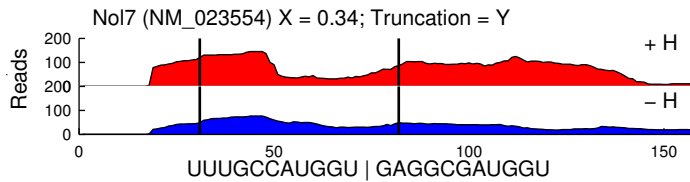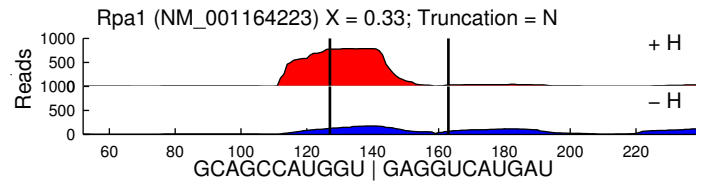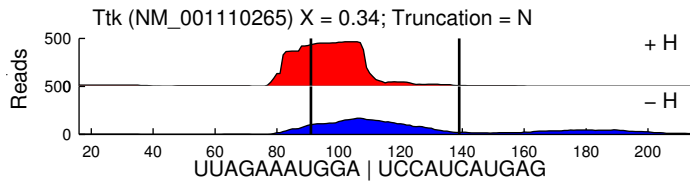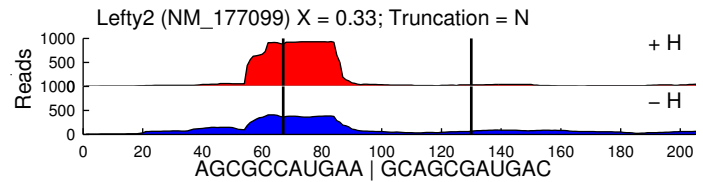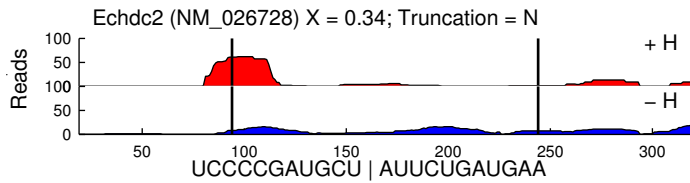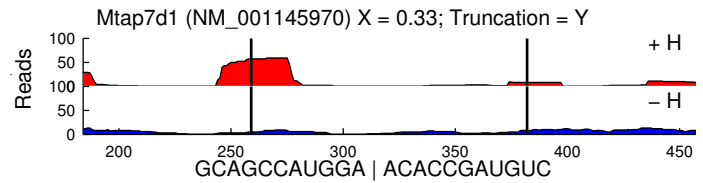

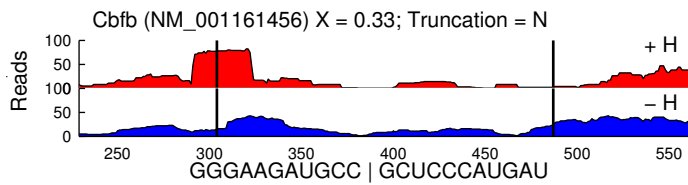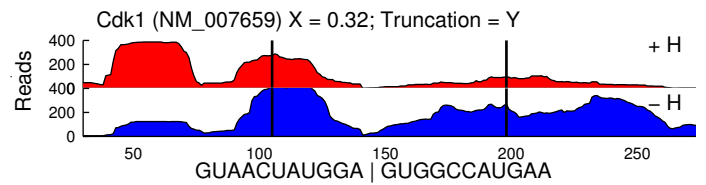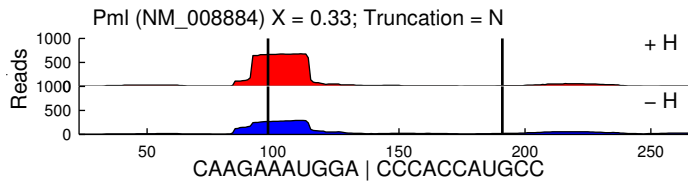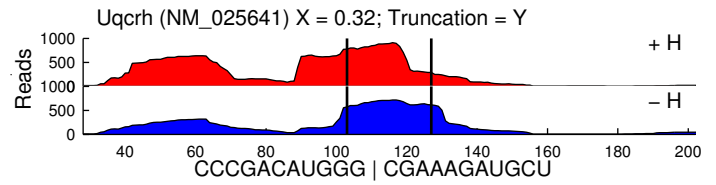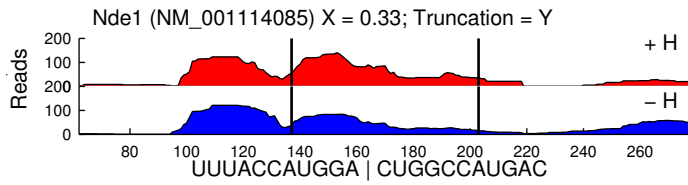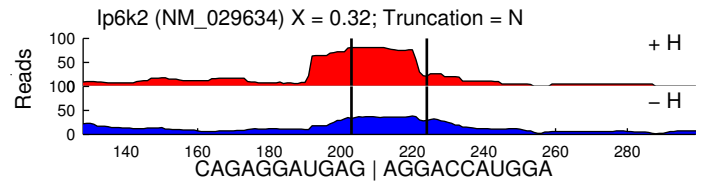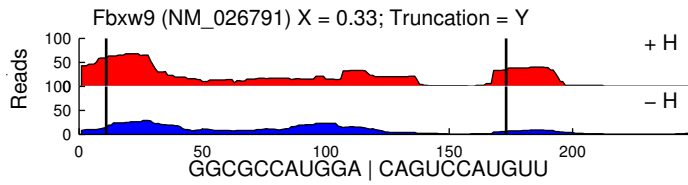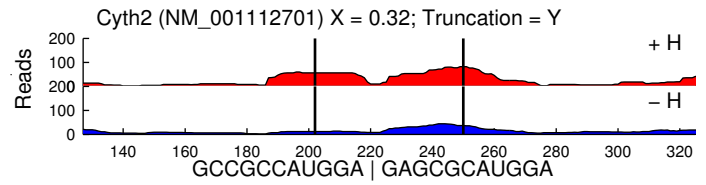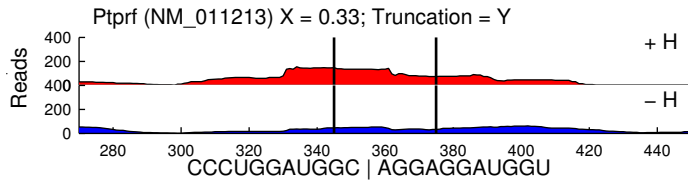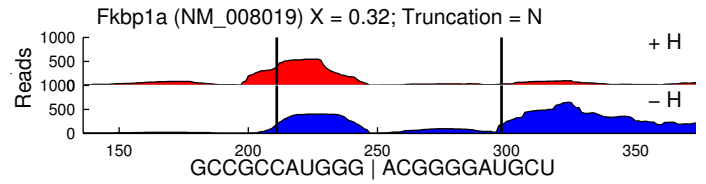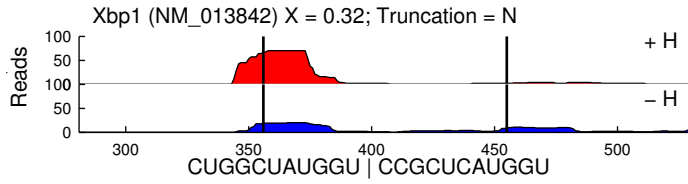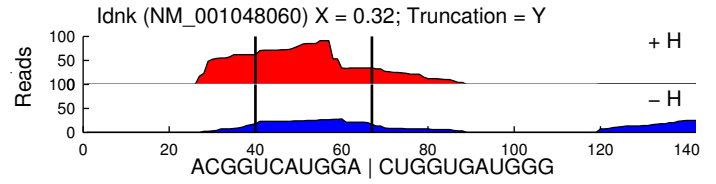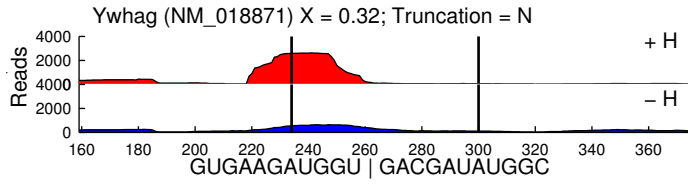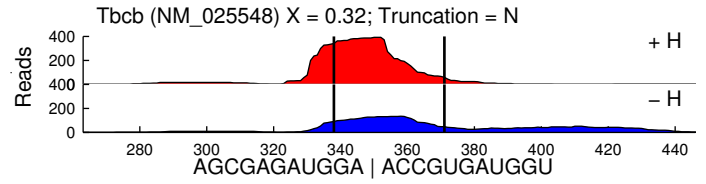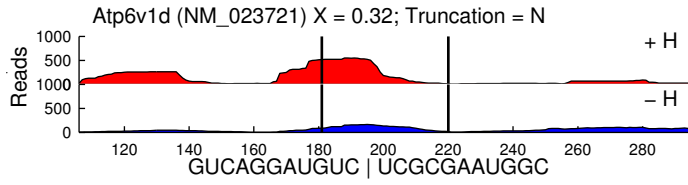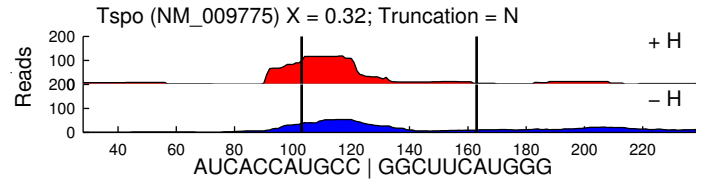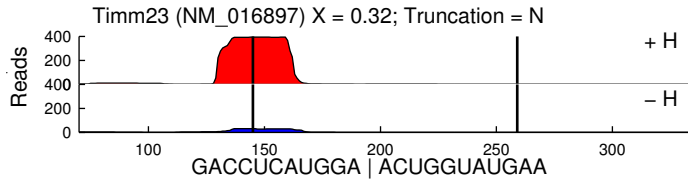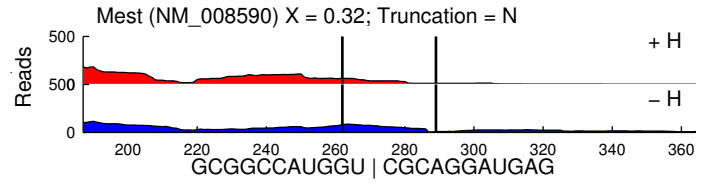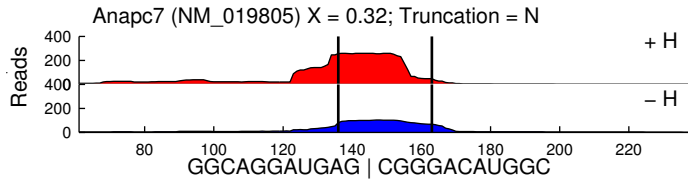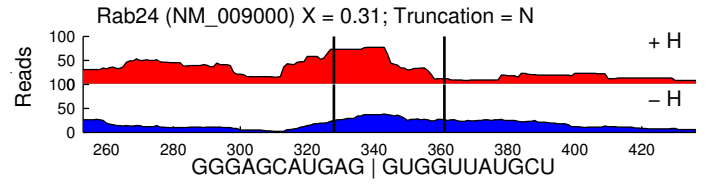

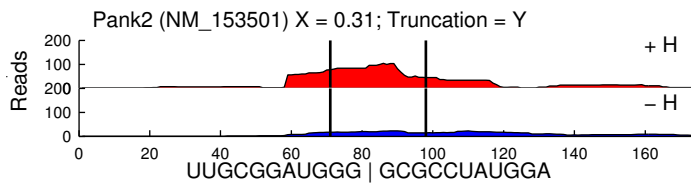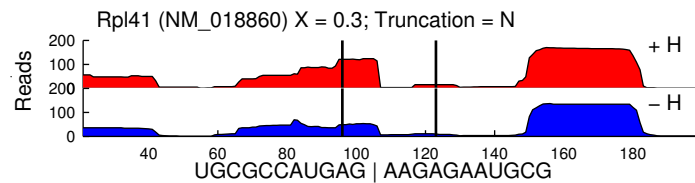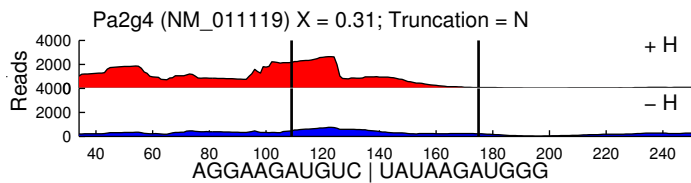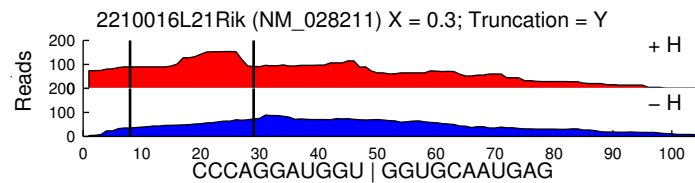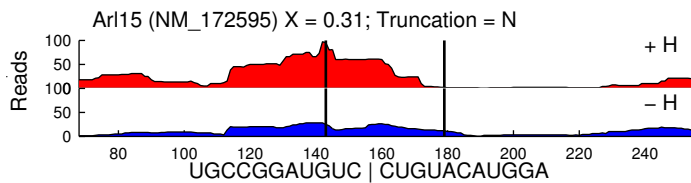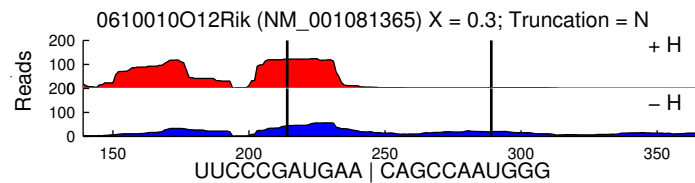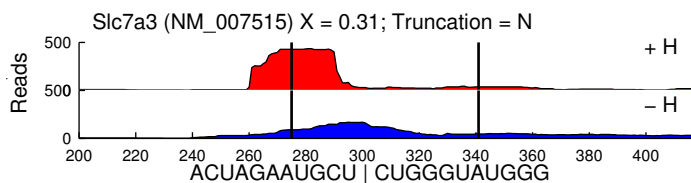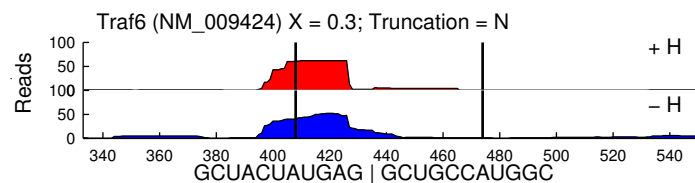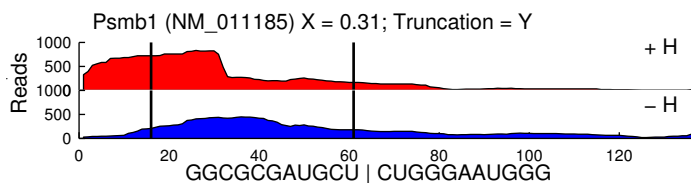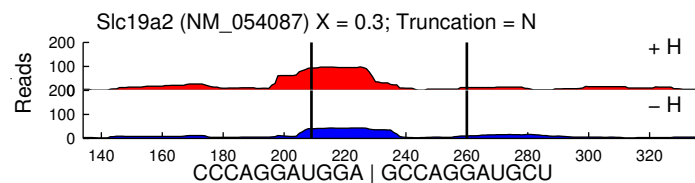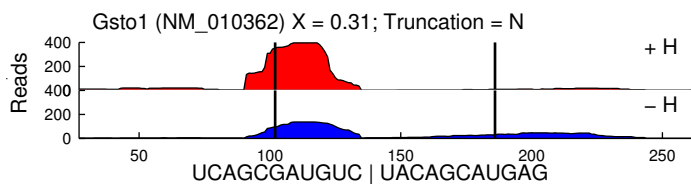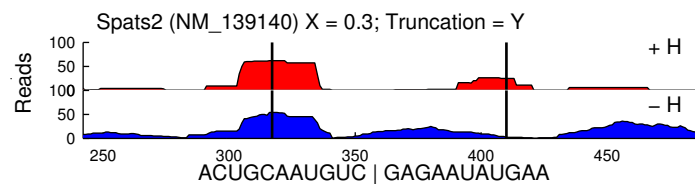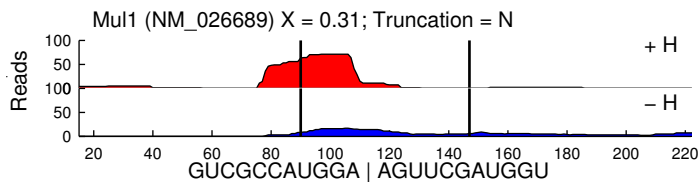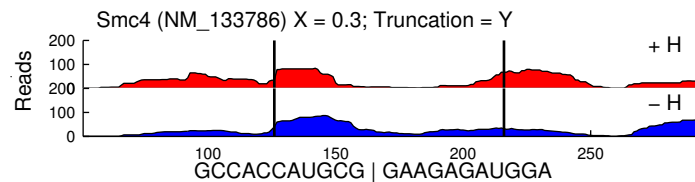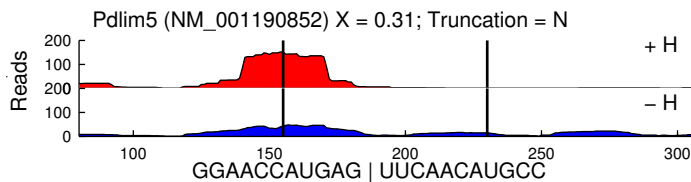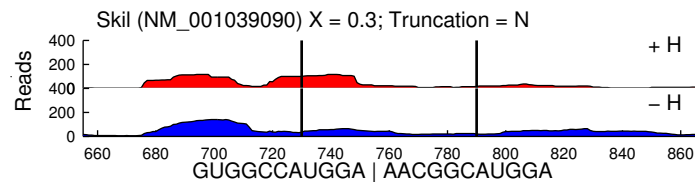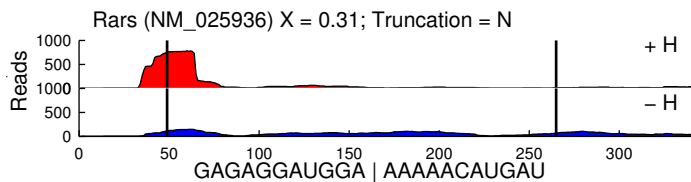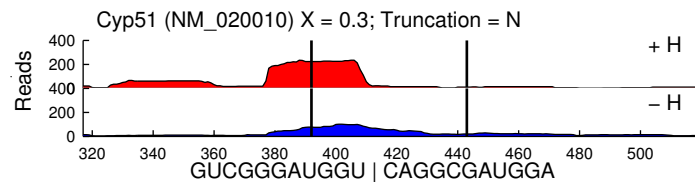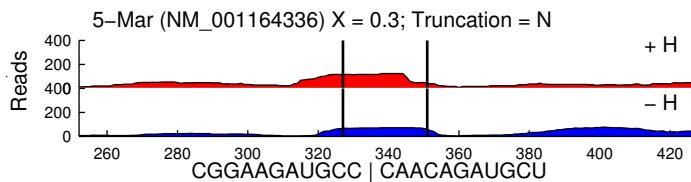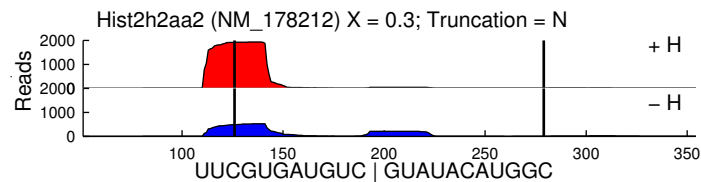

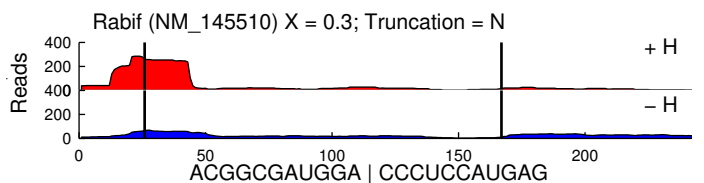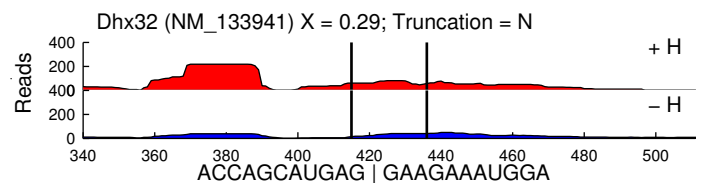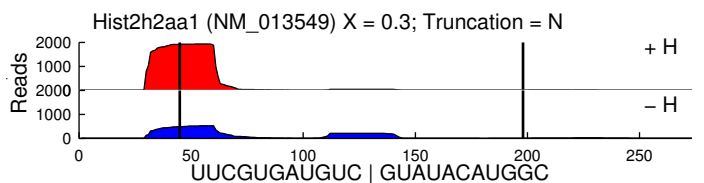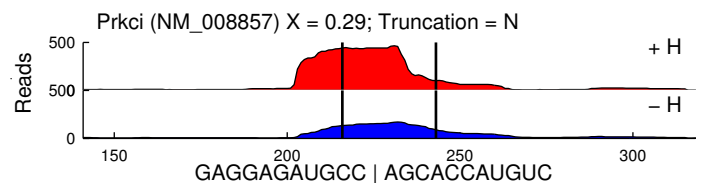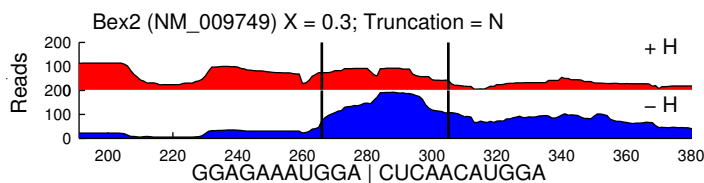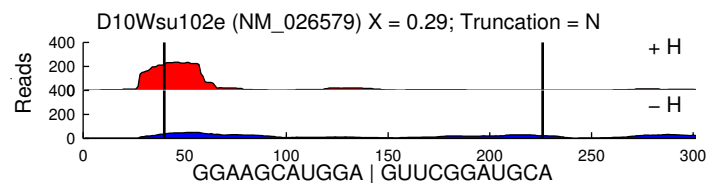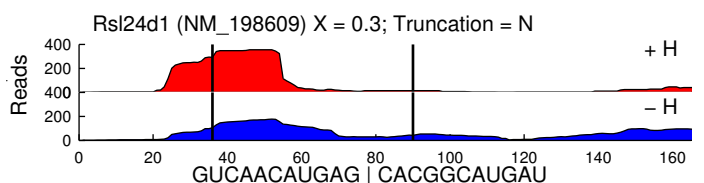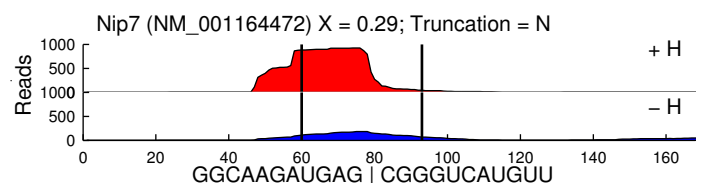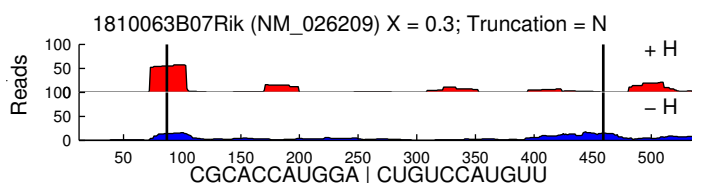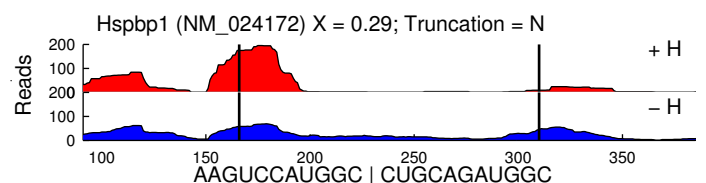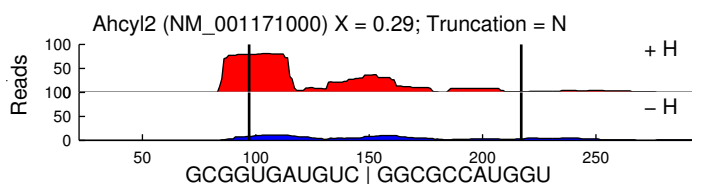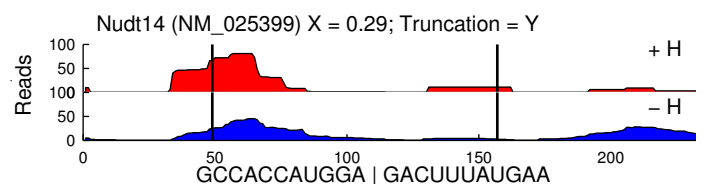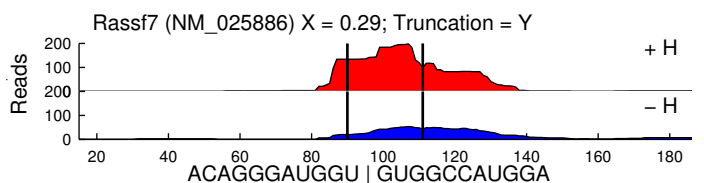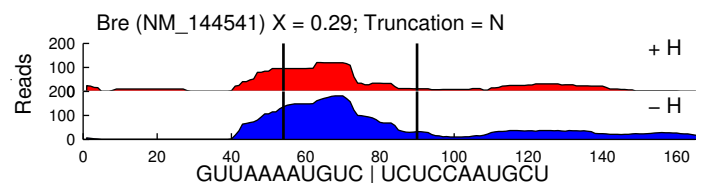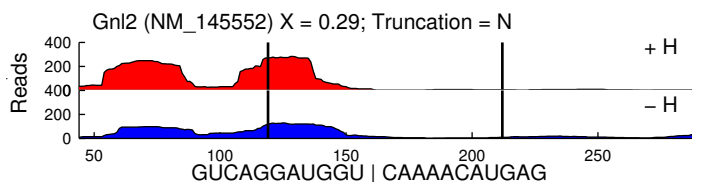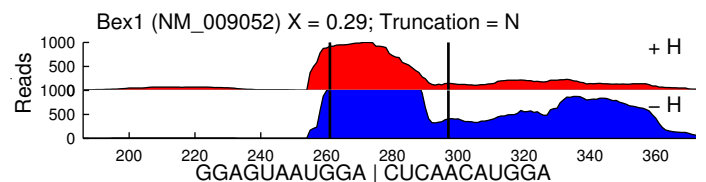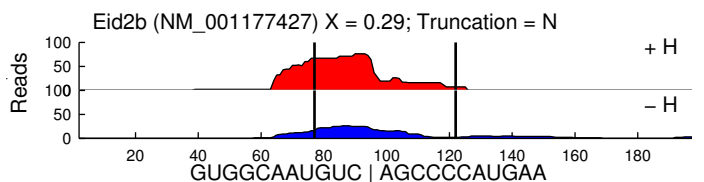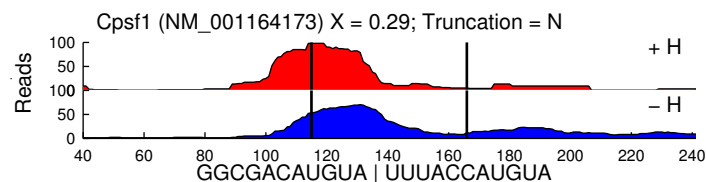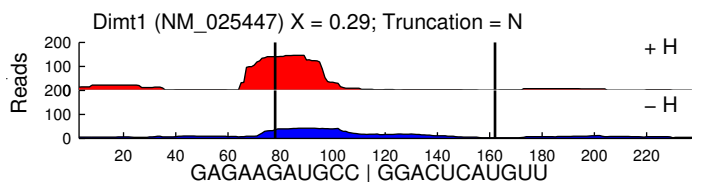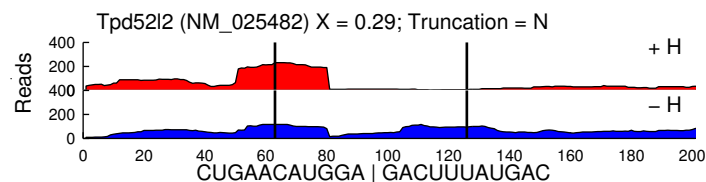

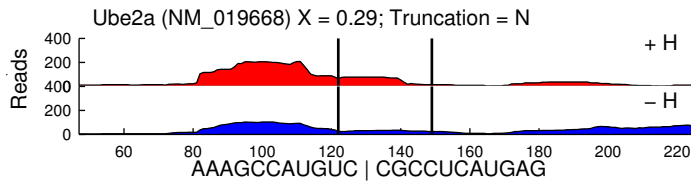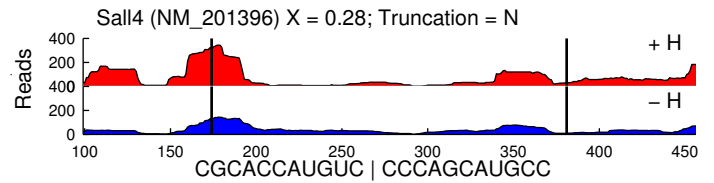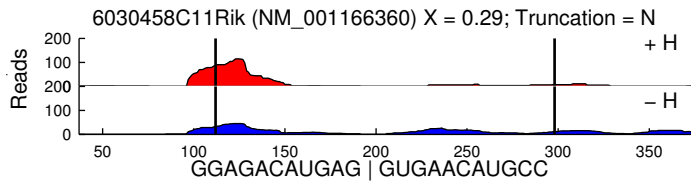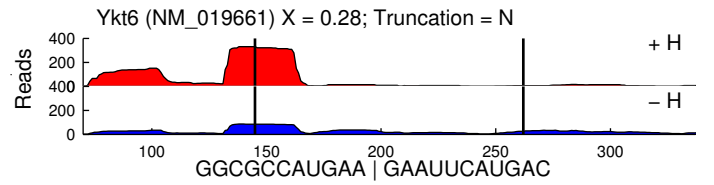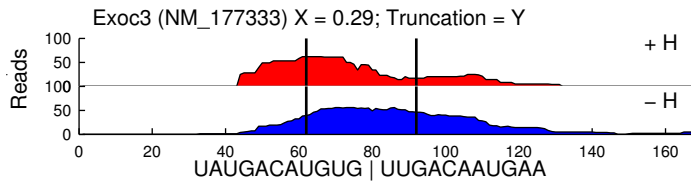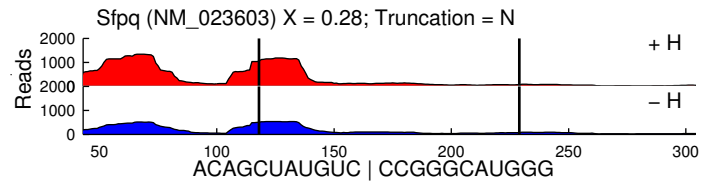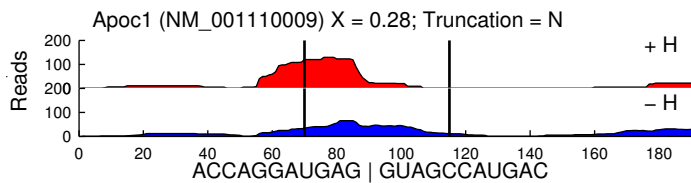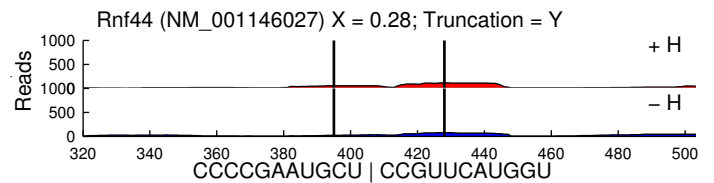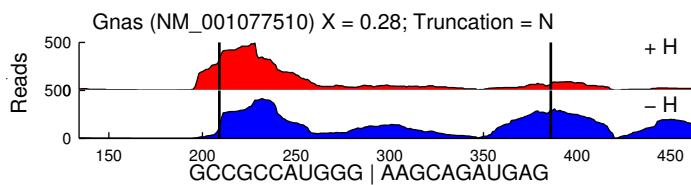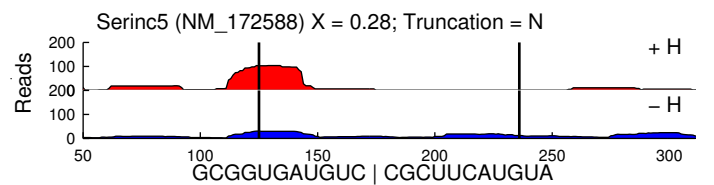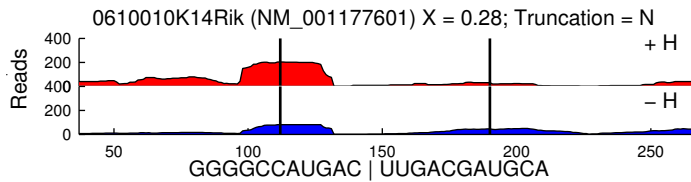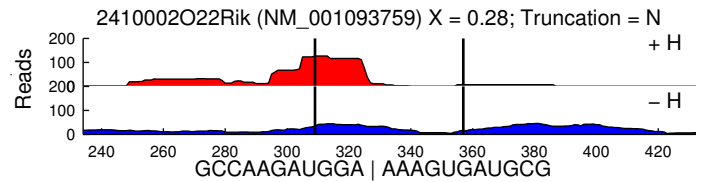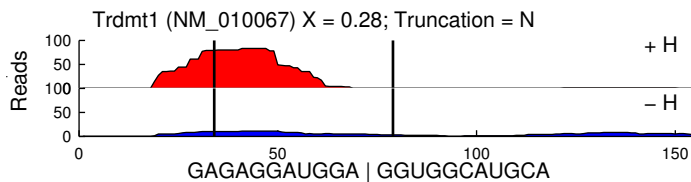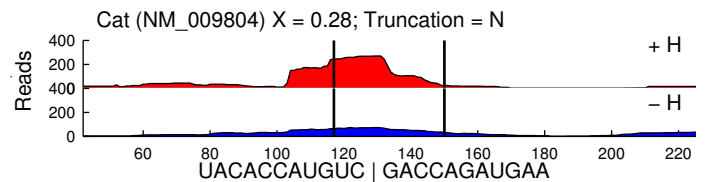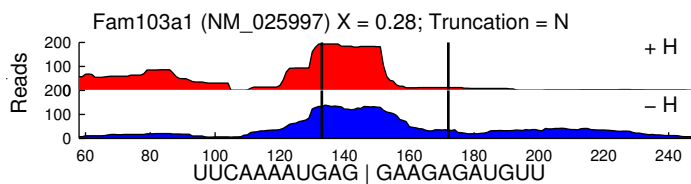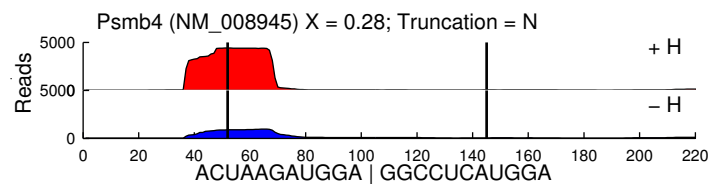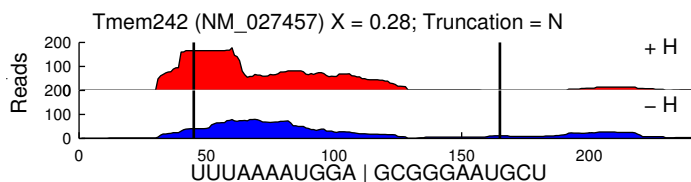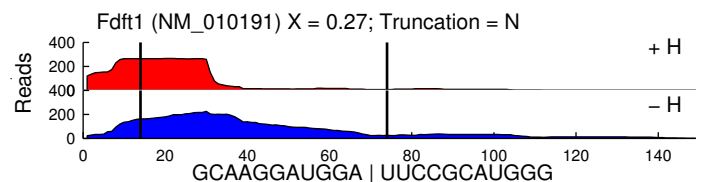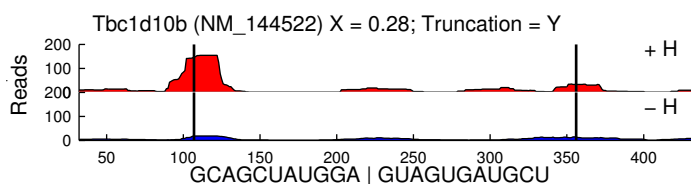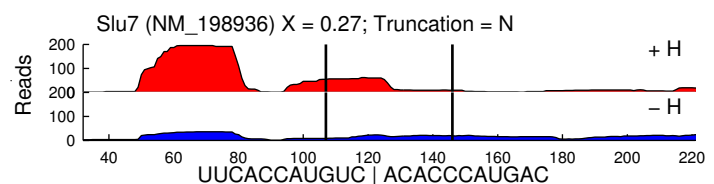

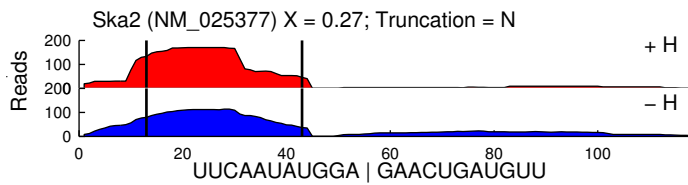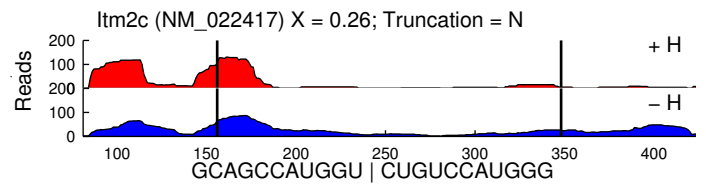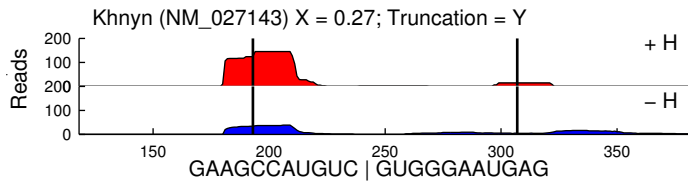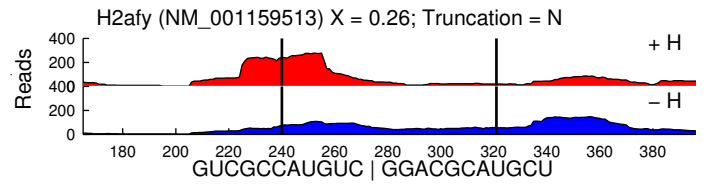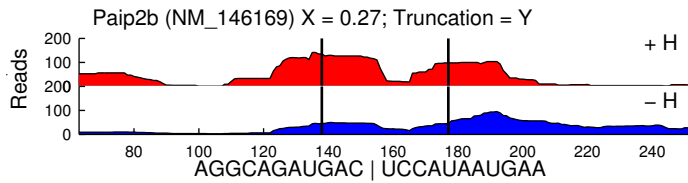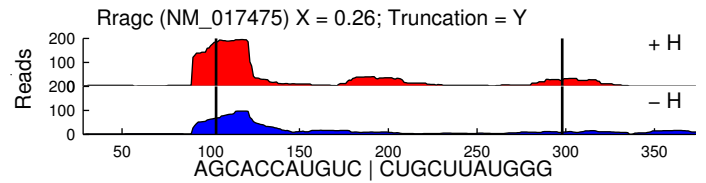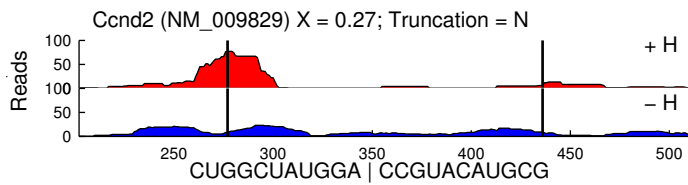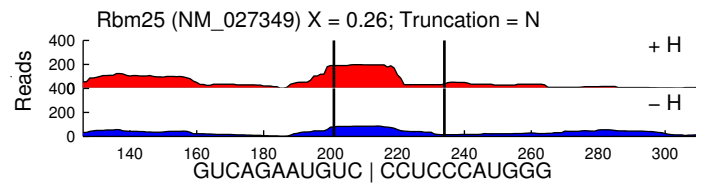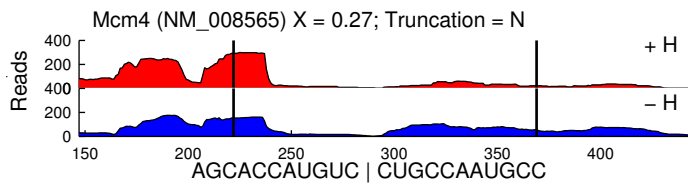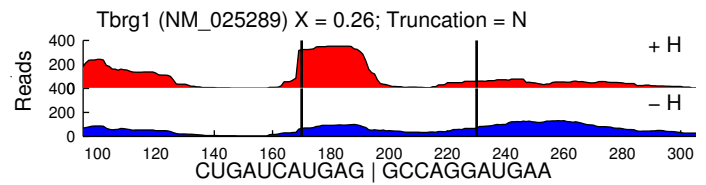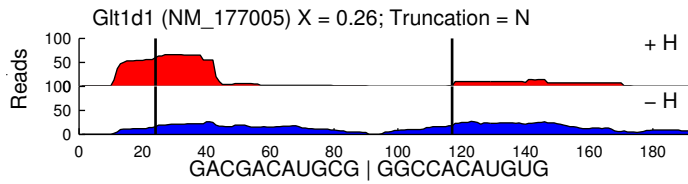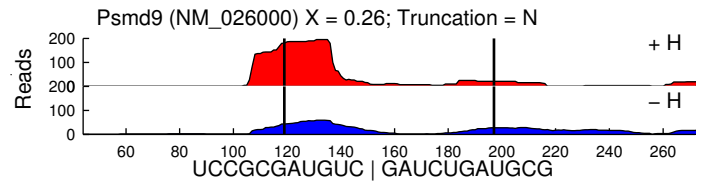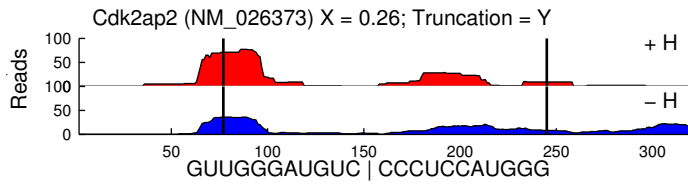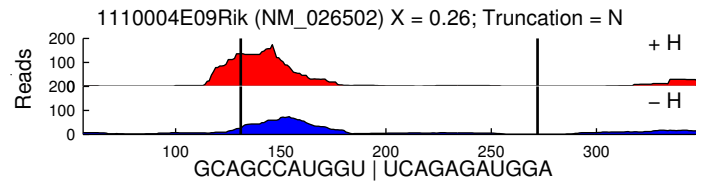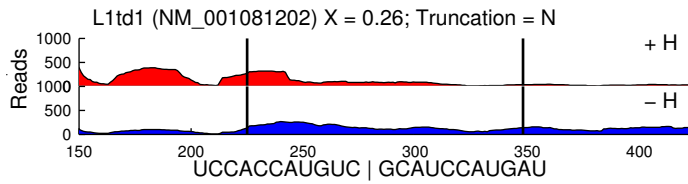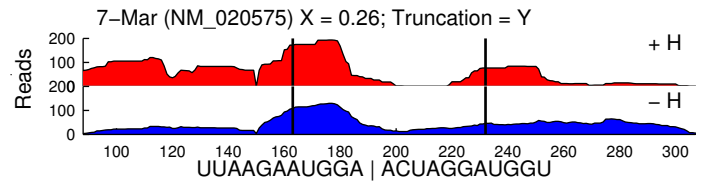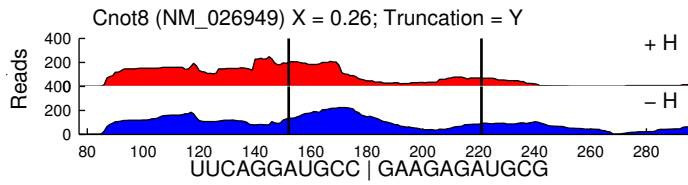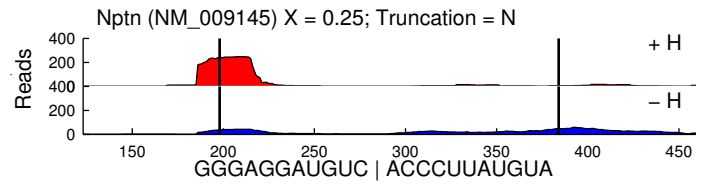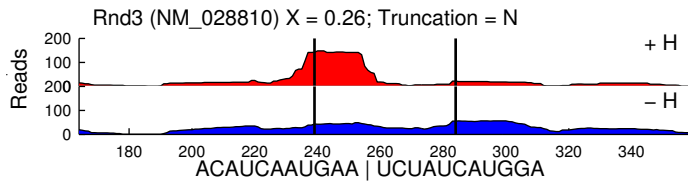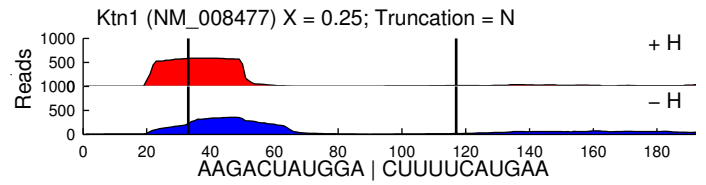

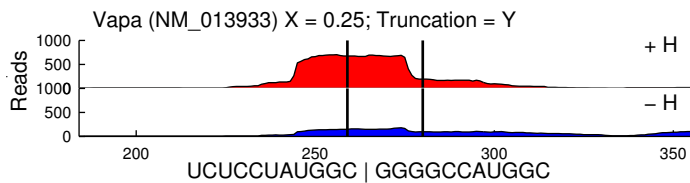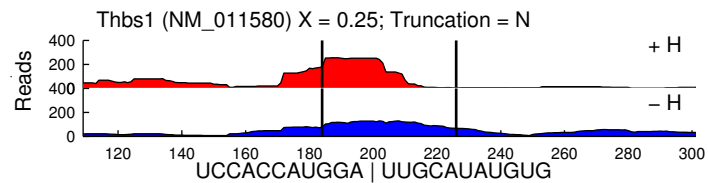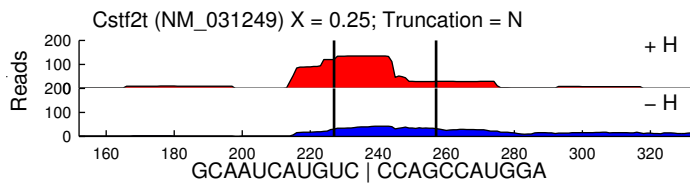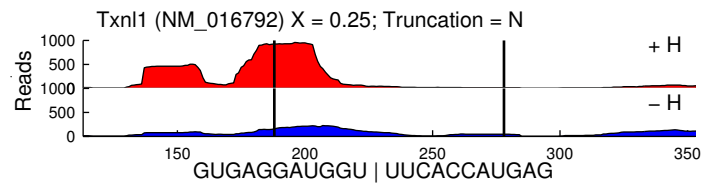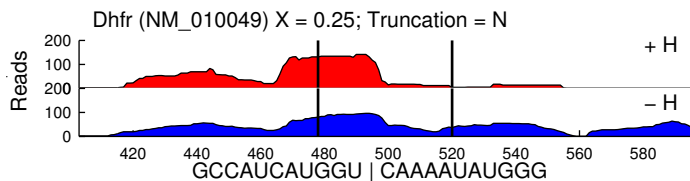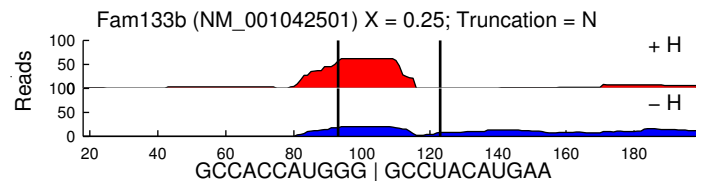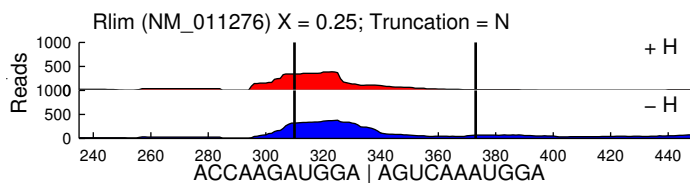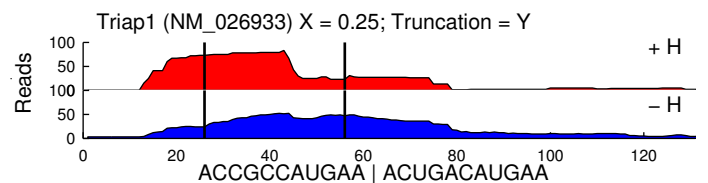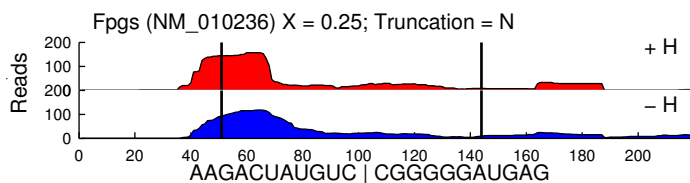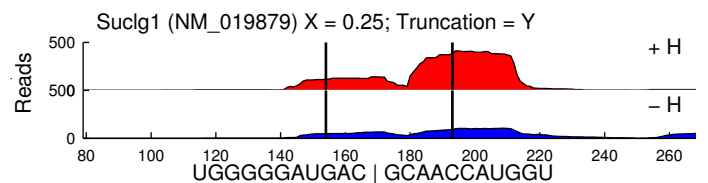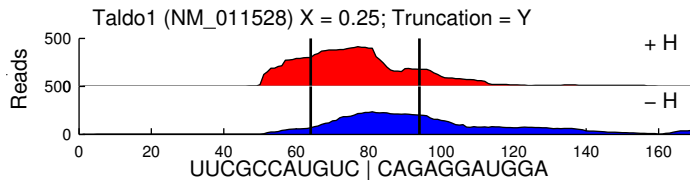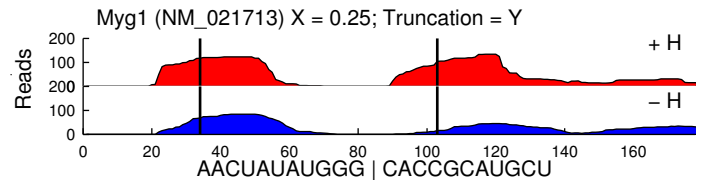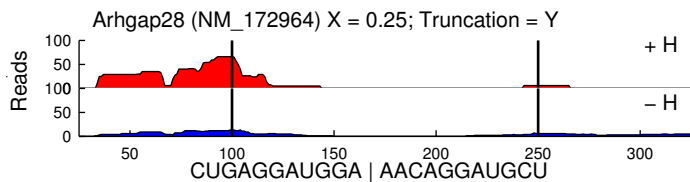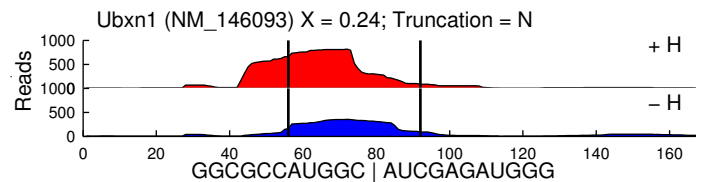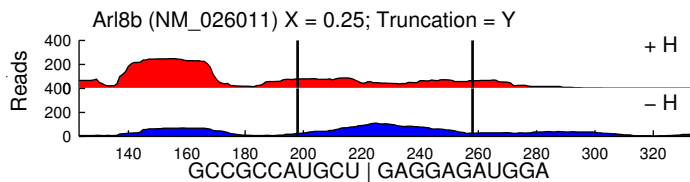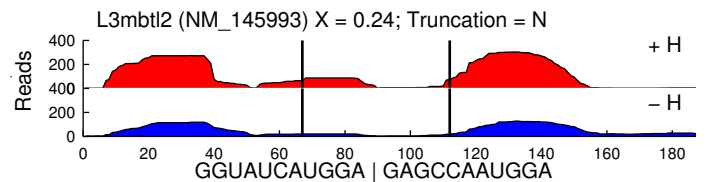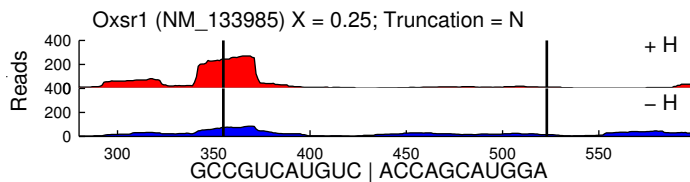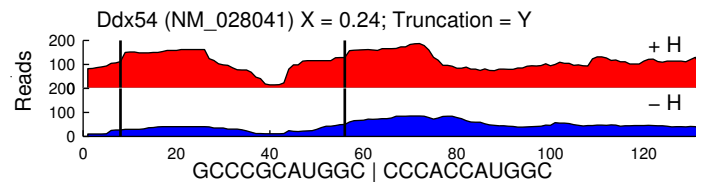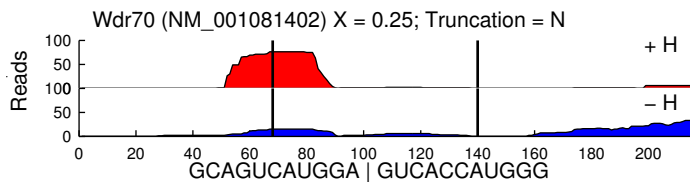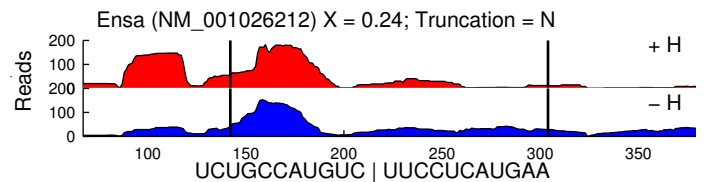

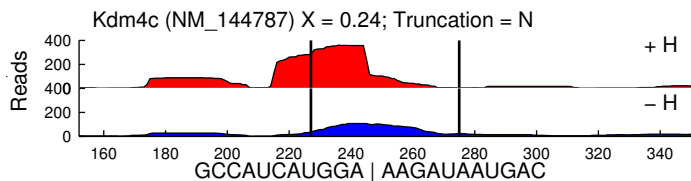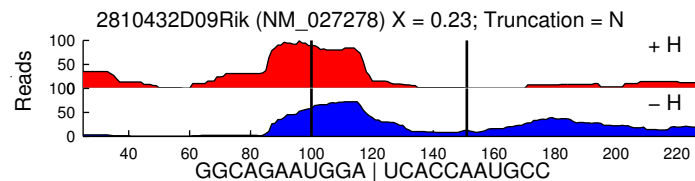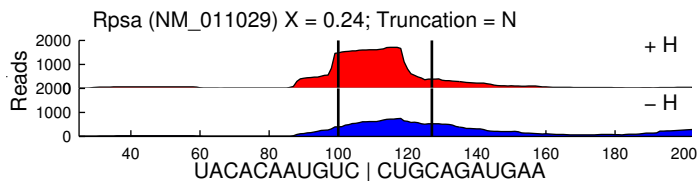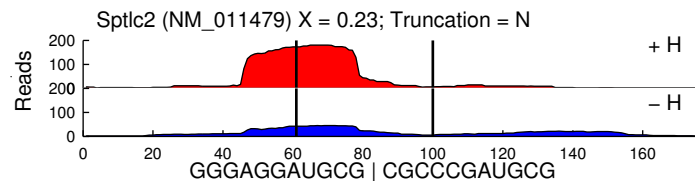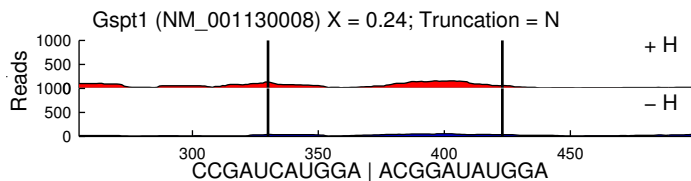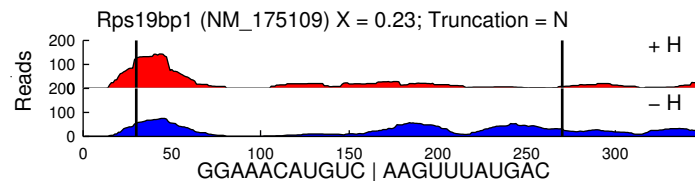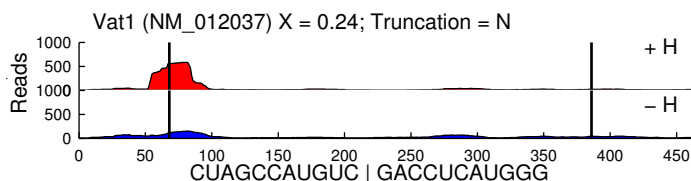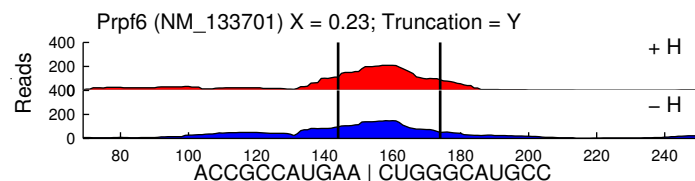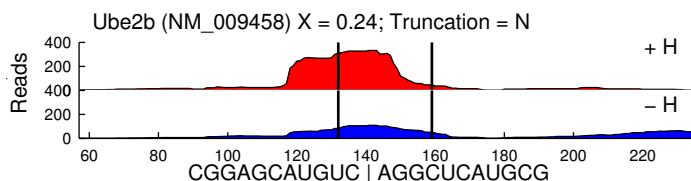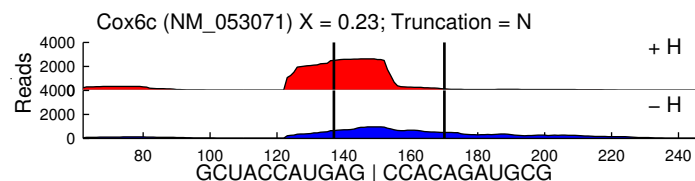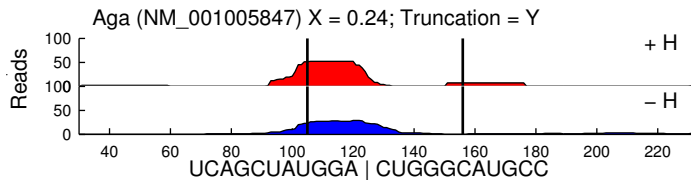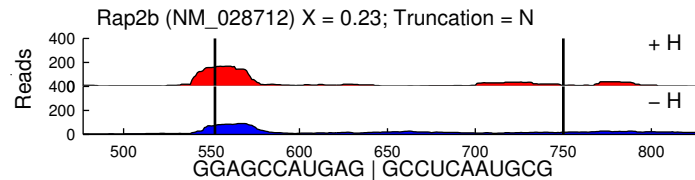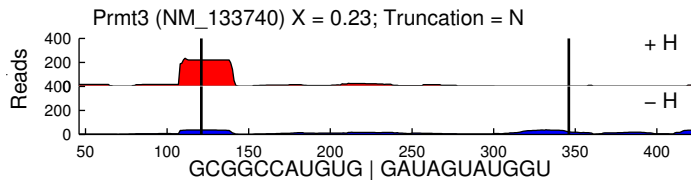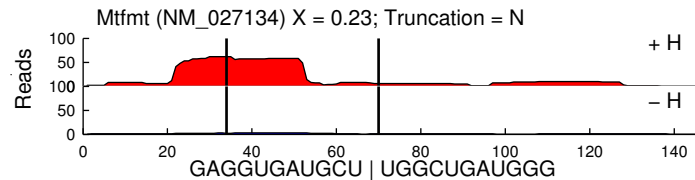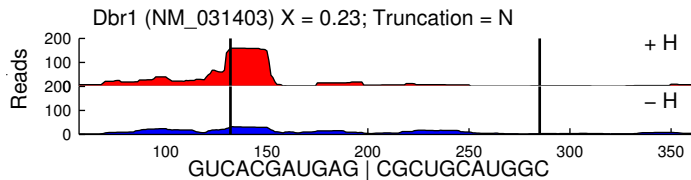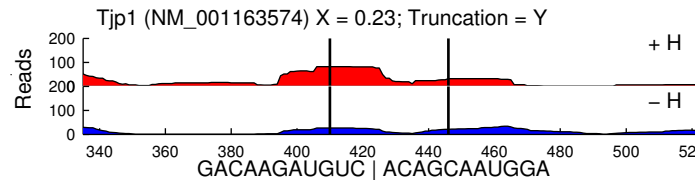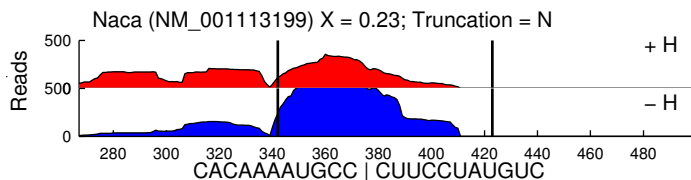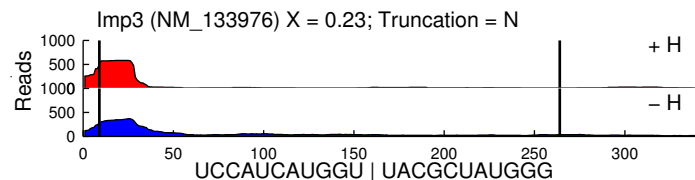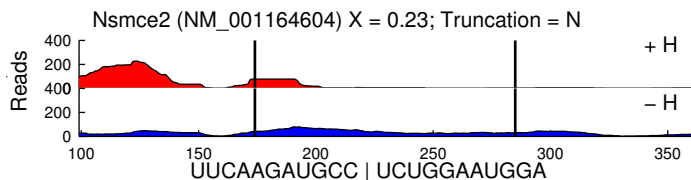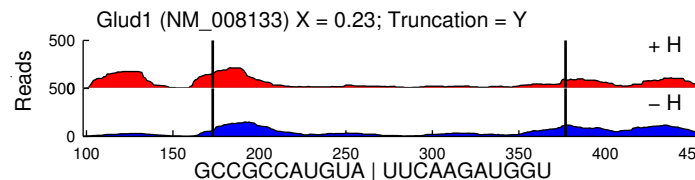

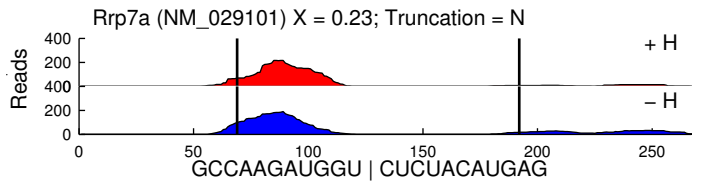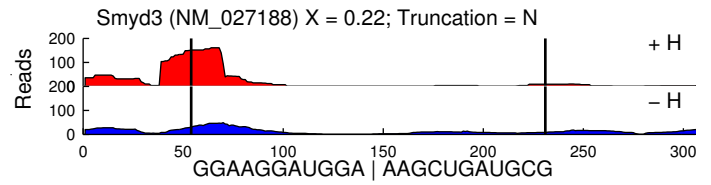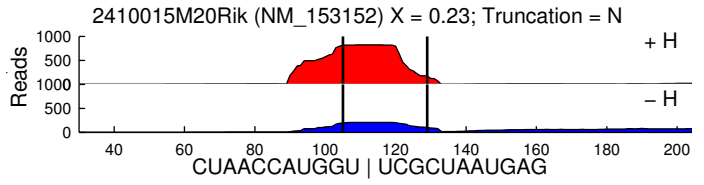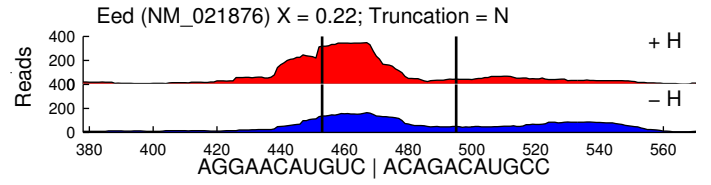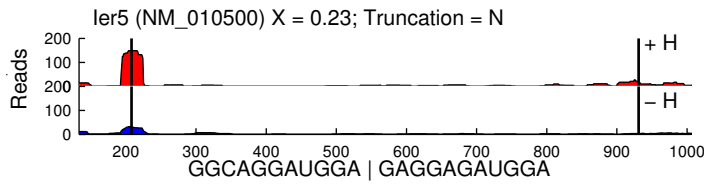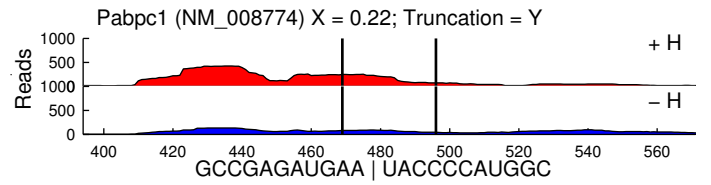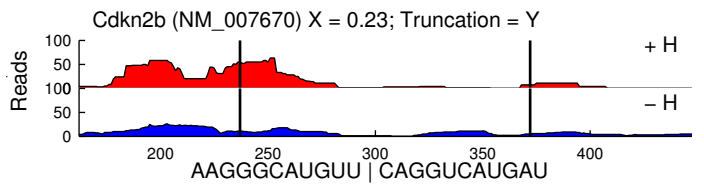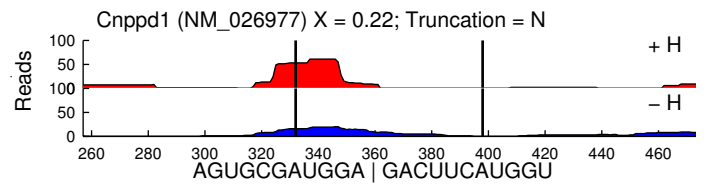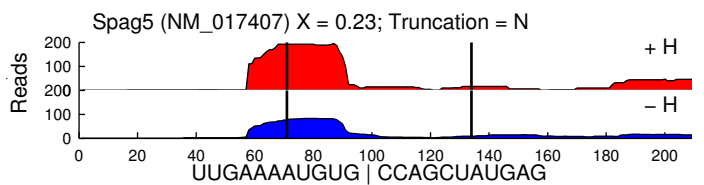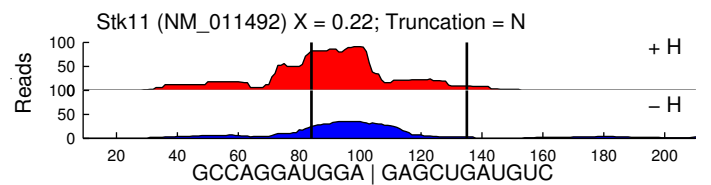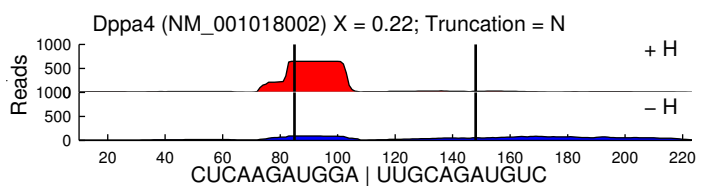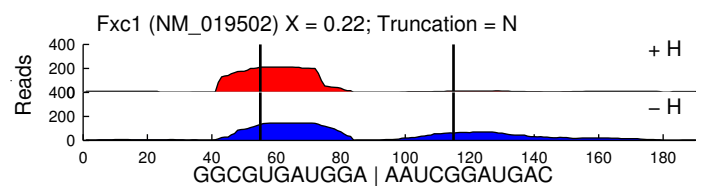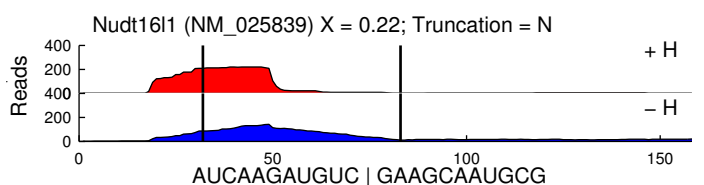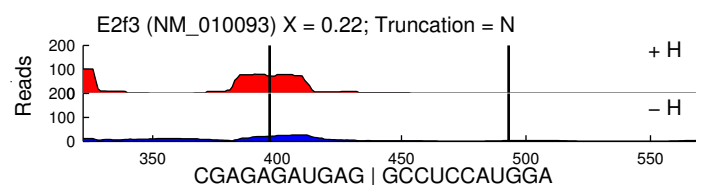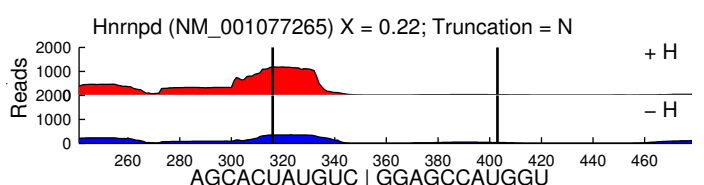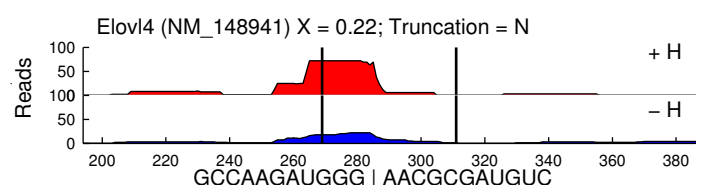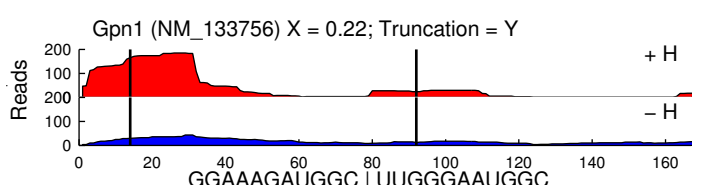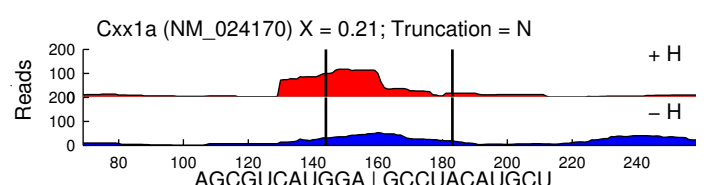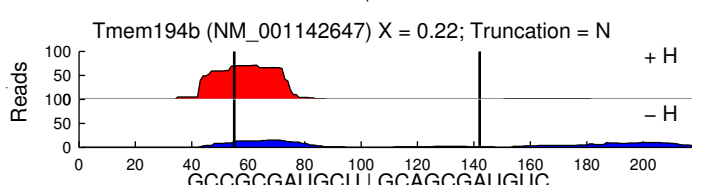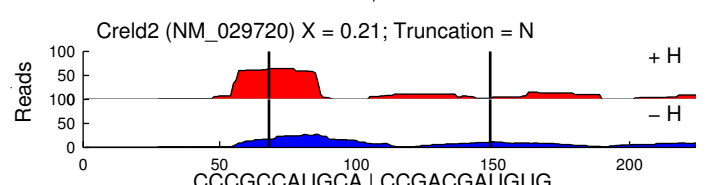

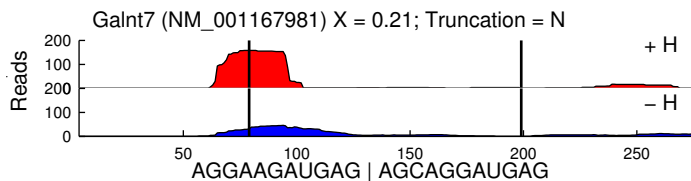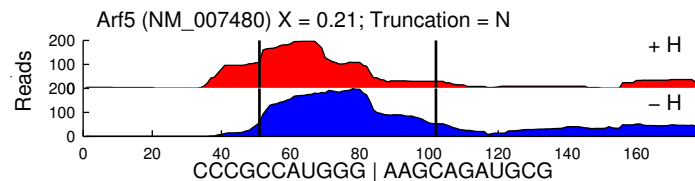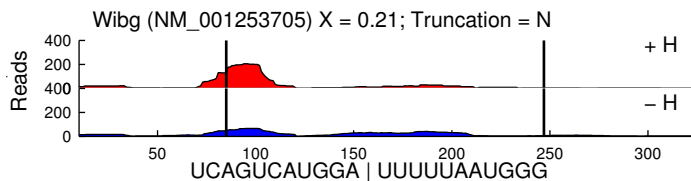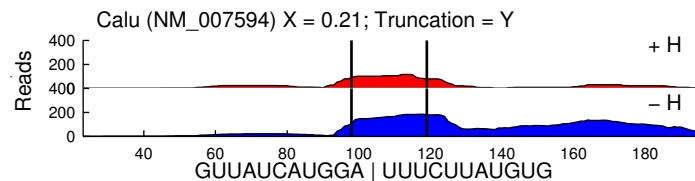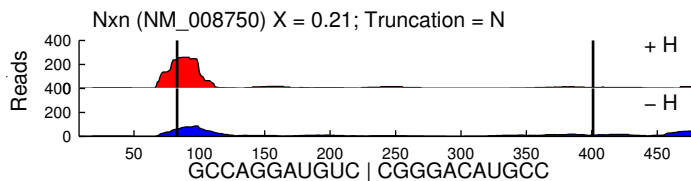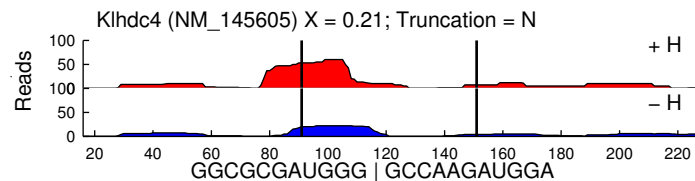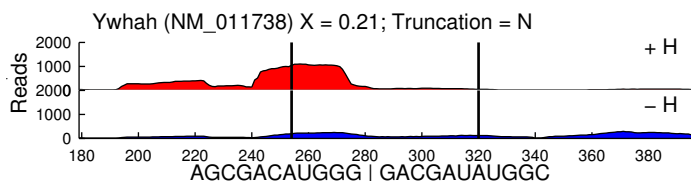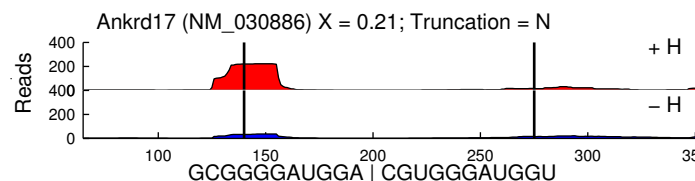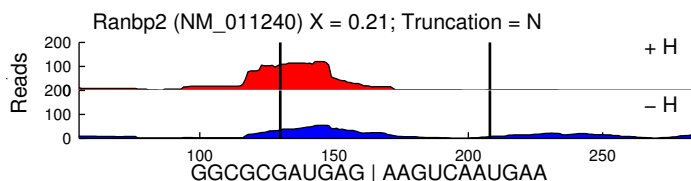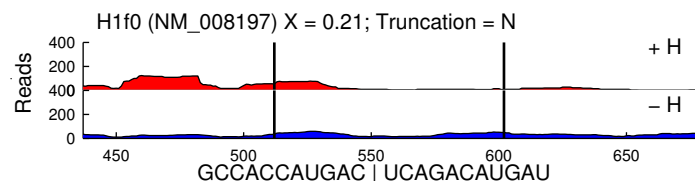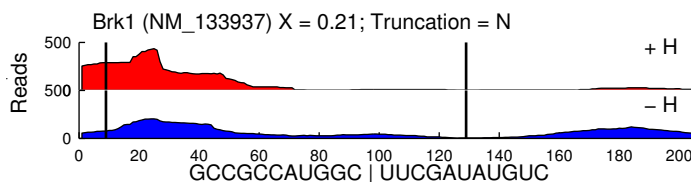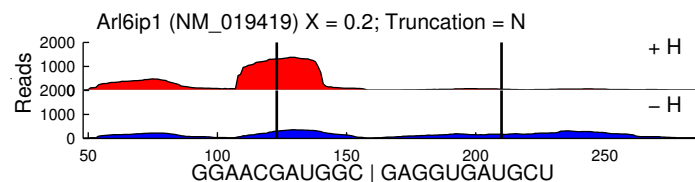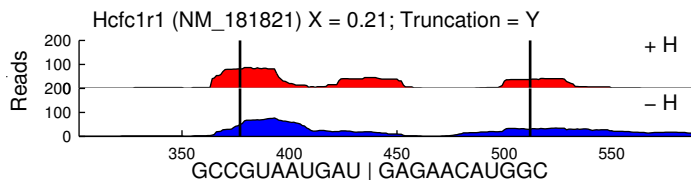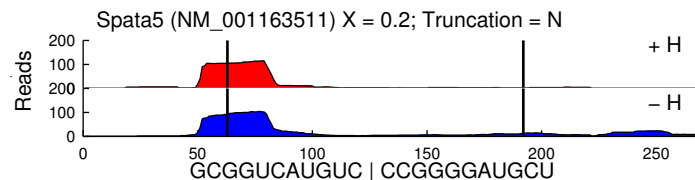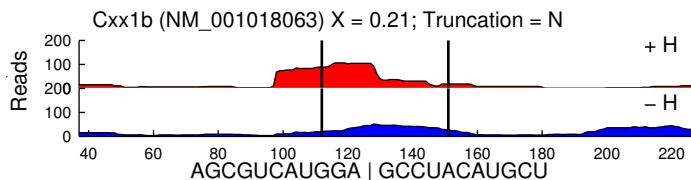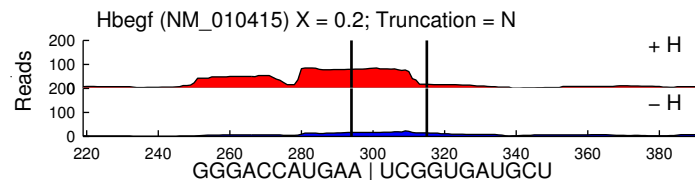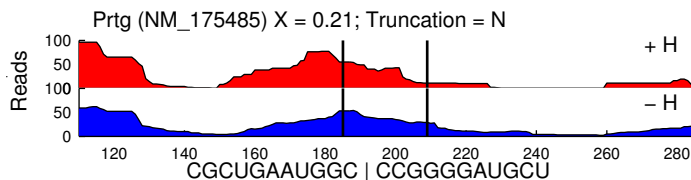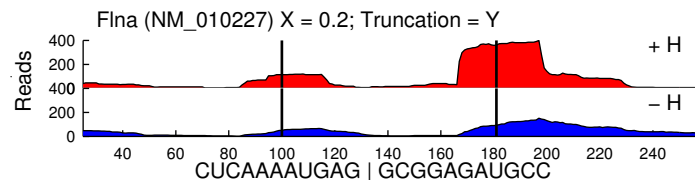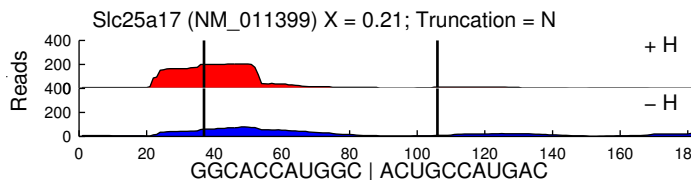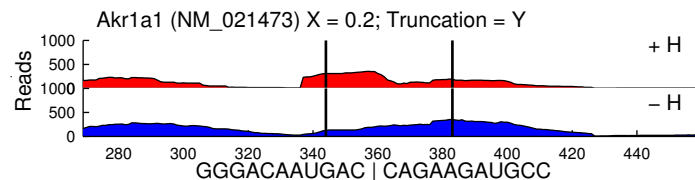

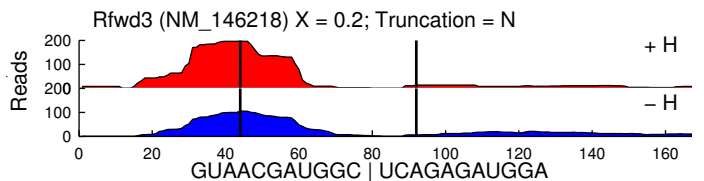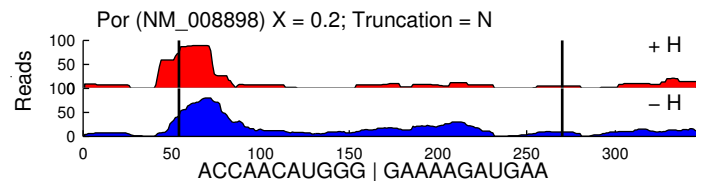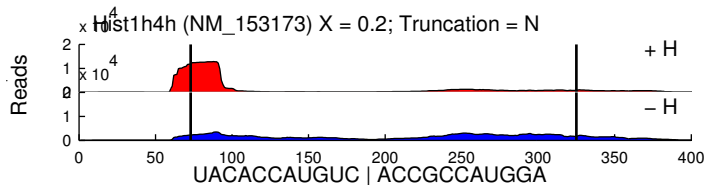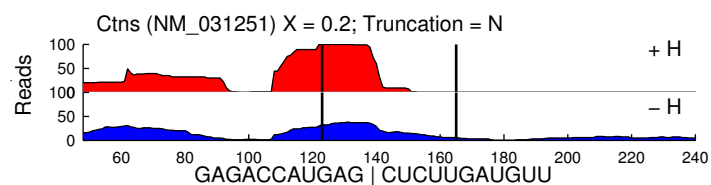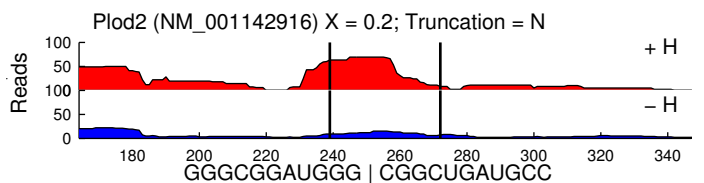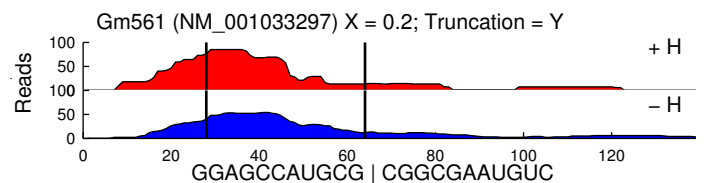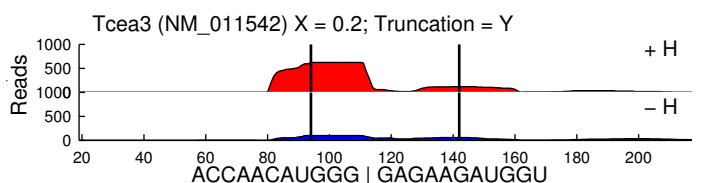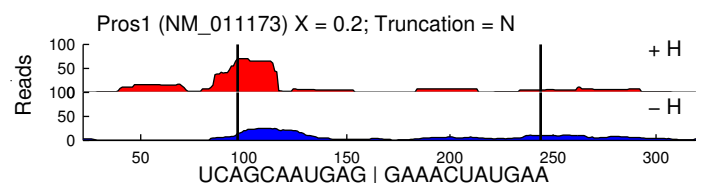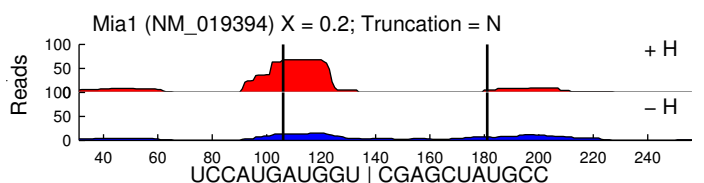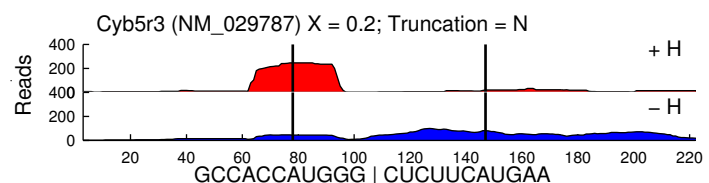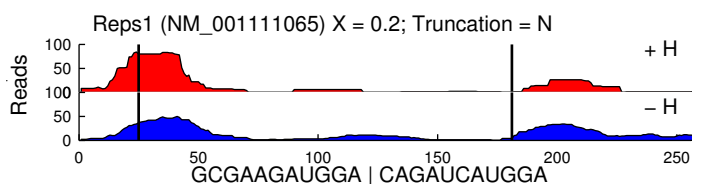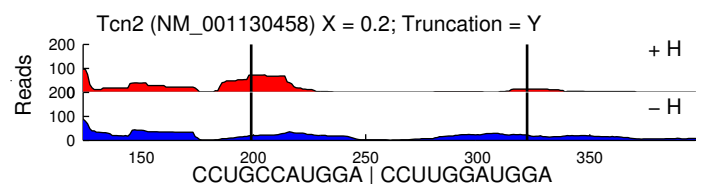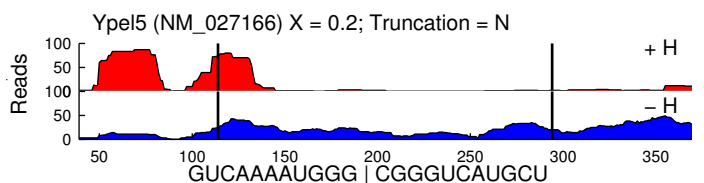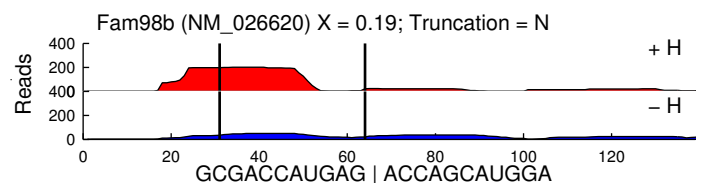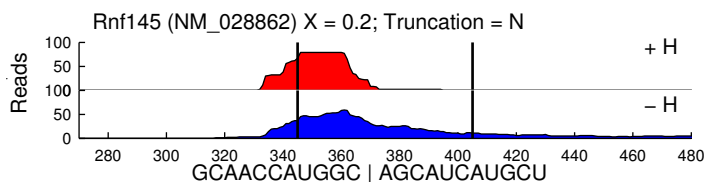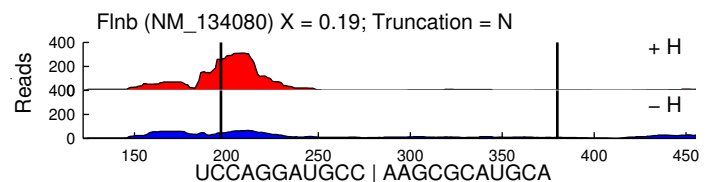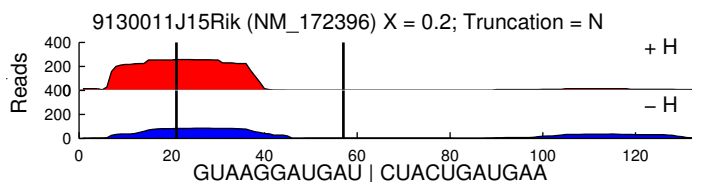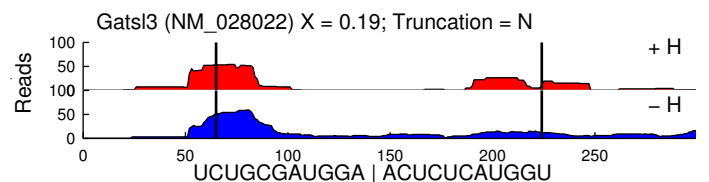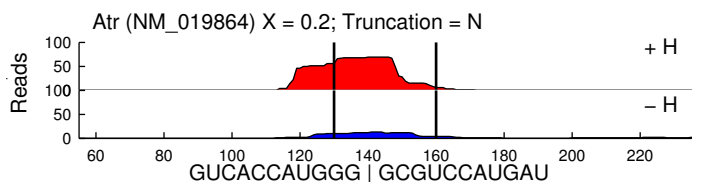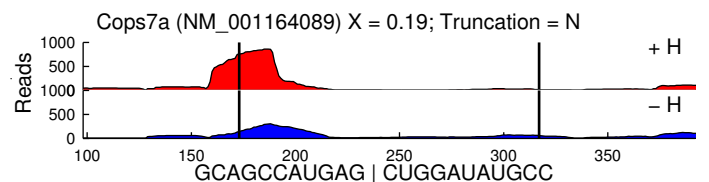

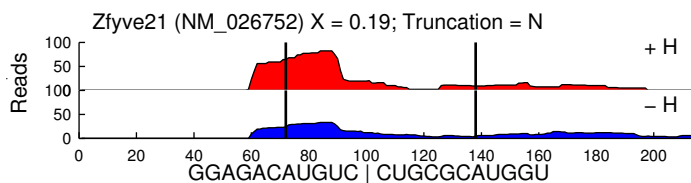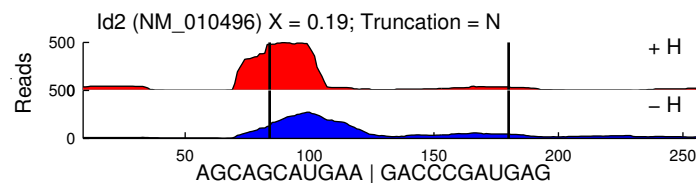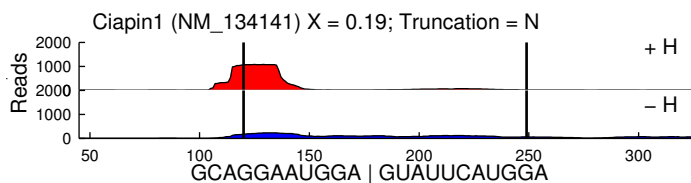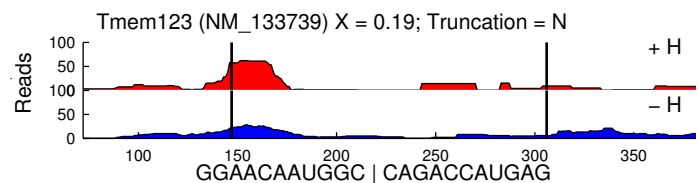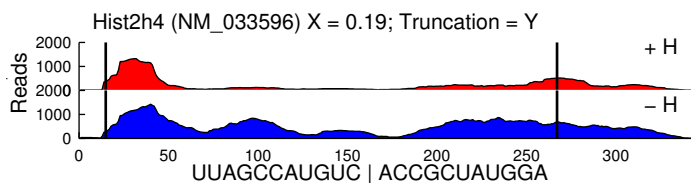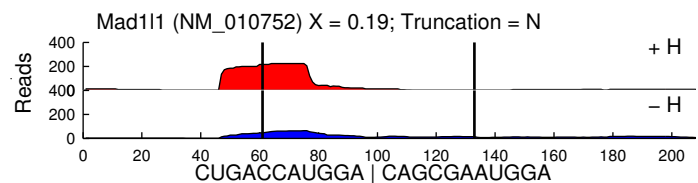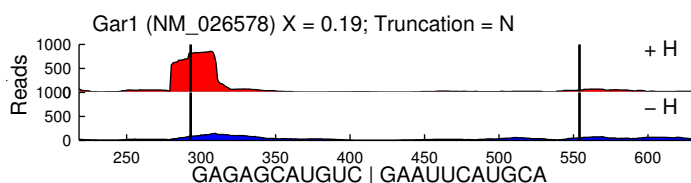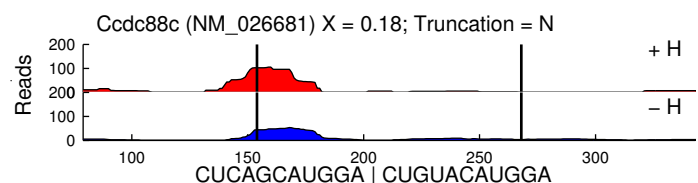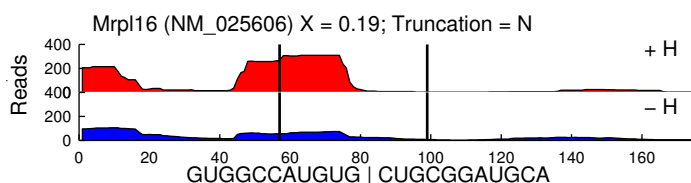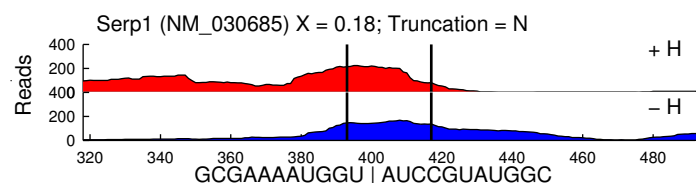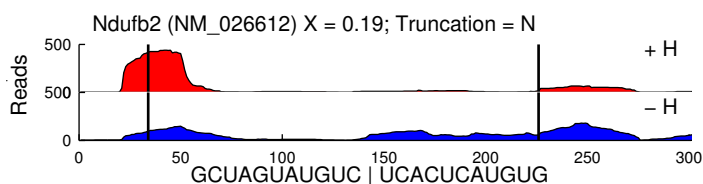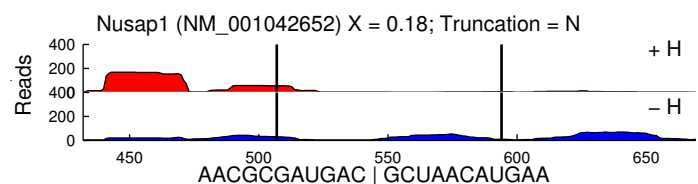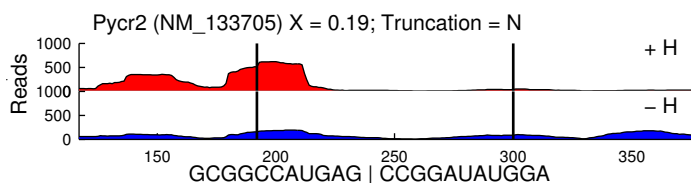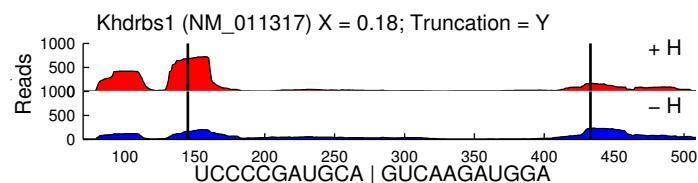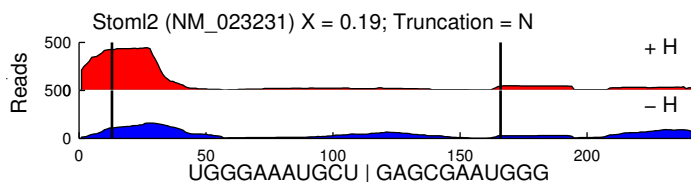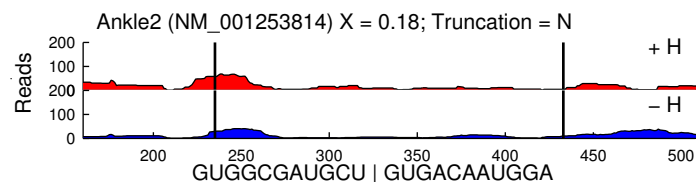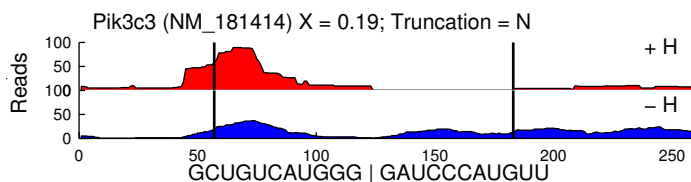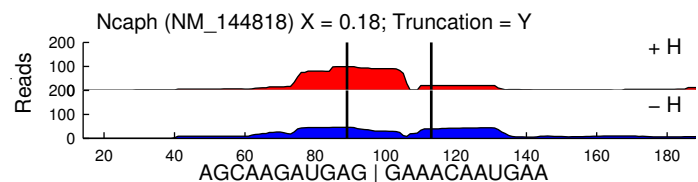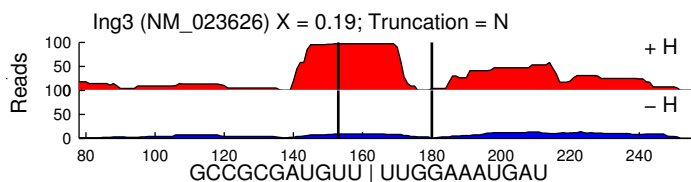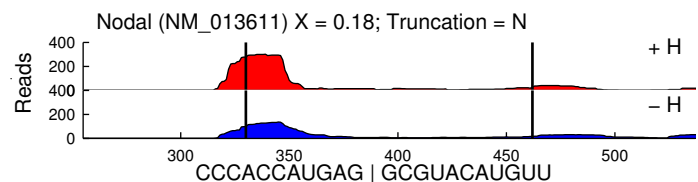

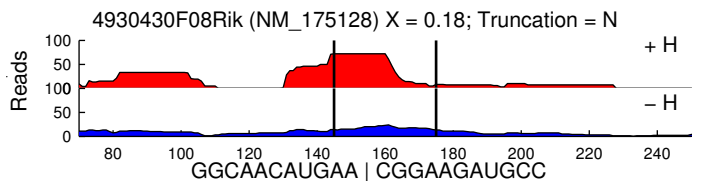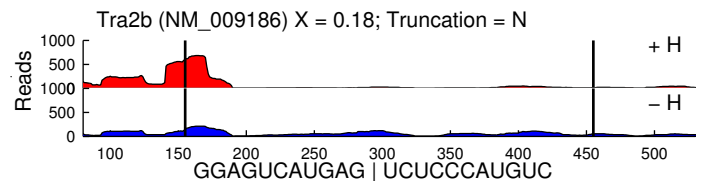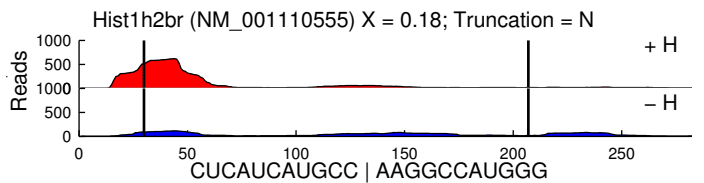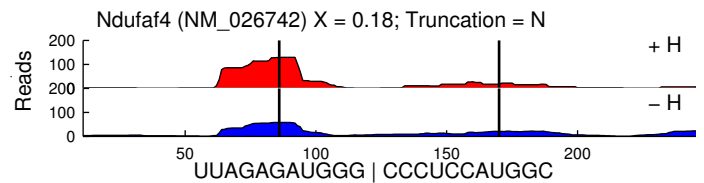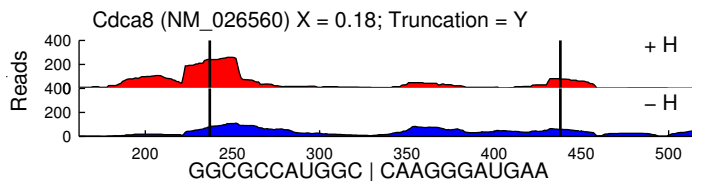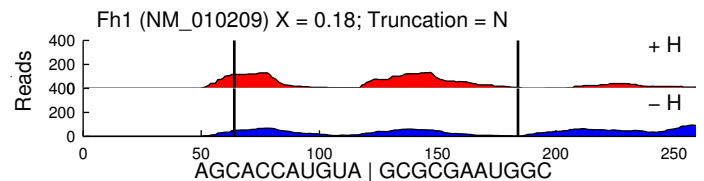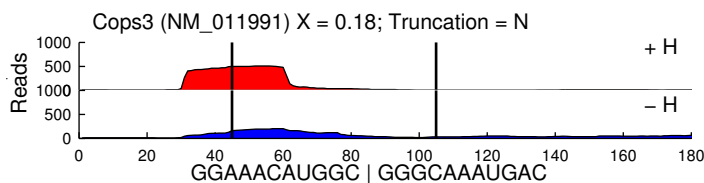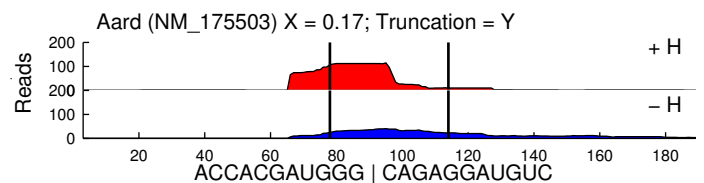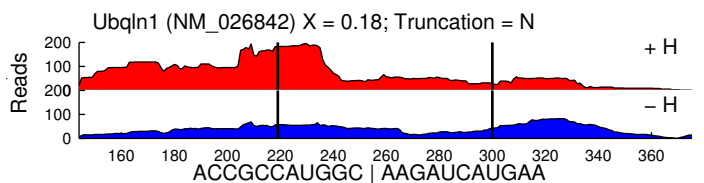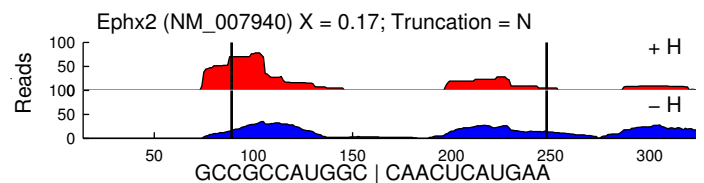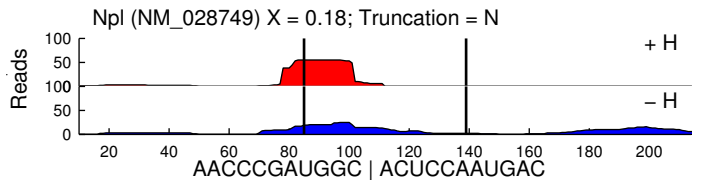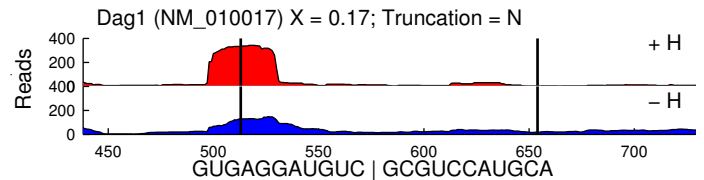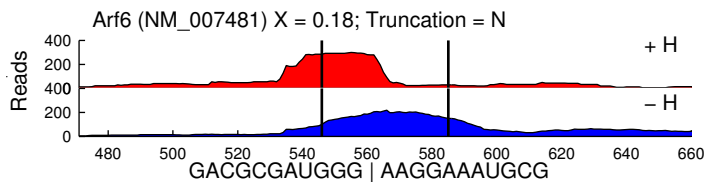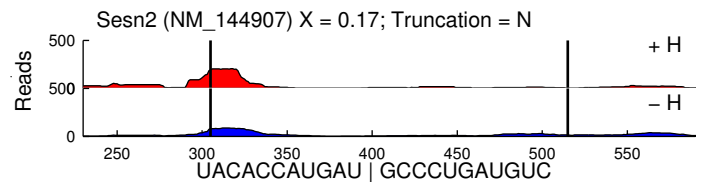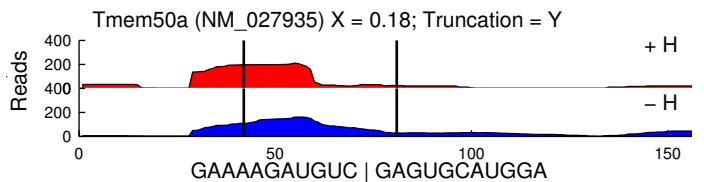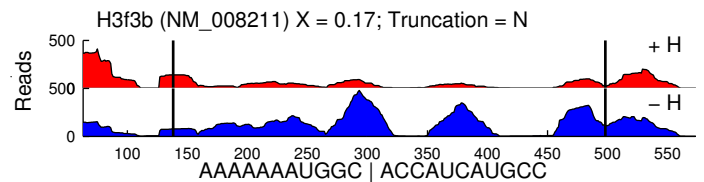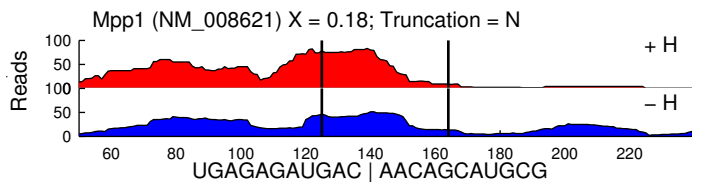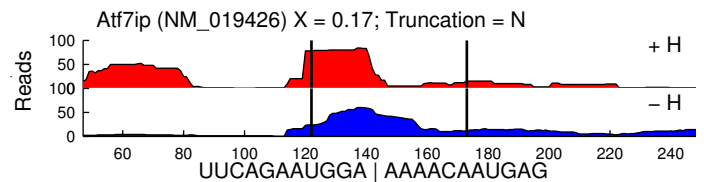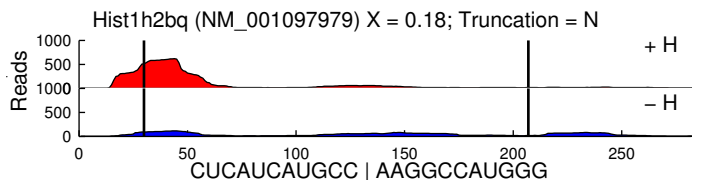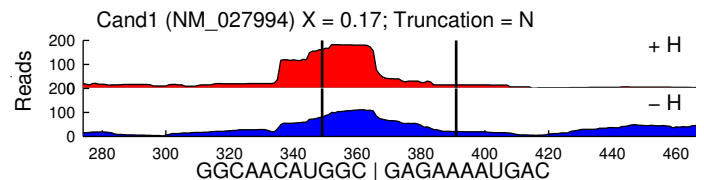

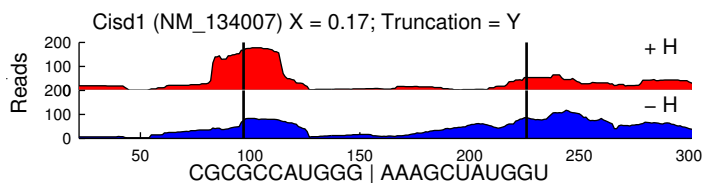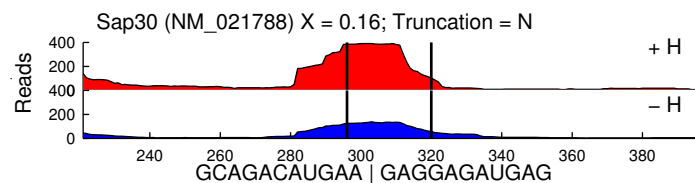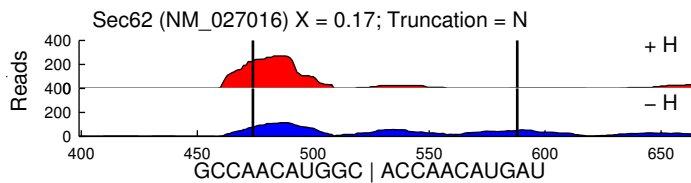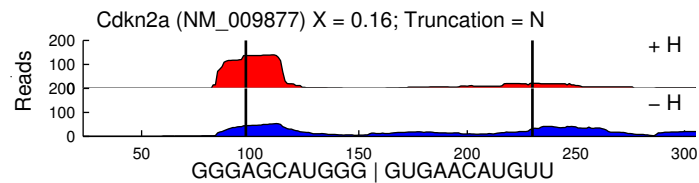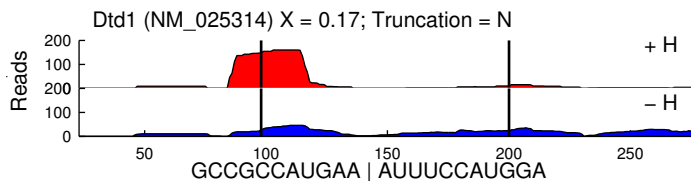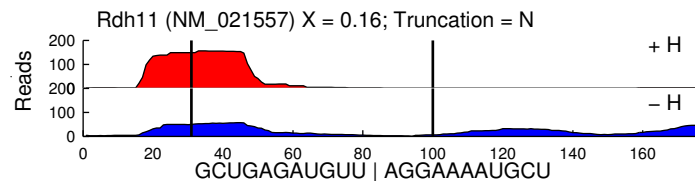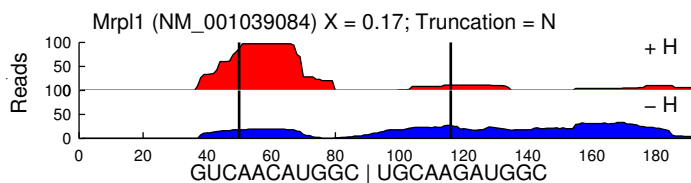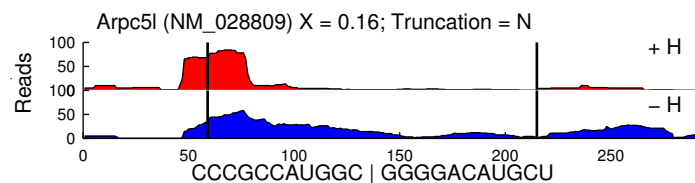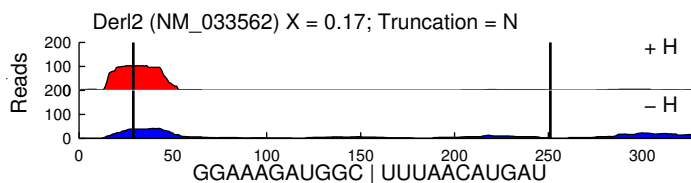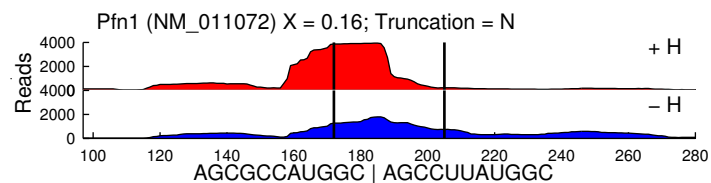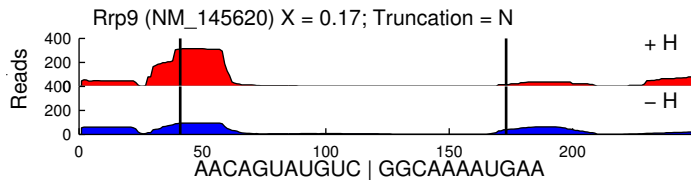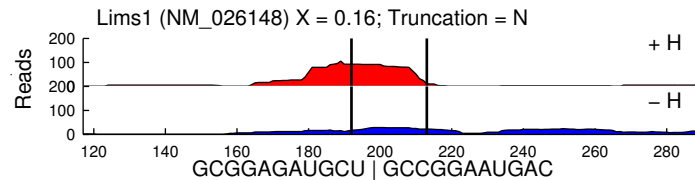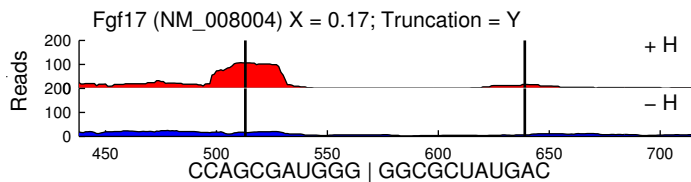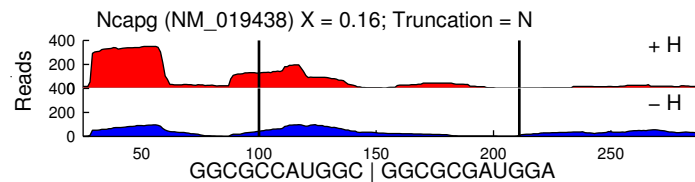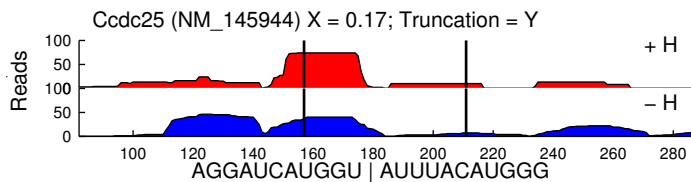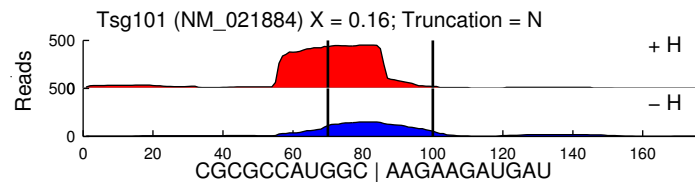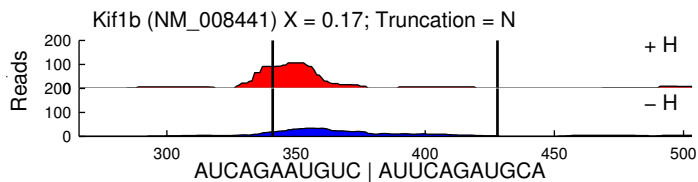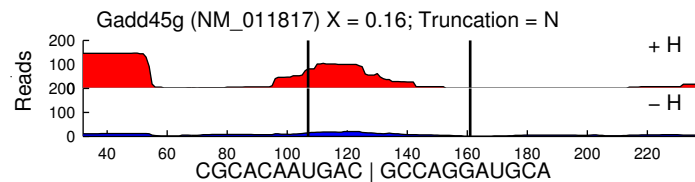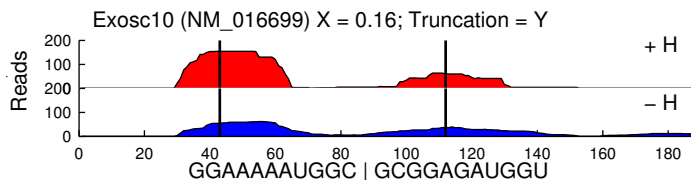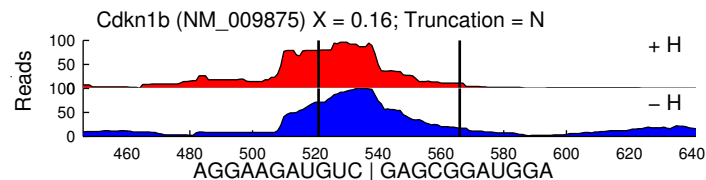

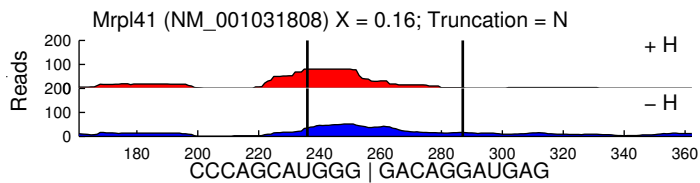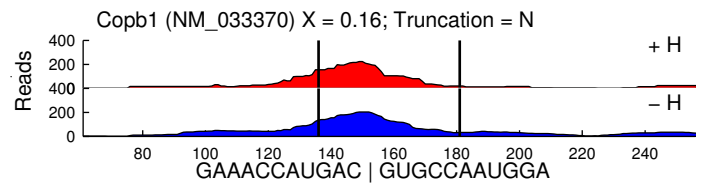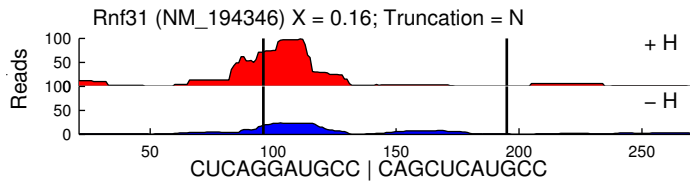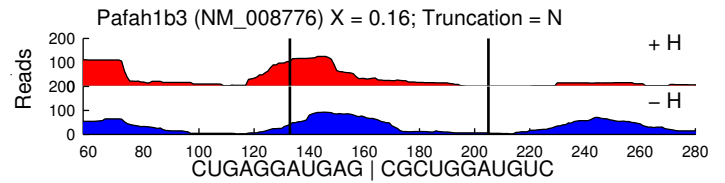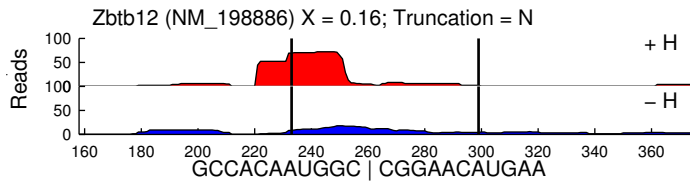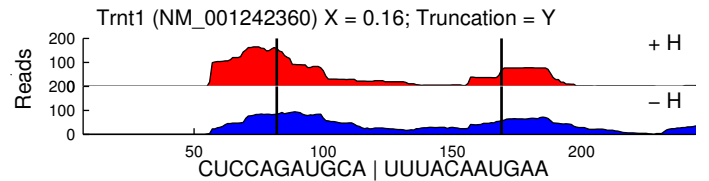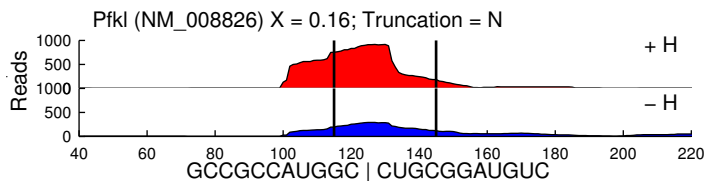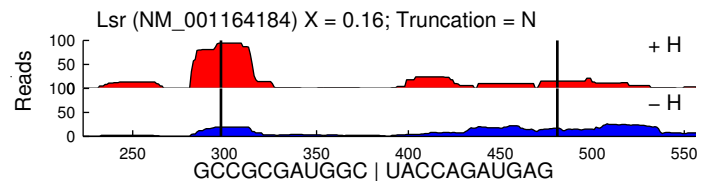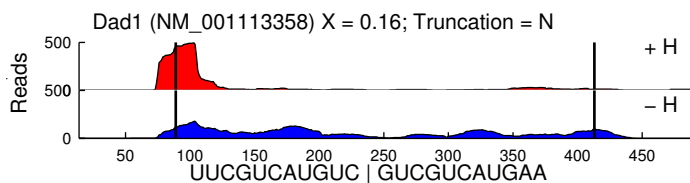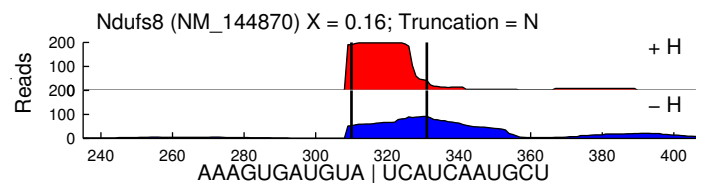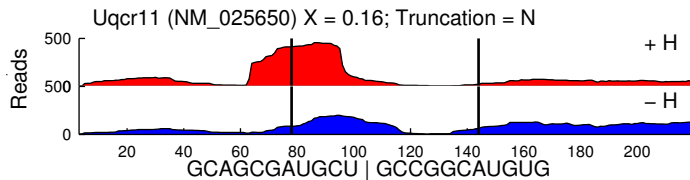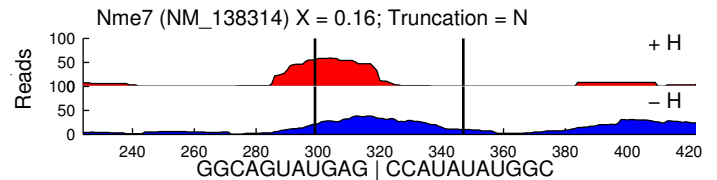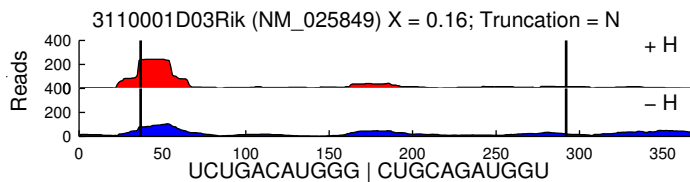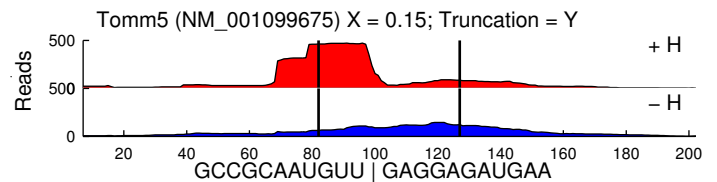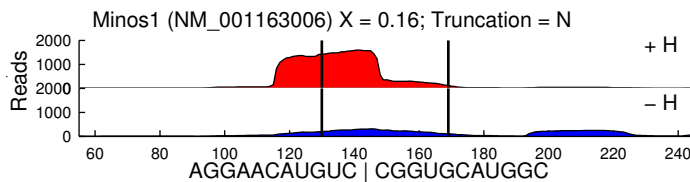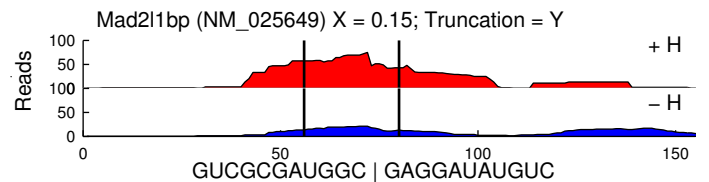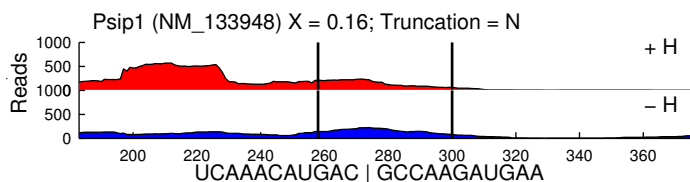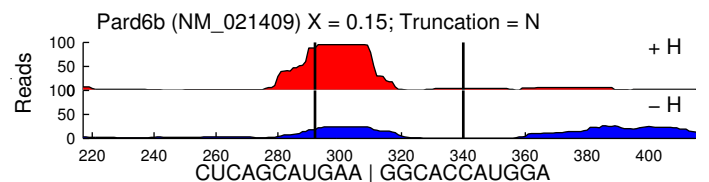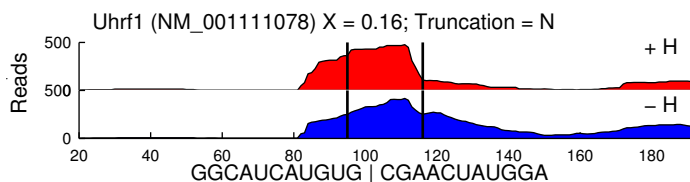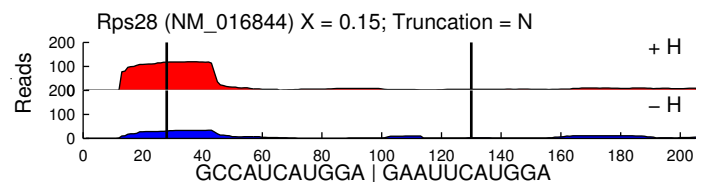

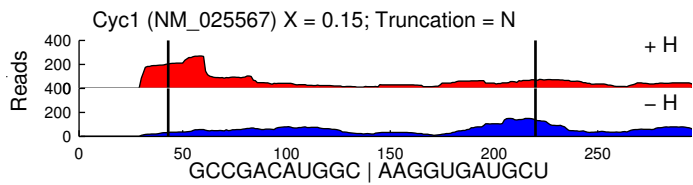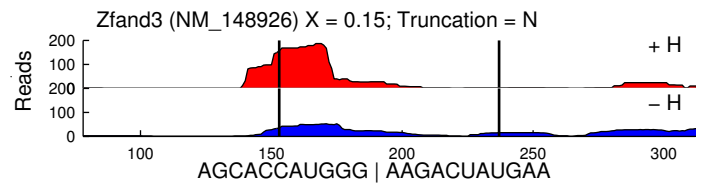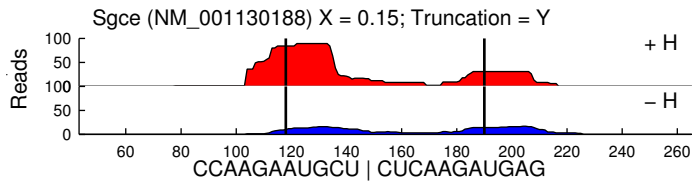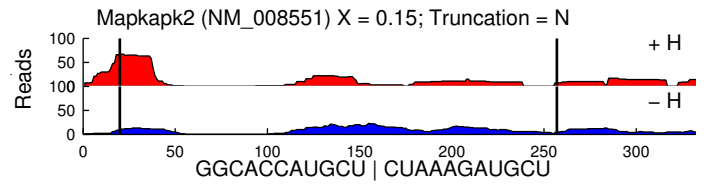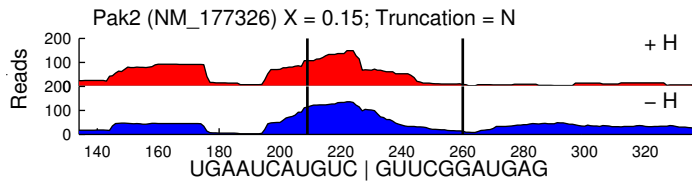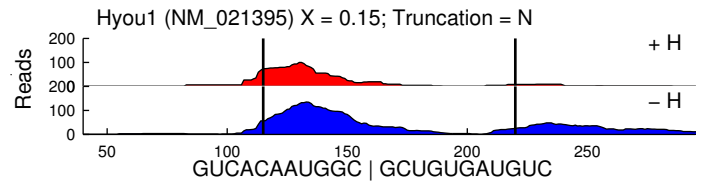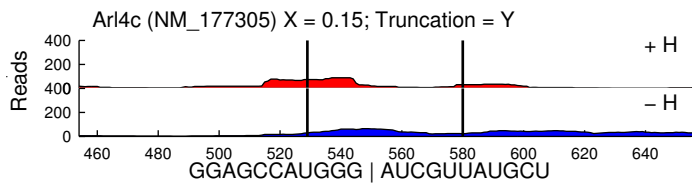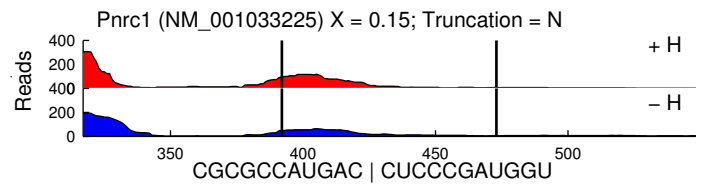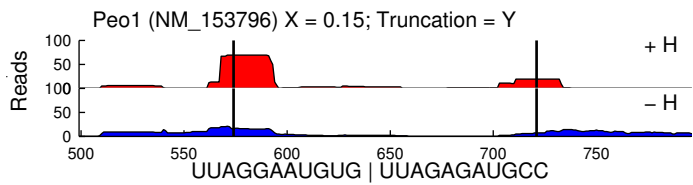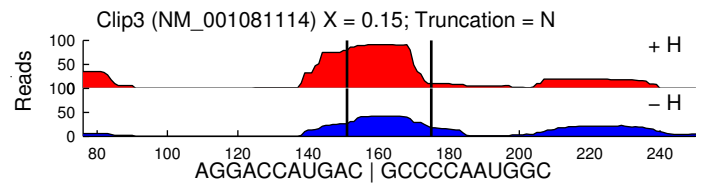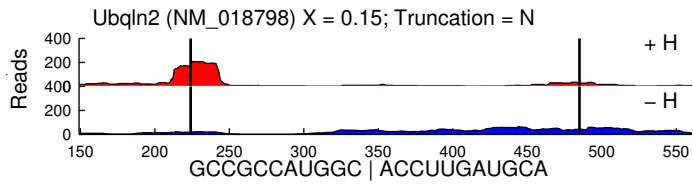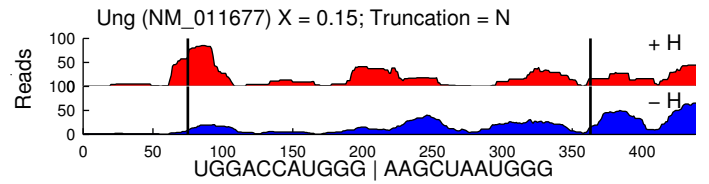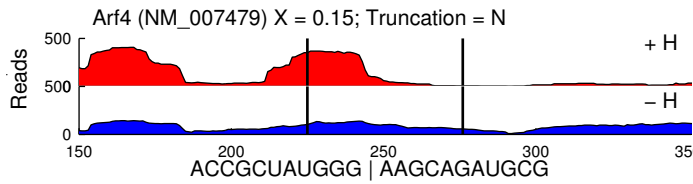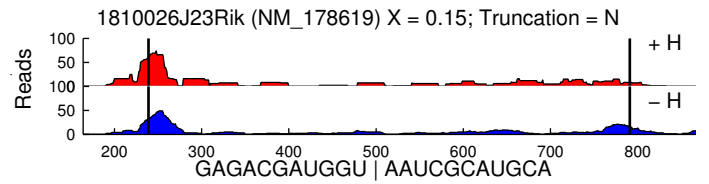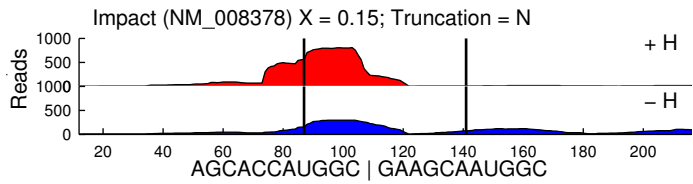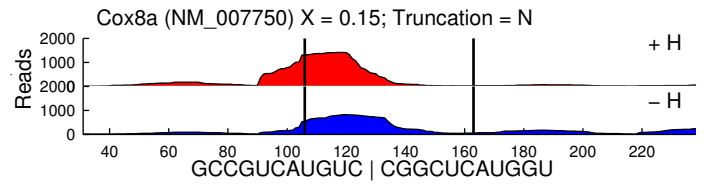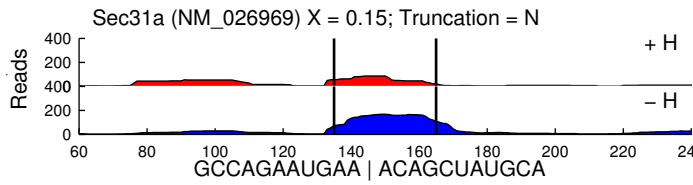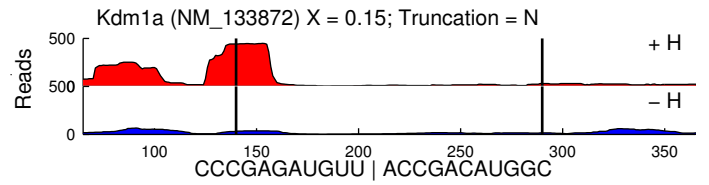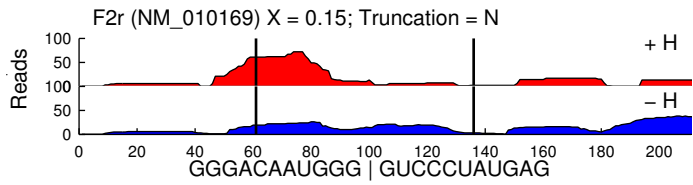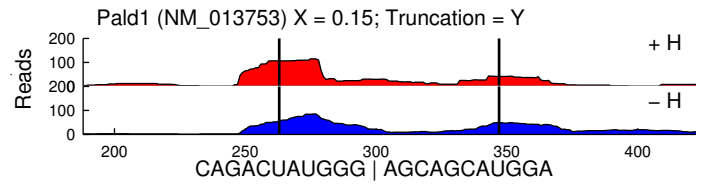

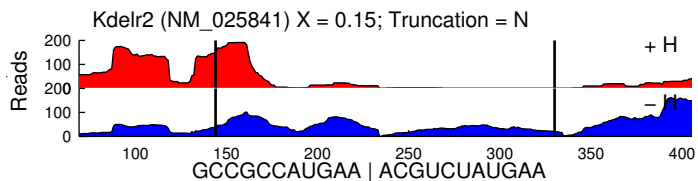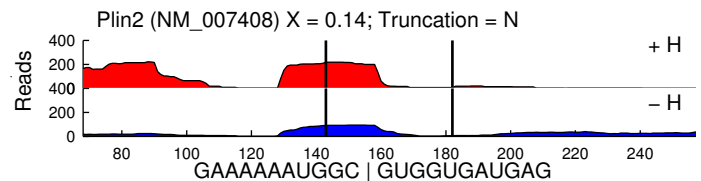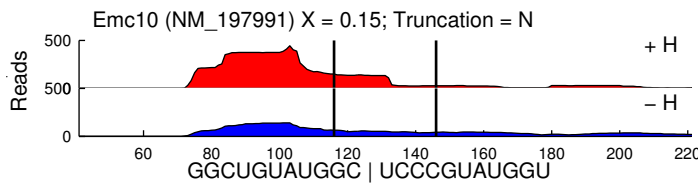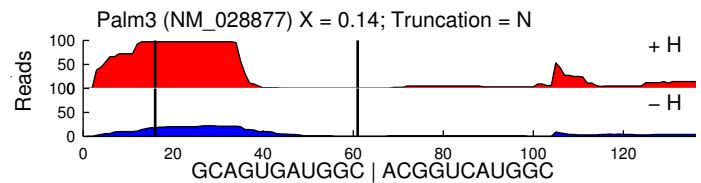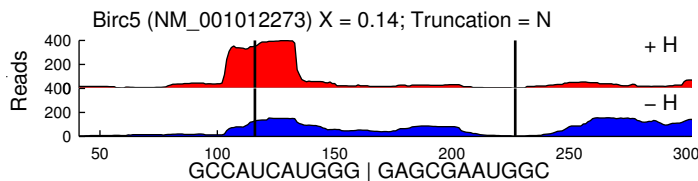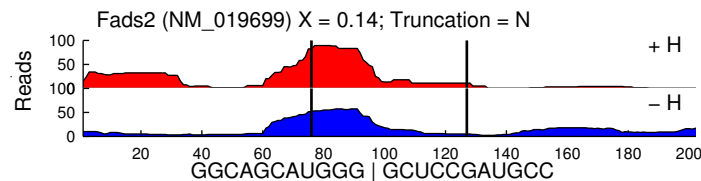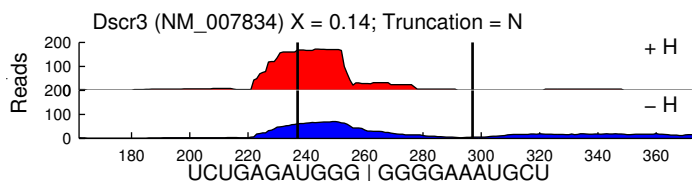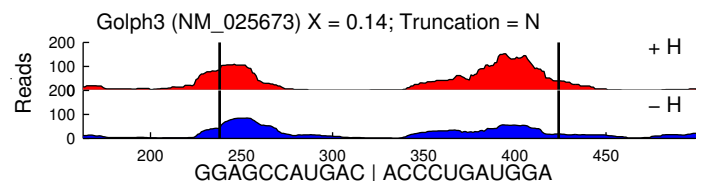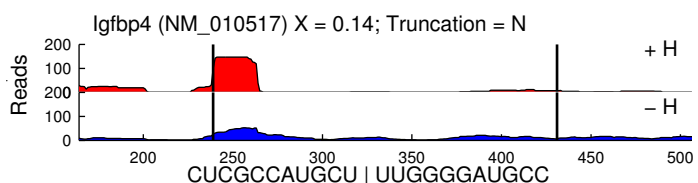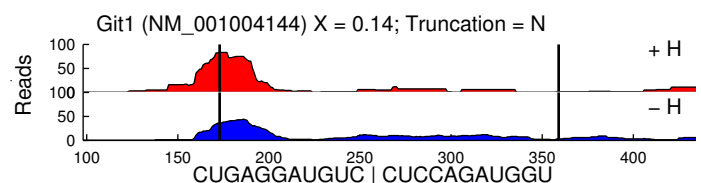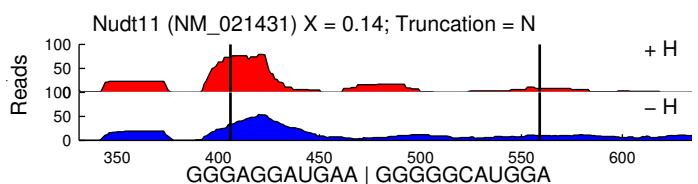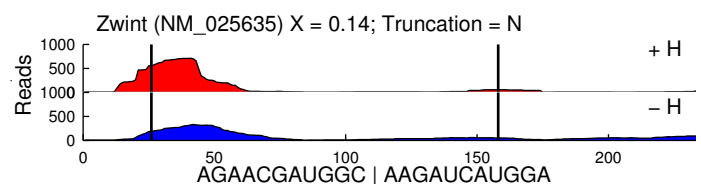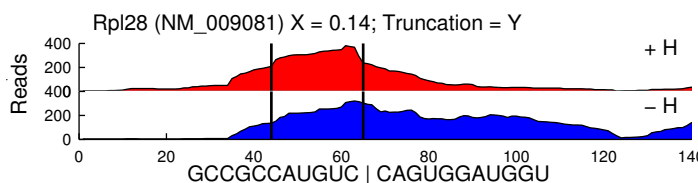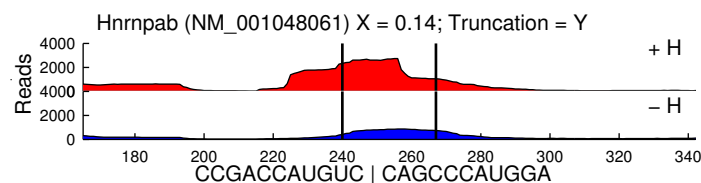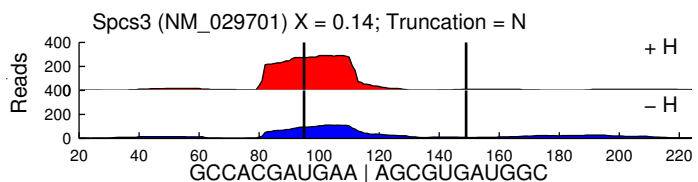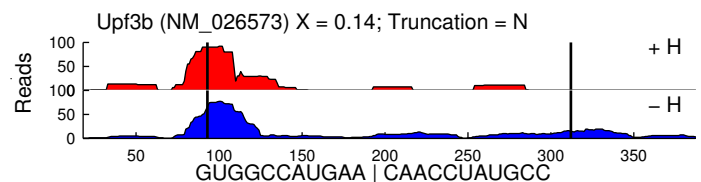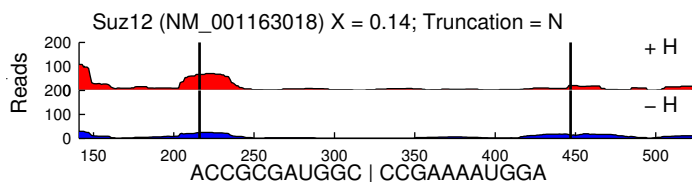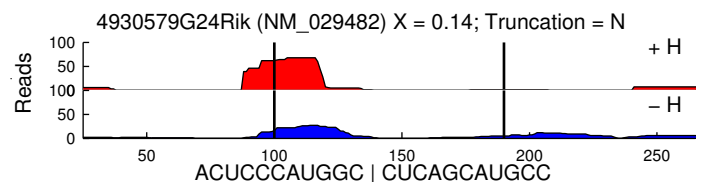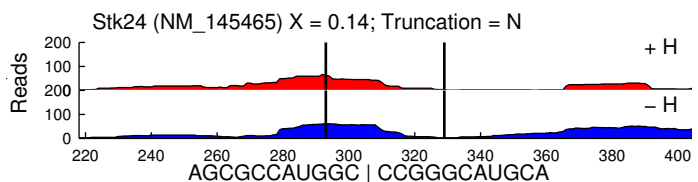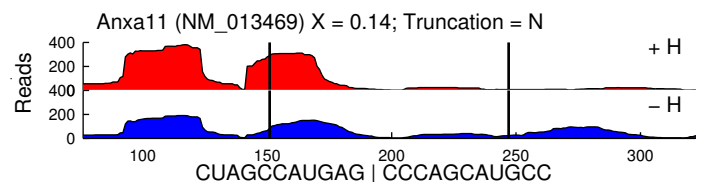

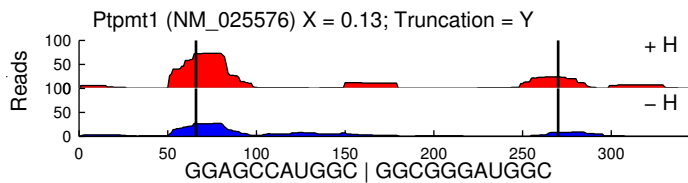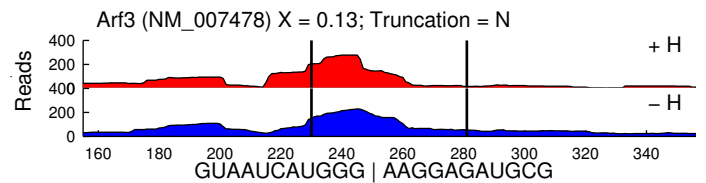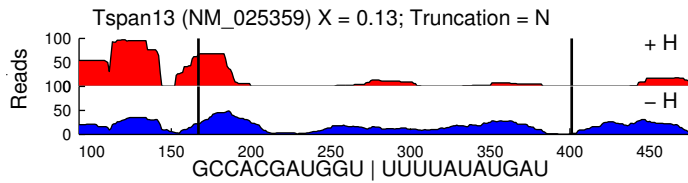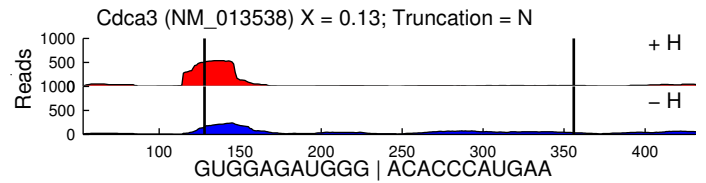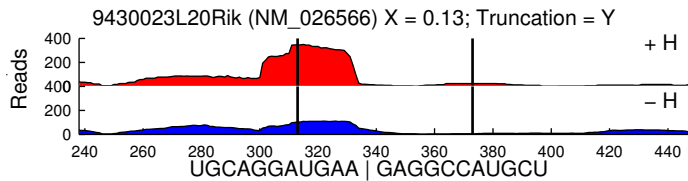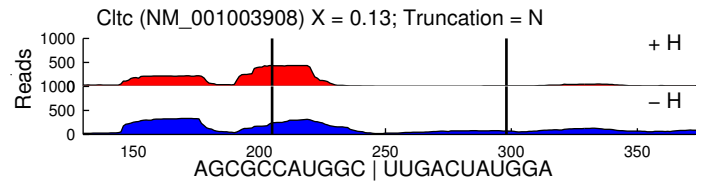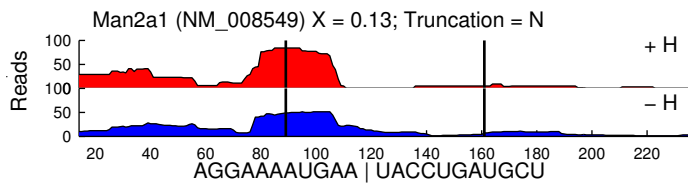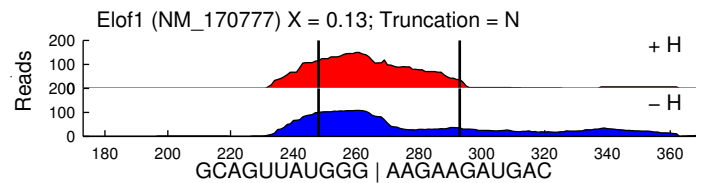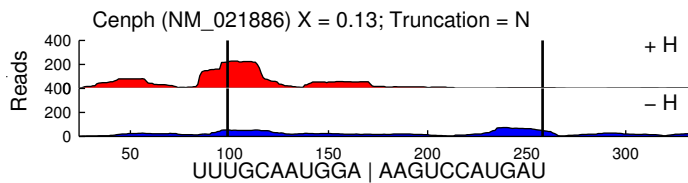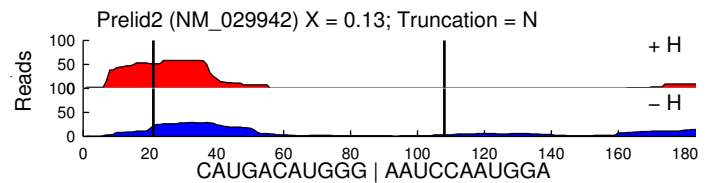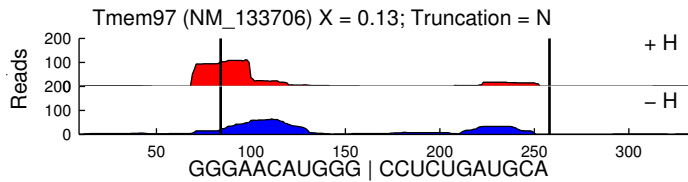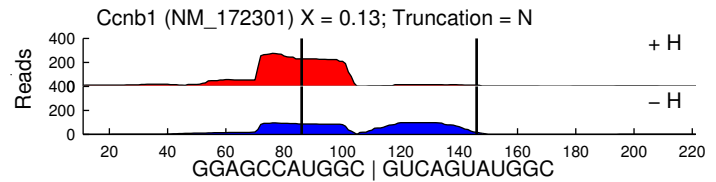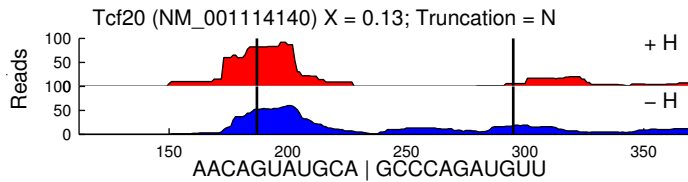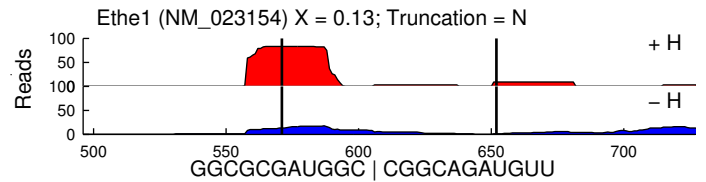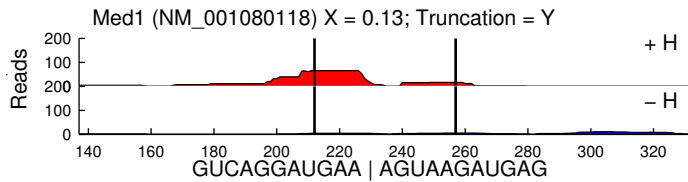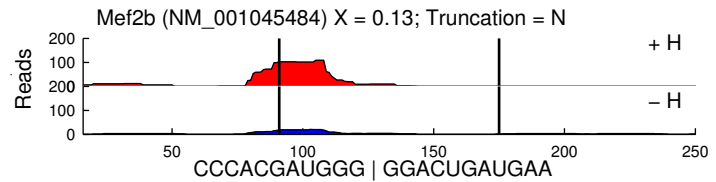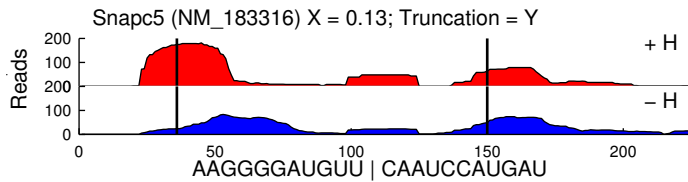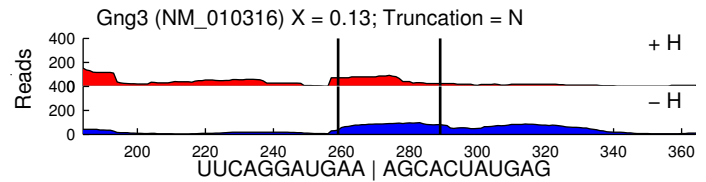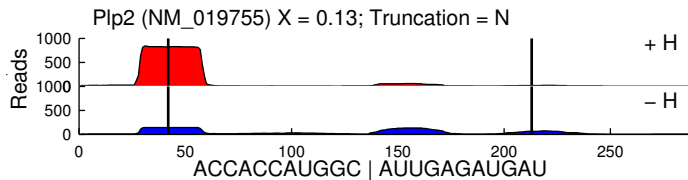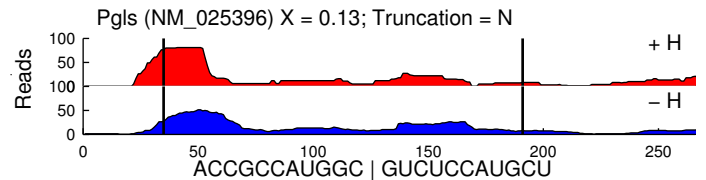

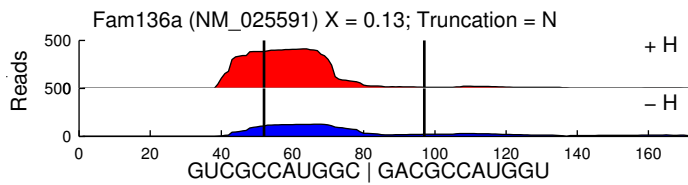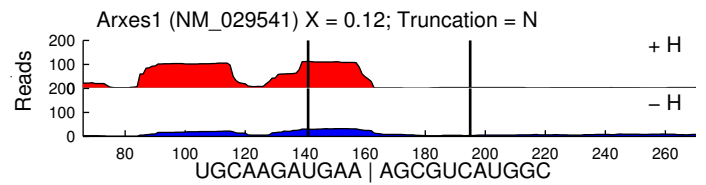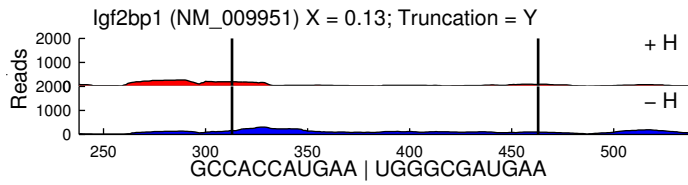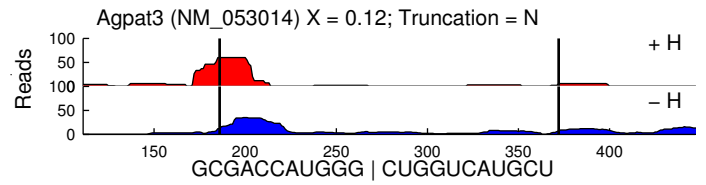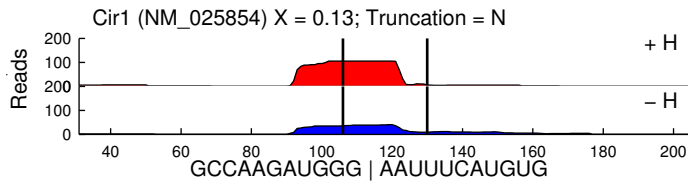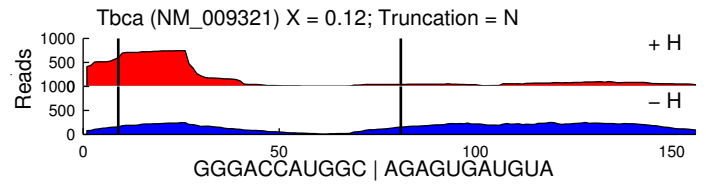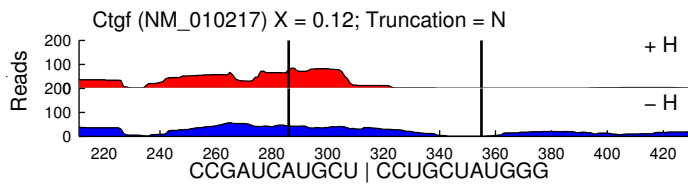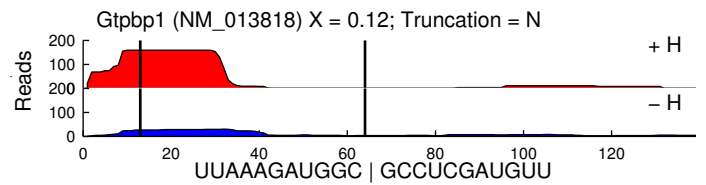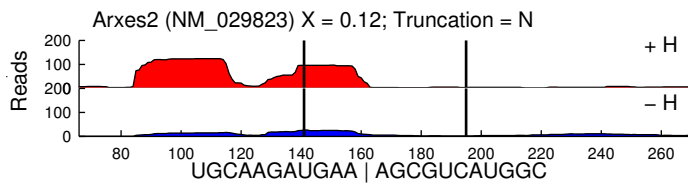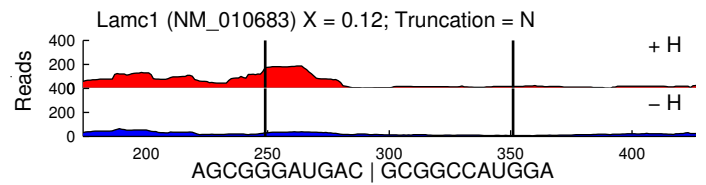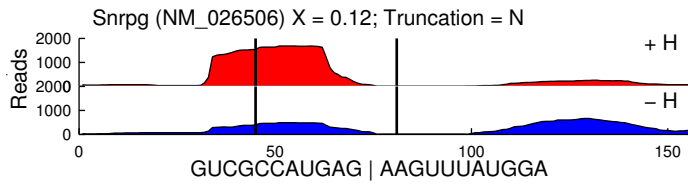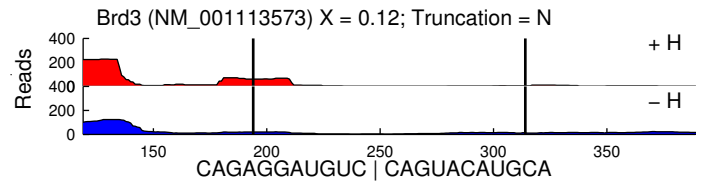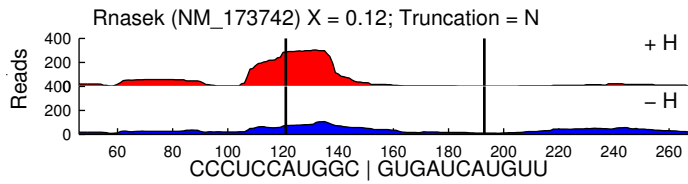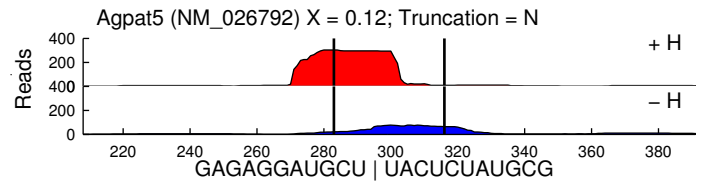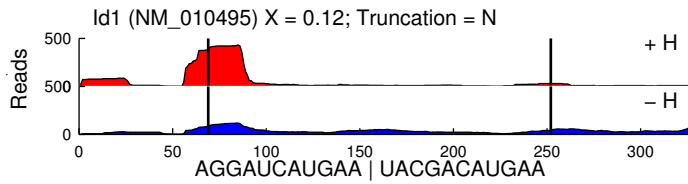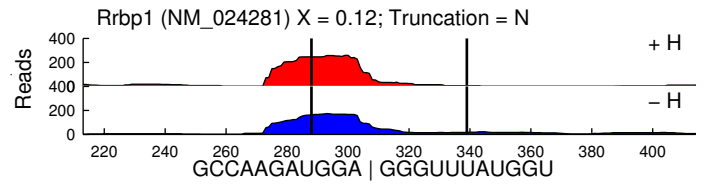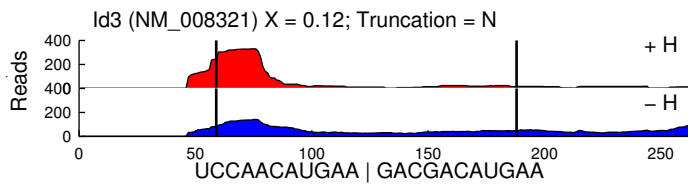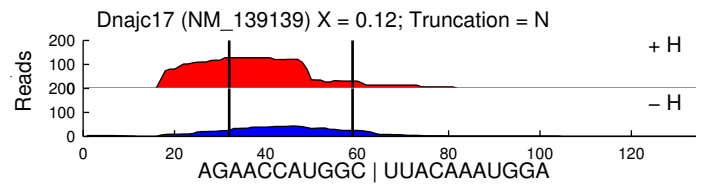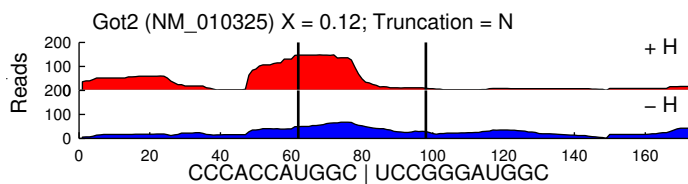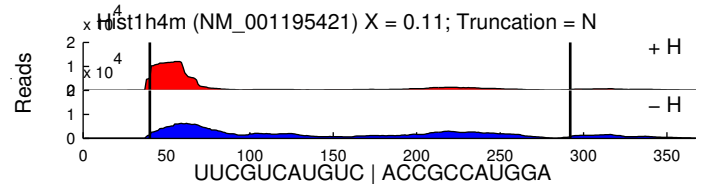

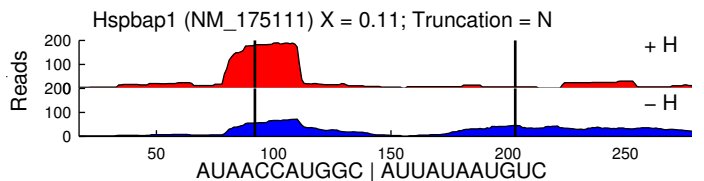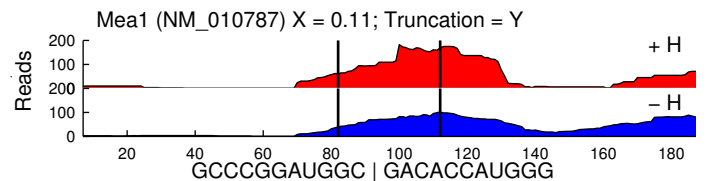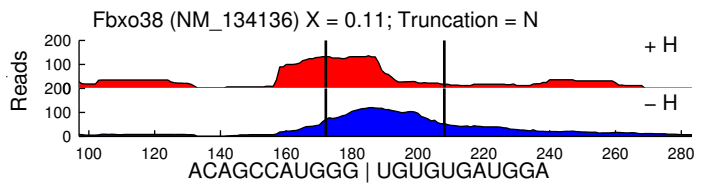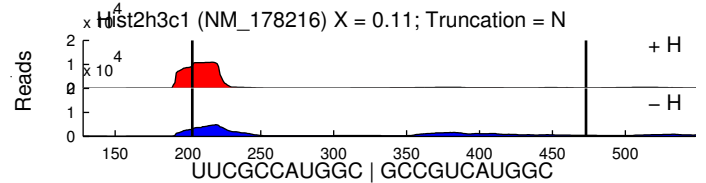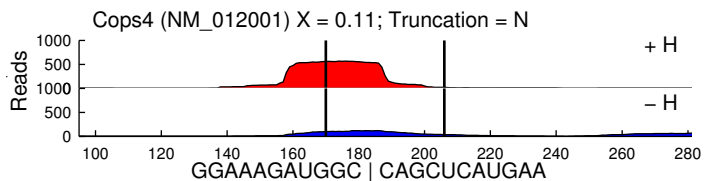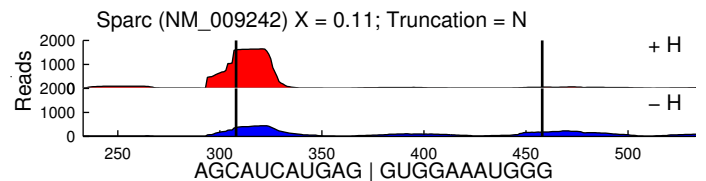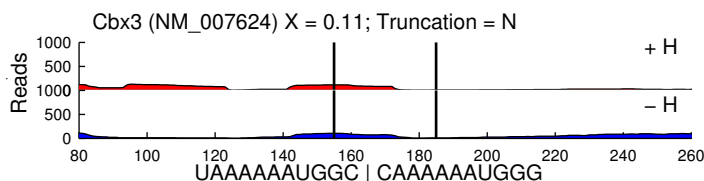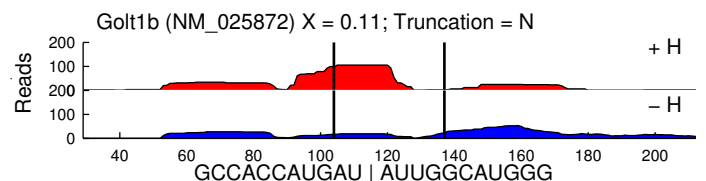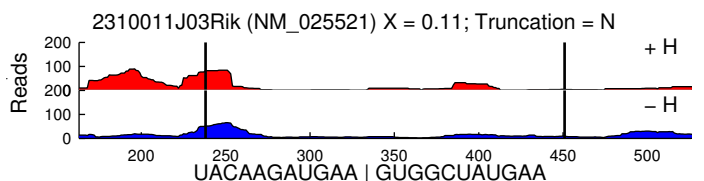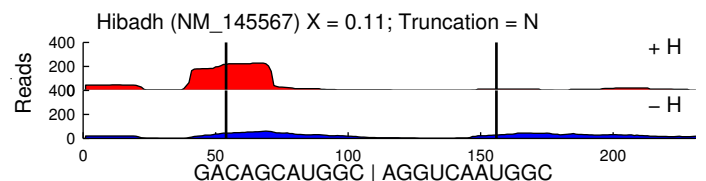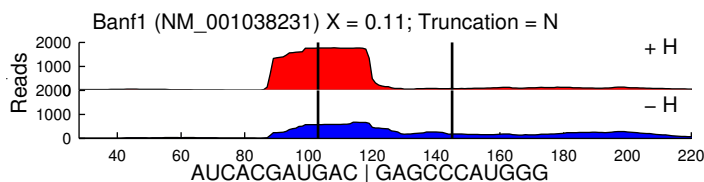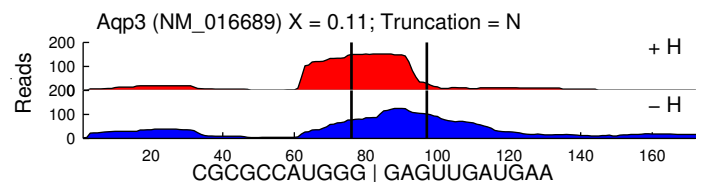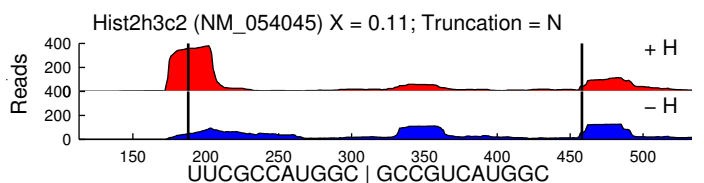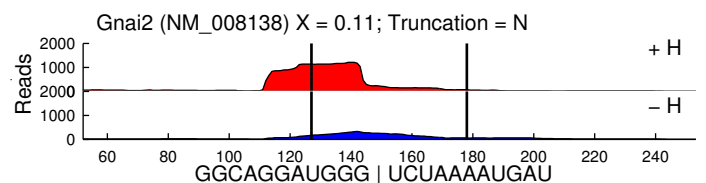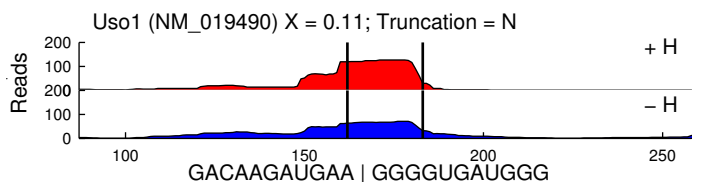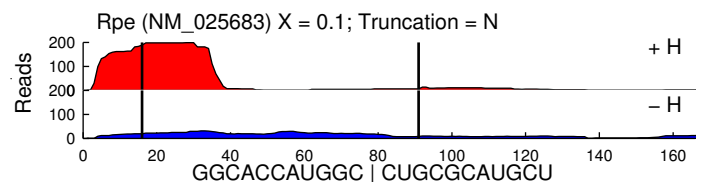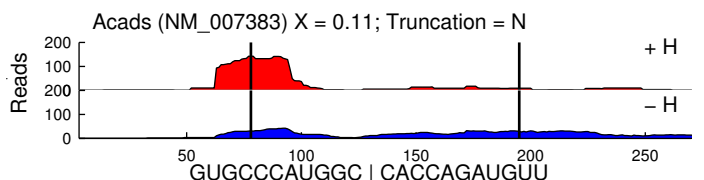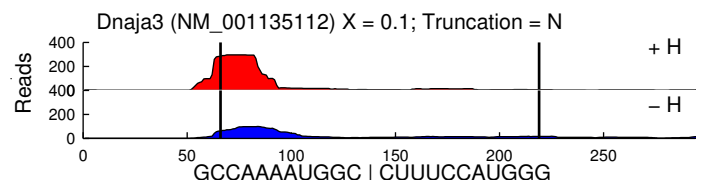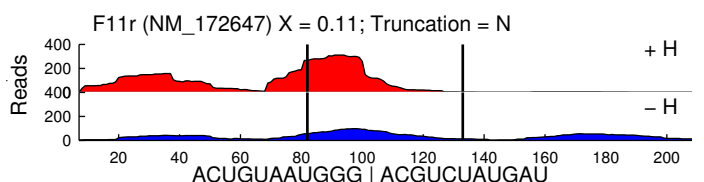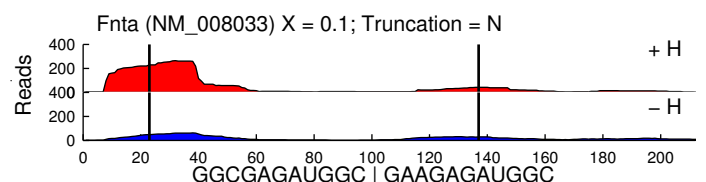

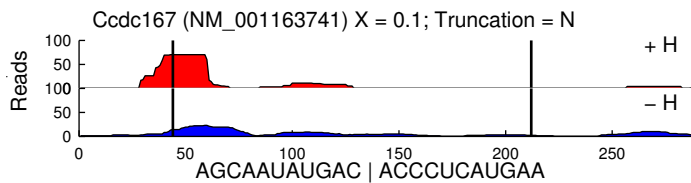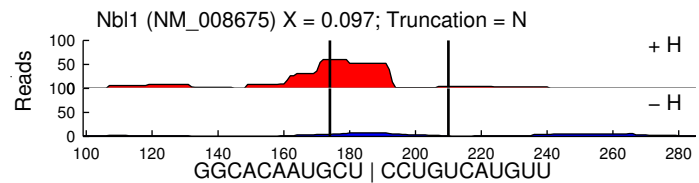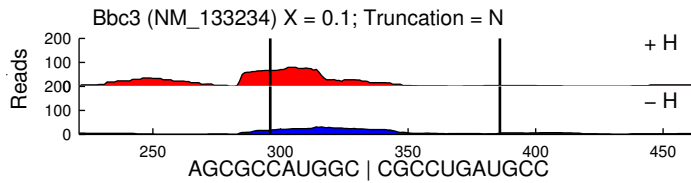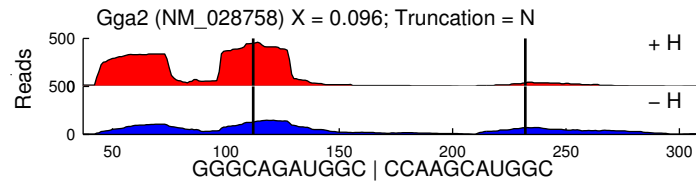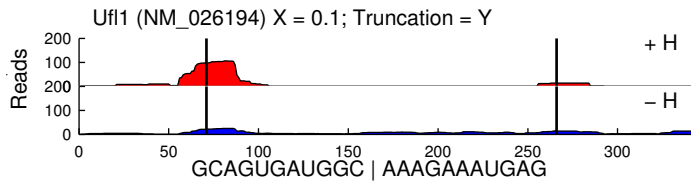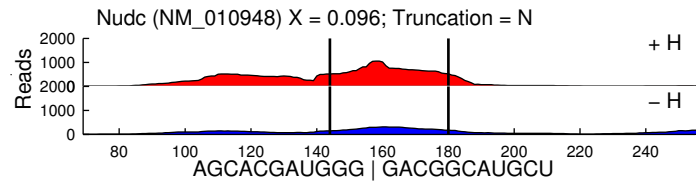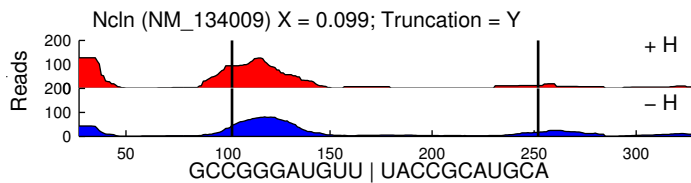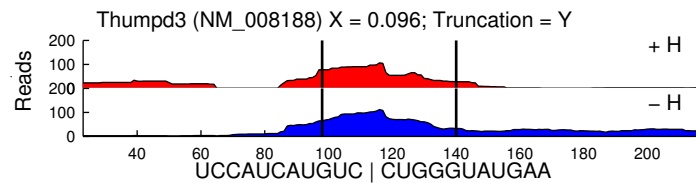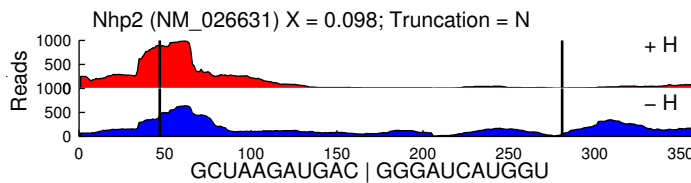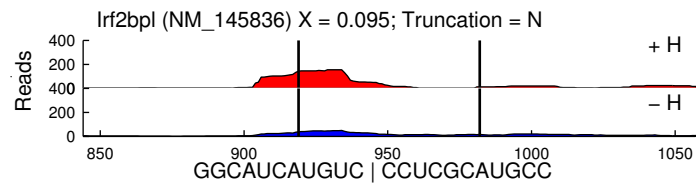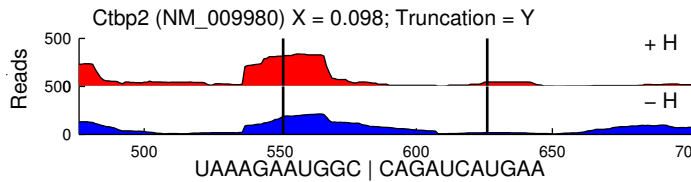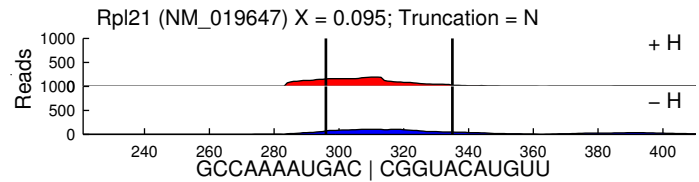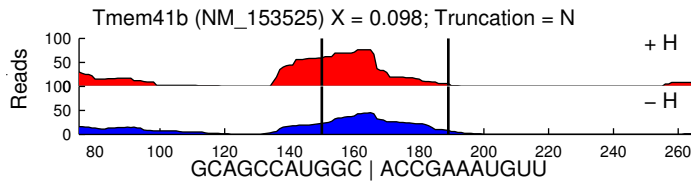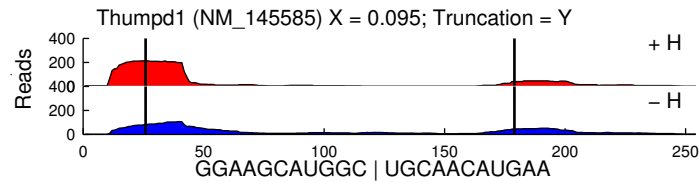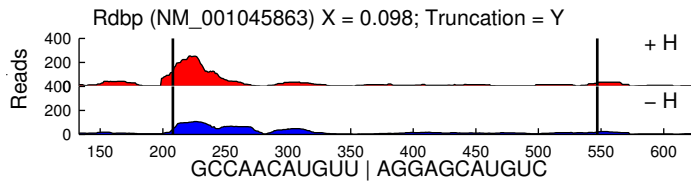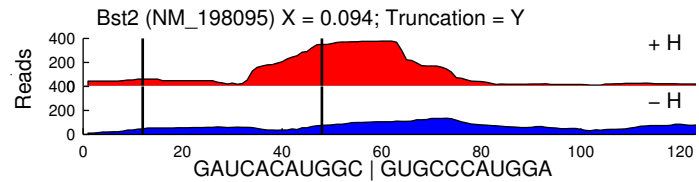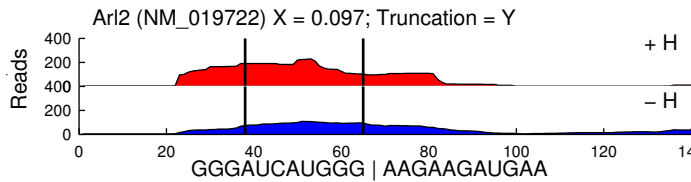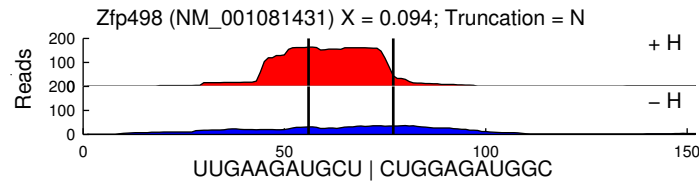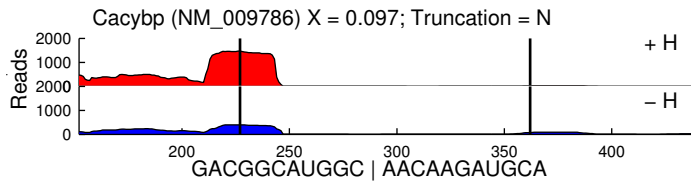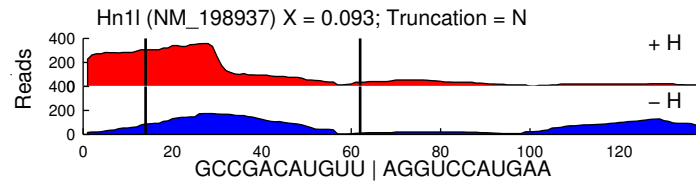

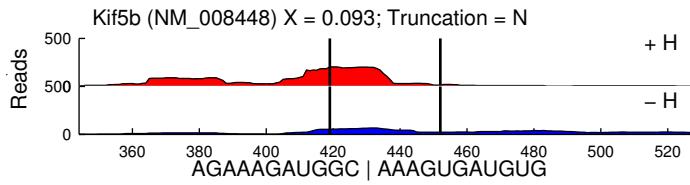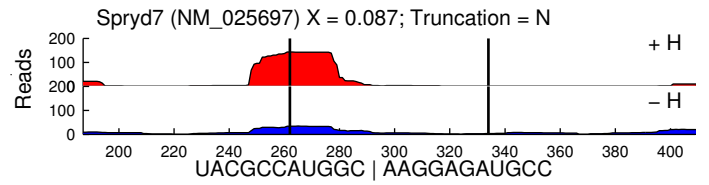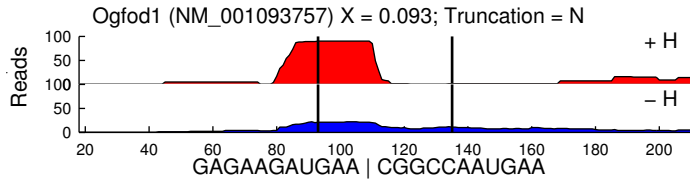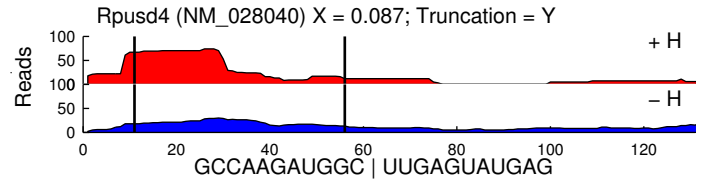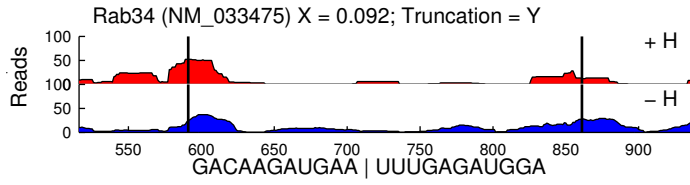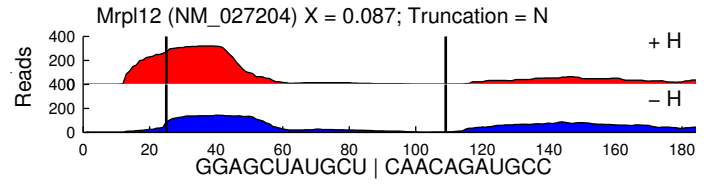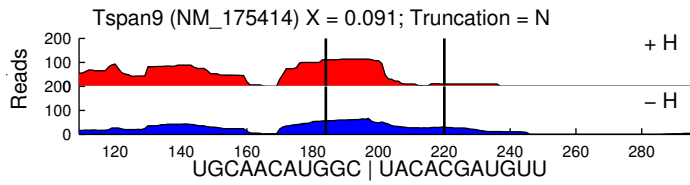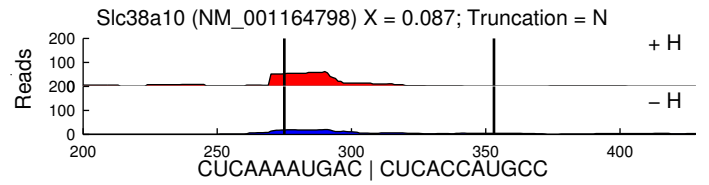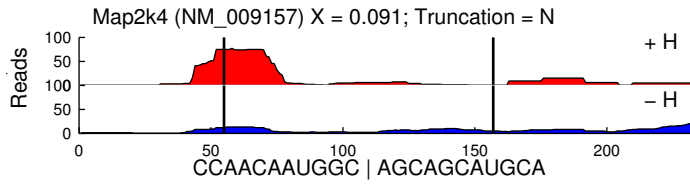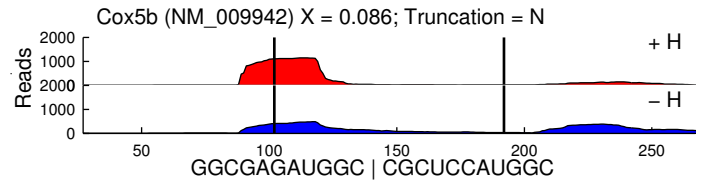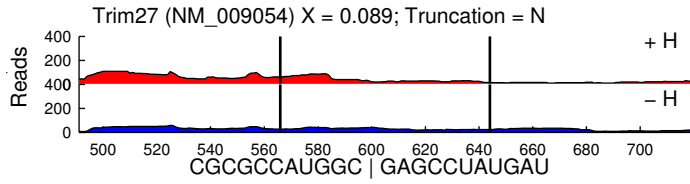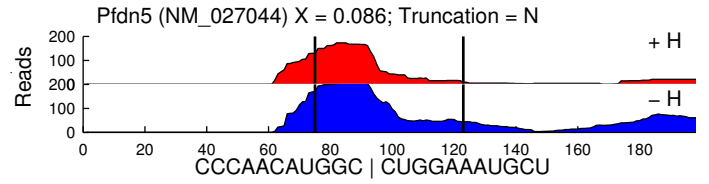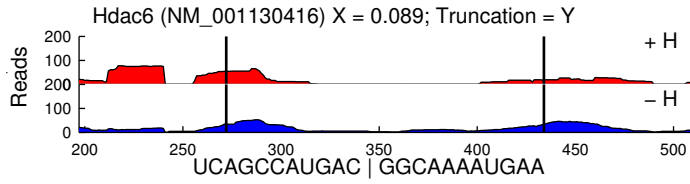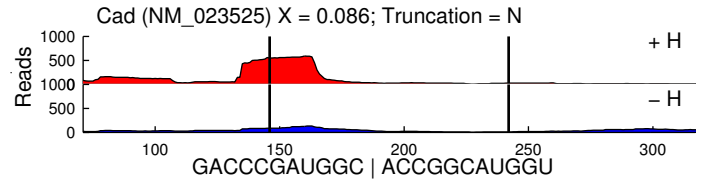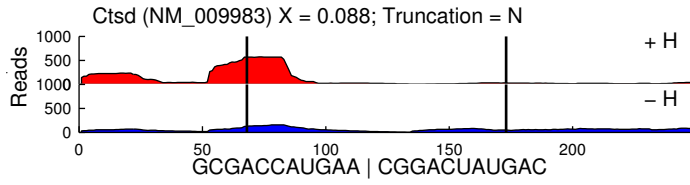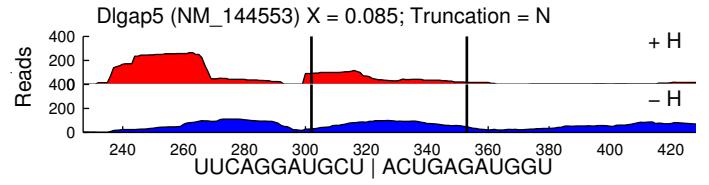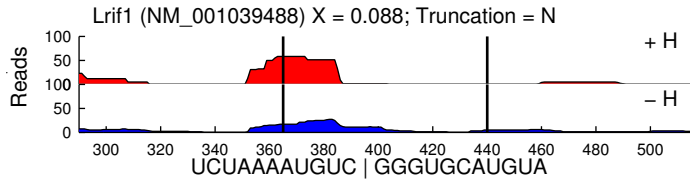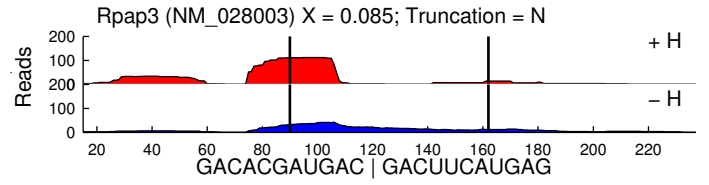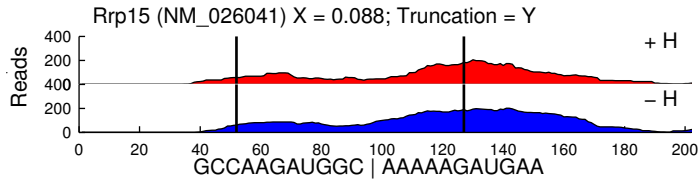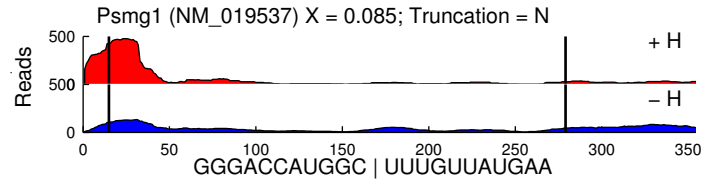

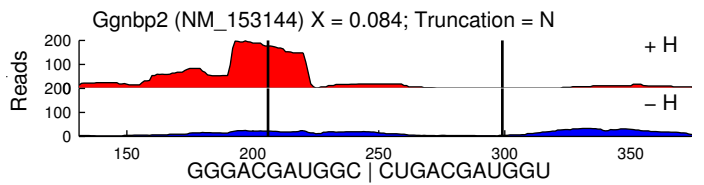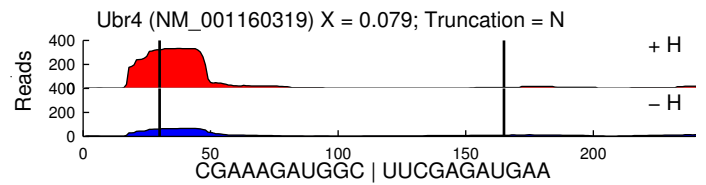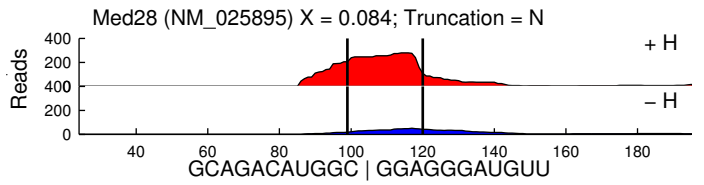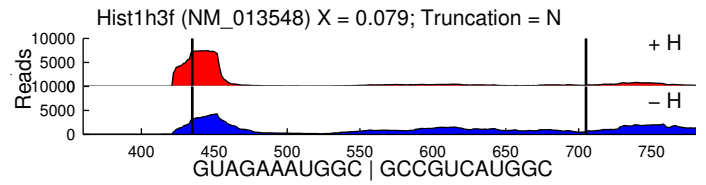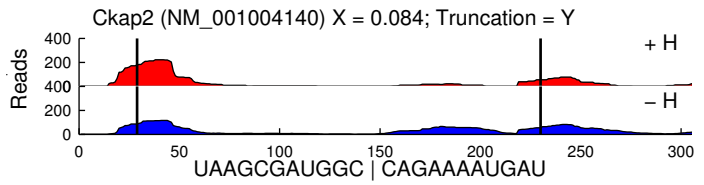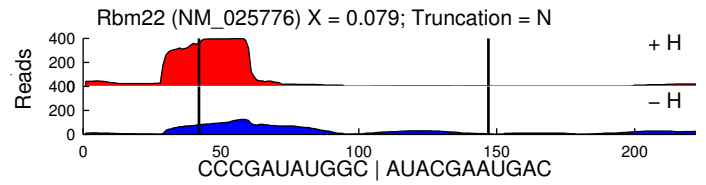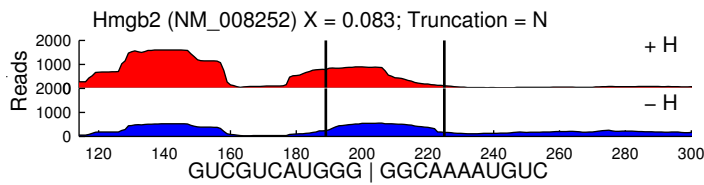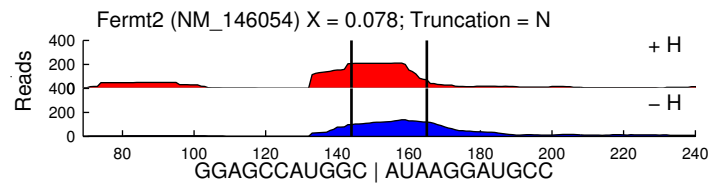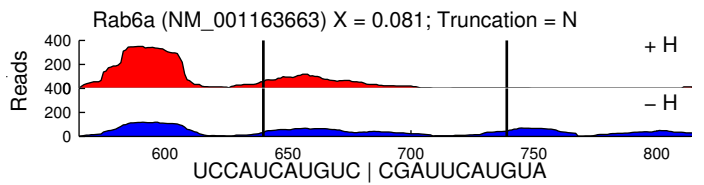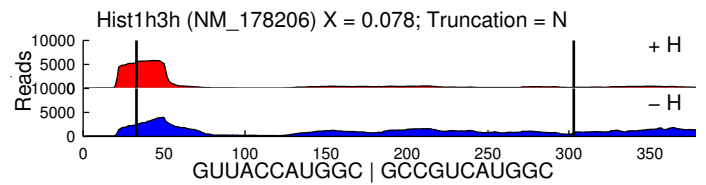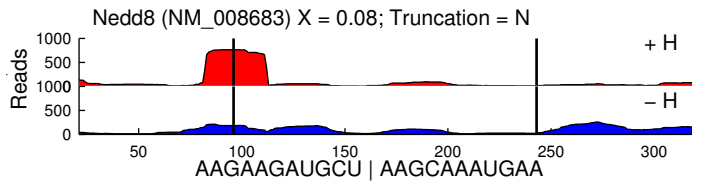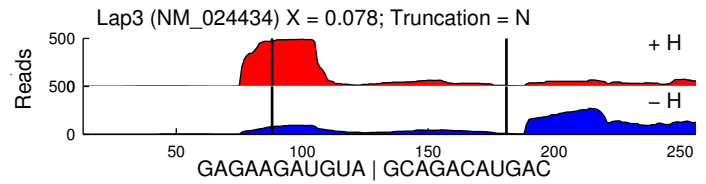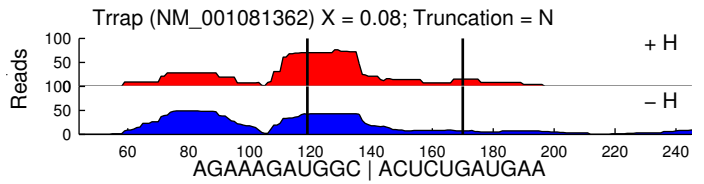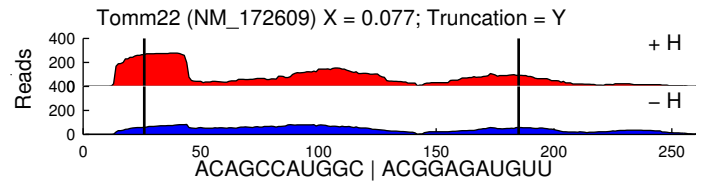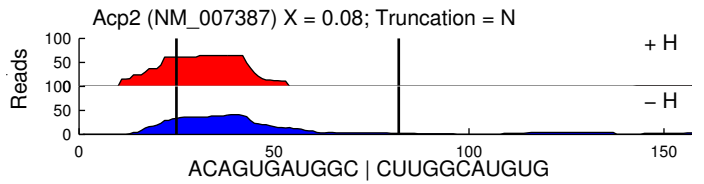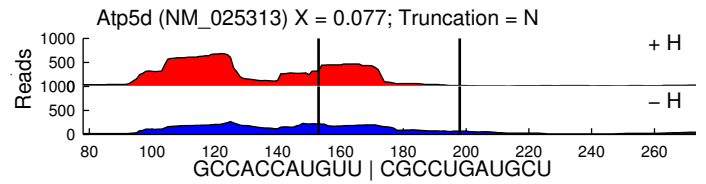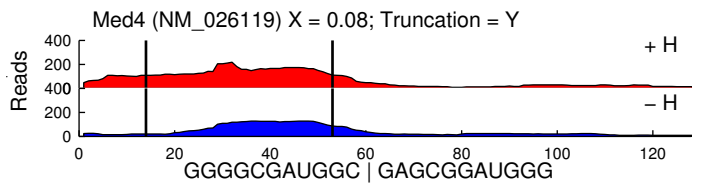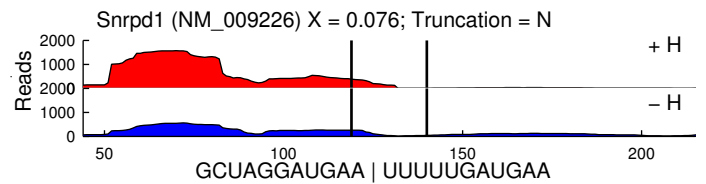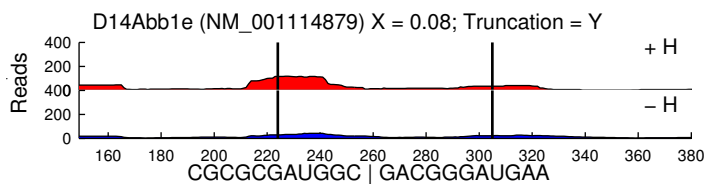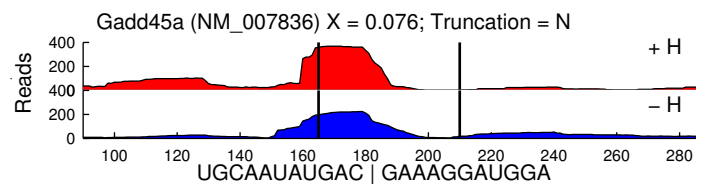

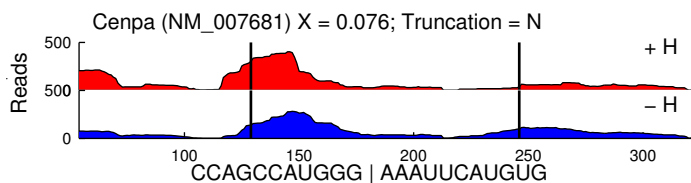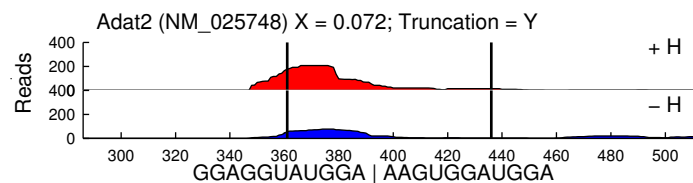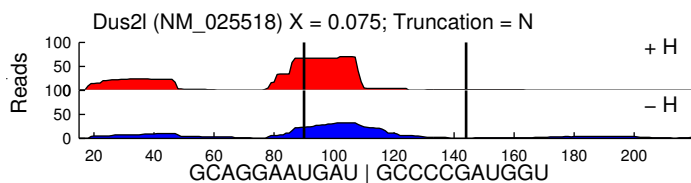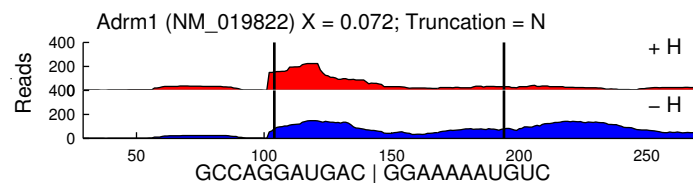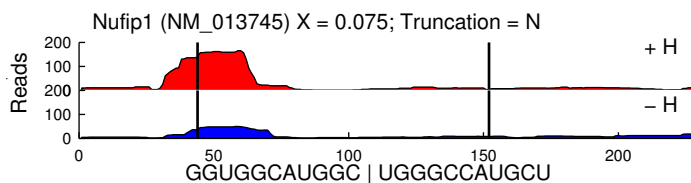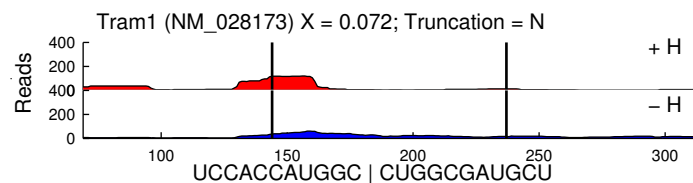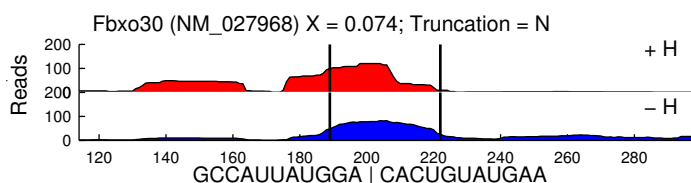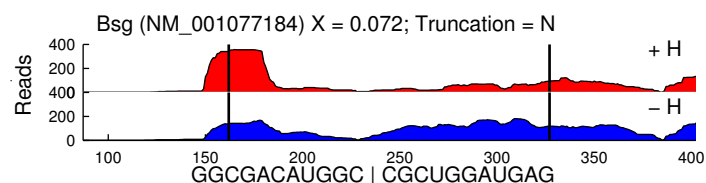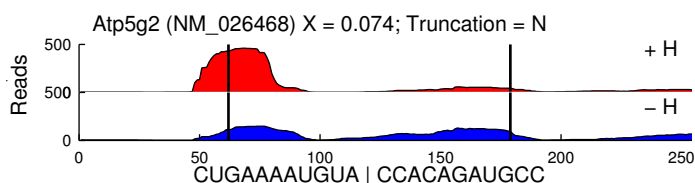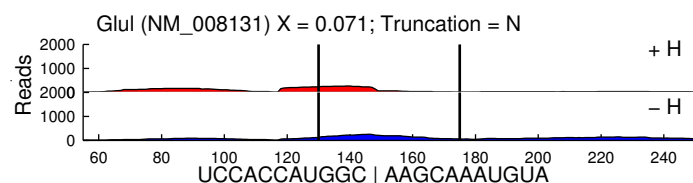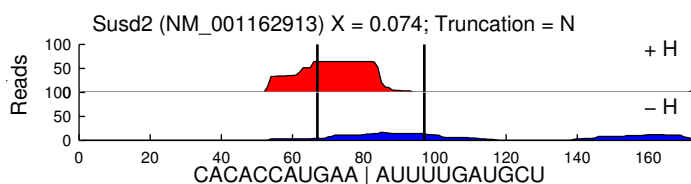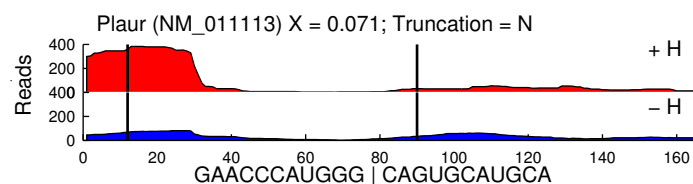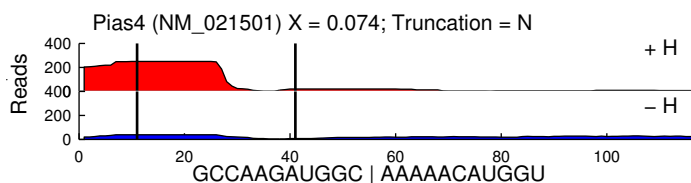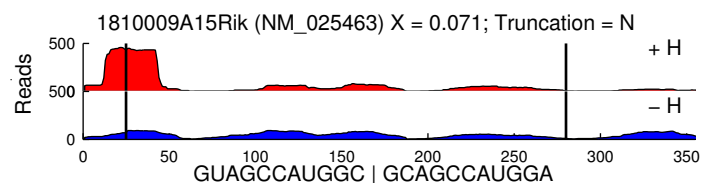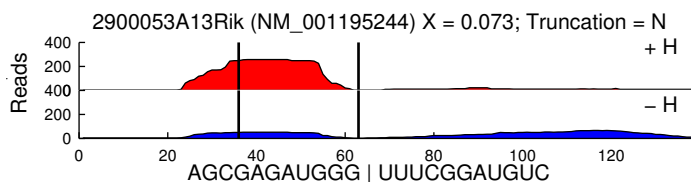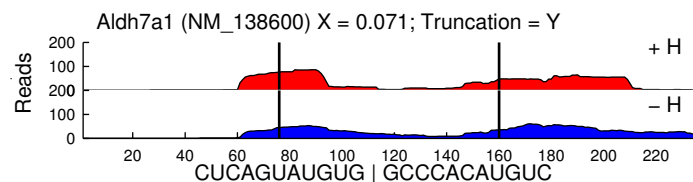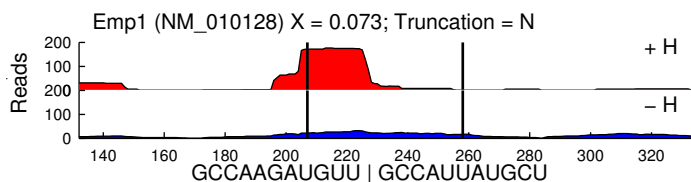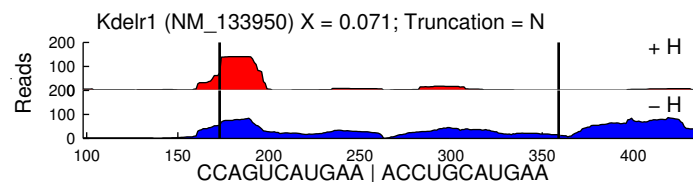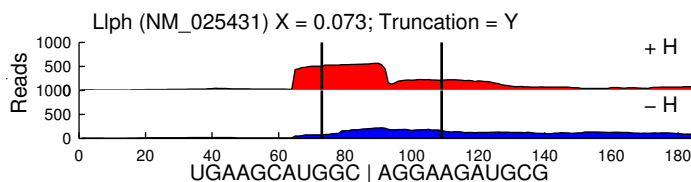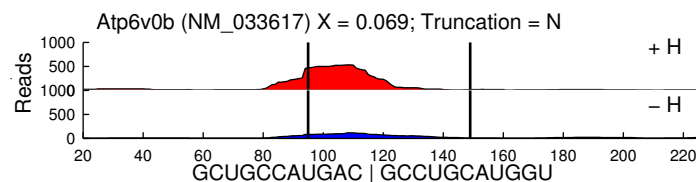

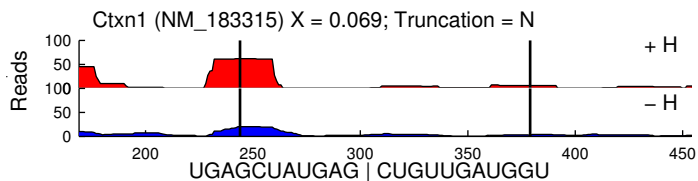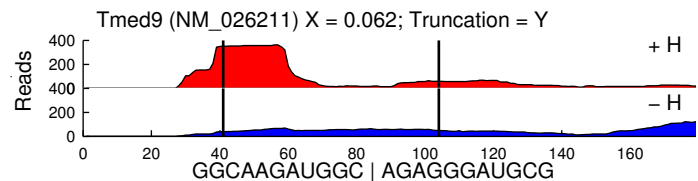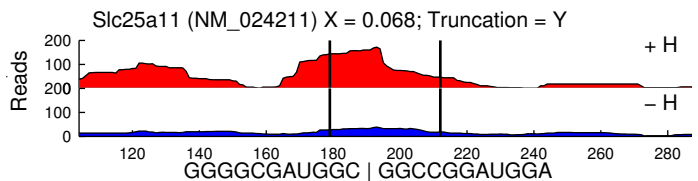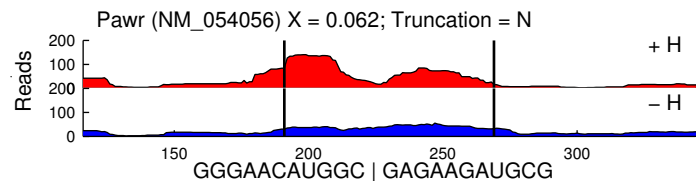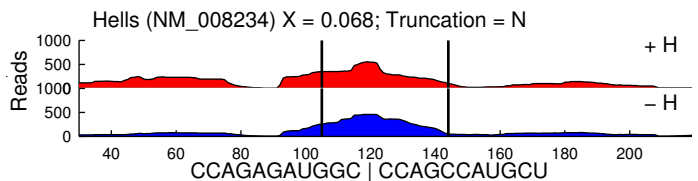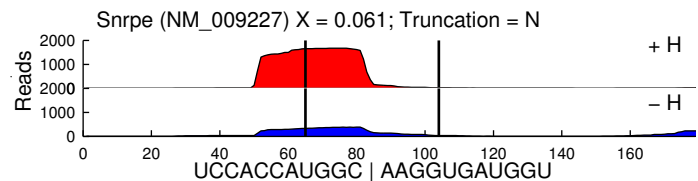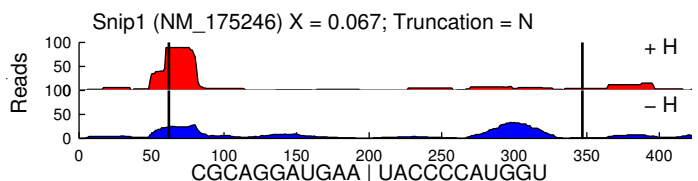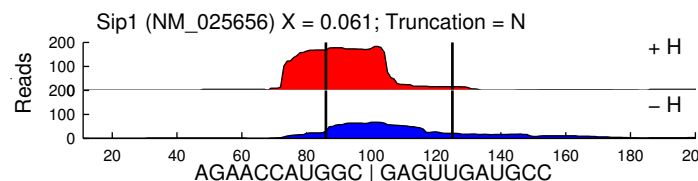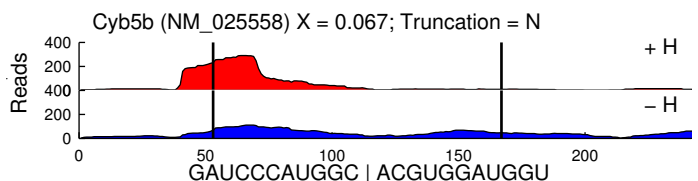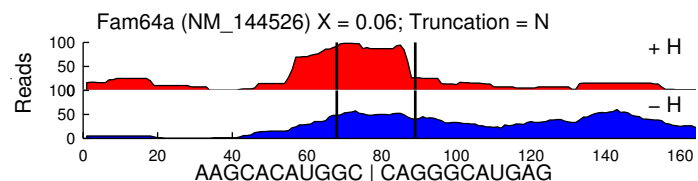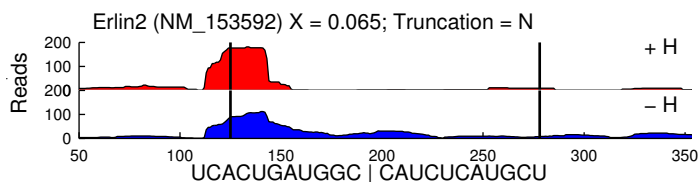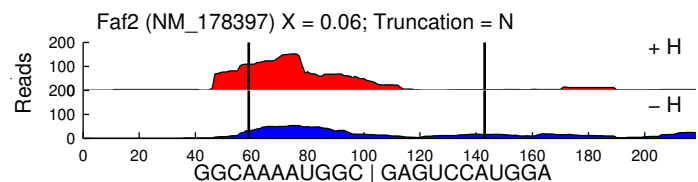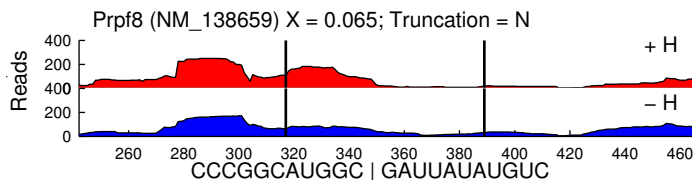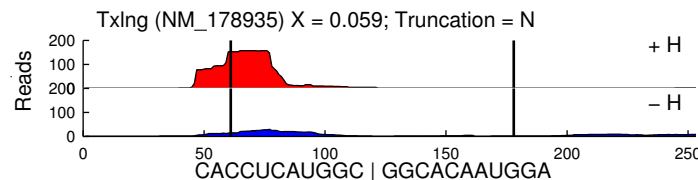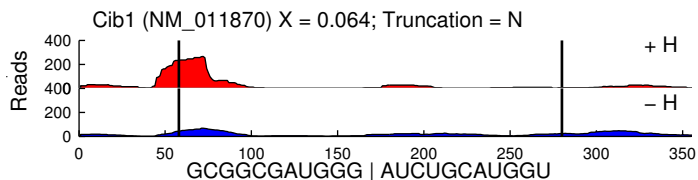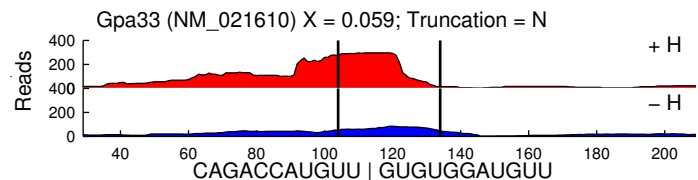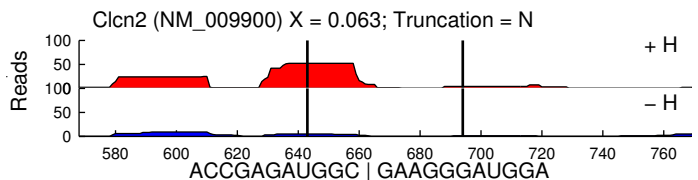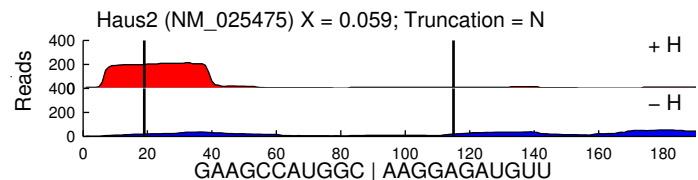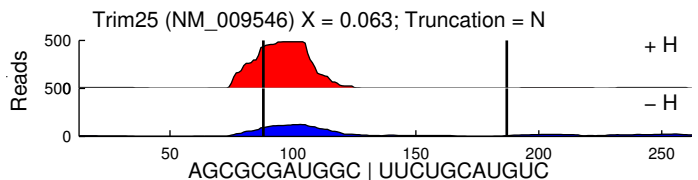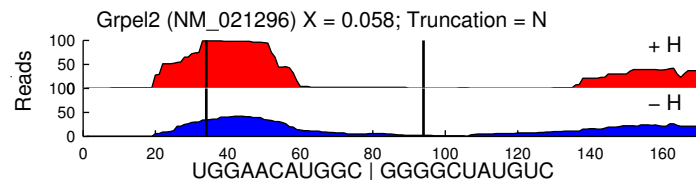

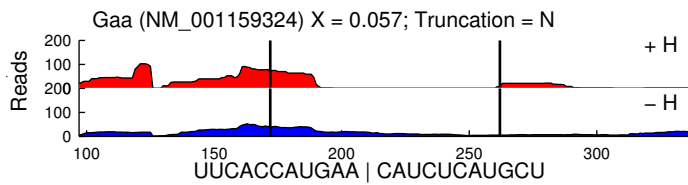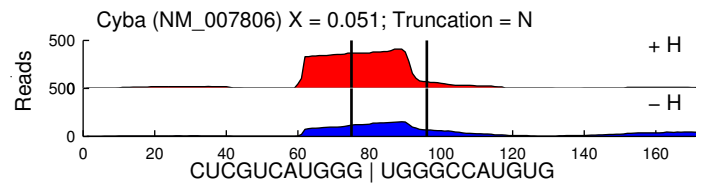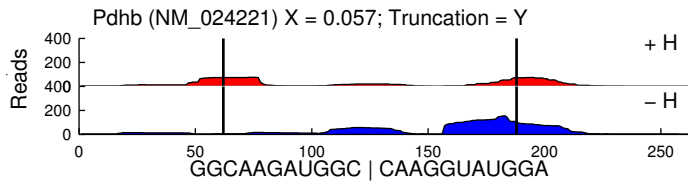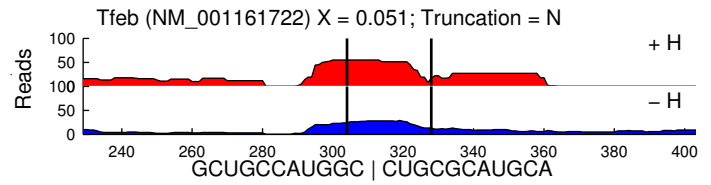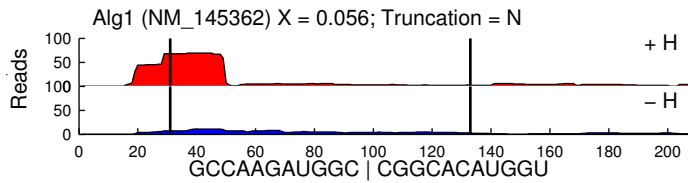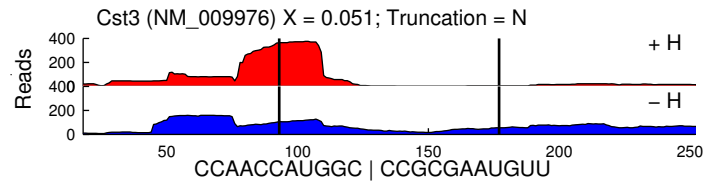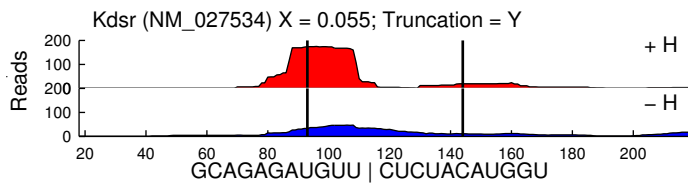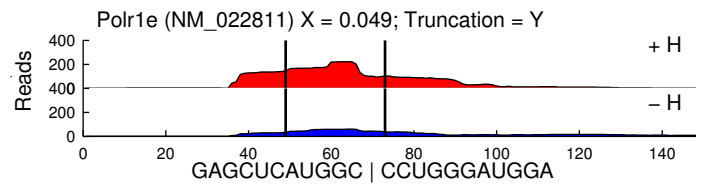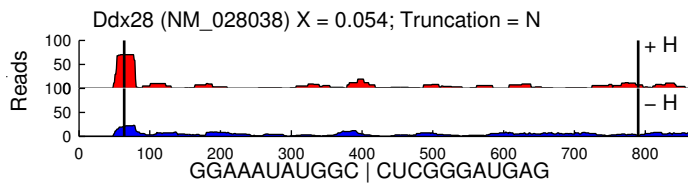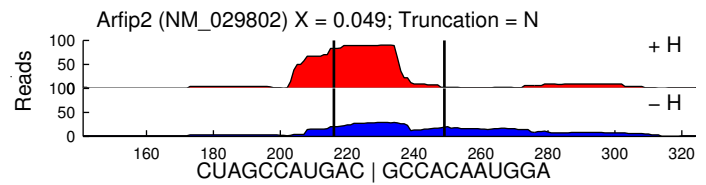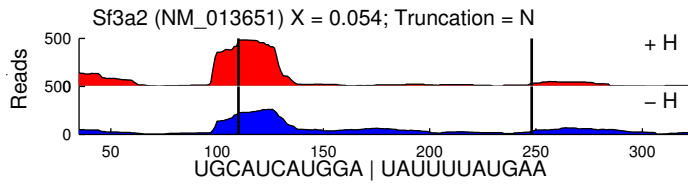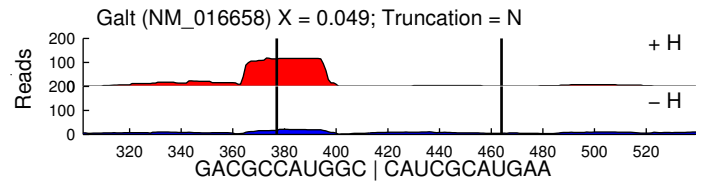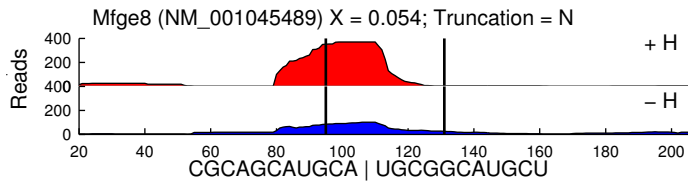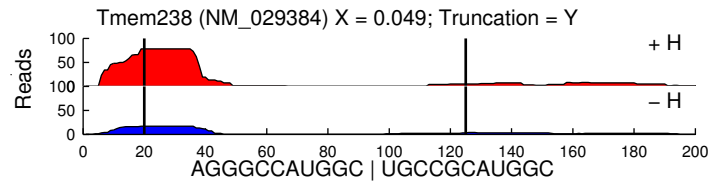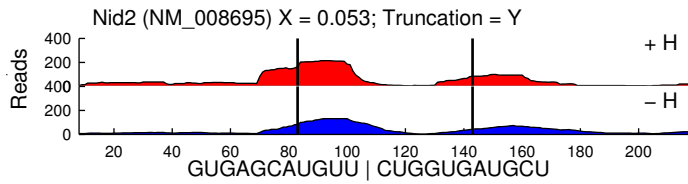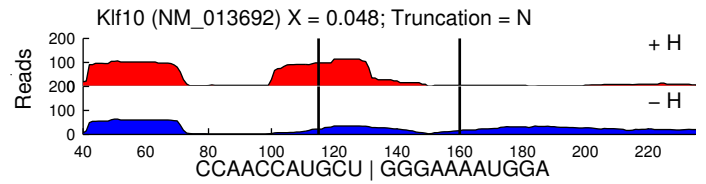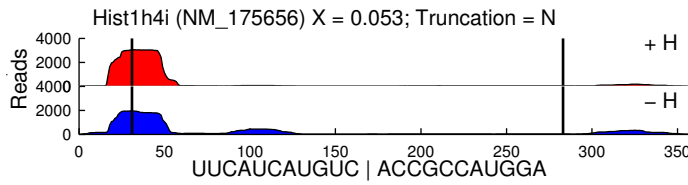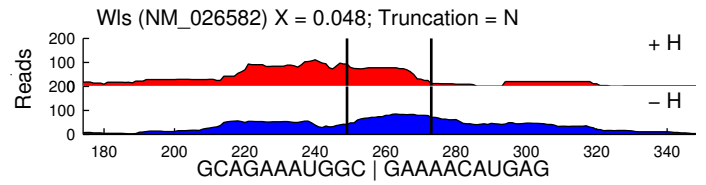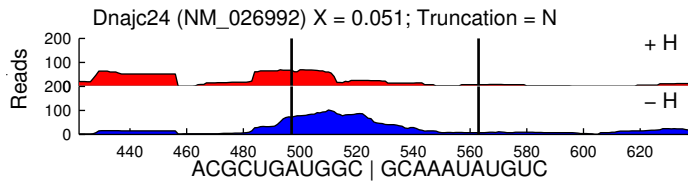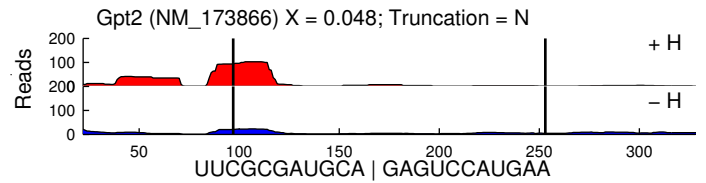

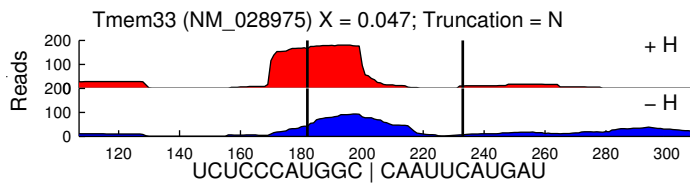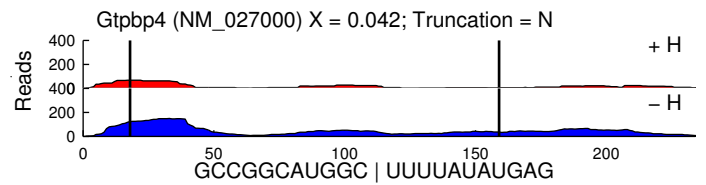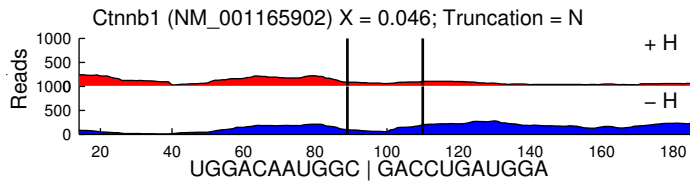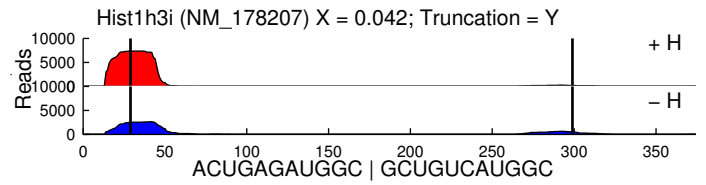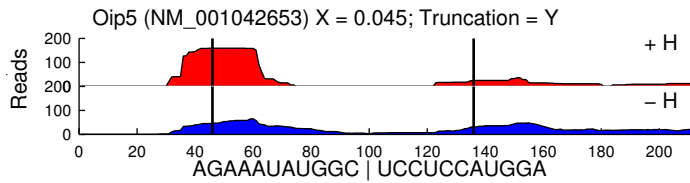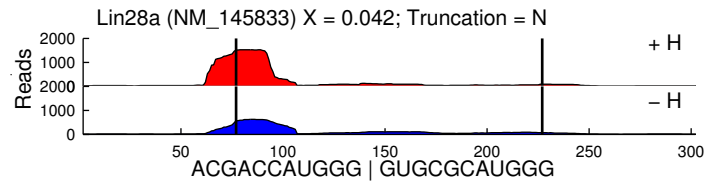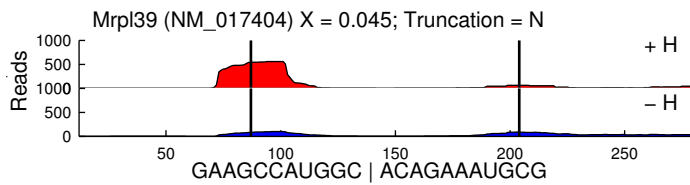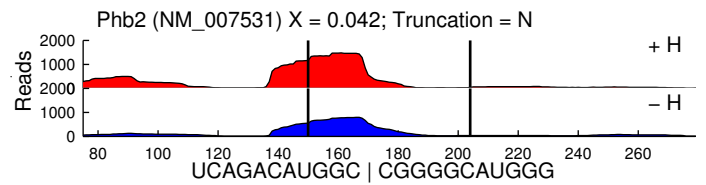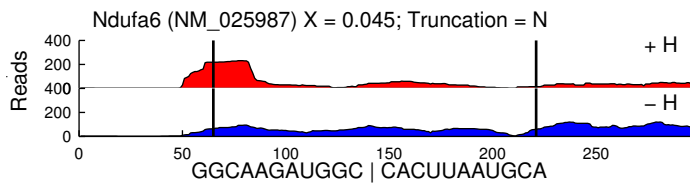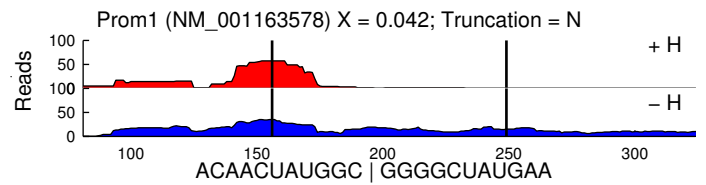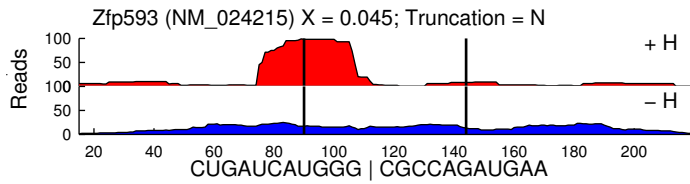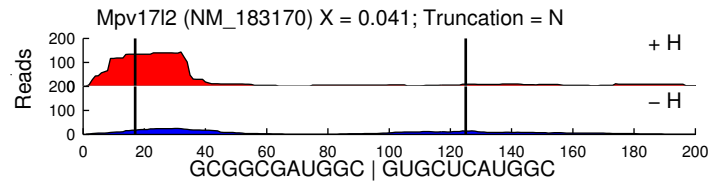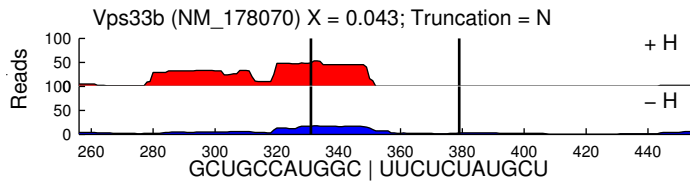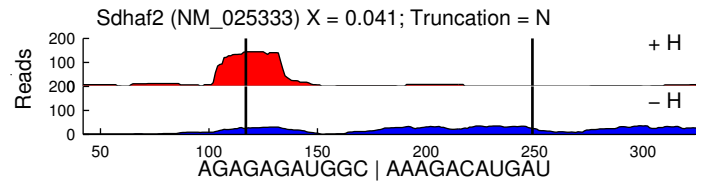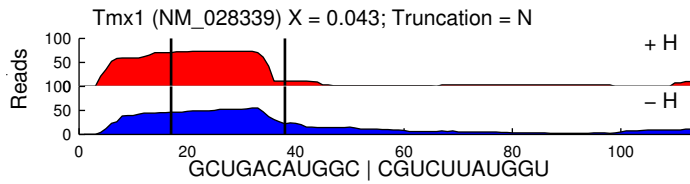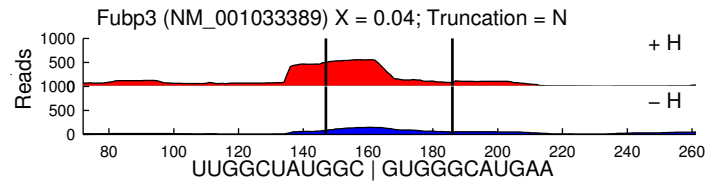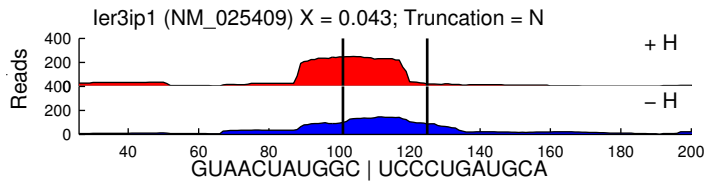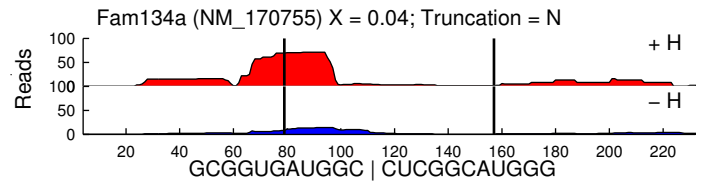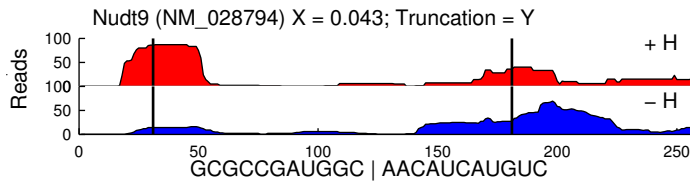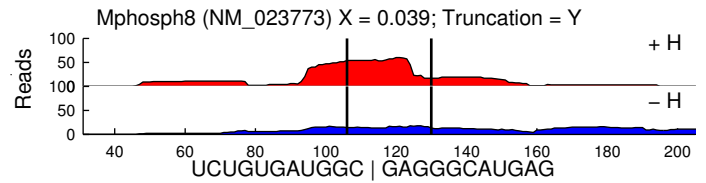

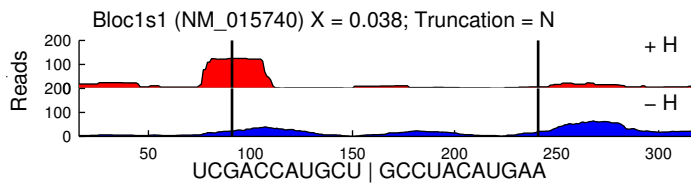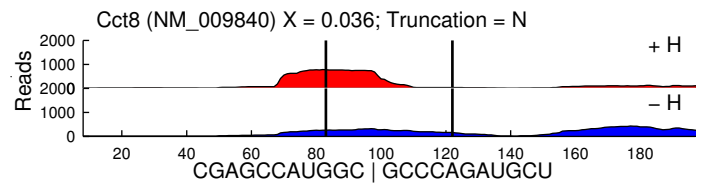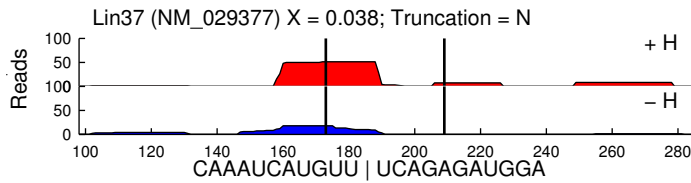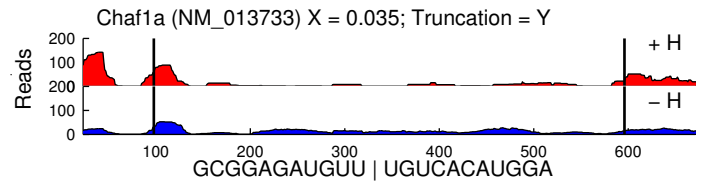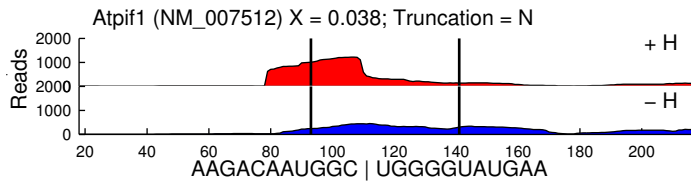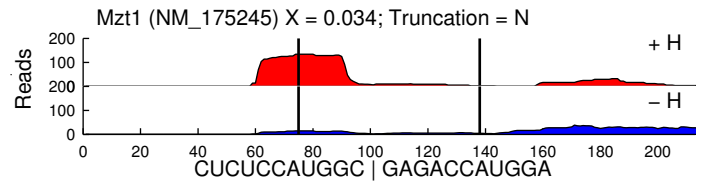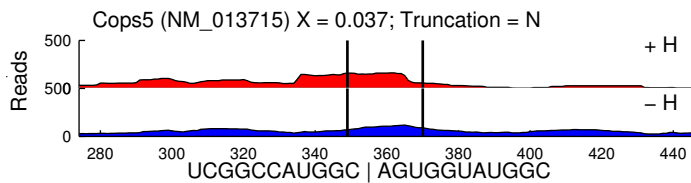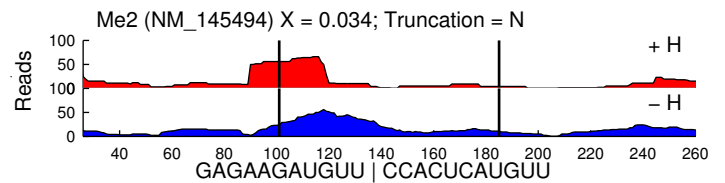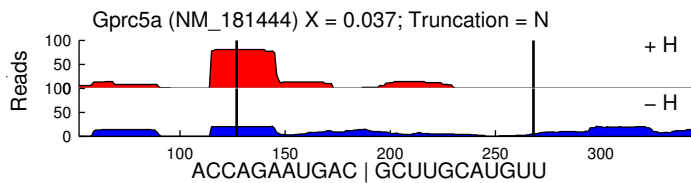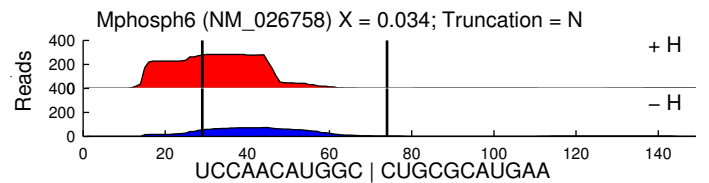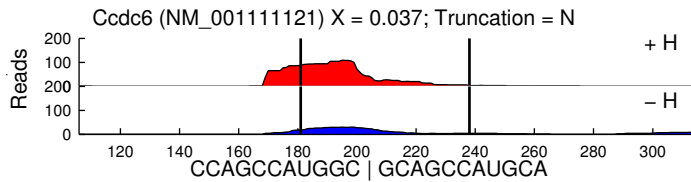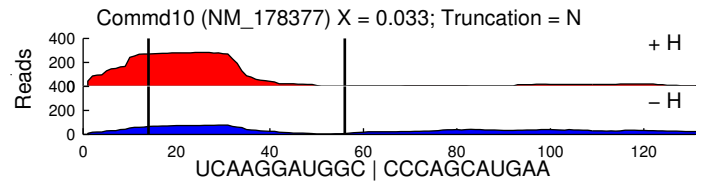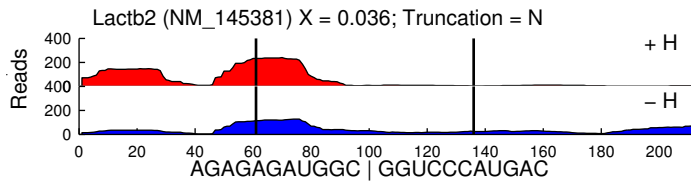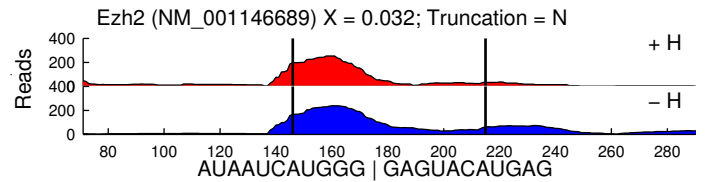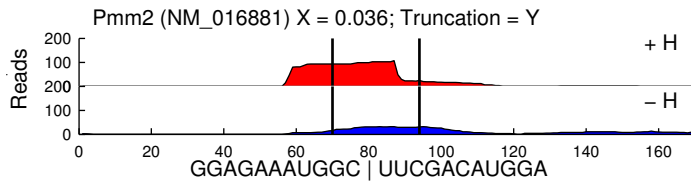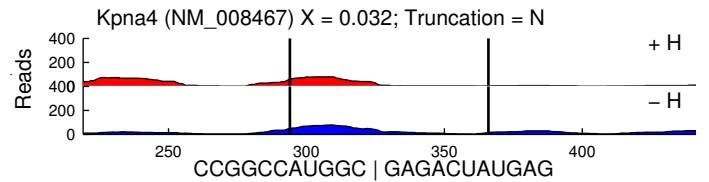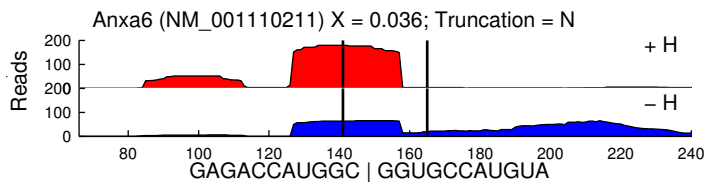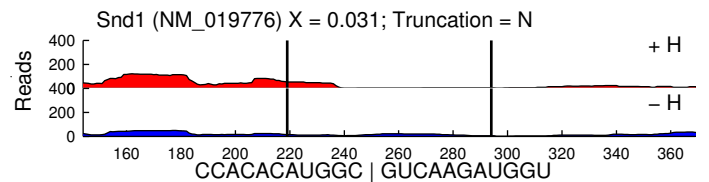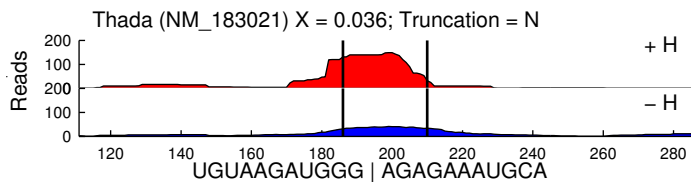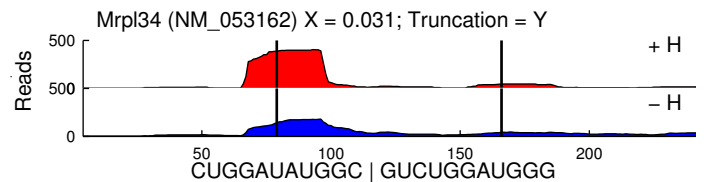

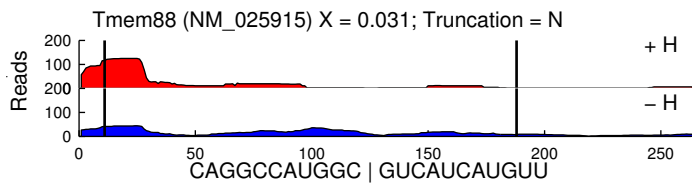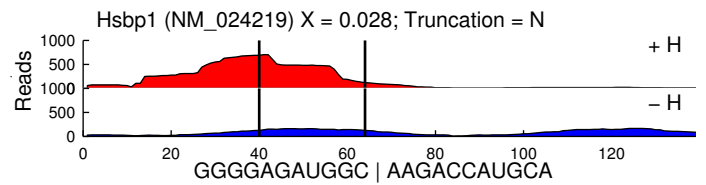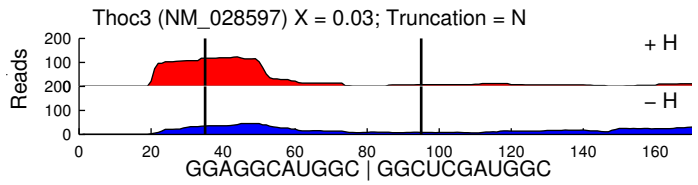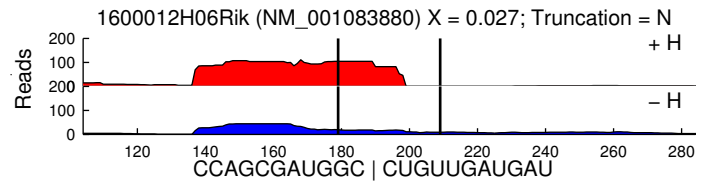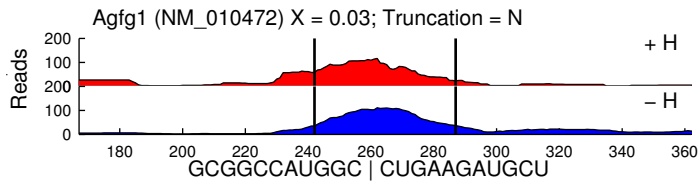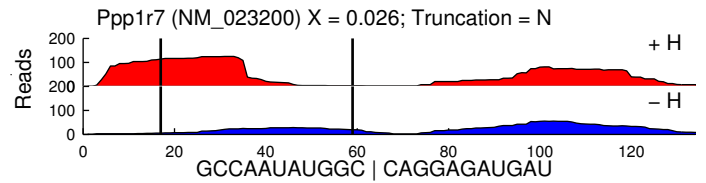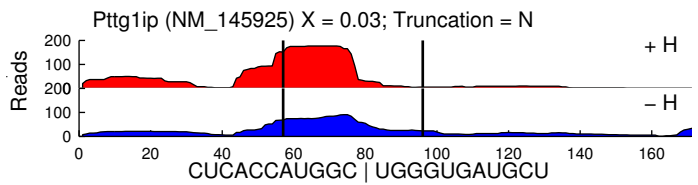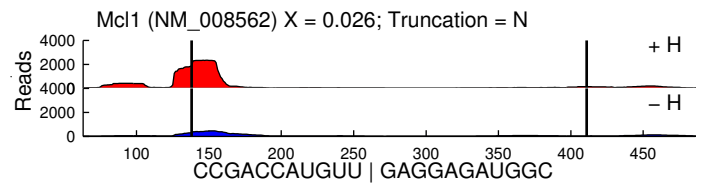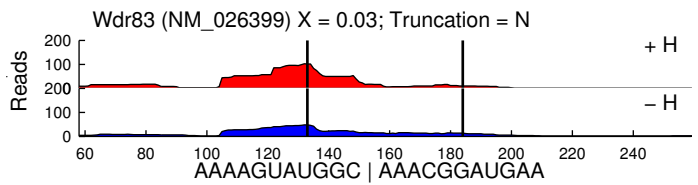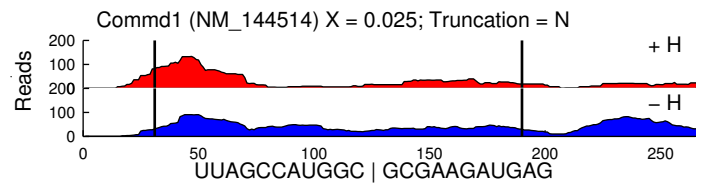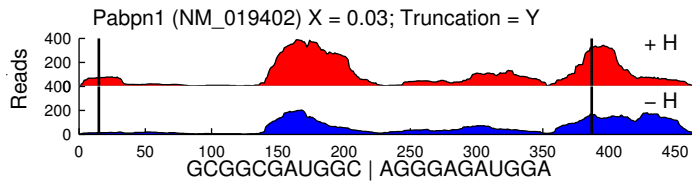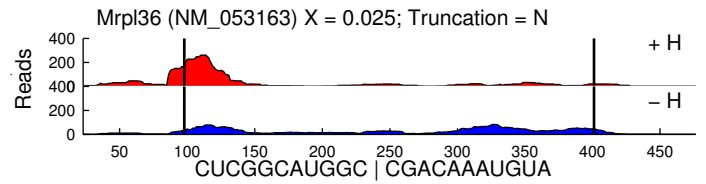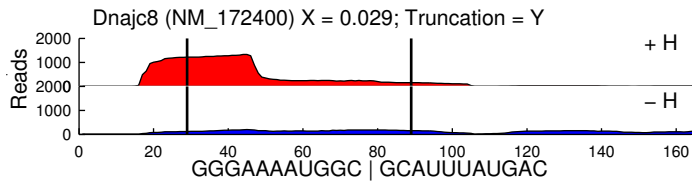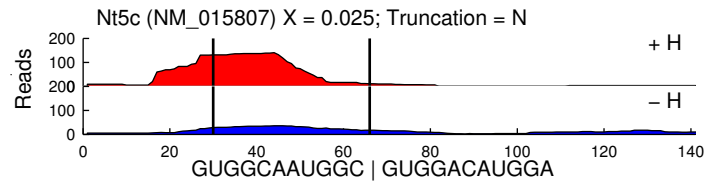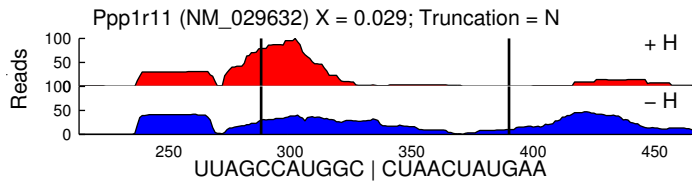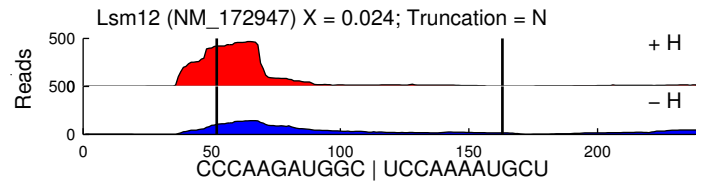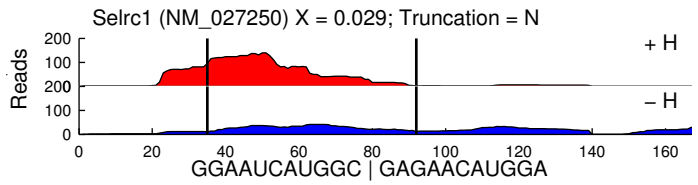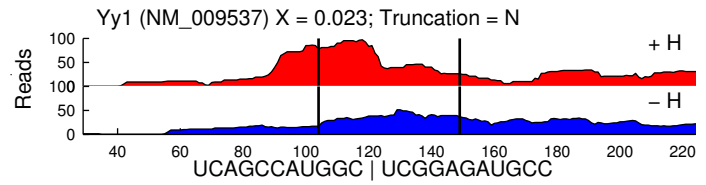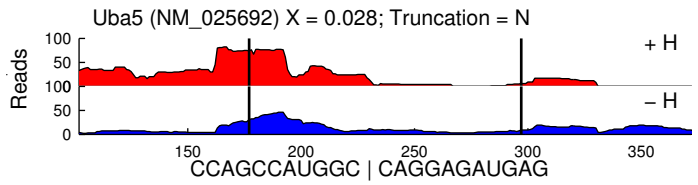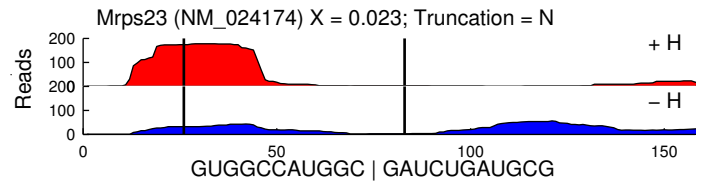

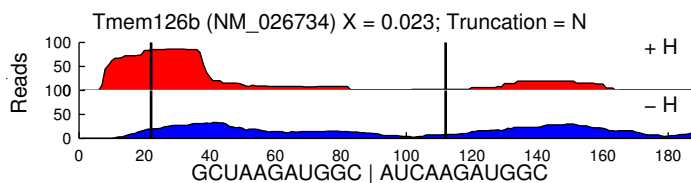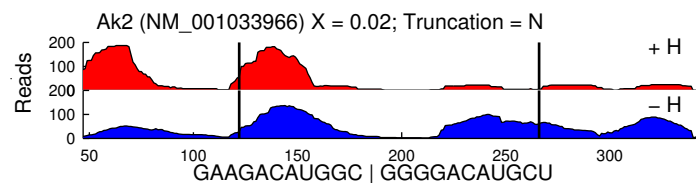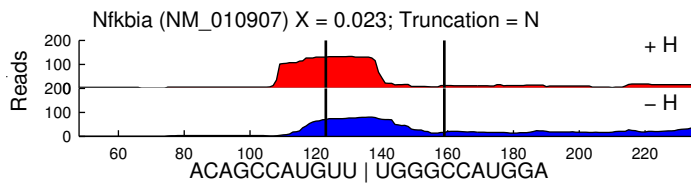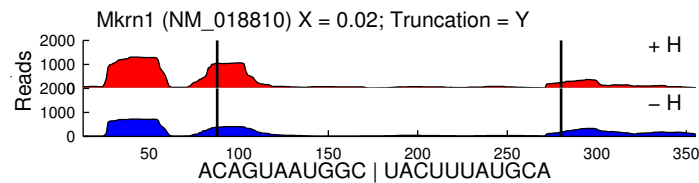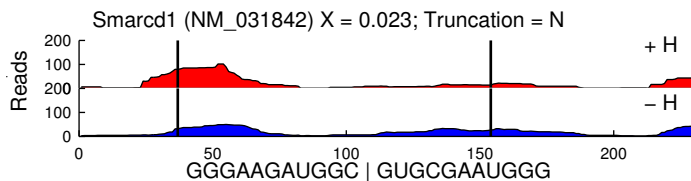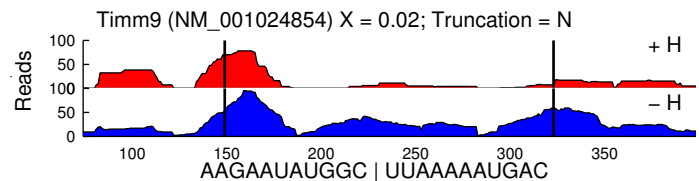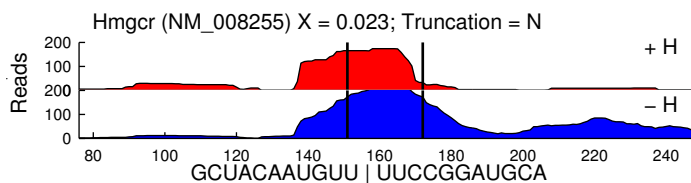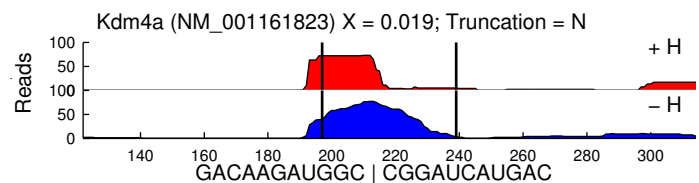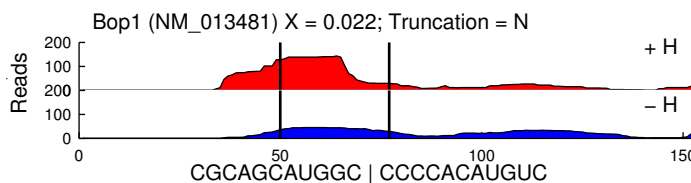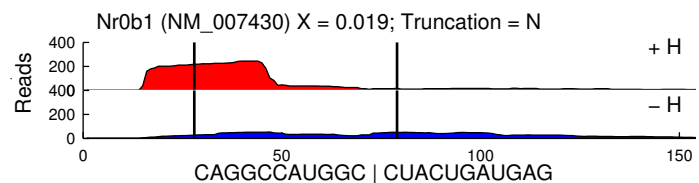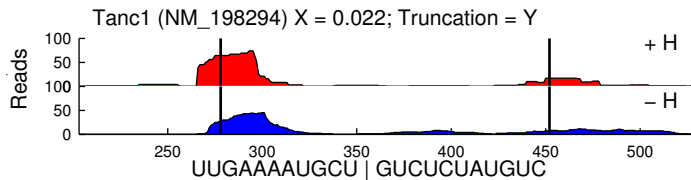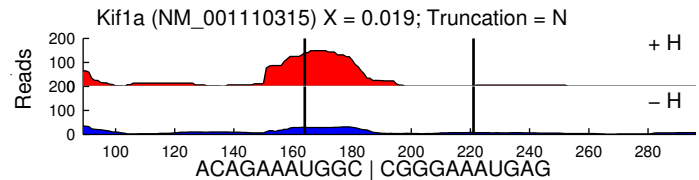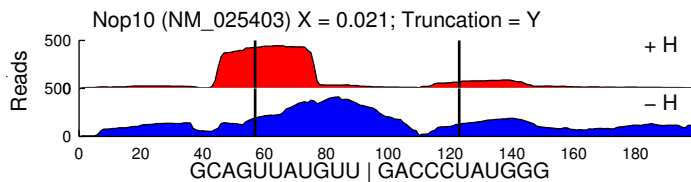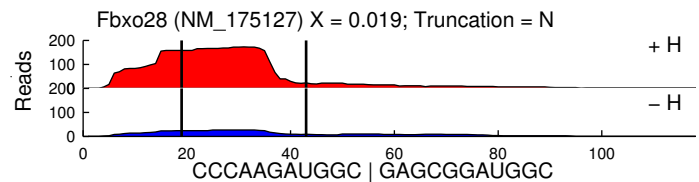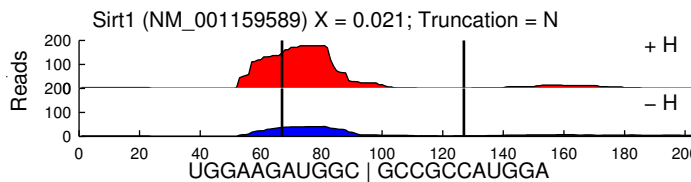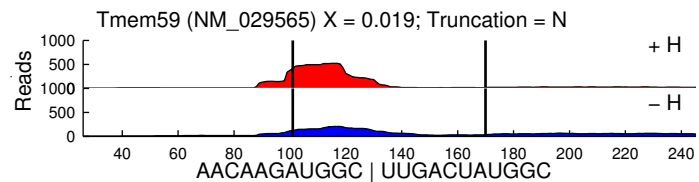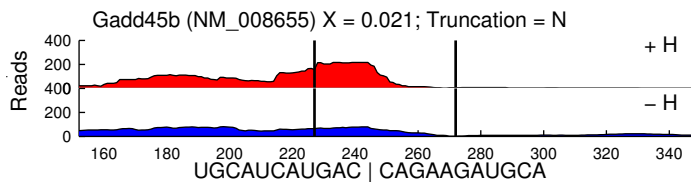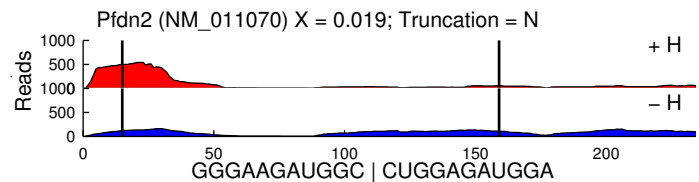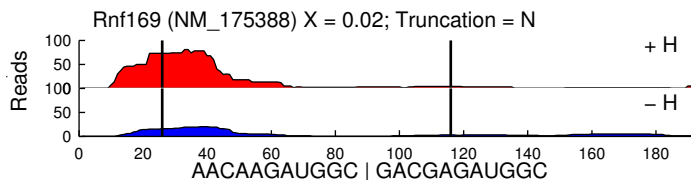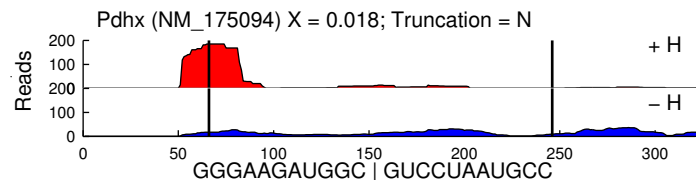

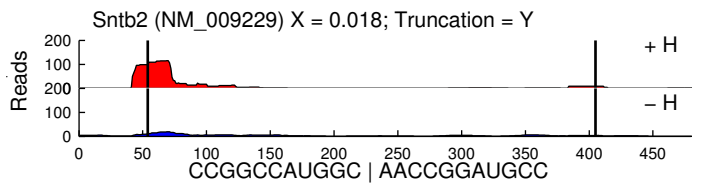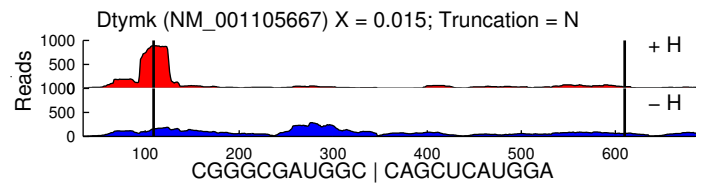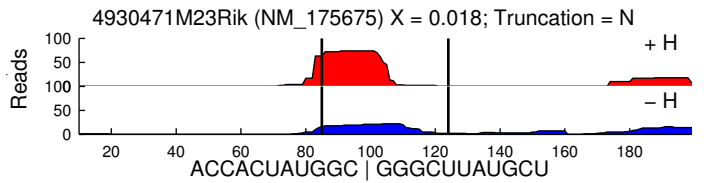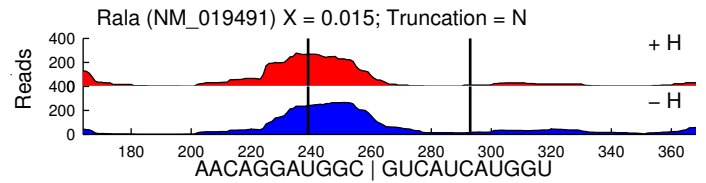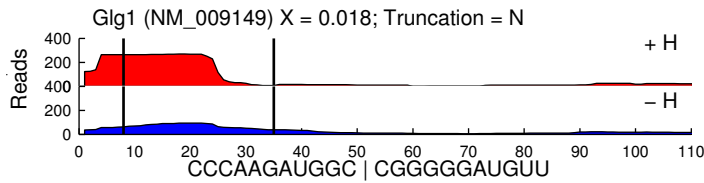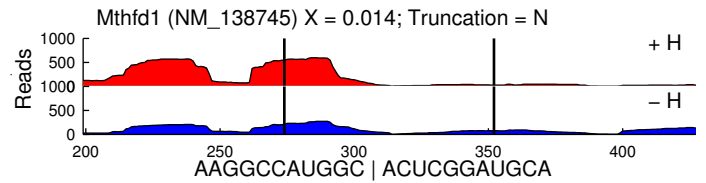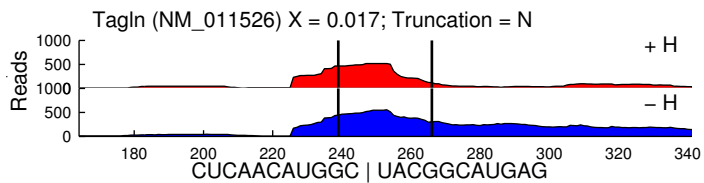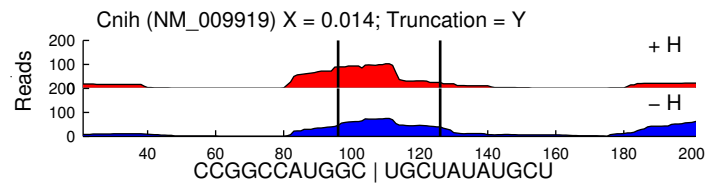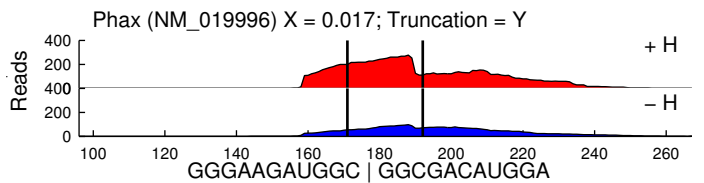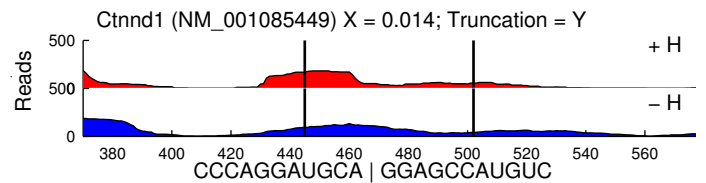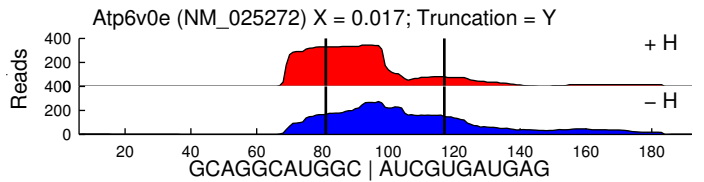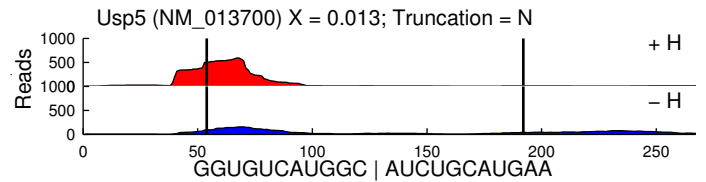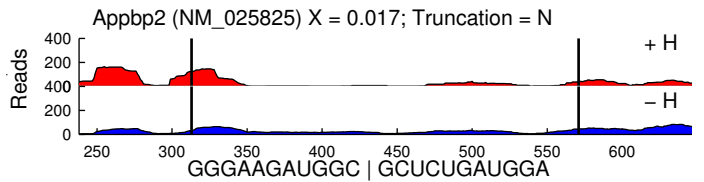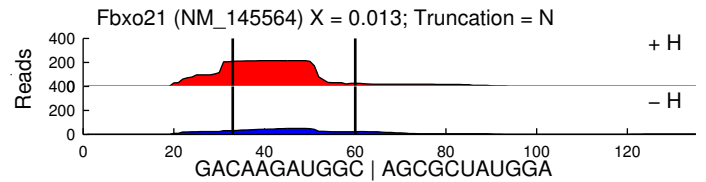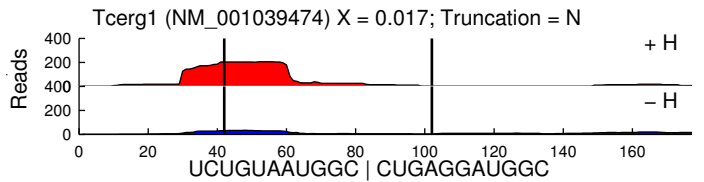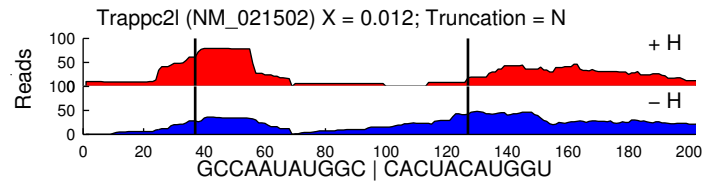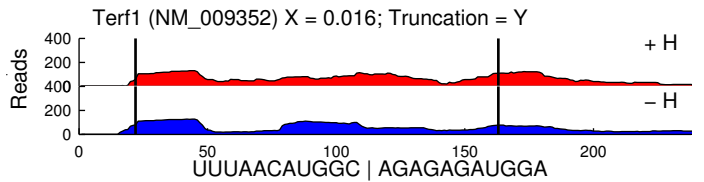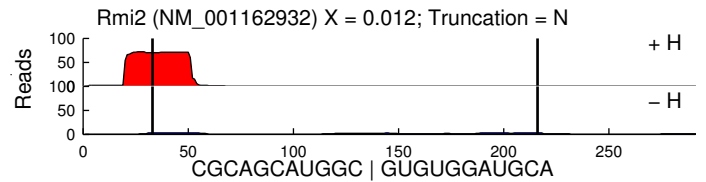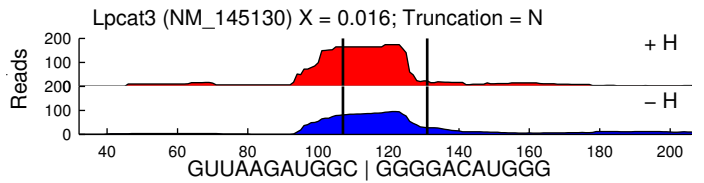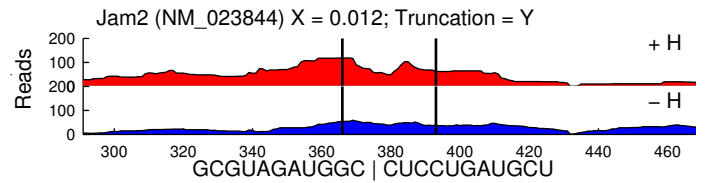

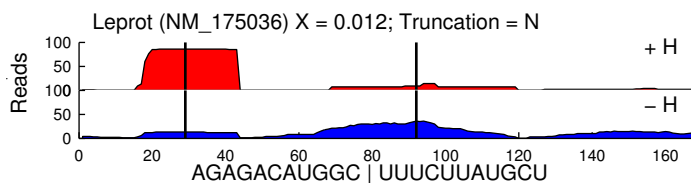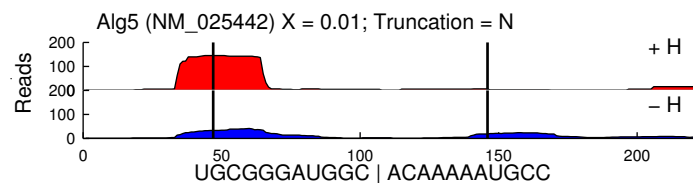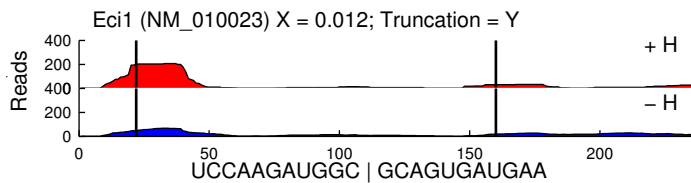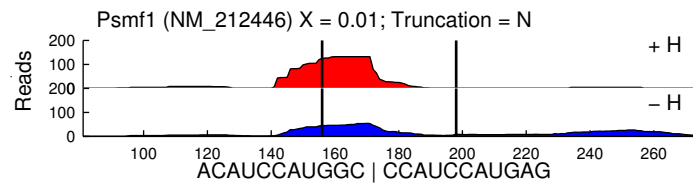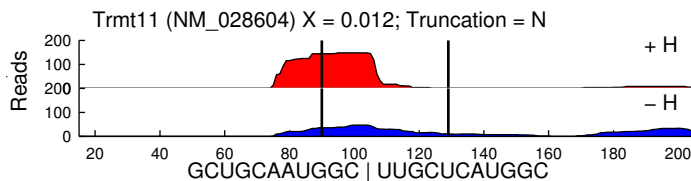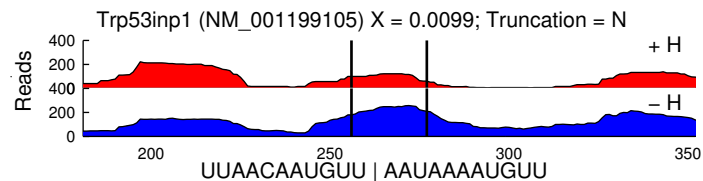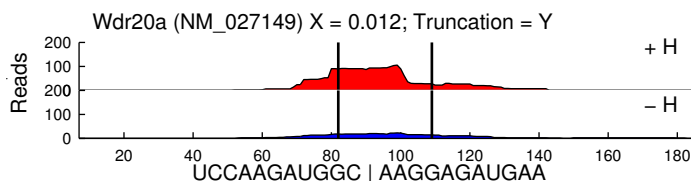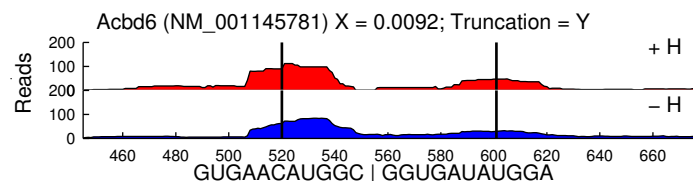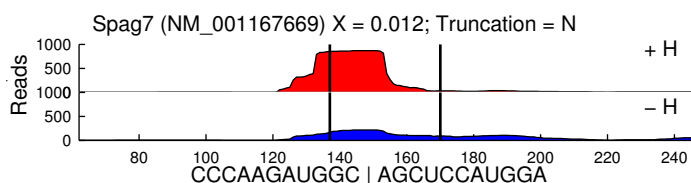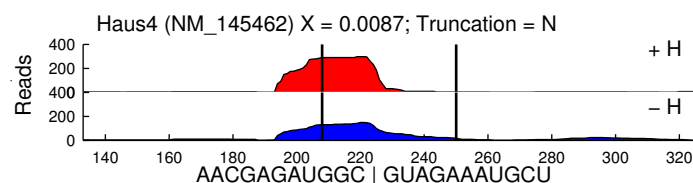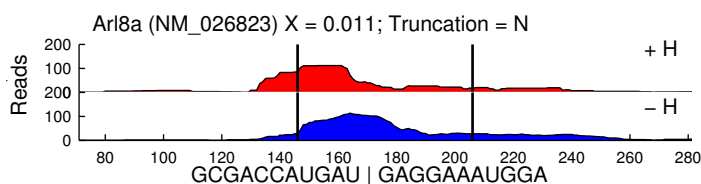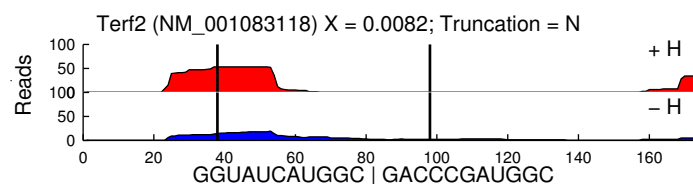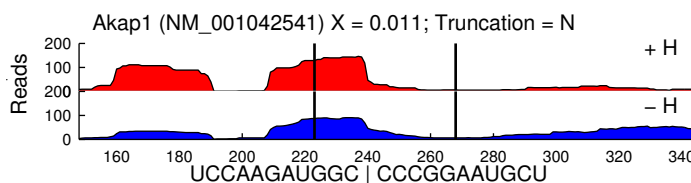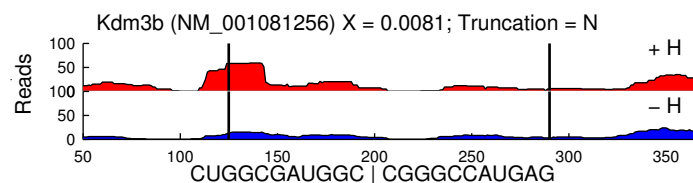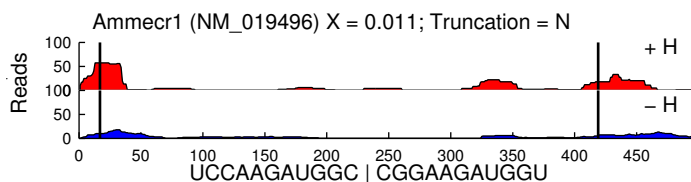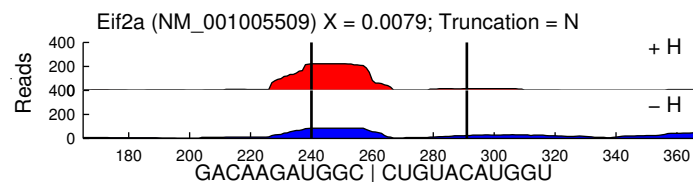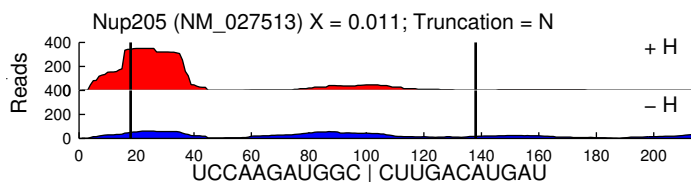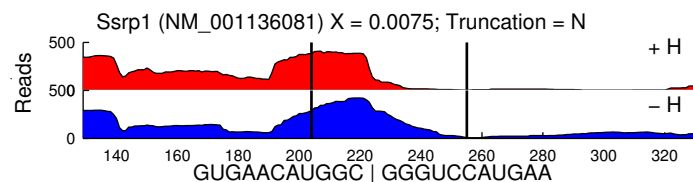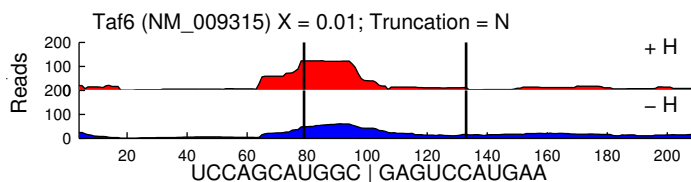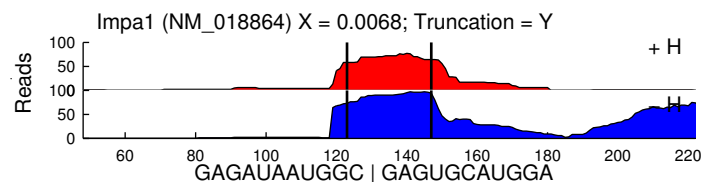

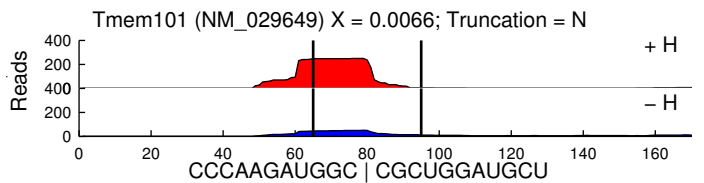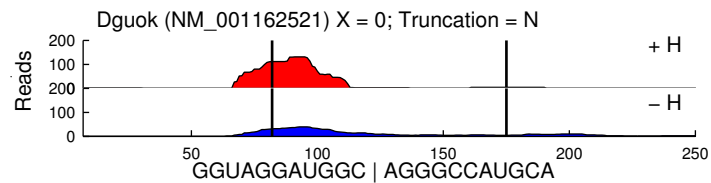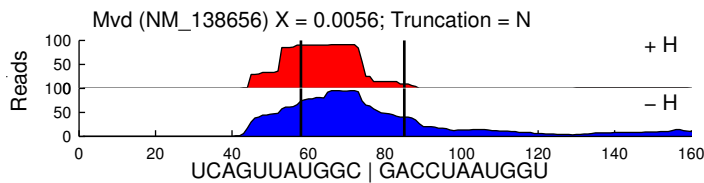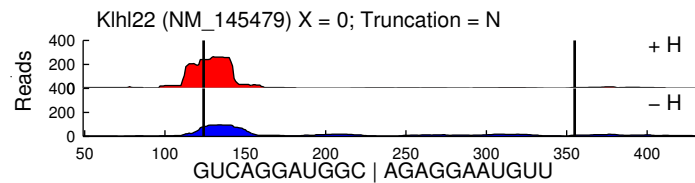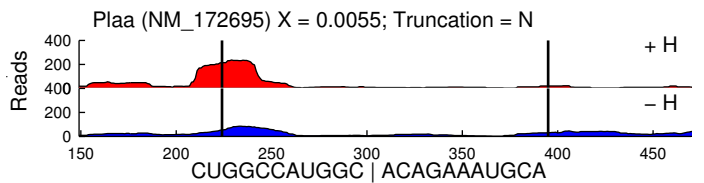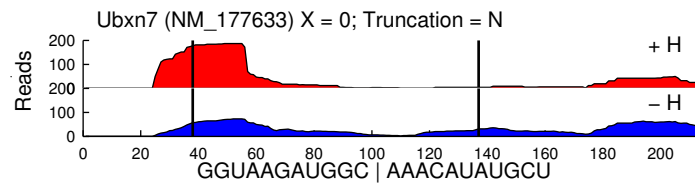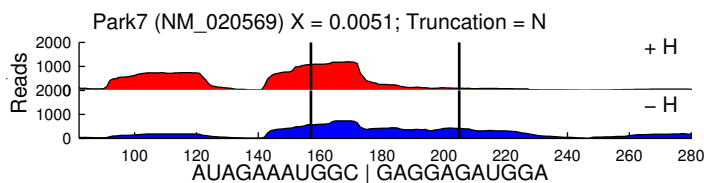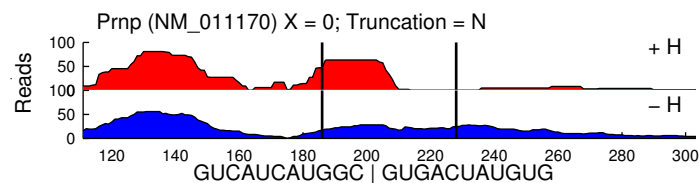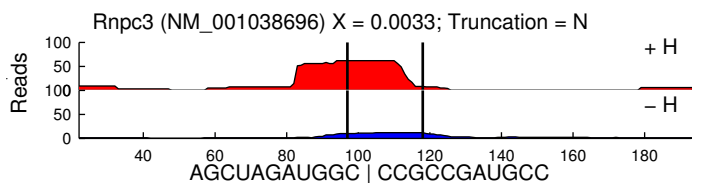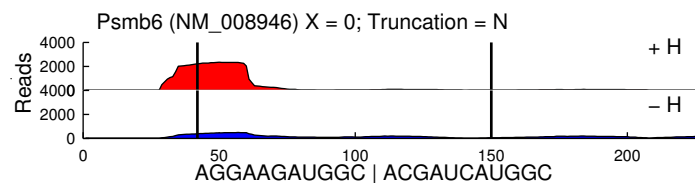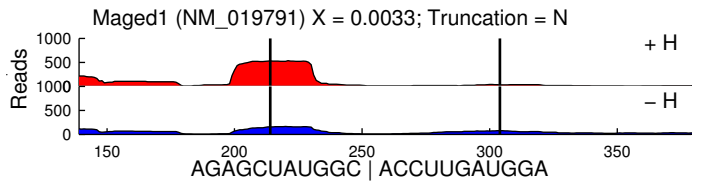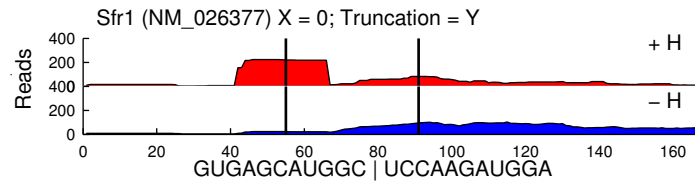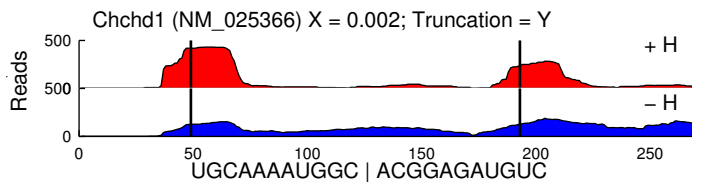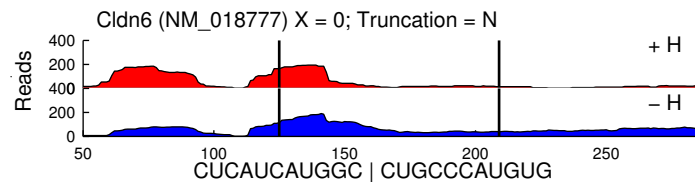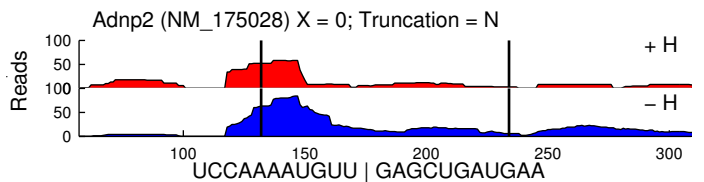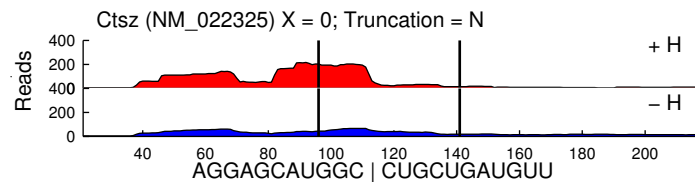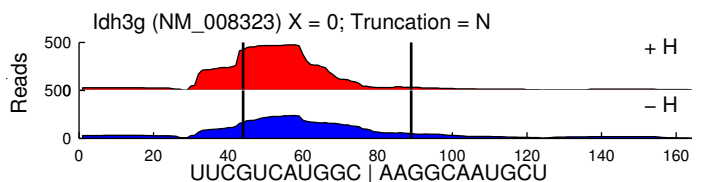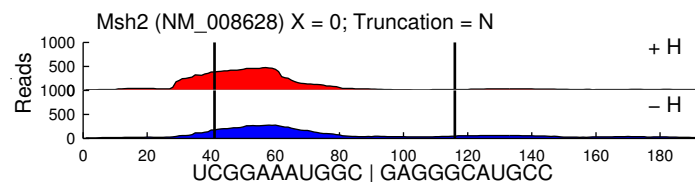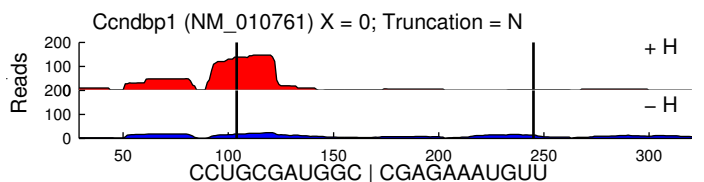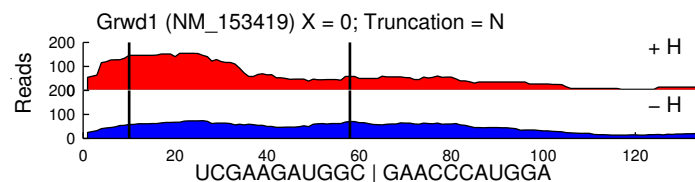

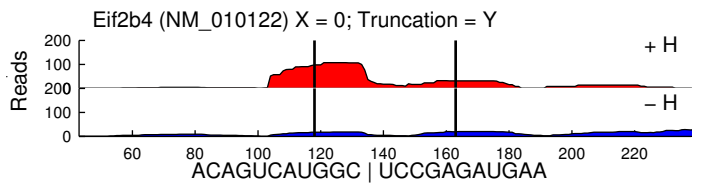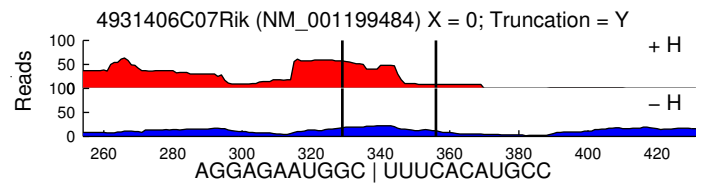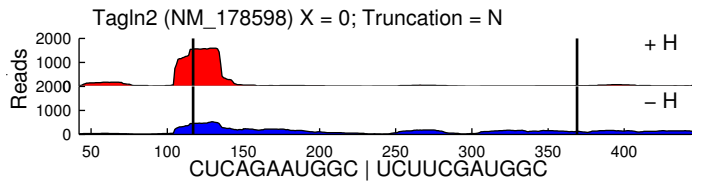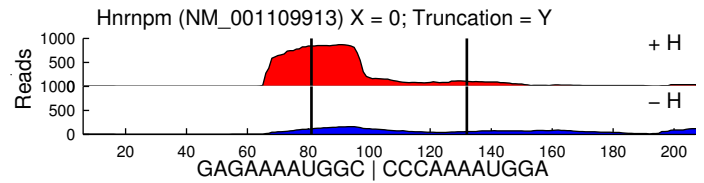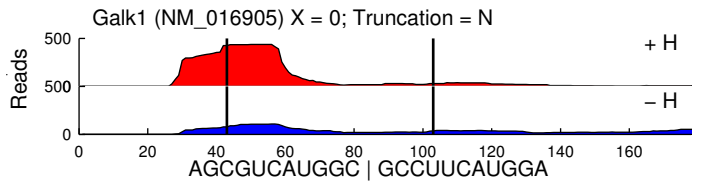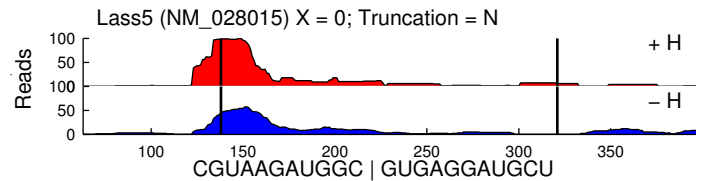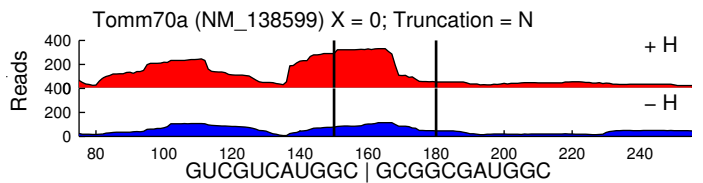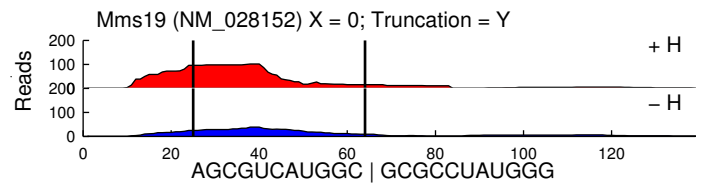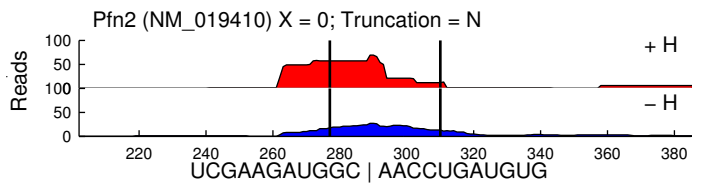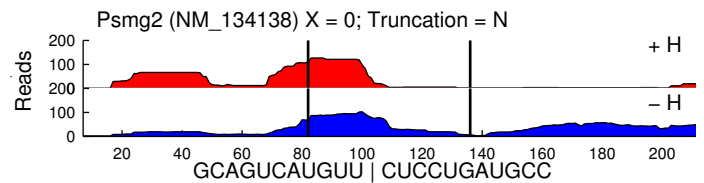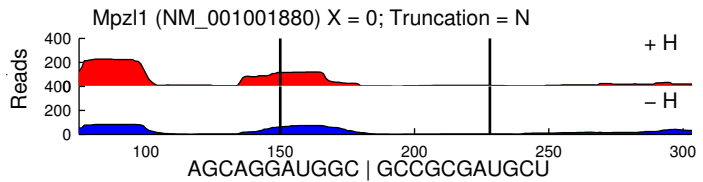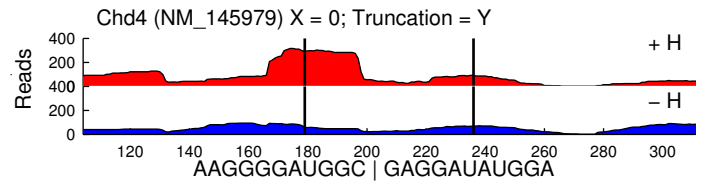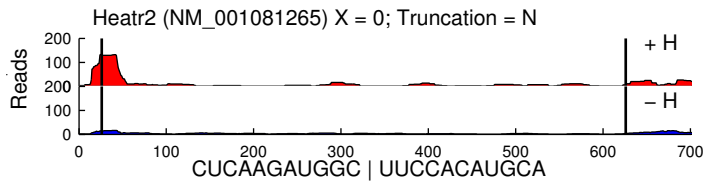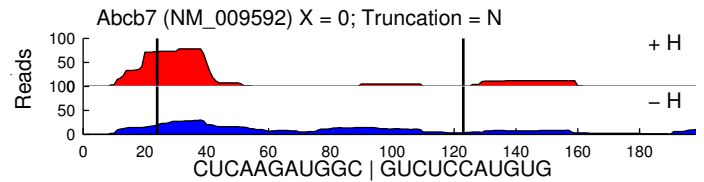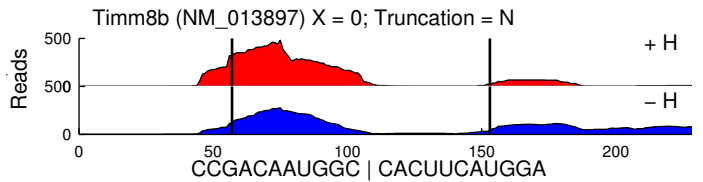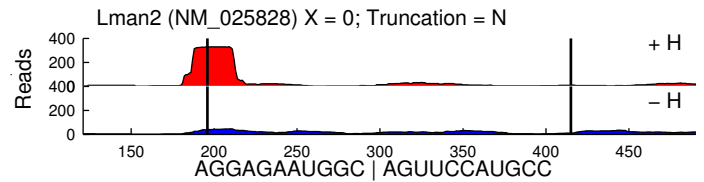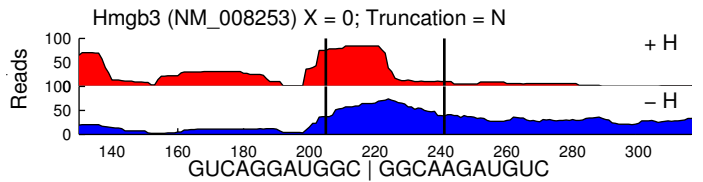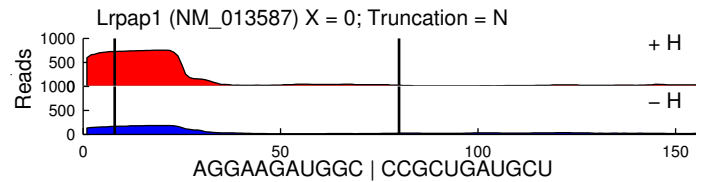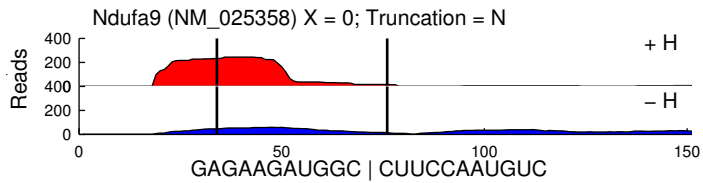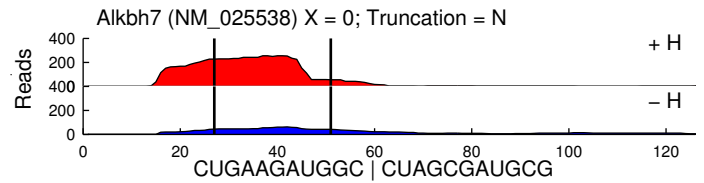

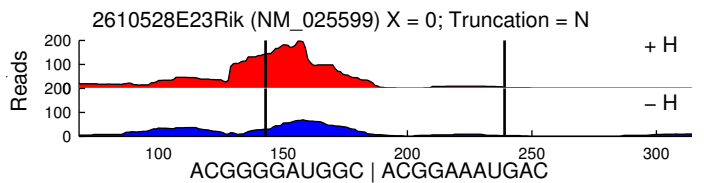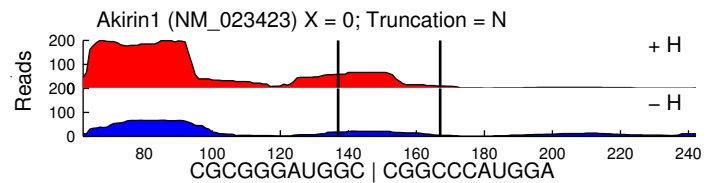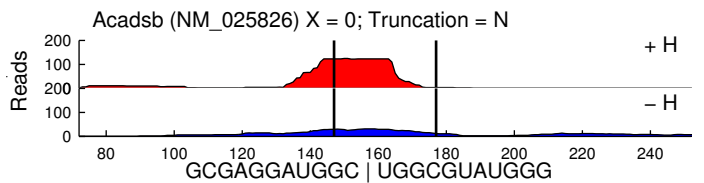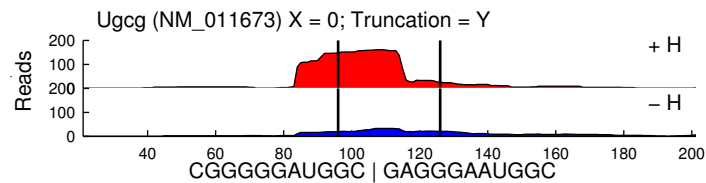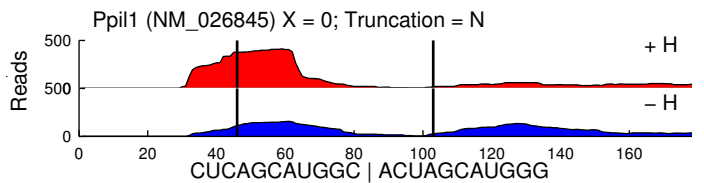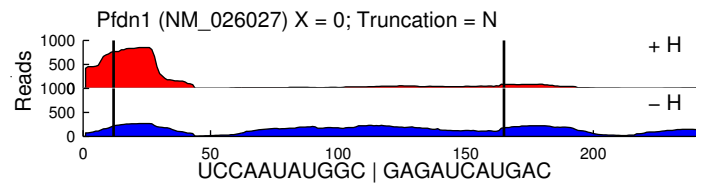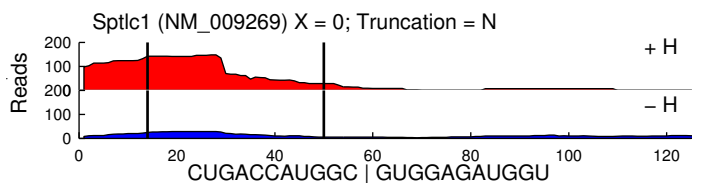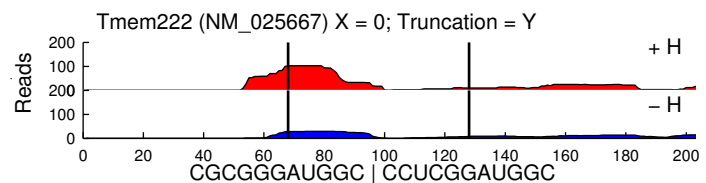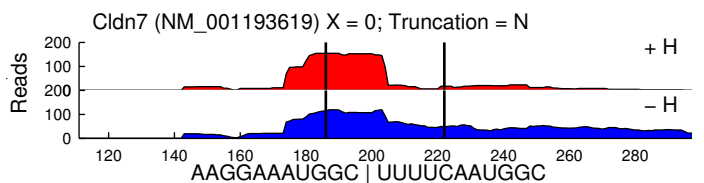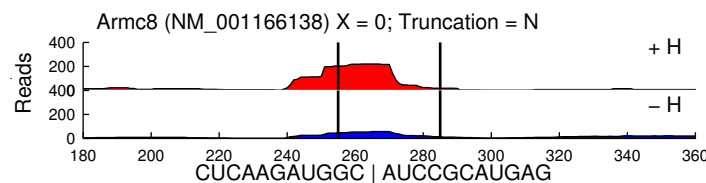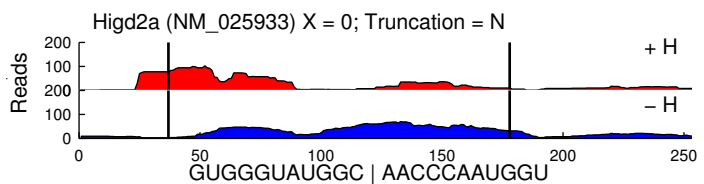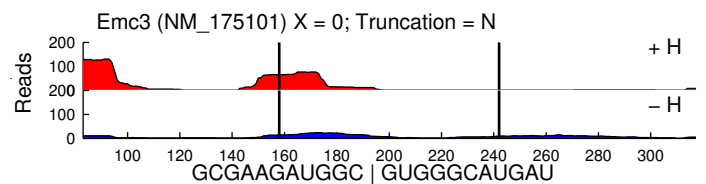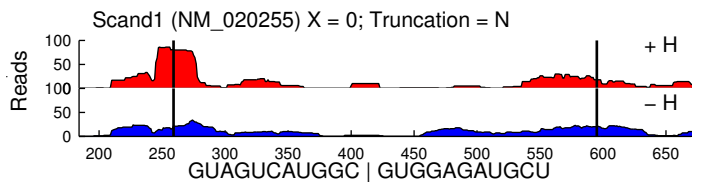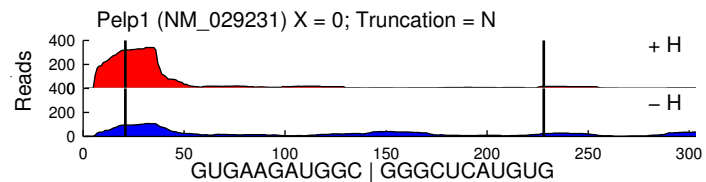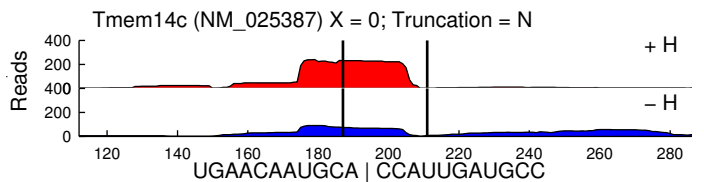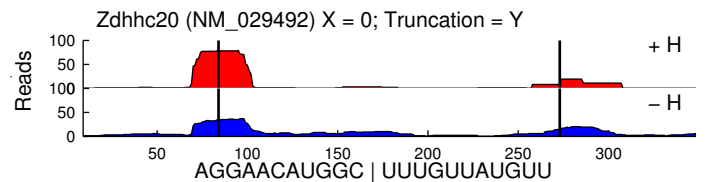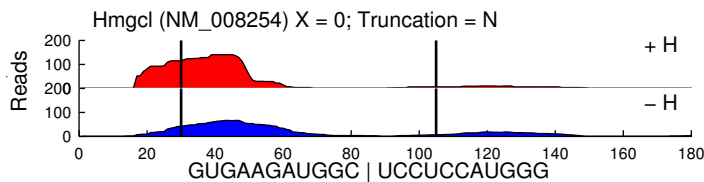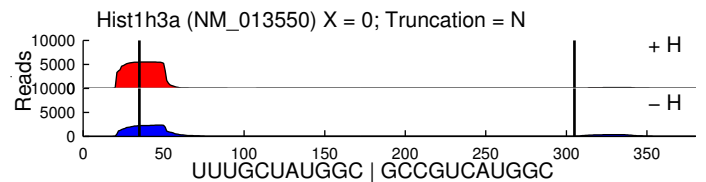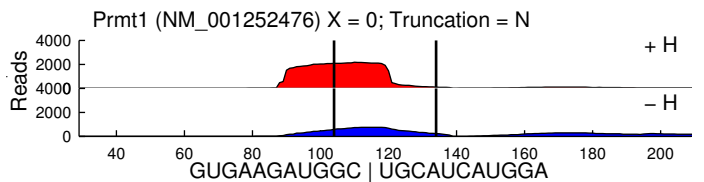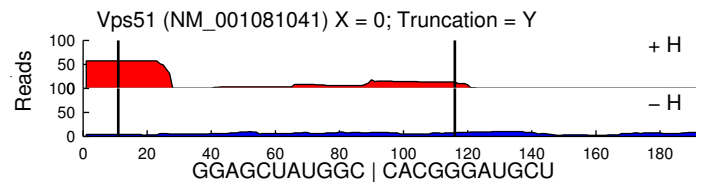

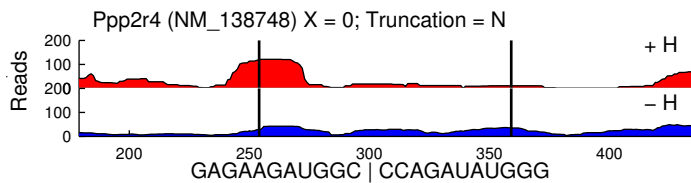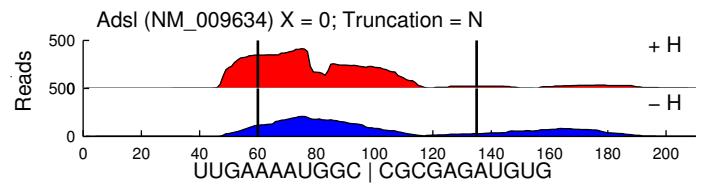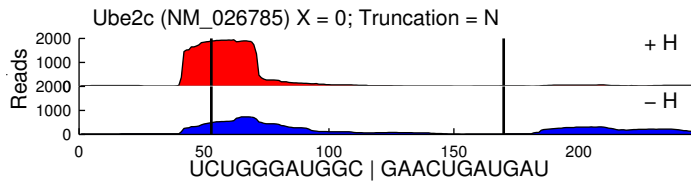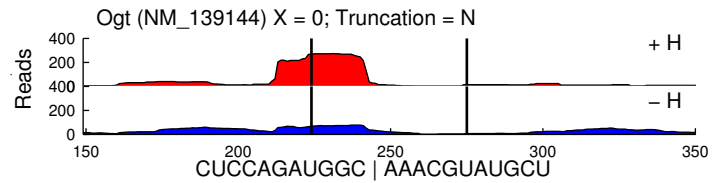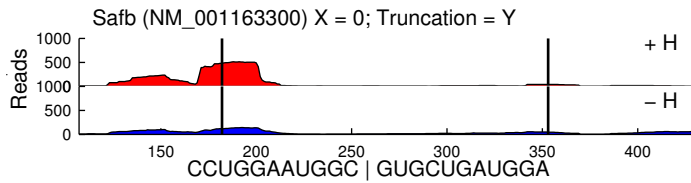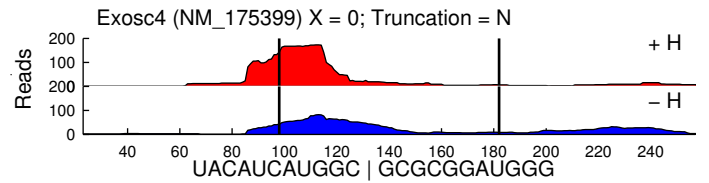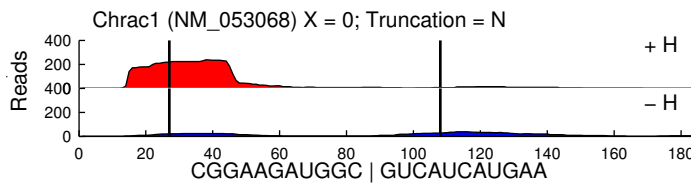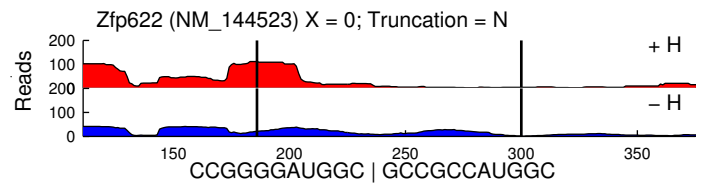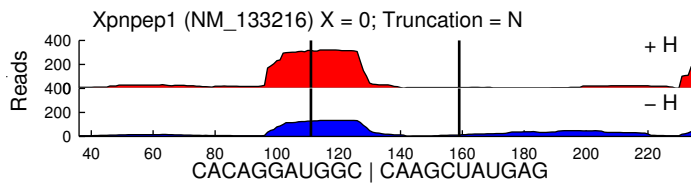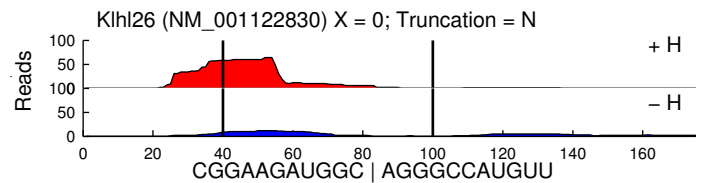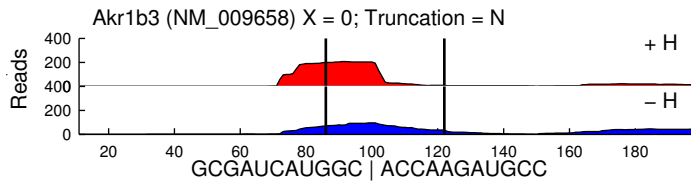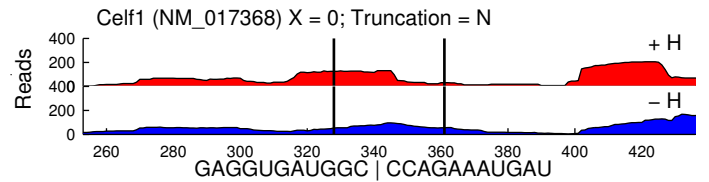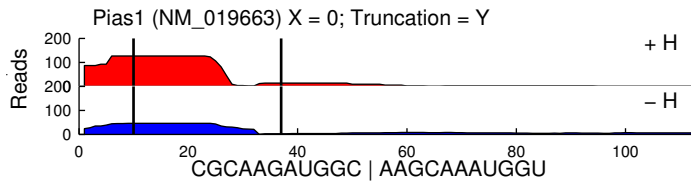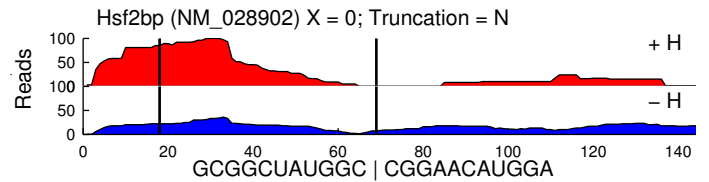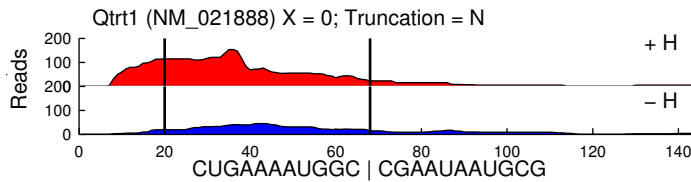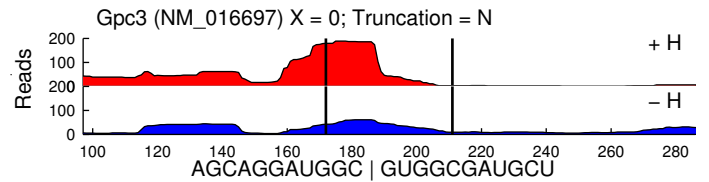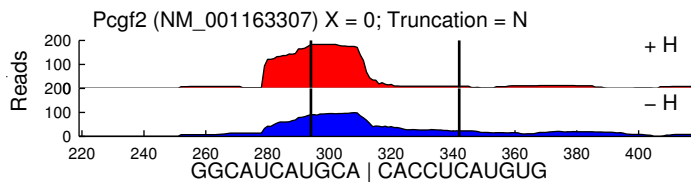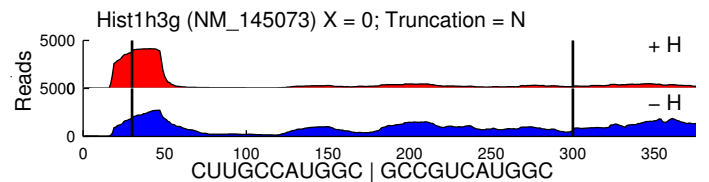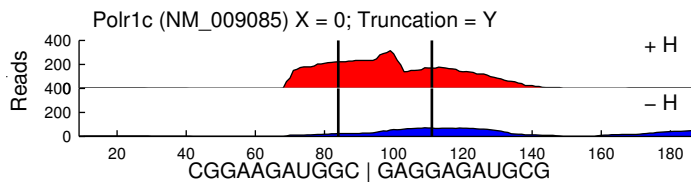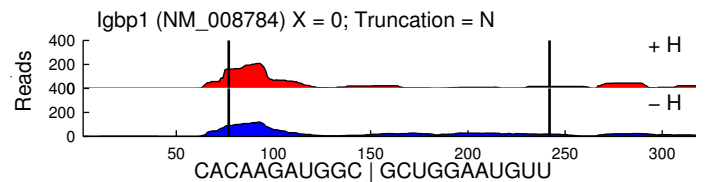

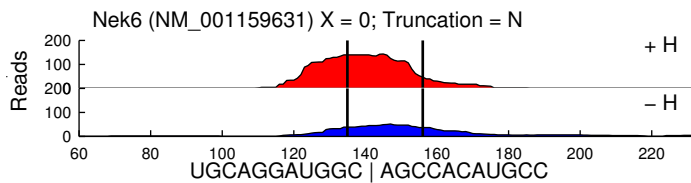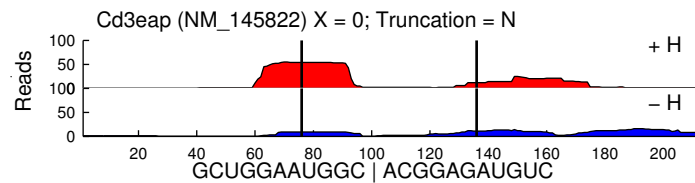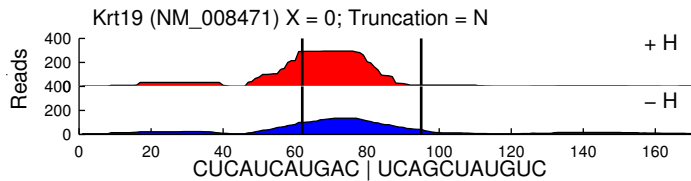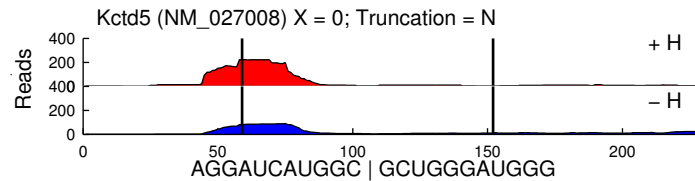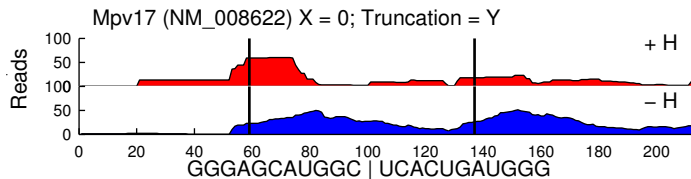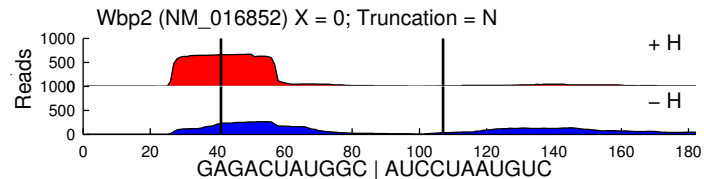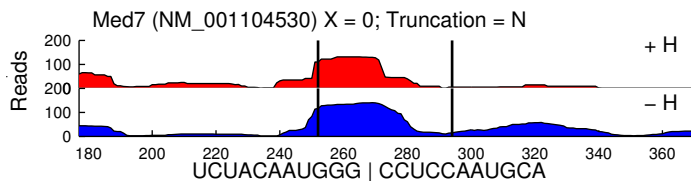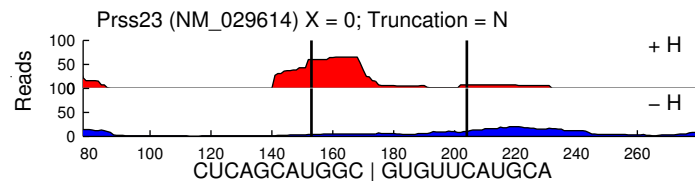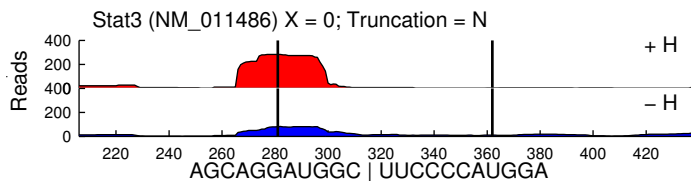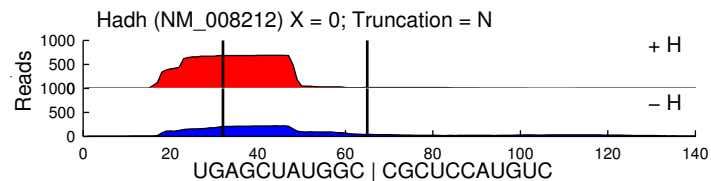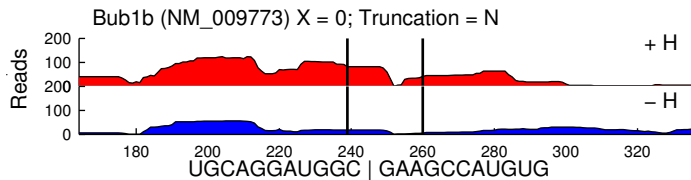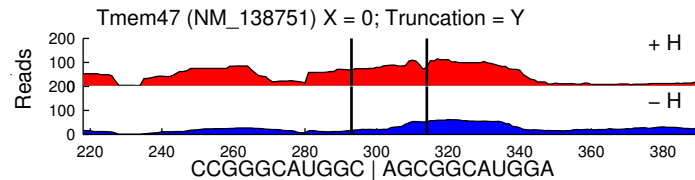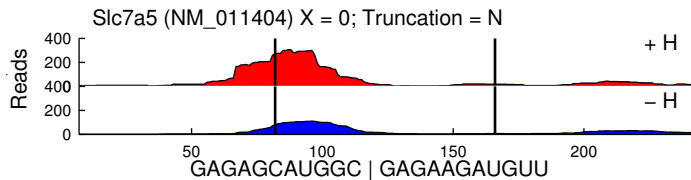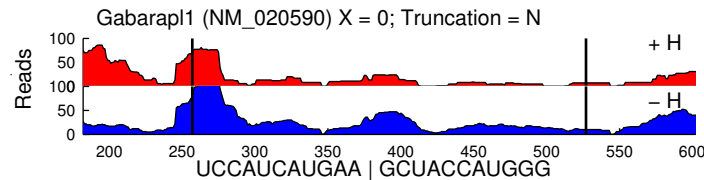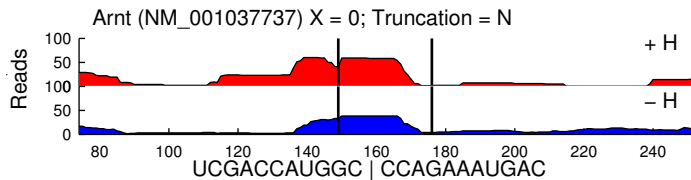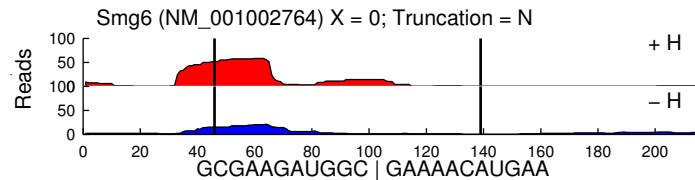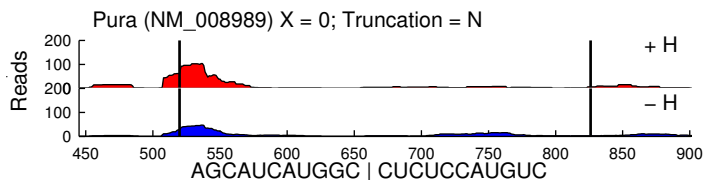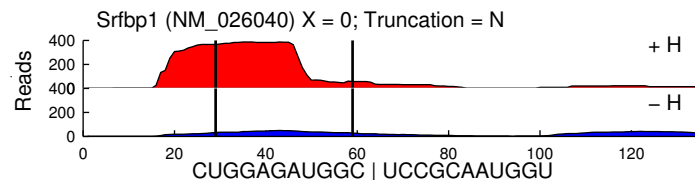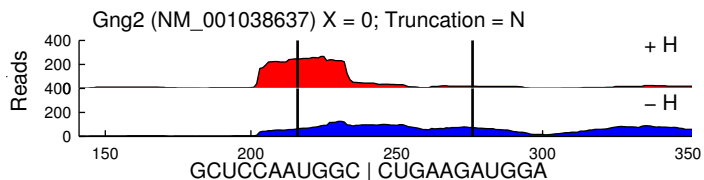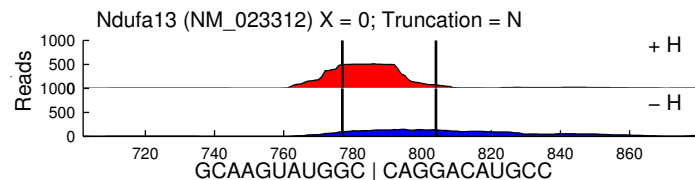

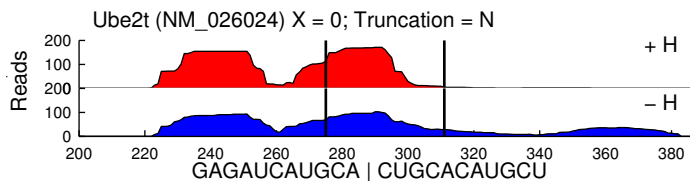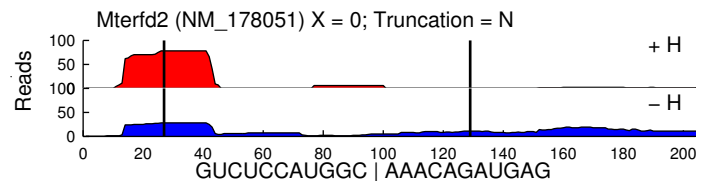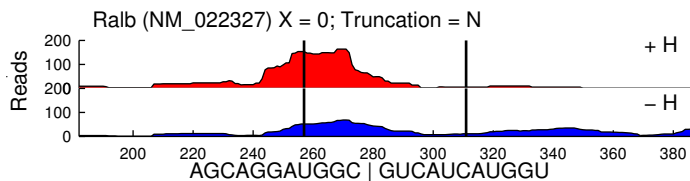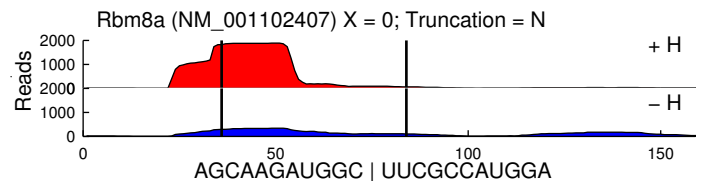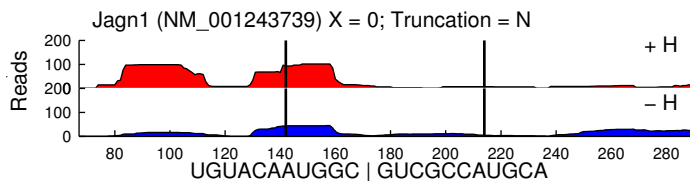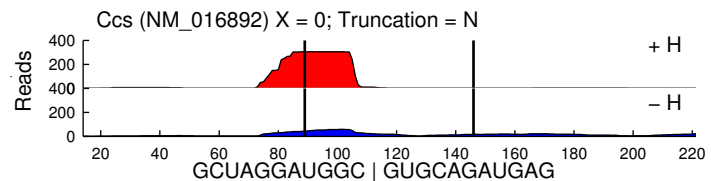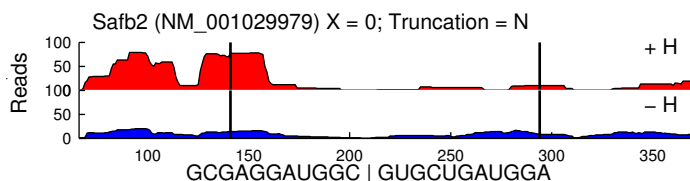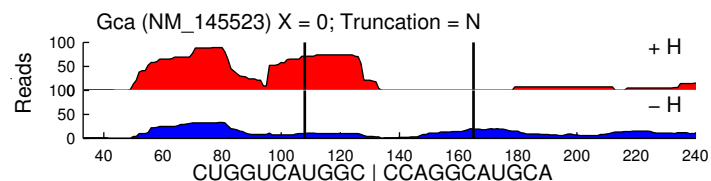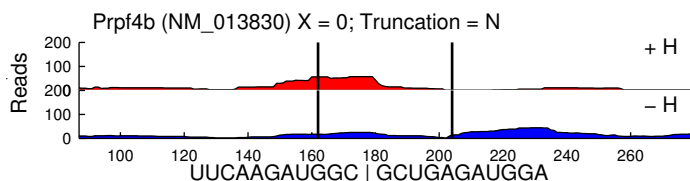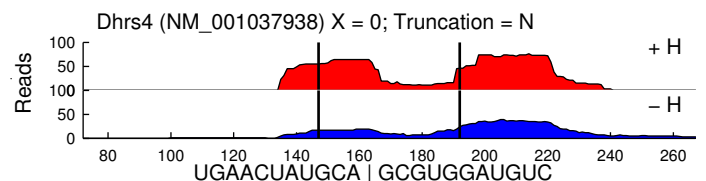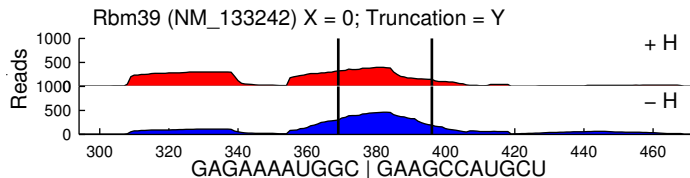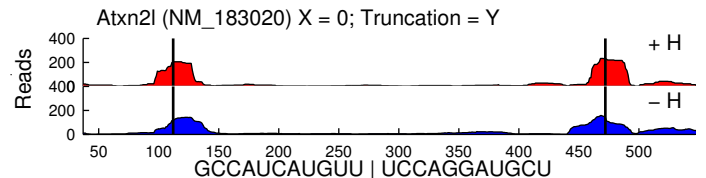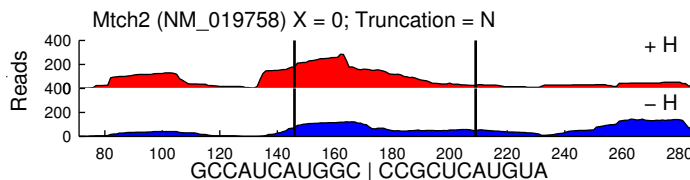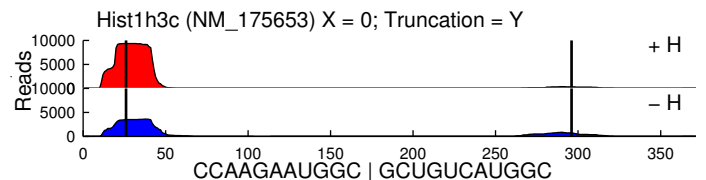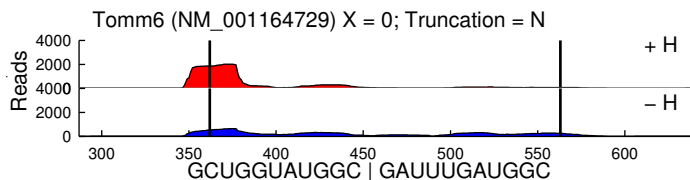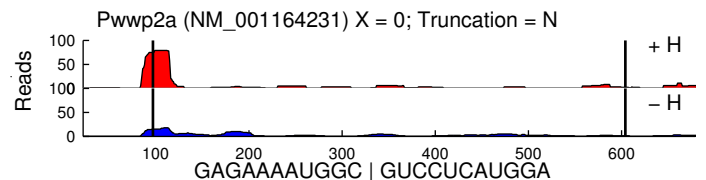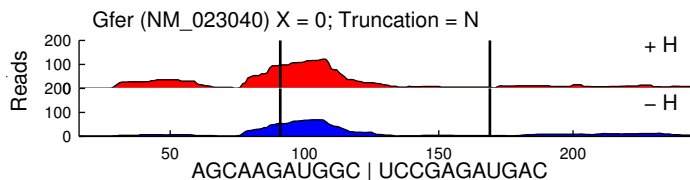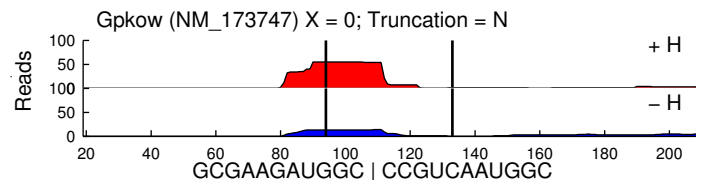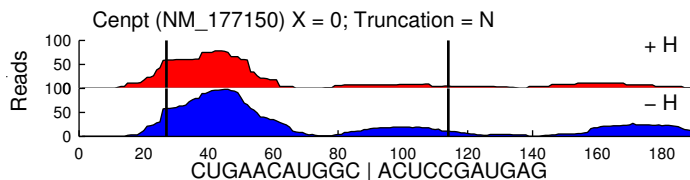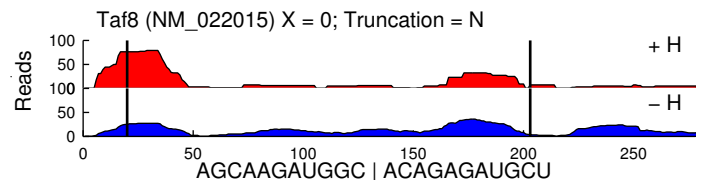

Supplement: Supplementary file 2 — Supplementary Figure S7 [file msb0010-0748-SD2.pdf]
